# Functional screening of TCR-like antibodies using STAR-T cell library for cancer immunotherapy

**DOI:** 10.1038/s44321-026-00455-z

**Published:** 2026-06-08

**Authors:** Yi Li, Daosheng Huang, Chang Liu, Zhixiao Zhou, Yilu Song, Dongmei Yang, Wei Rui, Zaopeng Yang, Li Yu, Chenliang Wang, Zheyu Zheng, Jiasheng Wang, Yang-Xin Fu, Xin Lin

**Affiliations:** 1 School of Basic Medical Sciences, Tsinghua University, Beijing, 100084 China; 2 Changping Laboratory, Beijing, 102206 China; 3 Tsinghua-Peking Center for Life Sciences, Beijing, 100084 China; 4 BriSTAR Immunotech, Beijing, 102206 China; 5 State Key Laboratory of Molecular Oncology, Beijing, 100084 China

**Keywords:** Biotechnology & Synthetic Biology, Cancer, Immunology

## Abstract

Adoptive T-cell therapies engineered with T-cell receptors (TCRs) or TCR-like antibodies have shown considerable promise in cancer immunotherapy. However, identifying tumor antigen-specific TCR-like antibodies, particularly against human leukocyte antigen-presented neoantigens, remains challenging. Here, we present a function-based, rather than affinity-based, antibody screening platform utilizing Synthetic T-cell receptor and Antigen Receptor (STAR)-T cell libraries. We found that antigen engagement in STAR-T cells triggers synchronous receptor endocytosis and T-cell activation, and we integrated these paired processes into an Endocytosis-Activation (E-A) functional readout for antibody screening. Applying E-A functional screening, we rapidly identified multiple nanobodies targeting the cell-surface antigen CD22 as well as the intracellular neoantigen P53^R175H^. STAR-T cells engineered with these nanobodies mediated potent anti-tumor efficacy both in vitro and in vivo. Furthermore, this platform yielded nanobodies that can be directly reformatted into other therapeutic modalities, including chimeric antigen receptors and bispecific antibodies, while maintaining cytotoxic function. Overall, the E-A screening platform links antibody discovery directly to T-cell function, providing a robust approach for identifying therapeutic antibodies, especially neoantigen-specific nanobodies, for T cell-based cancer immunotherapy.

## Synopsis

STAR-T cells exhibit antigen-dependent receptor endocytosis coupled with activation, enabling a functional screening platform that directly links antibody discovery to T-cell activity for rapid isolation of therapeutic TCR-like antibodies against both surface antigens and HLA-presented neoantigens.

- Antigen engagement in STAR-T cells triggers synchronous receptor endocytosis and CD69 upregulation, which together form a dual-parameter Endocytosis-Activation (E-A) functional readout.
- E-A functional screening identified CD22-specific nanobodies from a > 10⁶-diversity T-cell library, with lead candidate CD22-1 VHH exhibiting nanomolar affinity and potent in vivo anti-tumor efficacy in xenograft models.
- The platform identified multiple P53R175H/HLA-A*02:01-specific VHHs, with P53-1 VHH STAR-T cells demonstrating efficient tumor suppression in both KMS26 and K562 tumor models.
- E-A screening outperformed conventional phage display, achieving >10-fold enrichment of functional clones while eliminating clones defective for mammalian expression.
- Identified nanobodies were directly reformatted into STARs, CARs (BBz/28z), and bispecific antibodies, all retaining potent antigen-specific cytotoxicity in vitro and in vivo.

## Abstract (web summary)

STAR-T cells exhibit antigen-dependent receptor endocytosis coupled with activation, enabling a functional screening platform that directly links antibody discovery to T-cell activity for rapid isolation of therapeutic TCR-like antibodies against both surface antigens and HLA-presented neoantigens.

## Graphical abstract

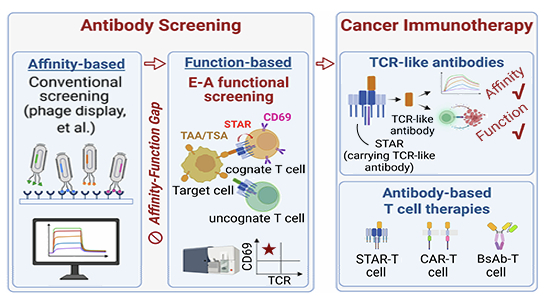

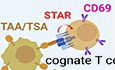

The paper explained **Problem**

Adoptive T-cell therapies using T-cell receptors (TCRs) or TCR-like antibodies have shown great promise for cancer immunotherapy. However, discovering antibodies that recognize tumor-specific antigens—particularly neoantigens presented by human leukocyte antigen (HLA) molecules—remains a major challenge. Traditional affinity-based screening methods (such as phage display) often yield binders that lack functional activity in T cells, leading to high false-positive rates and lengthy downstream validation. There is an urgent need for a screening platform that directly links antibody discovery to T-cell function.

**Results**

We observed that antigen engagement in Synthetic T-cell receptor and Antigen Receptor (STAR)-T cells triggers two synchronous events: receptor endocytosis and T-cell activation. Leveraging this finding, we developed an endocytosis-activation (E-A) functional screening platform that integrates these paired processes into a unified readout. Using this approach, we rapidly identified multiple TCR-like nanobodies targeting the cell-surface antigen CD22 as well as the intracellular neoantigen P53^R175H^ presented by HLA-A*02:01. STAR-T cells engineered with these nanobodies mediated potent anti-tumor efficacy both in vitro and in vivo. Furthermore, the identified nanobodies were directly reformatted into other therapeutic modalities—including chimeric antigen receptors (CARs) and bispecific antibodies—while maintaining their cytotoxic functions.

**Impact**

This study establishes a function-based, rather than affinity-based, antibody screening platform that directly links antibody discovery to T-cell activity. By integrating endocytosis and activation into a dual-functional readout, the E-A screening platform efficiently identifies therapeutic antibodies against both tumor-associated antigens and tumor-specific neoantigens, with minimal off-target effects. This approach addresses the affinity-function gap that has long hindered antibody discovery for T-cell-based cancer immunotherapy, offering a robust and scalable strategy for developing next-generation cancer immunotherapies.

## Introduction

Antibody therapeutics and T cell-based therapies are profoundly transforming cancer treatment (Alpaugh and Cicchetti, 2019; Crescioli et al, 2024; Goydel and Rader, 2021; Rosenberg and Restifo, 2015; Zinn et al, 2023). Yet, discovering T-cell receptor (TCR)-like antibodies that recognize diverse tumor antigens, particularly human leukocyte antigen (HLA)-presented tumor-specific neoantigens, remains difficult (He et al, 2019; Westcott et al, 2021). The core challenge is that biochemical binding affinity does not reliably predict T-cell functionality in a physiological context, especially for peptide-HLA complexes whose presentation abundance is low and conformation can be unstable (Yamamoto et al, 2019). This affinity-function gap leads to lengthy downstream validation and a high false-positive rate, which hinders antibody discovery for T-cell-based cancer immunotherapy.

Conventional antibody screening approaches have advanced the field but are constrained by this fundamental limitation. Hybridoma and in vitro display methods (for example, phage and yeast display) efficiently screen binders, while their affinity-based selection can be decoupled from T-cell functionality (Jakobovits et al, 2007; Kanamori et al, 2014; Kretzschmar and von Ruden, 2002; Miller et al, 2008; Saeed et al, 2017). Microfluidic single-cell platforms increase throughput and facilitate recovery of rare clones, but typically rely on proxy readouts and require specialized instrumentation, thereby impeding broad deployment (Fitzgerald and Leonard, 2017; Pedrioli and Oxenius, 2021; Shapiro et al, 2023; Shembekar et al, 2018; Sun et al, 2022). Despite recent advances, AI-guided de novo antibody design yields modest practical hit rates (Bennett et al, 2024), while remaining tied to yeast display for high-throughput experimental validation and affinity maturation (Cao et al, 2022). This reliance likely reflects the limited scale and uneven quality of training datasets that explicitly capture antibody recognition of peptide-HLA complexes.

Cell-based functional screening seeks to further bridge the affinity-function gap by directly linking antigen recognition to T-cell functionality. For instance, chimeric antigen receptor (CAR)-T cells engineered with antibody libraries were applied for antibody screening, using T-cell activation as a functional readout (Alonso-Camino et al, 2013; Bloemberg et al, 2020; Ma et al, 2021). However, the high tonic signaling and limited sensitivity of CARs can confound specificity and mask genuine antigen-dependent activation (Labanieh and Mackall, 2023). Alternatively, while TCR trogocytosis has been utilized for cognate TCR identification, it is not readily compatible with antibody-fragment discovery (Li et al, 2019). Informed by these limitations and our previous work, which demonstrated that antibody-based Synthetic T-cell receptor and Antigen Receptor (STAR) achieves TCR-like high antigen sensitivity (Huang et al, 2024) with minimal tonic signaling (Liu et al, 2021b), we hypothesized that the STAR architecture could serve as a superior platform to screen antibody libraries in T cells with low false-positive rate and high efficiency, thereby enriching functional antibodies directly for antibody-based T-cell therapies.

Here, we define an endocytosis-activation (E-A) functional index in STAR-T cells as a unified metric that integrates two synchronous, orthogonal events upon antigen engagement: receptor endocytosis and early T-cell activation, as two independent facets of TCR signaling (Charpentier and King, 2021; Cibrian and Sanchez-Madrid, 2017; Evnouchidou et al, 2022). By applying E-A functional index-based screening, we rapidly isolated antigen-specific TCR-like nanobodies from STAR-T cell libraries. These nanobodies, which are the variable domains of heavy-chain-only antibodies (HCAbs), also known as VHHs, target the canonical cell-surface antigen CD22 and the HLA-A*02:01-presented P53^R175H^ neoantigen. These nanobodies conferred potent anti-tumor efficacy to STAR-T cells both in vitro and in vivo. Furthermore, they were directly reformatted into diverse modalities, including CARs and bispecific antibodies, confirming their versatile therapeutic utility. Together, by integrating a physiologically faithful, dual-readout functional index into STAR-T cell libraries, this work provides a versatile and scalable platform for discovering therapeutic antibodies against tumor antigens, particularly neoantigens, thereby advancing T cell-based cancer immunotherapy.

## Results

### Cognate antigen triggers STAR endocytosis and activation of STAR-T cells in an antigen-specific manner

**Figure EV1:**
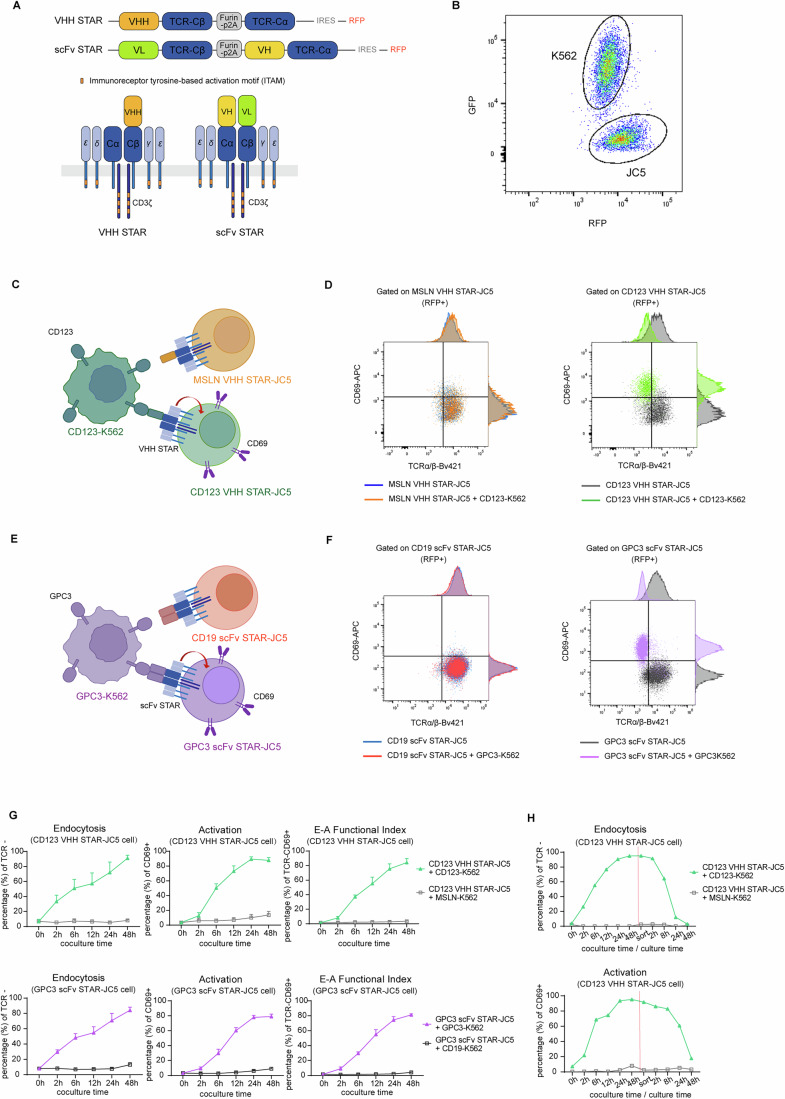
Supporting characterization of the E-A functional index in STAR-T cells using VHH and scFv formats. (**A**) Schematic structures of VHH STAR and scFv STAR constructs, each followed by an internal ribosome entry site (IRES) and a red fluorescent protein (RFP). (**B**) Flow cytometry gating for JC5 and K562 cell identification. JC5 cells were identified by RFP expression; K562 cells were identified by GFP expression. (**C**,** E**) Schematics of the co-culture assays of the VHH STAR-JC5 cells and scFv STAR-JC5 cells with swapped target antigens. For the VHH STAR group (**C**), K562 target cells were transduced to express tumor membrane antigen CD123 and co-cultured with JC5 cells transduced with cognate CD123 VHH STAR or non-cognate MSLN VHH STAR control. For scFv STAR group (**E**), K562 target cells were transduced to express tumor membrane antigen GPC3 and co-cultured with JC5 cells transduced with cognate GPC3 scFv STAR or non-cognate CD19 scFv STAR control. Same coloring indicates cognate antigen-antibody pairs. Red arrows represent the endocytosis process. Purple homodimers represent the surface molecule CD69. (**D**,** F**) Flow cytometry analysis of surface STAR (TCRα/β) and CD69 expression on RFP^+^ JC5 cells in (**C**,** E**) after 24 h co-culture. (**G**) Kinetics of STAR endocytosis (TCRα/β^–^), CD69 activation (CD69^+^), and the dual-parameter E-A functional index (TCRα/β^–^CD69^+^) in RFP^+^ JC5 cells co-cultured with cognate or non-cognate target cells. (**H**) Reversibility of the E-A functional index. STAR endocytosis and CD69 activation were analyzed in RFP^+^ JC5 cells at the indicated time points before and after removal of antigen stimulation (via FACS sorting at 48 h). The E:T ratio used in all co-culture experiments is 1:1. Data in (**D**) and (**F**–**H**) are representative of three independent experiments. Data in (**G**) are presented as mean ± SEM.

STAR is a synthetic double-chain TCR-based chimeric receptor, which combines the antigen-recognition domain of antibody with constant regions of TCR that engage endogenous CD3 signaling machinery. Based on our previous works, STARs exhibit lower tonic signaling and higher sensitivity than conventional CARs (Fig. EV1A) (Burton et al, 2023; Huang et al, 2024; Liu et al, 2021b; Wang et al, 2022). Therefore, STAR-T cells were selected as the preferred platform for antibody screening in this study.

**Figure 1:**
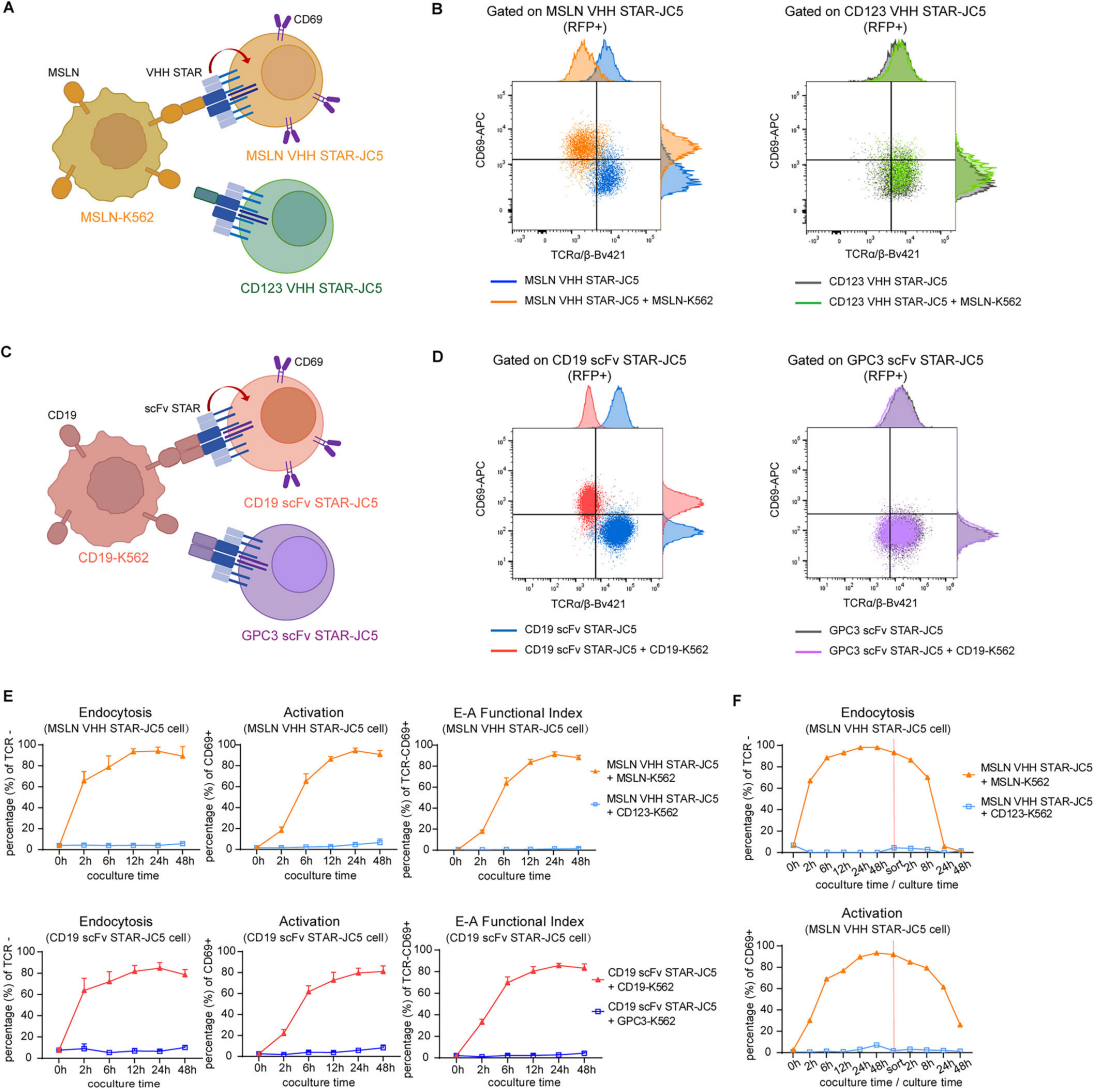
Characterization of the E-A functional index in STAR-T cells. (**A**,** C**) Schematics of the co-culture assays of the VHH STAR-JC5 cells and scFv STAR-JC5 cells. For the VHH STAR group (**A**), K562 target cells were transduced to express tumor membrane antigen MSLN and co-cultured with JC5 cells transduced with cognate MSLN VHH STAR or non-cognate CD123 VHH STAR control. For scFv STAR group (**C**), K562 target cells were transduced to express tumor membrane antigen CD19 and co-cultured with JC5 cells transduced with cognate CD19 scFv STAR or non-cognate GPC3 scFv STAR control. Same coloring indicates cognate antigen-antibody pairs. Red arrows represent the endocytosis process. Purple homodimers represent surface molecular CD69. (**B**,** D**) Flow cytometry analysis of surface STAR (TCRα/β) and CD69 expression on RFP^+^ JC5 cells from (**A**,** C**) after 24 h co-culture. (**E**) Kinetics of STAR endocytosis (TCRα/β^–^), CD69 activation (CD69^+^), and the dual-parameter E-A functional index (TCRα/β^–^CD69^+^) in RFP^+^ JC5 cells co-cultured with cognate or non-cognate target cells. (**F**) Reversibility of the E-A functional index. STAR endocytosis and CD69 activation were analyzed in RFP^+^ JC5 cells at the indicated time points before and after removal of antigen stimulation (via FACS sorting at 48 h). The E:T ratio used in all co-culture experiments is 1:1. Data in (**B**) and (**D**–**F**) are representative of three independent experiments. Data in (**E**) are presented as mean ± SEM. Source data are available online for this figure.

To explore antigen-specific behaviors of STAR-T cells, we first established cell lines expressing cognate antigen-STAR pairs. These included K562 cells expressing the membrane antigens mesothelin (MSLN) or CD123, and a TCR-deficient Jurkat cell line (JC5) expressing cognate STARs carrying either MSLN-specific or CD123-specific VHHs. To identify suitable indicators of T cell function upon antigen-specific stimulation, we co-incubated K562 cells with either cognate or non-cognate VHH STAR-JC5 cells and evaluated surface marker expression on JC5 cells via flow cytometry (Figs. 1A and EV1B,C). Notably, we observed a near-complete loss of surface STAR receptor on JC5 cells, as detected by TCRα/β antibody staining, along with a concomitant upregulation of the classical lymphocyte activation marker CD69, upon 24-hour stimulation with cognate antigens (Figs. 1B and EV1D). To assess the generality of these responses, we repeated the experiments using cognate antigen–scFv STAR pairs, including K562 cells expressing the membrane antigen CD19 or glypican 3 (GPC3), and JC5 cells expressing their cognate scFv-based STARs. Consistent with the VHH groups, similar patterns of receptor endocytosis and CD69 activation were observed in the scFv groups (Figs. 1C,D and EV1E,F). These results collectively demonstrate that STAR-JC5 cells exhibit antigen-specific STAR endocytosis and CD69 activation.

**Figure EV2:**
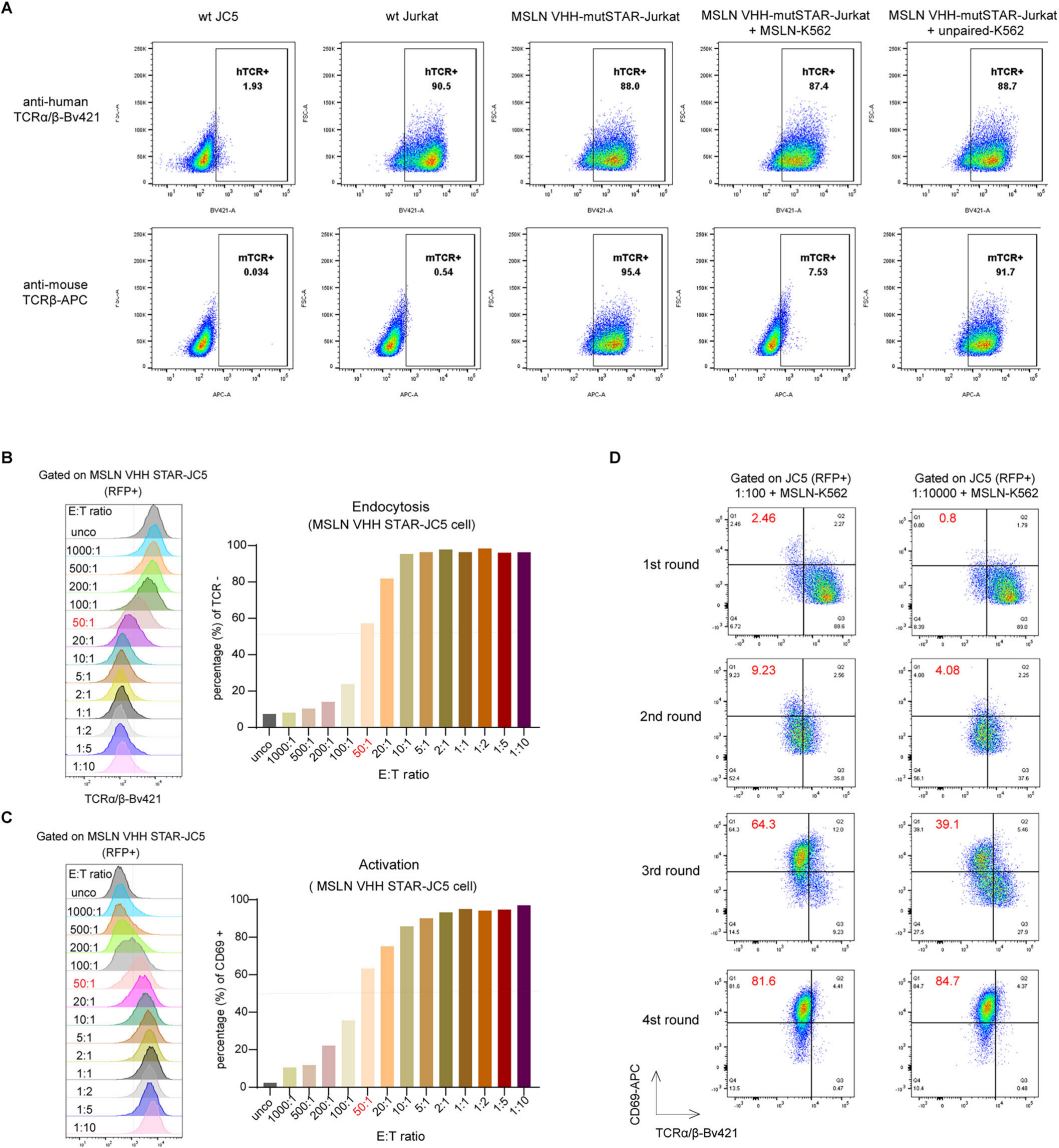
Sensitivity and specificity of the E-A functional index in STAR-T cells. (**A**) Flow cytometry analysis of TCR endocytosis in different T cell populations before and after co-culture with target cells. Cells were gated first by FSC for single cells, followed by staining with anti-human TCRα/β-BV421 (endogenous TCR) and anti-mouse TCRβ-APC (mutSTAR). Data are representative of three independent experiments. (**B**,** C**) Flow cytometry analysis of STAR endocytosis and CD69 activation in MSLN VHH STAR-JC5 cells after 24 h co-culture with target MSLN-K562 cells at different E:T ratios. “Unco” indicates JC5 cells cultured without target cells. (**D**) FACS plots of RFP^+^ JC5 cells from 1:100 or 1:10,000 model libraries after each round of functional screening against MSLN-K562 cells. Panel D is also shown in Fig. 2B. Data in (**B**, **C**) are representative of three independent experiments.

We further monitored the kinetics of STAR endocytosis and CD69 expression in JC5 cells following co-culture with target cells at different time points. We found that STAR endocytosis occurred more rapidly than CD69 expression within the first 2 h and both indicators subsequently showed synchronous dynamics over time (Figs. 1E and EV1G). Based on these findings, we integrated endocytosis and activation into a combined functional readout termed “E-A functional index”, which represents a TCR-negative and CD69-positive (TCR^−^CD69^+^) state. Upon removal of antigenic stimulation after 48 h of co-culture, endocytosed STAR receptors rapidly recycled to the T cell surface within 24 h, while CD69 expression returned to baseline levels more quickly (Figs. 1F and EV1H). In addition, we expressed a mutant STAR (Liu et al, 2021b) (mutSTAR) in wild-type (wt) Jurkat cells and confirmed that TCR endocytosis depends specifically on antigen engagement (Fig. EV2A). Collectively, these results establish the E-A functional index as a specific, reversible, and reusable metric for T cell effector function triggered by membrane antigens.

### E-A functional screening enables sensitive identification of nanobodies against specific cell-surface tumor antigens

**Figure 2:**
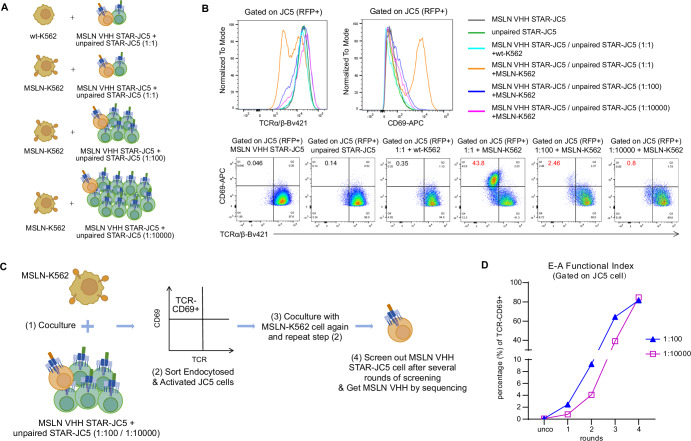
Sensitivity assessment and proof-of-concept screening using the E-A functional index. (**A**) Design of model libraries for sensitivity testing. MSLN VHH STAR-JC5 cells (cognate) were mixed with non-cognate STAR-JC5 cells at defined ratios of 1:1, 1:100, 1:10,000, and co-cultured with either wt-K562 cells or target MSLN-K562 cells. (**B**) Flow cytometry analysis of TCR^−^CD69^+^ JC5 cells in the model libraries after 24 h co-culture. Panel **B** is adapted from Fig. EV2D. (**C**) Schematic of the model screening workflow. The 1:100 and 1:10,000 model libraries underwent four rounds of E-A functional index-based selection followed by FACS sorting and sequencing. (**D**) Enrichment monitoring during model screening. Percentage of E-A functional index-positive (TCR^−^CD69^+^) JC5 cells in both model libraries across four screening rounds through flow cytometry analysis. Data in (**B**) are representative of three independent experiments. Source data are available online for this figure.

To evaluate the sensitivity of the E-A functional index, we co-incubated MSLN VHH STAR-JC5 cells with cognate MSLN-K562 cells at effector-to-target (E:T) ratios ranging from 1000:1 to 1:10. We found that the functional response scaled proportionally with the density of specific antigens, with as few as 2% of cognate target cells capable of triggering over 50% endocytosis and activation of JC5 cells (Fig. EV2B,C). We next assessed whether the E-A functional index could identify a cognate antibody from a background of non-cognate binders. To this end, we constructed three proof-of-concept MSLN VHH STAR-JC5 libraries spiked with non-cognate STAR-JC5 cells at ratios of 1:1, 1:100, and 1:10,000 (Fig. 2A). These mixtures were co-cultured with either wt-K562 cells or MSLN-K562 target cells, and the E-A functional index was measured (Fig. 2B). As expected, the percentage of TCR^−^CD69^+^ JC5 cells was ~50% in the 1:1 group, while it decreased to 2.46% in the 1:100 group and 0.8% in the 1:10,000 group when co-cultured with MSLN-K562 cells.

Based on these findings, we developed an antibody screening strategy termed E-A functional screening. We first applied this method to isolate MSLN VHH STAR-JC5 cells from the 1:100 and 1:10,000 libraries using fluorescence-activated cell sorting (FACS), followed by sequencing of the enriched antibody sequences (Fig. 2C). After four rounds of E-A functional screening, the TCR^−^CD69^+^ JC5 cells were highly enriched to over 80% in both groups and were sorted for Sanger sequencing (Figs. 2D and EV2D). Sequencing analysis revealed specific enrichment of the expected MSLN VHH antibody, with minimal background sequences detected (sequencing results are presented in Figure Source Data File—Fig. 2D). These results demonstrate that the E-A functional index can efficiently isolate cognate antibodies from STAR-JC5 libraries even at a dilution of 1:10,000.

**Figure 3:**
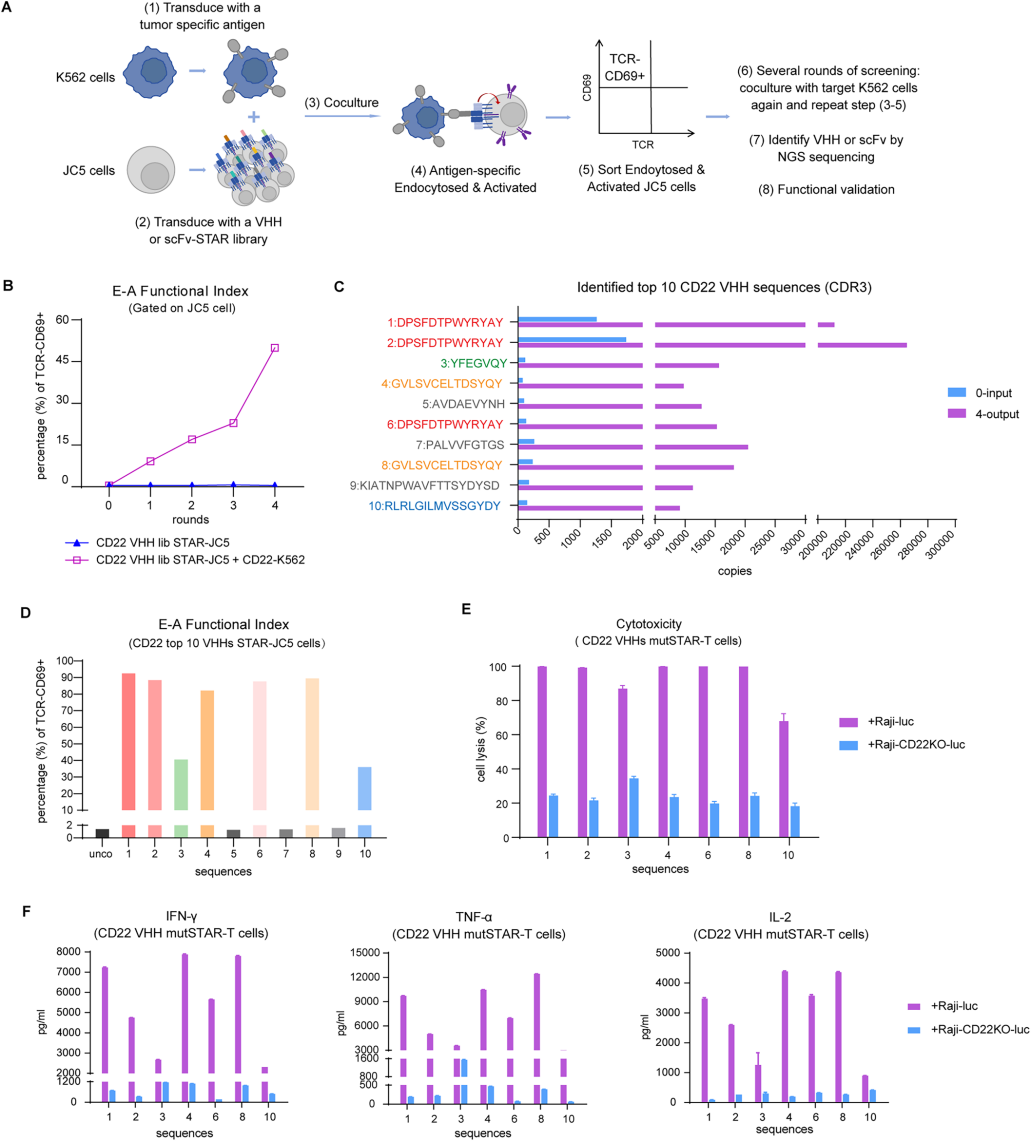
E-A functional screening identifies tumor antigen CD22-specific VHHs. (**A**) Schematic diagram of the E-A functional screening approach for tumor-specific antigen (TSA)-specific antibodies screening. (**B**) Flow cytometry analysis of E-A functional index (TCR^–^CD69^+^) in gated CD22 VHH lib STAR-JC5 cells after each of four rounds of E-A functional screening. (**C**) Identification of enriched CD22 VHHs by next-generation sequencing (CDR3 regions amino acid sequences of VHHs are shown). “0-input” represents the initial CD22 VHH lib. “4-output” represents the fourth-round screening product. The top ten candidates are ranked by the ratio of 4-output/0-input. (**D**) Flow cytometry analysis of E-A functional index in the top ten candidate VHHs-based STAR-JC5 cells co-cultured with target CD22-K562 cells. (**E**) Cytotoxicity (presented as percentage of specific cell lysis) of seven E-A functional index-positive CD22 VHHs-based STAR-T cells against either Raji cells expressing luciferase (Raji-luc) or CD22 knockout Raji-luc cells (Raji-CD22KO-luc). (**F**) Concentrations of IFN-γ, TNF-α, and IL-2 were measured by ELISA in co-culture supernatants from the experiments in (**E**). All co-culture experiments were performed at an E:T ratio of 1:1 for 24 h. Data in (**D**–**F**) are representative of three independent experiments. Data in (**E**, **F**) are presented as mean ± SEM. Source data are available online for this figure.

**Figure EV3:**
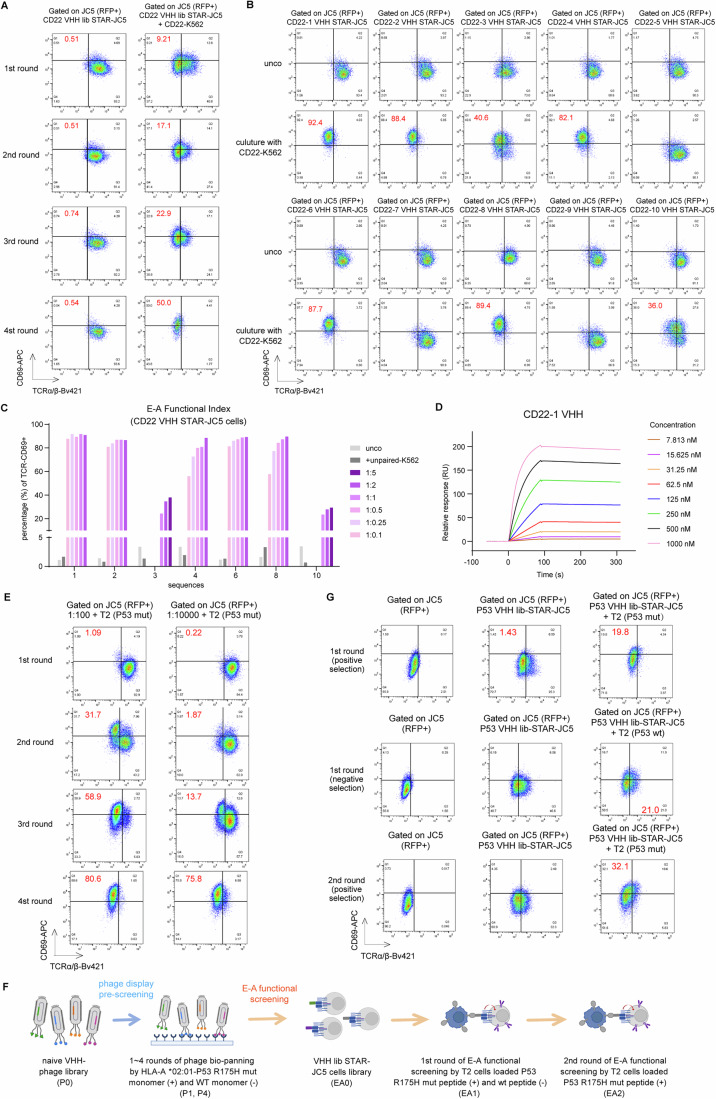
Supporting characterization of the E-A functional screening process for CD22 and P53^R175H^ VHHs. (**A**) FACS analysis of RFP^+^ JC5 cells from the CD22 VHH lib STAR-JC5 cell library after each round of functional screening before and after co-culture with target CD22-K562 cells (E:T = 1:1). (**B**) FACS analysis of the top ten enriched CD22 VHHs expressed in STAR-JC5 cells before and after co-culture with target CD22-K562 cells (E:T = 1:1). (**C**) Flow cytometry analysis of seven E-A functional index-positive CD22 VHHs-based STAR-JC5 cells before and after co-culture with target CD22-K562 cells at different E:T ratios or with unpaired K562 cells as a negative control (The results of CD22-3 and CD22-10 groups at E:T ratios of 1:0.5, 1:0.25, and 1:0.1 are not involved in this experiment). (**D**) Surface plasmon resonance (SPR) binding analysis of CD22-1 VHH (human IgG1-Fc dimer) binding to human CD22. Sensorgrams show responses from multi-cycle kinetics at increasing analyte concentrations. All co-culture experiments were performed for 24 h. Data are representative of three independent experiments. (**E**) FACS analysis of RFP^+^ JC5 cells from 1:100 or 1:10,000 model libraries after each round of functional screening against T2 cells loaded with 10 µM P53 R175H mutant (mut) peptide. (**F**) Screening scheme for the P53 VHH lib STAR-JC5 library. “+“ indicates positive selection using P53 R175H mut peptide; “–“ indicates negative selection using P53 wild-type (wt) peptide. Labels in parentheses (P0, P1, P4, EA0, EA1, and EA2) denote samples subjected to NGS analysis, where “P” indicates phage library samples and “EA” indicates E-A functional screening cell library samples, with numbers indicating the initial library (0) or screening round. (**G**) FACS analysis of gated P53 VHH lib STAR-JC5 cells after each round of functional screening. Round 1 used T2 cells loaded with 1 µM P53 R175H peptide for positive selection and 1 µM P53 wt peptide for negative selection; round 2 used T2 cells loaded with 1 µM P53 R175H peptide for positive selection.

To further validate the sensitivity and specificity of E-A functional screening, we constructed a VHH library by immunizing alpacas with CD22 protein and cloned the VHHs into the STAR format. The resulting library had an estimated diversity of >10^6^. This VHH STAR library was transduced into JC5 cells and co-cultured with K562 cells expressing the target antigen CD22 (Fig. 3A). Four rounds of E-A functional screening were performed until the E-A functional index reached 50% in the co-culture group (Figs. 3B and EV3A). The TCR^−^CD69^+^ JC5 cells from the fourth round were sorted by FACS and subjected to next-generation sequencing (NGS) as the output, with the original pooled library serving as the input control (Fig. 3C). NGS analysis revealed that five of the top ten enriched VHHs shared identical CDR3 sequences: three contained “DPSFDTPWYRYAY” (top 1, 2, and 6), and two contained “GVLSVCELTDSYQY” (top 4 and 8). We selected the top ten enriched VHHs, reconstituted them into STAR-JC5 cells, and re-evaluated their E-A functional index upon co-culture with CD22-K562 cells. Seven of the ten VHHs (excluding CD22-5, 7, and 9) induced substantial endocytosis and activation, with the five VHHs containing the shared CDR3 regions showing particularly strong responses (Figs. 3D and EV3B,C). To confirm functional efficacy, we engineered primary human T cells with mutSTARs carrying each of the seven selected CD22 VHHs. These T cells were then co-cultured with two target tumor cell lines expressing luciferase (luc): human B lymphoblastoid Raji cells, which endogenously express CD22 (Raji-luc), or CD22-knockout Raji cells (Raji-CD22KO-luc) as a negative control. Consistently, all seven CD22 VHH mutSTAR-T cells mediated robust CD22-specific tumor killing and secreted elevated levels of cytokines (IFN-γ, TNF-α, IL-2) in vitro (Fig. 3E,F). The binding characteristics of the lead candidate were further determined. CD22-1 VHH exhibited a therapeutically relevant affinity (*K*_d_ = 3.55 nM) for human CD22 protein in surface plasmon resonance (SPR) assays, thus providing a molecular basis for its robust functional efficacy (Fig. EV3D).

**Figure 4:**
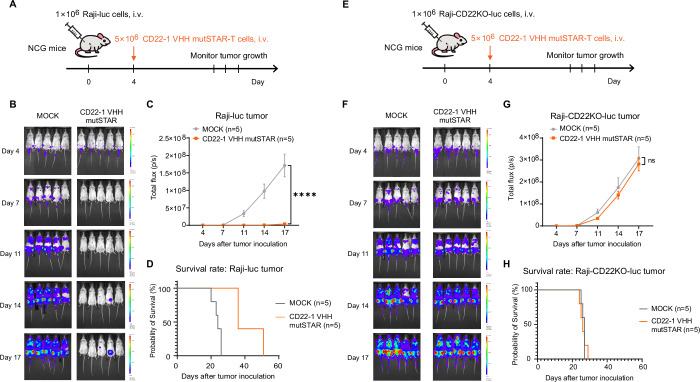
In vivo anti-tumor efficacy of anti-CD22 VHH mutSTAR-T cells. (**A**) Schematic of the experimental process for the Raji-luc xenograft mouse model. NCG mice were intravenously inoculated with 1 × 10^6^ CD22^+^ Raji-luc cells at day 0 and received 5 × 10^6^ CD22-1 VHH mutSTAR-T cells or control RFP^+^ T cells at day 4. (**B**) Representative bioluminescence images of tumor burden in Raji-luc xenografted mice. (**C**) Quantification of tumor progression by bioluminescence intensity in CD22^+^ Raji-luc model. Comparison of tumor fluorescence between control and treatment groups on day 17 showed ****P < 0.0001. (**D**) Survival curves of mice bearing CD22^+^ Raji-luc tumors. (**E**) Schematic of the experimental process for Raji-CD22KO-luc xenograft mouse model. (**F**) Representative bioluminescence images of tumor burden in Raji-CD22KO-luc xenografted mice. (**G**) Quantification of tumor progression by bioluminescence intensity in the Raji-CD22KO-luc model. Comparison of tumor fluorescence between control and treatment groups on day 17 showed *P* = 0.9972. (**H**) Survival curves of mice bearing Raji-CD22KO-luc tumors. Data were representative of three independent experiments (*n* = 5 mice per group). Tumor progression data in (**C**, **G**) are presented as mean ± SEM at the final time point. Statistical analysis was performed using two-way analysis of variance (ANOVA). ****P <0.0001; ns no significant difference (På 0.05). Source data are available online for this figure.

We next evaluated the in vivo anti-tumor activity of CD22-1 VHH mutSTAR-T cells. NOD-Prkdc^em26Cd52^Il2rg^em26Cd22^/NjuCrl (NCG) mice were intravenously injected with Raji-luc cells (Fig. 4A). Following in vivo bioluminescence imaging on day 4, mice were systematically stratified by tumor burden and infused with either CD22-1 VHH mutSTAR-T cells or control RFP-expressing T cells (MOCK-T cells), ensuring equivalent baseline tumor load across treatment groups. The tumor burden was monitored every few days. We found that treatment with CD22-1 VHH mutSTAR-T cells significantly suppressed tumor growth and extended survival (P < 0.0001) (Fig. 4B–D). To assess specificity and off-target toxicity, we repeated the experiment using Raji-CD22KO-luc cells. CD22-1 VHH mutSTAR-T cells showed no effect on CD22-knockout tumors, confirming antigen-specific targeting and minimal off-target activity in vivo (Fig. 4E–H). In summary, E-A functional screening provides a robust and efficient platform for identifying functional antibodies against tumor membrane antigens, with high sensitivity, specificity, and direct linkage to therapeutic efficacy.

### E-A functional screening efficiently identifies multiple P53^R175H^ neoantigen-specific TCR-like nanobodies

**Figure 5:**
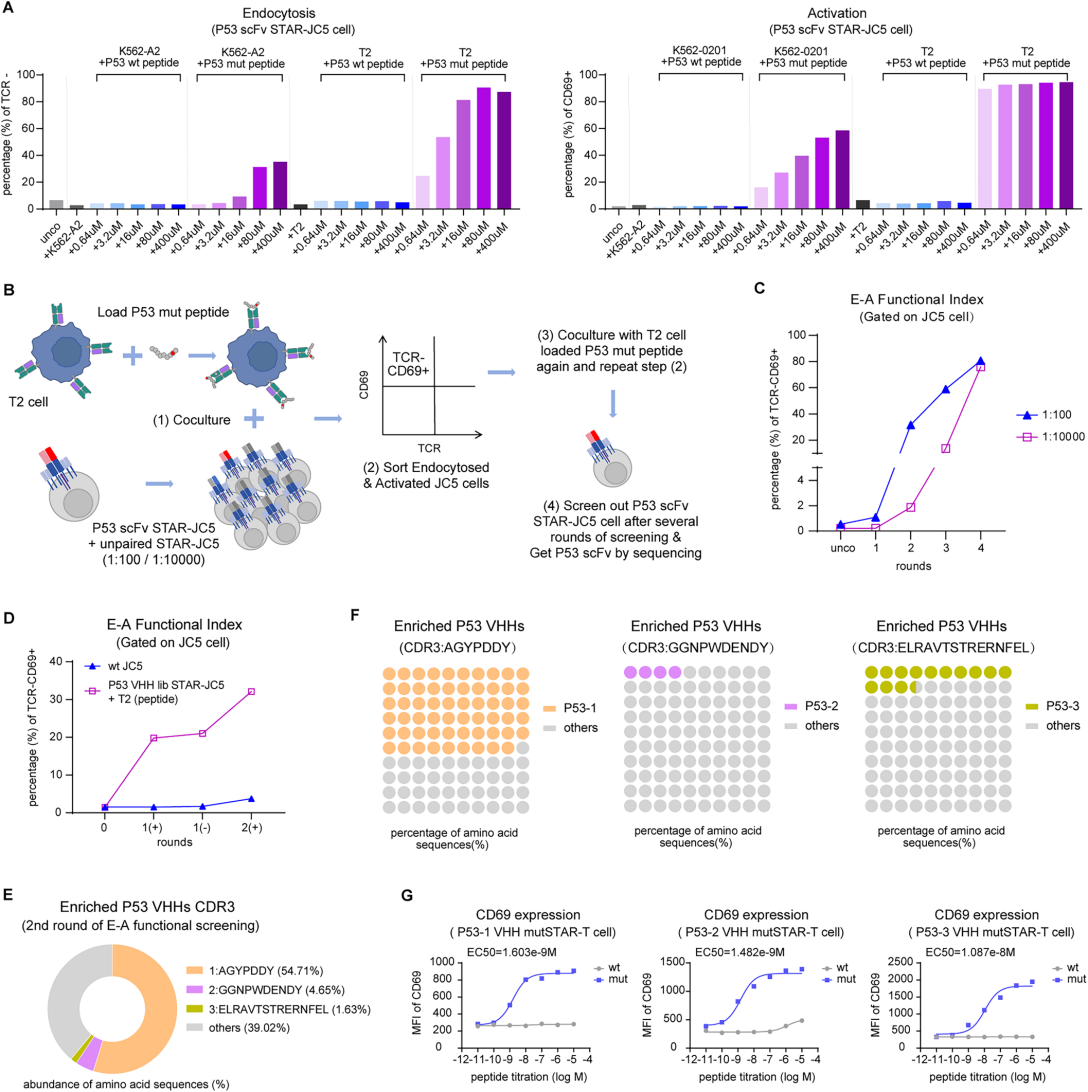
The neoantigen-specific E-A functional index enables the isolation of VHHs against the tumor P53^R175H^ neoantigen. (**A**) Flow cytometry analysis of STAR endocytosis (TCRα/β negative) and CD69 activation in P53 scFv STAR-JC5 cells co-cultured with target K562 cells expressing HLA-A*02:01 (K562-A2) or T2 cells loaded with indicated concentrations of P53 wt peptide (HMTEVVRRC) or P53 mut peptide (R175H: HMTEVVRHC). (**B**) Schematic of the E-A functional screening using model libraries containing P53 scFv STAR-JC5 cells spiked into non-cognate STAR-JC5 cells at 1:100 and 1:10,000 ratios. (**C**) Flow cytometry analysis of E-A functional index in the model libraries from (**B**) after each round of screening against T2 cells loaded with 10 µM P53 R175H mut peptides. (**D**) Flow cytometry analysis of E-A functional index in gated P53 VHH lib STAR-JC5 cells before and after three rounds of functional screening. Rounds 1 used T2 cells with 1 µM P53 R175H mut peptide as positive selection (+) and P53 wt peptide as negative selection (−); round 2 used positive selection (+) with 1 µM P53 R175H mut peptide. (**E**) NGS analysis of VHH CDR3 enrichment in the P53 VHH library after the second round of screening. CDR3 sequences and abundance are shown for the three dominant CDR3 clusters (CDR3-1, -2, -3) identified in the enriched library. (**F**) Relative abundance of three dominant candidate VHHs within their respective CDR3 clusters from (**E**). (**G**) Flow cytometry analysis of CD69 activation in three dominant candidate VHHs-based mutSTAR-T cells co-cultured with T2 cells loaded with different doses of P53 wt peptide or R175H mut peptide. MFI, mean fluorescence intensity. EC50, half maximal effective concentration. Data in (**A**, **G**) are representative of three independent experiments. Source data are available online for this figure.

The surface of tumor cells presents a diverse array of antigenic targets for immune recognition. These include not only tumor-associated membrane antigens but also tumor-specific neoantigens arising from genomic alterations, such as CDK4^R24C^, KRAS^G12V/C/D^, and others, which are presented on the cell surface by HLA molecules (Peri et al, 2023). Having established the feasibility of E-A functional screening for identifying membrane antigen-specific antibodies, we next sought to extend this strategy to the discovery of neoantigen-specific antibodies. We selected the most frequent missense mutant of the tumor suppressor gene *TP53* (P53^R175H^, presented by HLA-A*02:01) as a target neoantigen. A previously reported specific scFv (H2) (Hsiue et al, 2021) was constructed into STAR-JC5 cells to serve as a positive control in co-culture experiments. Meanwhile, we pulsed K562 cells expressing HLA-A*02:01 (K562-A2) and TAP-deficient T2 cells (which express HLA-A*02:01) with P53 R175H mutant (mut) peptide (HMTEVVRHC) or P53 wild-type (wt) peptide (HMTEVVRRC) at varying concentrations as target cells. After co-incubation, we found that only target cells pulsed with the mut peptide triggered endocytosis and activation in P53 scFv STAR-JC5 cells, with the response increasing in a peptide concentration-dependent manner. This phenomenon was not observed in wt peptide-pulsed groups, confirming the strict antigen specificity of the E-A functional index (Fig. 5A). Moreover, T2 cells elicited a stronger stimulatory response than K562 cells, likely because their TAP deficiency facilitates the presentation of purer exogenous peptides, making them a more suitable antigen-presenting cell line for this assay. These results suggest that the E-A functional index is neoantigen-specific, and its magnitude depends on antigen density.

To determine the sensitivity of the E-A functional index in isolating neoantigen-specific antibodies, we repeated the dilution experiments using P53 scFv STAR-JC5 cells spiked into non-cognate STAR-JC5 cells at ratios of 1:100 and 1:10,000. These mixtures were co-cultured with T2 cells loaded with the cognate P53 R175H mut peptide (Fig. 5B). After four rounds of functional screening, the E-A functional index-positive JC5 cells were substantially enriched to ~80% in both dilution groups (Figs. 5C and EV3E). Sanger sequencing confirmed the specific recovery of the expected P53 scFv antibody sequence (Figs. 5C and EV3E, sequencing results are presented in Figure Source Data File—Fig. 5C). These results demonstrate that the E-A functional index can efficiently isolate cognate neoantigen-specific antibodies from STAR-JC5 libraries even at a dilution of 1:10,000.

Although scFv and monoclonal antibodies (mAbs) targeting the neoantigen P53^R175H^ have been reported (Chai et al, 2024; Hsiue et al, 2021; Hwang et al, 2018), specific VHHs against this high-value target remain unexplored. The lack of such VHHs precludes their application in modular therapeutic formats where their small size and stability offer distinct pharmacological benefits. These properties make VHHs particularly valuable for accessing intracellular epitopes (Bao et al, 2021; Liu et al, 2021a). We therefore sought to apply our E-A functional screening platform to the direct identification of specific VHHs targeting the neoantigen P53^R175H^ presented by HLA-A*02:01.

We constructed a naïve alpaca VHH library and initially employed conventional phage display, an established screening technique, for preliminary selection (Fig. EV3F; Table EV1). Following bio-panning against both the HLA-A*02:01-P53 R175H mut monomer and the WT monomer, the library diversity (Chao1 predicted) was reduced from 9.65 × 10^8^ to 2.41 × 10^8^, and the number of observed unique amino acid sequences (S_obs) decreased from 2.71 × 10^8^ to 4.24 × 10^7^. Notably, however, the four rounds of phage selection did not yield substantial enrichment of specific binders, as reflected by the lack of substantial enrichment in the Top100 sequence coverage (Top100_cov% <6%), underscoring the limitations of affinity-based screening in isolating functional neoantigen-specific VHHs.

We then proceeded to convert the pre-screened VHH library into STAR-JC5 cells, establishing a VHH lib STAR-JC5 cells library for E-A functional screening, which leverages T cell activation as a functional readout, thereby circumventing the limitations of affinity-based methods. Strikingly, the conversion of the phage library into the STAR-JC5 format dramatically reshaped the library landscape (Tables EV2). This process alone substantially reduced the library complexity, with the Chao1 index plummeting by ~99.6% to 1.02 × 10^6^ and S_obs number falling by ~99.3% to 3.14 × 10^5^. Most importantly, it led to a remarkable enrichment, as evidenced by the Top100 sequence coverage soaring to 59.37%—representing an increase of over tenfold compared to the pre-conversion library. We attribute this effective library enrichment primarily to the stringent selection imposed by eukaryotic expression, which filters out VHH clones incapable of proper folding, post-translational modification, or membrane localization in mammalian cells, thereby efficiently enriching for therapeutically compatible candidates.

**Figure EV4:**
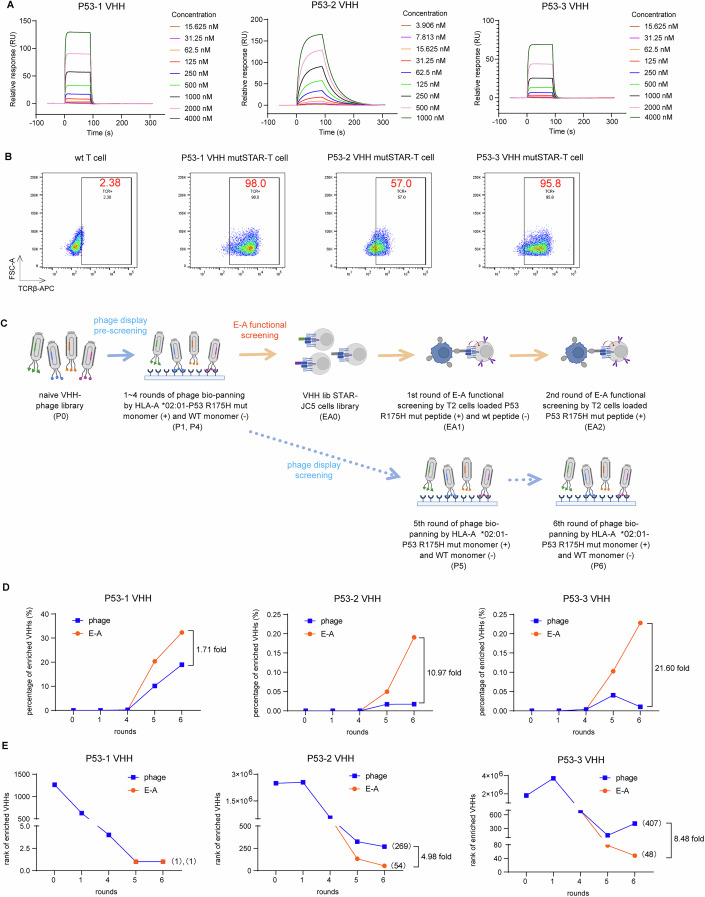
Characterization of P53^R175H^-specific VHHs and comparison of screening strategies (E-A index versus phage display). (**A**) SPR binding analysis of three P53 VHHs (human IgG1-Fc dimers) to immobilized P53 R175H-HLA-A*02:01 monomer. Sensorgrams show binding responses from multi-cycle kinetics at increasing analyte concentrations. Data are representative of three independent experiments. (**B**) Flow cytometry analysis of surface expression of different P53 VHH mutSTAR constructs on primary human T cells. Cells were gated by FSC for single cells, and mutSTAR expression was detected using anti-mouse TCRβ-APC staining. (**C**) Schematic of the parallel screening strategy. Following four pre-screening rounds of phage display, two additional rounds of phage screening (P5, P6) were performed concurrently with two rounds of E-A functional screening of the converted cell library (EA1 and EA2). “+“ indicates positive selection using P53 R175H mut peptide; “–“ indicates negative selection using P53 wt peptide. Labels in parentheses (P0, P1, P4, P5, P6, EA0, EA1, and EA2) denote samples subjected to NGS analysis, where “P” indicates phage library samples and “EA” indicates E-A functional screening cell library samples, with numbers indicating the initial library (0) or screening round. (**D**) The relative amino acid sequence abundance (%) of functional antibodies P53-1, -2, and -3 at key stages of the screening process. (**E**) The rank positions of functional antibodies P53-1, -2, and -3 within the library across selection rounds. X-axis labels in (b, c): “0” indicates the initial naïve VHH library; “1” and “4” indicate phage pre-screening rounds 1 and 4; “5” indicates E-A cell library screening round 1 (EA1) and phage screening round 5 (P5); “6” indicates E-A cell library screening round 2 (EA2) and phage screening round 6 (P6). Fold-enrichment values compare the final output ratios (EA2 vs P6) between the two methods.

We subsequently performed two rounds of E-A functional screening. The first round employed T2 cells loaded with the P53 R175H mut peptide for positive selection, alongside T2 cells loaded with the P53 wt peptide for negative selection to eliminate clones with nonspecific binding or self-activating properties. Upon completion of the first round, the population of E-A functional index-positive cells reached 21%. A second round of positive selection further increased this rate to 32.1% (Figs. 5D and EV3G). Deep sequencing of the post-screening output revealed a focused library: the Chao1 index decreased by 50.6% to 5.04 × 10^5^, the S_obs number fell by 43.3% to 1.78 × 10^5^, while the Top100 sequence coverage rose substantially to 72.56%, indicating a highly enriched state. Given the critical role of CDR3 in antigen-antibody recognition, we performed a comprehensive analysis of the CDR3 region sequences of antibodies within the library and ranked them based on their relative abundance (Fig. 5E). We found that after two rounds of E-A functional screening, three dominant CDR3 sequences were significantly enriched (1, 2, and 3). Clones containing these CDR3s collectively constituted over 60% of all amino acid sequences in the second-round library. From the sequence clusters harboring these CDR3s, we selected three dominant candidate clones (P53-1, P53-2, and P53-3) for further validation (Fig. 5F). Surface plasmon resonance (SPR) analysis revealed that these VHHs bound to the P53 R175H-HLA-A*02:01 monomer with dissociation constant (*K*_d_) of 2.85 μM, 375 nM, 5.25 μM, respectively, with *K*_d_ values in the low to moderate micromolar range, typical of initial-stage nanobody candidates isolated from naïve libraries (Fig. EV4A). We subsequently constructed these VHHs into the mutSTAR format and confirmed their successful expression on primary human T cells (Fig. EV4B). We next evaluated the functional specificity of these three VHH mutSTAR-T cells by stimulating them with serially diluted peptides (Fig. 5G). All three candidates exhibited dose-dependent activation specifically in response to the P53 mut peptide. Notably, P53-2, which exhibited the highest binding affinity in SPR analysis, also demonstrated the highest functional sensitivity, with an EC_50_ of 1.482 nM, compared to 1.603 nM for P53-1 and 10.87 nM for P53-3. A minor degree of nonspecific activation for this high-sensitivity candidate was observed only at non-physiologically high peptide concentrations (>1 µM), a level substantially exceeding physiological nanomolar-range HLA-peptide complexes. In contrast, P53-1 and P53-3 VHH mutSTAR-T cells maintained strict specificity across all tested concentrations, showing no detectable activation to the wt peptide.

### STAR-T cells engineered with E-A screened nanobodies exhibit both potent anti-tumor efficacy and low cross-reactivity

**Figure 6:**
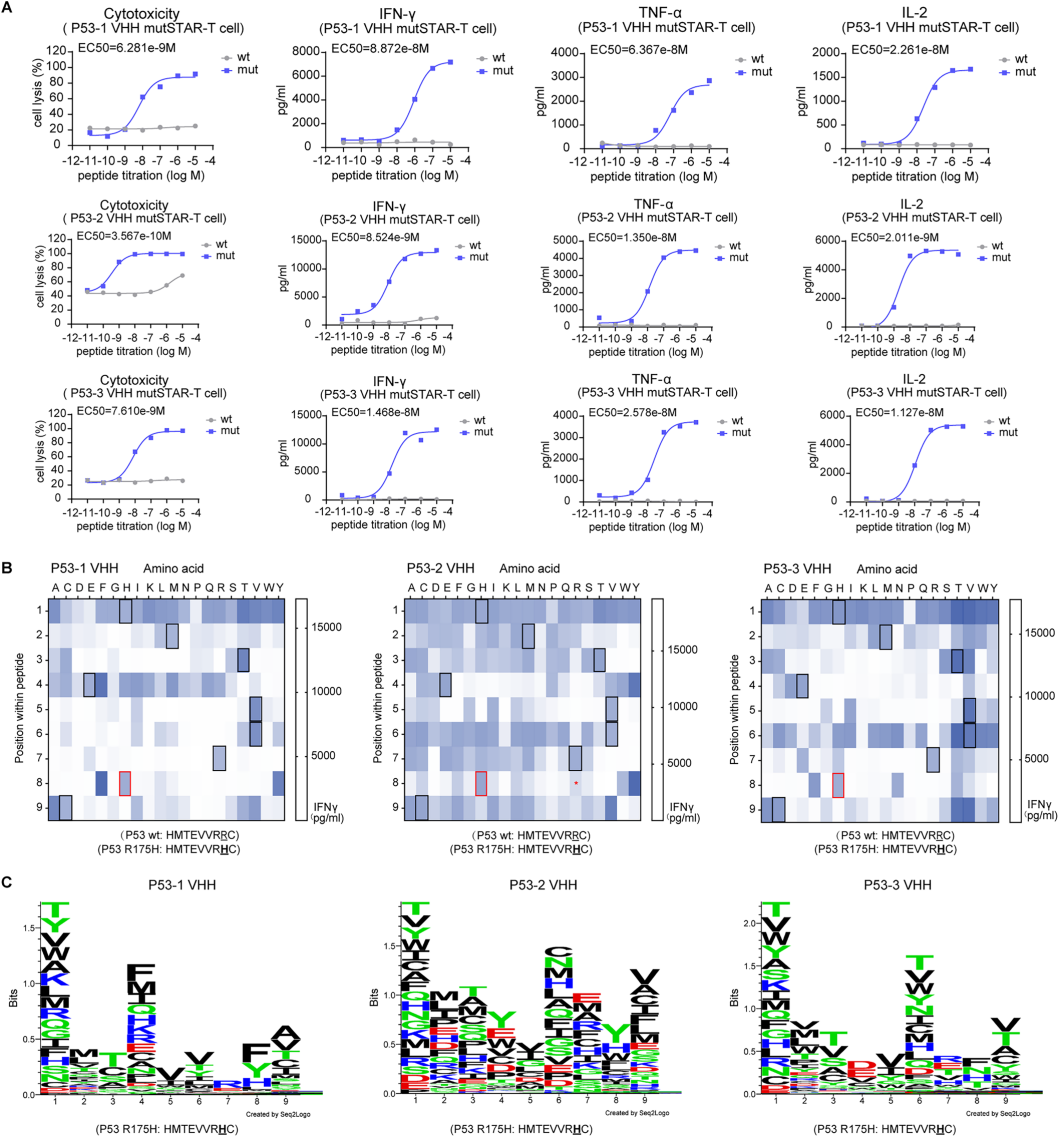
In vitro functional characterization and cross-reactivity profiling of P53^R175H^-specific VHHs. (**A**) Cytotoxicity (percentage of specific cell lysis) and cytokine secretion (IFN-γ, TNF-α, and IL-2) by the three E-A functional index-positive candidate VHHs-based mutSTAR-T cells in response to T2 cells loaded with titrated P53 wt peptide or R175H mut peptide. (**B**) Peptide scanning mutagenesis analysis of P53 VHHs cross-reactivity. Each position in the P53 R175H peptide (HMTEVVR**H**C) was substituted with the other 19 amino acids. T2 cells pulsed with individual variant peptides (10 μM) were co-cultured with P53-1, -2, -3 VHH mutSTAR-T cells, and IFN-γ release was measured (mean of three technical replicates, heatmap). Black boxes: parental peptide residues; red box: mutation site (H^175^); red asterisk: wild-type residue (R^175^). (**C**) Seq2Logo representation of P53 VHHs binding specificity. Logo graphs were generated from the IFN-γ response data (divided by 10⁵) using the PSSM-Logo algorithm. All co-culture were performed for 24 h at an E:T ratio of 1:1. Data in (**A**) are representative of three independent experiments. EC50, half maximal effective concentration. Source data are available online for this figure.

Having established the antigen specificity and activation dynamics of the selected candidates, we next evaluated their therapeutic potential in anti-tumor functional assays. To this end, we assessed the cytotoxicity and cytokine release (IFN-γ, TNF-α and IL-2) of the three P53 VHHs-based mutSTAR-T cells (Fig. 6A). In a bioluminescence-based cytotoxicity assay, all three candidates mediated substantial lysis of T2 cells loaded with the P53 mut peptides. Consistent with the activation profiling, P53-2 VHH mutSTAR-T cells exhibited minimal nonspecific killing against wt peptide-pulsed targets, which was only detectable at high peptide concentrations. Accordingly, all three mutSTAR-T cell groups secreted significant levels of cytokines upon stimulation with the mut peptide. Taken together, these functional data confirm that the E-A functional screening platform successfully identified multiple VHHs capable of specifically recognizing the neoantigen P53^R175H^ in the context of HLA-A*02:01, and that the resulting STAR-T cells can mount a potent and specific anti-tumor response in vitro.

A critical challenge in developing immunotherapeutic antibodies is mitigating off-target toxicity. To systematically evaluate the potential cross-reactivity of these three functional P53 VHHs, we applied a scanning mutagenesis approach to profile their specificity across a spectrum of peptide variants. We constructed a comprehensive peptide library by systematically substituting each amino acid in the P53 R175H peptide with the other 19 common amino acids. The resulting 171 variant peptides were individually loaded onto T2 cells, which were then co-cultured with the respective P53 VHH mutSTAR-T cells, and T cell activation was quantified by IFN-γ release (Fig. 6B). The results revealed that P53-1 and P53-3 VHHs exhibited superior specificity compared to P53-2, particularly towards the C-terminal half of the peptide at which the mutant residue (H^175^) is located. Most amino acid substitutions within this critical region abrogated recognition. In contrast, the N-terminal portion of the peptide displayed greater tolerance to substitutions. This distinct specificity profile was visually summarized in a Seq2Logo graph (Fig. 6C).

**Figure 7:**
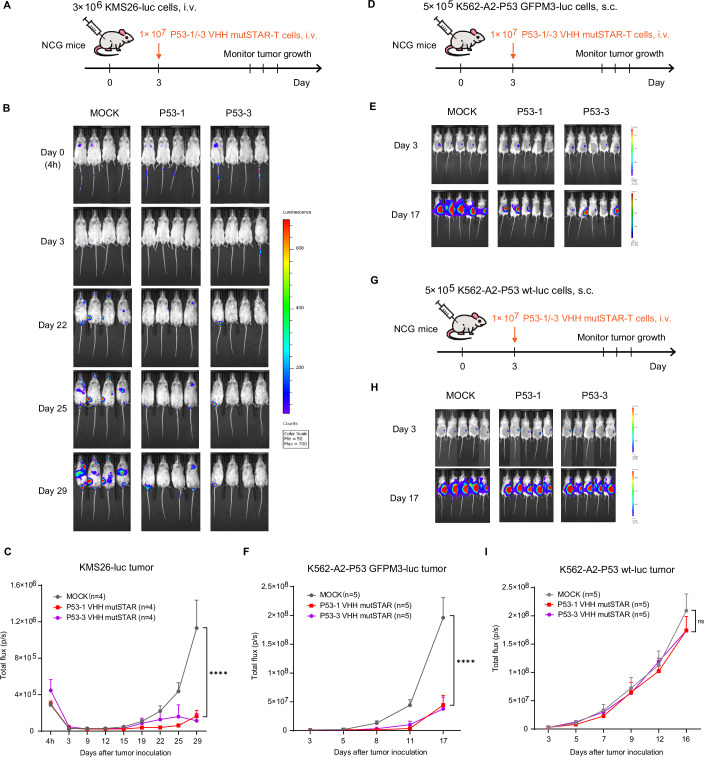
In vivo anti-tumor efficacy of anti-P53^R175H^ VHHs mutSTAR-T cells. (**A**) Schematic of the experimental process for the KMS26 mouse model. NCG mice were intravenously inoculated with 3 × 10^6^ KMS26-luc cells (endogenously expressing P53^R175H^ neoantigen and HLA-A*02:01) at day 0, and 1 × 10^7^ P53-1 or P53-3 VHH mutSTAR-T cells were intravenously injected at day 3. (**B**) Representative bioluminescence images of KMS26-luc tumor burden. Mice received either P53-1 VHH mutSTAR-T cells, P53-3 VHH mutSTAR-T cells, or control RFP-expressing T cells (Mock; *n* = 4 per group). (**C**) Quantification of KMS26-luc tumor progression by bioluminescence intensity. Tumor fluorescence on day 29: MOCK vs. P53-1 VHH mutSTAR group, ****P < 0.0001; MOCK vs. P53-3 VHH mutSTAR group, ****P < 0.0001; P53-1 VHH mutSTAR group vs. P53-3 VHH mutSTAR group, *P* = 0.9249 (ns). (**D**,** G**) Schematic of the experimental process for the K562 mouse model. NCG mice were subcutaneously inoculated with 5 × 10^5^ K562-A2-P53-GFPM3-luc cells stably expressing P53^R175H^ neoantigen (**D**) or K562-A2-P53 wt-luc cells stably expressing P53 wt protein (**G**) at day 0, and 1 × 10^7^ P53-1 or P53-3 VHH mutSTAR-T cells were intravenously injected at day 3. (**E**,** H**) Representative bioluminescence images of K562-luc tumors (*n* = 5 per group). (**F**) Quantification of K562-A2-P53-GFPM3-luc tumor progression by bioluminescence intensity. Tumor fluorescence on day 17: MOCK vs. P53-1 VHH mutSTAR group, ****P < 0.0001; MOCK vs. P53-3 VHH mutSTAR group, ****P < 0.0001; P53-1 VHH mutSTAR group vs. P53-3 VHH mutSTAR group, På 0.9999 (ns). (**I**) Quantification of K562-A2-P53-wt-luc tumor progression by bioluminescence intensity. Tumor fluorescence on day 16: MOCK vs. P53-1 VHH mutSTAR group, *P* = 0.5602 (ns); MOCK vs. P53-3 VHH mutSTAR group, *P* = 0.5620 (ns); P53-1 VHH mutSTAR group vs. P53-3 VHH mutSTAR group, På 0.9999 (ns). Data were representative of three independent experiments and are presented as mean ± SEM. Statistical analysis of quantification in (**C**,** F**, **I**) was performed using two-way analysis of variance (ANOVA). ****P < 0.0001; ns no significant difference (På 0.05). Source data are available online for this figure.

The in vitro analyses established P53-1 and P53-3 VHHs as highly enriched candidates with superior specificity, prompting an evaluation of their anti-tumor efficacy in vivo. We first intravenously engrafted NCG mice with KMS26 multiple myeloma cells, which endogenously express both the P53^R175H^ neoantigen and HLA-A*02:01, establishing a disseminated hematologic malignancy model (Fig. 7A). Following in vivo bioluminescence imaging, mice were systematically stratified by tumor burden at four hours after tumor inoculation, and infused with either P53-1 VHH mutSTAR-T cells, P53-3 VHH mutSTAR-T cells, or control RFP-expressing T cells (Mock-T cells) at day 3. The tumor burden was monitored every few days. We found that both P53-1 and P53-3 VHH mutSTAR-T cells mediated significant suppression of tumor growth (*P* < 0.0001) (Fig. 7B,C). To further validate the anti-tumor efficacy and on-target, off-tumor toxicity, we engineered K562 mouse models stably expressing either the P53^R175H^ tandem minigenes (K562-A2-GFPM3-luc) or the P53 wt protein (K562-A2-P53 wt-luc), and subsequently performed STAR-T cell therapies (Fig. 7D–I). In mice bearing subcutaneous tumors expressing the P53^R175H^ neoantigen, both P53-1 and P53-3 VHH mutSTAR-T cells consistently mediated potent and specific tumor suppression (*P* < 0.0001). Notably, neither showed any significant off-target activity on P53 wt control tumors, confirming the high specificity of these candidates and demonstrating no detectable on-target, off-tumor toxicity in vivo. Collectively, these in vivo findings demonstrate that the P53-1 and P53-3 VHH-based STAR-T cells mediate potent and antigen-specific anti-tumor activity. This conclusively validates that the E-A functional screening platform is a powerful tool for discovering functional neoantigen-specific antibodies with direct therapeutic potential.

### E-A screened nanobodies can be adapted into multiple therapeutic modalities

**Figure 8:**
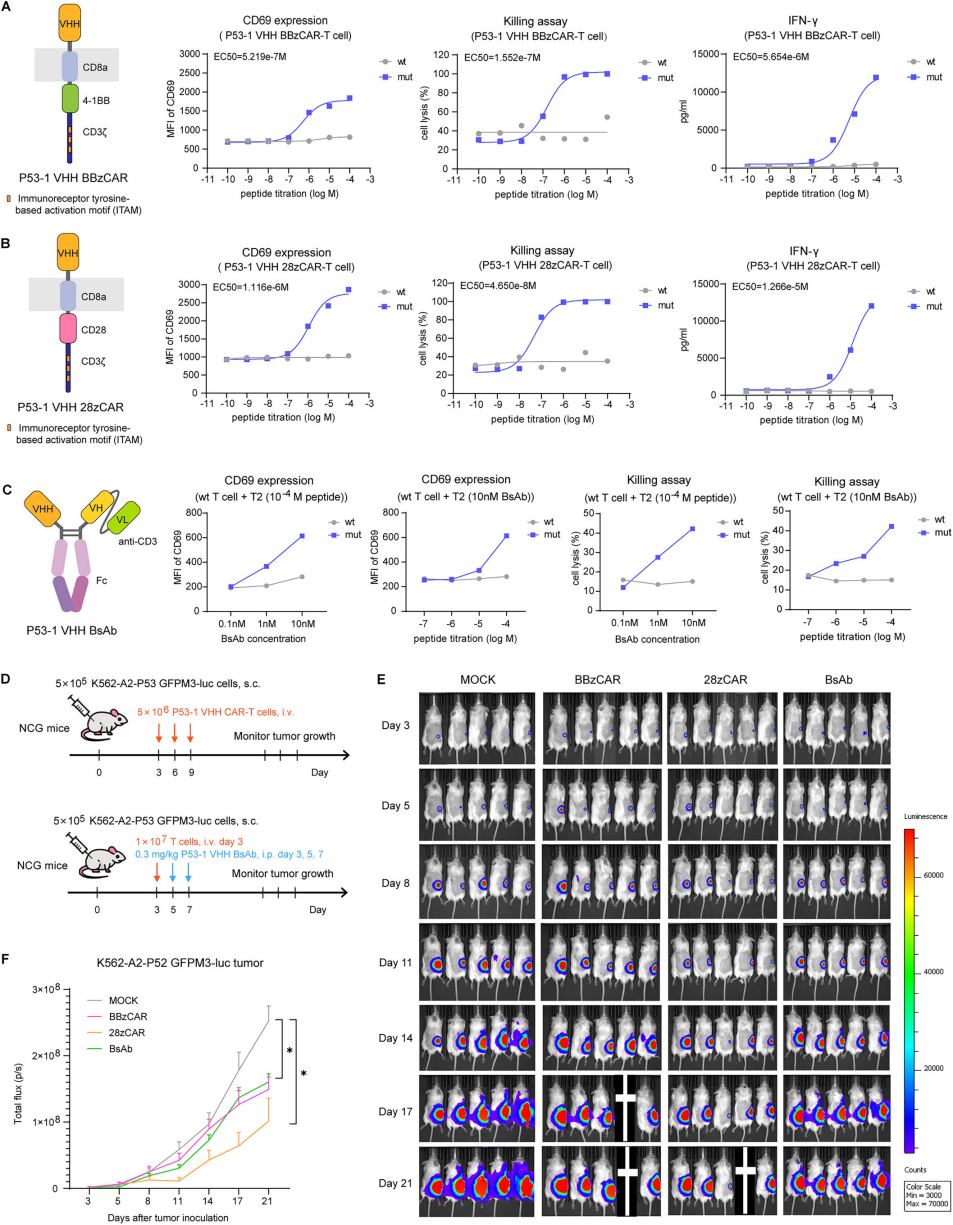
CAR-T cells and a bispecific antibody with P53-1 VHH enable potent anti-tumor efficacy. (**A**) Schematic structure and functional characterization of P53-1 VHH in BBzCAR format. CD69 activation, cytotoxicity, and IFN-γ secretion were assessed against T2 cells loaded with titrated P53 wt or R175H mut peptides. (**B**) Schematic structure and functional characterization of P53-1 VHH in 28zCAR format. (**C**) Schematic structure and functional characterization of P53-1 VHH in BsAb format. (**D**) Experimental schematic for comparative in vivo evaluation. NCG mice bearing subcutaneous K562-A2-p53-GFPM3-luc tumors (5 × 10⁵ cells) received either: P53-1 VHH BBzCAR-T cells (5 × 10⁶ cells intravenously, days 3, 6, 9), P53-1 VHH 28zCAR-T cells (same regimen), or human primary T cells (1 × 10⁷ intravenously, day 3) followed by P53-1 VHH BsAb (0.3 mg/kg intraperitoneally, days 3, 5, 7). (**E**) Representative bioluminescence images of tumor burden across treatment groups (*n* = 5 mice per group). (**F**) Quantification of tumor progression by bioluminescence intensity. Tumor fluorescence on day 21: MOCK vs. BBzCAR group, *P = 0.0385; MOCK vs. 28zCAR group, *P = 0.0464; MOCK vs. BsAb group, *P = 0.0427; BBzCAR vs. 28zCAR group, *P* = 0.6457 (ns); BBzCAR vs. BsAb group, *P* = 0.9565 (ns); 28zCAR vs. BsAb group, *P* = 0.4708 (ns). All in vitro co-cultures (**A**–**C**) were performed for 20 h at an E:T ratio of 1:1. Data were representative of three independent experiments and are presented as mean ± SEM. Statistical analysis of quantification in (**F**) was performed using two-way analysis of variance (ANOVA). *P < 0.05. EC50, half maximal effective concentration. Source data are available online for this figure.

To evaluate the therapeutic versatility of the antibodies screened by E-A screening from STAR-T cell libraries, we engineered the P53-1 VHH into both CAR and bispecific T cell engager (BsAb) formats. We first constructed P53-1 VHH CAR with either 4-1BB (BBzCAR) or CD28 (28zCAR) costimulatory domains as previously described (Liu et al, 2021b), and transduced them into primary human T cells (Fig. 8A,B). Upon co-culturing with T2 cells loaded with P53 R175H mut peptide or wt peptide, both CAR-T cell variants exhibited similar antigen-specific activation, potent cytotoxicity, and significant IFN-γ release. We also produced a P53-1 VHH BsAb comprising an anti-CD3 scFv and Fc domain, designed to bridge T cells via CD3 and tumor cells via the P53 R175H-HLA-A*02:01 complex (Fig. 8C). To determine the functional thresholds, we titrated both the antigen density and the BsAb concentration. The results demonstrated that T cells, in the presence of 10 nM BsAb, mediated substantial activation and cytotoxicity against target cells pulsed with 10^−4^ M P53 R175H mut peptide. Reducing the BsAb concentration revealed a dose-dependent response, with 1 nM sufficient to trigger distinct T cell effector functions.

We further assessed the in vivo efficacy of these therapeutic modalities. NCG mice bearing subcutaneous K562-A2-P53-GFPM3-luc tumors were stratified and treated. For CAR groups, the P53-1 VHH BBzCAR-T cells or P53-1 VHH 28zCAR-T cells were infused intravenously at days 3, 6, and 9. For the BsAb group, mice were infused with 1 × 10^7^ in vitro expanded human primary T cells via lateral tail vein injection on day 3, and subsequently received intraperitoneal injection of P53-1 VHH BsAb at day 3, 5, and 7 (Fig. 8D). Control mice received non-transduced T cells. We found that both CAR-T and BsAb treatments resulted in significant tumor suppression (*P* < 0.05), demonstrating that the P53-1 VHH candidate confers potent anti-tumor activity across multiple therapeutic platforms (Fig. 8E,F). Most importantly, the potent in vivo efficacy of P53-1 VHH delivered via these distinct therapeutic modalities underscores the robustness and direct translational utility of antibodies discovered through our E-A functional screening platform for enabling cancer immunotherapy.

## Discussion

Over the past few decades, antibody-based cancer therapies have witnessed remarkable advancements, driving a significant surge in the demand for high-affinity and highly specific antibodies (Carter and Rajpal, 2022). Previously reported antibody discovery methods—most notably phage display and hybridoma technologies—have enabled the identification of antibodies to tumor-associated antigens (TAAs), but they largely rely on affinity-based screening and require substantial post-selection functional validation. Phage display can also be hampered by nonspecific binding to reagents or plastics and by propagation of such binders across iterative rounds (t Hoen et al, 2012).

In this study, we introduce an Endocytosis-Activation (E-A) functional screening method that leverages a STAR-T cell library to identify antibodies directly in a human T cell line, thereby prioritizing candidates with the desired cellular activity, including both recognition of antigens and TCR signal transduction. Our side-by-side comparison (Fig. EV4C–E; Tables EV1–4) demonstrates that E-A functional screening effectively complements affinity-based phage display, with superior enrichment kinetics and efficiency. Consistent with these observations, conventional phage panning exhibited limited enrichment beyond five rounds, a phenomenon potentially attributable to the overgrowth of nonspecific clones with growth advantages. In contrast, E-A screening effectively enriched rare binders that would otherwise require additional rounds of selection, underscoring its utility in accessing the full repertoire of functional clones. Direct analysis reveals that E-A screening not only enriched the dominant P53^R175H^/HLA-A*02:01-reactive clone more rapidly and potently, but also identified putative functional, low-abundance clones—which exhibited comparable functional output to the dominant clone—that were neglected by phage display alone. These results underscore the unique advantage of E-A screening in prioritizing functional candidates. Moreover, converting the library to the STAR-T cell format served as a critical pre-enrichment step that markedly reduced diversity by eliminating clones defective for expression, folding, or membrane trafficking in mammalian cells, thus substantially enhancing the library’s functional quality for E-A screening.

**Figure EV5:**
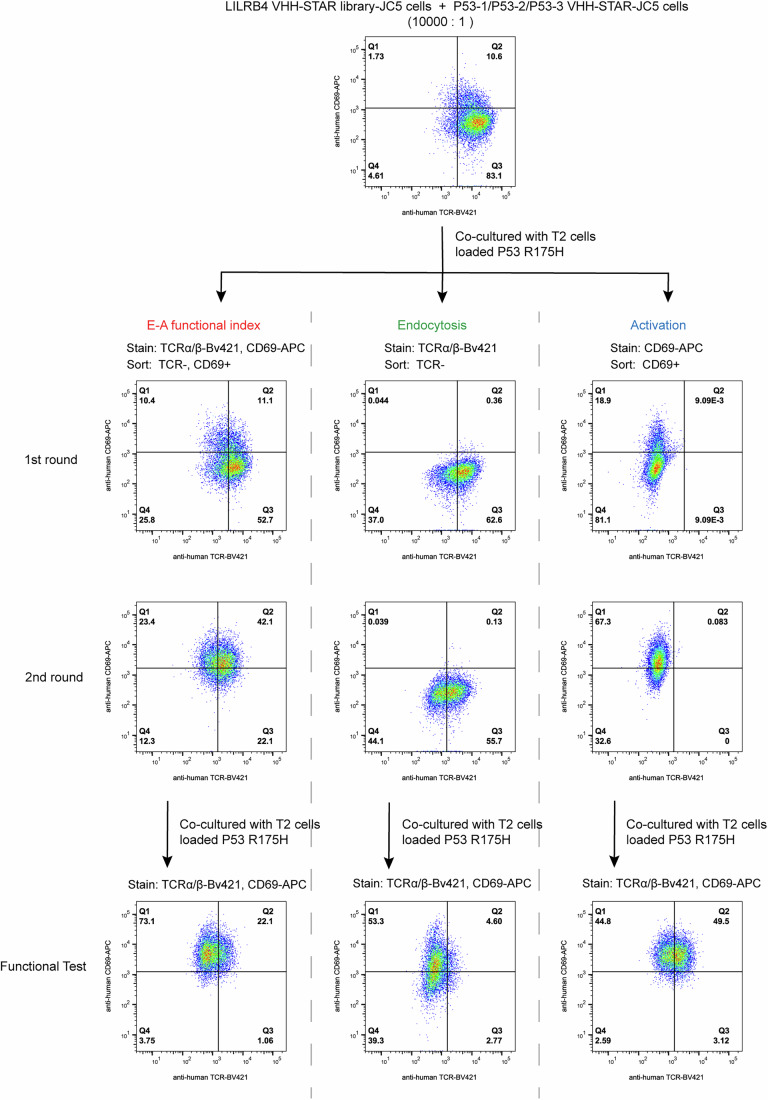
Screening workflow for evaluating different selection strategies. A LILRB4-specific VHH STAR-JC5 cell library, generated from immunized alpaca and spiked with known functional clones (P53-1, -2, -3) at a 1:10,000 dilution, was co-cultured with P53 R175H mut peptide-loaded T2 cells and subjected to two rounds of selection using either the dual-parameter E-A functional index (TCR^−^CD69^+^), TCR^-^ only, or CD69^+^ only strategies. Enriched populations were analyzed by flow cytometry for TCR endocytosis and CD69 expression after stimulation.

A defining advantage of the E-A functional screening method is its dual-functional readout, which not only significantly reduces nonspecific antibody enrichment compared to other cell-based methods (Bloemberg et al, 2020; Carrara et al, 2022; Doerner et al, 2014; Ma et al, 2021; Zhang et al, 2014), but also operates with better efficiency and precision than single-readout systems(Alonso-Camino et al, 2013) (Fig. EV5). This two-parameter gating of STAR receptor endocytosis and T-cell activation (CD69 upregulation) effectively mitigates common confounders in CAR-T cell library panning (Liu et al, 2021b), such as antigen-independent tonic signaling. By directly quantifying functional activity, our approach reduces the need for extensive downstream validation, thereby streamlining the antibody discovery process. Notably, the incorporation of rigorous positive and negative selection steps within the E-A screening framework remains essential to exclude nonspecific or non-functional binders.

In this study, using E-A functional screening, we successfully identified not only antibodies targeting cell-surface antigens but also multiple TCR-like antibodies capable of recognizing HLA-presented neoantigens on tumor cells. These neoantigen targets offer distinct advantages due to their unique tumor specificity and absence in normal tissues, which can largely circumvent on-target/off-tumor toxicity and resistance issues, making them ideal targets for personalized tumor therapy (Schumacher and Schreiber, 2015; Zhang et al, 2021). Among these, the P53 mutation-specific STARs are of particular interest given their recognition of a shared driver mutation hotspot, suggesting potential for broader therapeutic application. Functional characterization confirmed their ability to mediate antigen-specific T cell activation and tumor cell-specific cytotoxicity. Building on these findings, further studies will focus on preclinical evaluation to support potential clinical translation.

TCR-like antibodies are a novel class of antibodies that can recognize peptide-MHC complexes on tumor cells by mimicking TCR specificity, showing considerable promise in cancer immunotherapy targeting such tumor neoantigens (He et al, 2019). Conventionally, such antibodies are screened using phage or yeast surface display combined with synthetic or immunized libraries, which are primarily affinity-based and require additional functional verification (Douglass et al, 2021). In contrast, our E-A functional screening strategy enables more rapid and efficient identification of specific TCR-like antibodies against HLA-presented neoantigens, as demonstrated by the successful isolation of TCR-like nanobodies targeting the public neoantigen P53^R175H^. Notably, these nanobodies offer several advantages over scFv-format antibodies: their smaller size facilitates engineering and enhances tumor penetration, while also conferring potentially lower immunogenicity. Moreover, the E-A-screened TCR-like nanobodies can be readily reformatted into diverse therapeutic modalities—including STARs, CARs, and bispecific antibodies—providing versatile avenues for personalized cancer immunotherapy targeting tumor-specific neoantigens.

In summary, the E-A functional screening platform using STAR-T cell libraries provides a cell-based strategy for prioritizing antibody candidates with on-target cellular activity against both TAAs and HLA-presented neoantigens. This approach, which integrates endocytosis and early activation signaling of STAR-T cells, addresses the affinity-function discordance in conventional discovery by directly capturing functional clones. While further investigation is required to optimize the E-A-screened antibodies for clinical translation, this platform holds considerable value for adoptive T-cell therapy by enabling highly specific and efficient identification of TCR-like antibodies against tumor-specific neoantigens, thereby enhancing the safety and efficacy of cancer immunotherapy.

## Methods

**Table:** Reagents and tools table

Reagent/resource	Reference or source	Identifier or catalog number
**Experimental models**		
Lenti-X 293T cells	Takara Biomedical Technology Co., Ltd.	Cat# Z2180N
KMS26 cells	ATCC	Cat# CRL-3344
K562 cells	ATCC	Cat# CCL-243
Raji cells	ATCC	Cat# CCL-86
Jurkat E6.1 cells	ATCC	Cat# TIB-152
T2 cells	BriStar Immunotech Co., Ltd.	N/A
JC5 cells (TCRα/β knockout Jurkat)	This study	N/A
Primary human PBMCs	Shanghai Oribiotech Biotechnology Co., Ltd.	N/A
NCG mice (NOD-Prkdcem26Cd52Il2rgem26Cd22/NjuCrl)	GemPharmatech Co.	Cat# T001475
**Recombinant DNA**		
pHAGE lentiviral vector (EF1α promoter)	This study/Laboratory stock	N/A
pHAGE lentiviral vector (hEF1α-HTLV promoter)	This study/Laboratory stock	N/A
psPAX2 (packaging plasmid)	Addgene	Cat# 12260
pMD2.G (envelope plasmid)	Addgene	Cat# 12259
pMDLg/pRRE (packaging plasmid)	Addgene	Cat# 12251
pRSV-Rev (packaging plasmid)	Addgene	Cat# 12253
MSLN VHH STAR construct	This study (PCT/CN2022/130106)	N/A
CD123 VHH STAR construct	This study (US20160251440)	N/A
CD19 scFv STAR construct (FMC63)	This study (Nicholson et al, 1997)	N/A
GPC3 scFv STAR construct (GC33)	This study (Nakano et al, 2010)	N/A
CD3 scFv construct (OKT3)	This study (Van Wauwe et al, 1980)	N/A
CD22 VHH library in STAR pHAGE	This study	N/A
Naïve VHH library in pADL-22c	This study	N/A
P53^R175H^-HLA-A*02:01-specific VHHs	This study	N/A
Luciferase-encoding pHAGE plasmid	This study	N/A
P53-GFPM3 tandem minigenes construct	This study	N/A
HLA-A*02:01-encoding pHAGE plasmid	This study	N/A
**Antibodies**		
Anti-human TCRα/β-BV421	BioLegend	Cat# 306722
Anti-human CD69-APC	BioLegend	Cat# 310910
Anti-mouse TCRβ-APC	BioLegend	Cat# 109212
Anti-human CD69-APC/Cy7	BioLegend	Cat# 310914
HLA-A2-APC	BioLegend	Cat# 343307
Fixable Viability Dye eFluor 506	eBioscience	Cat# 65-0866-18
Anti-human CD3 (for T cell activation)	BioLegend	Cat# 317326
Anti-human CD28 (for T cell activation)	BD Biosciences	Cat# 555725
Human fibronectin	BD Biosciences	Cat# 354008
CD22 VHHs (functional candidates)	This study	N/A
P53^R175H^-specific VHHs (P53-1, -2, -3)	This study	N/A
**Oligonucleotides and other sequence-based reagents**		
TRAC gRNA sequence: GTCTCTCAGCTGGTACACGGC	This study	N/A
TRBC gRNA sequence: GGGCTCAAACACAGCGACCTC	This study	N/A
CD22KO gRNA: GGTATCCGATCCAATTGCAG	This study	N/A
VHH-Forward primer: GATGTGCAGCTGCAGGAGTC	This study	N/A
VHH-Reverse primer: TGAGGAGACGGTGACCTGGGT	This study	N/A
P53 wt peptide: HMTEVVRRC	GenScript	Custom synthesis
P53 R175H mut peptide: HMTEVVRHC	GenScript	Custom synthesis
P53 peptide scanning mutagenesis library (171 variants)	GenScript	Custom synthesis
**Chemicals, enzymes and other reagents**		
DMEM	Gibco	Cat# C11995500BT
RPMI 1640	Gibco	Cat# C11875500BT
RPMI 1640 (for primary T cells)	Invitrogen	Cat# 11875135
Fetal bovine serum (FBS)	ExCell Bio	Cat# 12A056
Heat-inactivated FBS (for primary T cells)	Gibco	Cat# 10091148
Penicillin-Streptomycin	Yeasen	Cat# 60162ES76
Recombinant human IL-2	Peprotech	Cat# 200-02
Polyethyleneimine (PEI)	Polysciences Inc.	Cat# 24765-1
Polybrene	Yeasen	Cat# 40804ES86
PEG8000	Sigma-Aldrich	Cat# 89510
D-Luciferin, sodium salt	Yeasen	Cat# 40902ES02
RIPA lysis buffer	Beyotime	Cat# P0013B
Luciferase reagent	Yeasen	Cat# 11401ES80
**Software**		
FlowJo	BD Biosciences	https://www.flowjo.com
GraphPad Prism 9	GraphPad Software	https://www.graphpad.com
Living Image Software	PerkinElmer	https://www.perkinelmer.com
seqkit	Open source	https://bioinf.shenwei.me/seqkit/
**Other**		
B Cell Isolation Kit	Stemcell	Cat# 17954_C
FastPure Cell/Tissue Total RNA Isolation Kit	Vazyme	Cat# RC112-01
TIANamp Genomic DNA Kit	Tiangen	Cat# DP304-02
TIANgel Midi Purification Kit	Tiangen	Cat# DP219-03
Seamless cloning kit	Clone Smarter	Cat# C5891-50
Protein A affinity chromatography resin	N/A	N/A
50 kDa ultrafiltration disks	N/A	N/A
Human IFN-γ ELISA Kit	Invitrogen	Cat# 88-7316-88
Human TNF-α ELISA Kit	eBioscience	Cat# 88-7346-88
Human IL-2 ELISA Kit	Invitrogen	Cat# 88-7025-88

### Cell lines and primary cells

Lenti-X 293T cells were purchased from Takara Biomedical Technology Co., Ltd. KMS26, K562, Raji and Jurkat E6.1 cells were purchased from American Type Culture Collection (ATCC). JC5 cells were derived from Jurkat E6.1 cells by knocking out TCRα and TCRβ chains with a CRISPR-Cas9 system [guide RNA (gRNA) sequences: TRBC_ GGGCTCAAACACAGCGACCTC and TRAC_GTCTCTCAGCTGGTACACGGC]. T2 cells were obtained from BriStar Immunotech Co., Ltd. Primary human peripheral blood mononuclear cells (PBMCs) were purchased from Shanghai Oribiotech Biotechnology Co., Ltd. All cell lines were authenticated by short tandem repeat (STR) profiling and tested negative for mycoplasma contamination using a PCR-based method. Lenti-X 293T cells were cultured in DMEM (Gibco, cat. C11995500BT) supplemented with 10% (v/v) FBS (ExCell Bio, cat. 12A056) and 100 IU/ml penicillin-streptomycin (Yeasen, cat. 60162ES76). KMS26, K562, Raji, Jurkat E6.1, JC5 and T2 cells were cultured in RPMI 1640 (Gibco, cat. C11875500BT) supplemented with 10% (v/v) FBS (ExCell Bio, cat. 12A056) and 100 IU/ml penicillin-streptomycin (Yeasen, cat. 60162ES76). Primary human PBMCs were cultured in RPMI 1640 (Invitrogen, cat. 11875135) supplemented with 10% (v/v) heat-inactivated FBS (Gibco, cat. 10091148), 100 IU/ml penicillin-streptomycin (Yeasen, cat. 60162ES76) and 200 IU/mL rhIL-2 (Peprotech, cat. 200-02). All cells were cultured at 37 °C with 5% atmospheric CO_2_.

### Vector construction

Lentiviral vector (pHAGE) with a plasmid backbone containing an EF1α promoter and an IRES-linker GFP reporter was used for transducing the K562 target cell line. A fusion protein gene (P53-GFPM3) of GFP full-length gene and P53 R175H (HMTEVVR**H**C) tandem minigenes (3×TACAAGCAGTCACAGCACATGACGGAGGTTGTGAGGCACTGCCCCCACCATGAGCGC, coding protein: 3×YKQSQHMTEVVR**H**CPHHER) was constructed in a pHAGE plasmid for transducing in vivo K562-A2-P53-GFPM3 tumor cell line. A full-length P53 wt gene was constructed in a pHAGE plasmid for transducing the in vivo K562-A2-P53 wt tumor cell line. Luciferase-encoding genes were constructed in pHAGE plasmids carrying the puromycin resistance gene as an effective selection marker or a GFP reporter gene. Another pHAGE vector with a plasmid backbone containing an hEF1α-HTLV promoter and an IRES-linker RFP reporter was used for transducing T cell lines and primary T cells. The plasmid backbone structure for STAR, mutSTAR, BBzCAR and 28zCAR has been previously described (Liu et al, 2021b). In brief, STAR was designed as an antibody-TCR chimera by ligating human TCR constant regions with variable regions of an antibody, and mutSTAR was a mutant of STAR by replacing human TCR constant regions with murine counterparts and introducing the following modifications: an additional interchain disulfide bond within TCRα/β constant regions, hydrophobic substitutions to TCRα transmembrane domain, and cytoplasmic domains from CD3ζ (UniProtKB-P20963, amino acids 52 to 164), 4-1BB (UniProtKB-Q07011, amino acids 214 to 255) and CD28 (UniProtKB-P10747, amino acids 180 to 220). Sequences for VHHs and scFvs used in this study were as previously described: MSLN VHH (PCT/CN2022/130106), CD123 VHH (US20160251440), CD19 scFv (FMC63) (Nicholson et al, 1997), GPC3 scFv (GC33) (Nakano et al, 2010), CD3 scFv (OKT3) (Van Wauwe et al, 1980). Gene fragments were assembled by seamless cloning kit (Clone Smarter, #C5891-50) into vector backbones together with a Kozak sequence for optimal translation.

### Lentivirus production

Lentiviruses encoding MSLN, CD123, CD19, GPC3, CD22, P53-GFPM3, P53 wt, HLA-A*02:01, and luciferase were packaged using packing and envelope plasmids psPAX2 and pMD2.G in Lenti-X 293T cells. Lentiviruses encoding VHH STARs, VHH STAR libraries, VHH mutSTARs and VHH CARs were packaged using packing and envelope plasmids pMD2.G, pMDLg/pRRE and pRSV-Rev in Lenti-X 293 T cells. Plasmids were transfected into cells using polyethyleneimine (Polysciences Inc., #24765-1). The virus-containing culture medium supernatants were collected at 48 and 72 h after transfection and filtered through a 0.45-μm syringe filter. The viruses were concentrated by mixing with PEG8000 (Sigma-Aldrich) overnight at 4 °C and centrifuged for 30 min at 3500 rpm.

### Cell lines construction

KMS26, K562, Raji and T2 cells were engineered and used as target cells. JC5 cells and primary human T cells were engineered and used as effector cells. KMS26, K562, Raji, T2 and JC5 cells were spin-infected with concentrated viral supernatants supplemented with 10 μg/ml Polybrene (Yeasen, cat. 40804ES86) for 90 min at 1500 rpm at 32 °C and replaced with fresh RPMI 1640 medium 12 h later. On day 3 post-infection, the reporter-positive target cells were sorted by FACS (fluorescence-activated cell sorting) or puromycin-resistant cells were selected to establish derivative cell lines as indicated. K562 cells stably expressing HLA-A*02:01 (K562-A2 cells) were prepared through transduction with lentiviruses of HLA-A*02:01. Raji-CD22KO cells were derived from Raji cells by knocking out CD22 with a CRISPR-Cas9 system [gRNA sequence: GGTATCCGATCCAATTGCAG]. All target cell lines were stably expressing luciferase through transduction with lentiviruses encoding luciferase.

### T cell activation and transduction

To transduce primary human T cells, PBMCs (1 × 10^6^ cells/ml) were activated in 48-well plates coated with 5 μg/ml anti-human CD3 (Biolegend, cat. 317326), 1 μg/ml anti-human CD28 (BD Biosciences, cat. 555725) and 5 μg/ml human fibronectin (BD Biosciences, cat. 354008) in T cell medium (PBMCs medium). After 36 h of activation, the cells were spin-infected with concentrated viral supernatants for 90 min at 1700 rpm at 32 °C, and replaced with fresh T cell medium 24 h later. The transduction efficiency was determined using flow cytometry 72 h post-transduction.

### Library generation

All animal experiments were approved by the Committee on the Ethics of Animal Experiments of the Institute of Microbiology, Chinese Academy of Science (IMCAS) and conducted in compliance with the recommendations in the Guide for the Care and Use of Laboratory Animals of IMCAS Ethics Committee.

The CD22 immune VHH library was generated via alpaca immunization, a service outsourced to BriStar Immunotech Co., Ltd. The CD22 VHH STAR-T cell library and all the library screening were performed in-house. In brief, a healthy alpaca was immunized subcutaneously in the neck area with 200 μg of CD22 protein mixed with MnJ adjuvant every 2 weeks. A week after the sixth immunization, peripheral blood of the alpaca was collected and B cells were sorted using B Cell Isolation Kit (Stemcell, cat. 17954_C). RNA of the sorted B cells was extracted using FastPure Cell/Tissue Total RNA Isolation Kit (Vazyme, cat. RC112-01) and reverse transcribed into cDNA, which was used as template in PCR to obtain VHH library with the following primers: VHH-Forward, 5’-GATGTGCAGCTGCAGGAGTC-3’; VHH-Reverse, 5’-TGAGGAGACGGTGACCTGGGT-3’. The VHH library was then constructed into the STAR pHAGE plasmid under the control of hEF1α-HTLV promoter and GM-CSF signal peptide (before TCRBC chain). Then the CD22 VHH STAR library was transduced to JC5 cells by lentiviral infection to generate the CD22 VHH lib STAR-JC5 cell library.

The naïve VHH library was generated by BriStar Immunotech Co., Ltd. through peripheral blood collection from healthy (never antigen-challenged) alpacas (outsourced service). The subsequent VHH STAR-T cell library and all the library screening were performed in-house. In brief, peripheral blood of healthy alpaca was collected and total B cells were isolated using B Cell Isolation Kit (Stemcell, cat. 17954_C). RNA of the sorted B cells was extracted using FastPure Cell/Tissue Total RNA Isolation Kit (Vazyme, cat. RC112-01) and reverse transcribed into cDNA, which served as a template for PCR amplification of the VHH library with VHH-Forward and VHH-Reverse primers. The VHH library was then cloned into the pADL-22c phagemid vector to generate a naïve alpaca VHHs-phage library. The library capacity and diversity of the phage libraries——including the initial phage library and the output libraries from the 1st, 4th, 5th, and 6th rounds of phage bio-panning—were assessed by next-generation sequencing (NGS) on Illumina PE150 platform. After four rounds of phage bio-panning, the pre-screened VHH library was cloned into the STAR pHAGE plasmid under the control of hEF1α-HTLV promoter and GM-CSF signal peptide (before TCRBC chain). The capacity and diversity of the cell libraries—including the initial cell library and the output libraries after the first and second rounds of E-A functional screening—were assessed by NGS on the Illumina PE250 platform. The total sequencing read counts for each round screening are listed in Tables EV1 and 2. Raw sequencing data were processed and analyzed using custom code and available tools, including bbmerge and seqkit for sequence merge, amino acid translation and statistics.

### Peptide loading

The peptides in this study were all synthesized by Nanjing GenScript Biotech Corporation at a purity of >98%. Peptides were dissolved in DMSO at a 10 mM working stock and stored at −20 °C. Gradient dilutions of the peptides were performed using RPMI 1640 medium. Target cells (5 × 10^5^ cells/ml) were loaded with the specified concentration of peptides for 2 h at 37 °C. After incubation, the cells were washed three times with PBS and resuspended in medium for co-incubation experiments.

### BsAb production

Using the heterodimeric Fc variant technology KiHss-AkKh, as previously described (Wei et al, 2017), we fused the VHH fragment of P53-1 VHH to the Fc-engineered variant (LALAPG), and the anti-CD3 scFv to the hole variant Fc region. The P53-1 VHH bispecific antibody (BsAb) was produced via transient co-transfection of plasmids encoding both arms into FreeStyle 293-F cells. The supernatant containing BsAbs was purified using Protein A affinity chromatography, following the manufacturer’s instructions. The purified proteins were desalted into PBS using 50 kDa ultrafiltration disks and subsequently filtered through a 0.22 µm membrane to remove aggregates and achieve sterility. The heterogeneity and purity of the proteins were then confirmed by SDS-PAGE analysis.

### Flow cytometry

Cells were stained with fluorophore-conjugated antibodies (0.5 μg/ml) in PBS at a concentration of 1 × 10^7^ cells/ml. The following antibodies were used: anti-human TCRα/β-BV421 (BioLegend, cat. 306722), anti-human CD69-APC (BioLegend, cat. 310910), anti-mouse TCRβ-APC (BioLegend, cat. 109212), anti-human CD69-APC/Cy7 (BioLegend, cat. 310914), fixable viability dye eFluor 506 (eBioscience, #65-0866-18) and HLA-A2-APC (BioLegend, cat. 343307). Flow cytometry data were acquired by BD Fortessa and analyzed using FlowJo software (BD Biosciences).

### Cytotoxicity assay

In order to establish target cells for a bioluminescence-based cytotoxicity assay, a panel of cell lines were generated via lentiviral transduction, including Raji-luciferase, Raji-CD22KO-luciferase, KMS26-luciferase, K562-A2-P53-GFPM3-luciferase, K562-A2-P53 wt-luciferase and T2-luciferase. 100,000 target cells in T cell medium were seeded in 96-well plates, co-cultured with effector T cells at 1:1 E: T ratios for 24 h in triplicate. Wells containing only target cells served as negative controls for baseline cytotoxicity. Subsequently, the supernatant was removed, and the collected cells were treated with 100 μL 1 × RIPA lysis buffer (Beyotime, cat. P0013B) on ice for 30 min. After centrifuging for 10 min at 3500 rpm, 50 µL lysate supernatant was mixed with 50 µL Luciferase reagent (Yeasen, cat.11401ES80) in a LumaPlate-96 white plate (Nunc, cat. 463201), followed by measuring BLI with a luminometer (Promega GloMax® 96 Microplate Luminometer). The % specific lysis of tumor cells was calculated using the formula: % specific lysis = (luminescence of target cells − luminescence of target cells co-cultured with effector cells)/luminescence of target cells × 100.

### Enzyme-linked immunosorbent assay

The cytokine responses of T cells were determined by the cytokines released into the culture medium. The cytokine concentrations were measured using the following enzyme-linked immunosorbent assay kits per the manufacturer’s instructions: human IFN-γ (Invitrogen, cat. 88-7316-88), human TNF-α (eBioscience, cat. 88-7346-88) and human IL-2 (Invitrogen, cat. 88-7025-88).

### In vivo anti-tumor activity in a xenograft tumor model

Six- to eight-week-old male NCG mice were purchased from GemPharmatech Co., Ltd (Nanjing, China; Strain NO. T001475: NOD/ShiLtJGpt-*Prkdc*^*em26Cd52*^*Il2rg*^*em26Cd22*^/Gpt, SPF) and kept under specific pathogen-free conditions (with a 12-h light/dark cycle and unrestricted access to food and water) in the animal facility of Tsinghua University. Mice health was monitored daily. All mouse experiments were conducted according to Institutional Animal Care and Use Committee (IACUC)-approved protocols (Approval No.15-LX1). Tumor burden was measured using a sensitive in vivo luminescence imaging (IVIS Spectrum, PerkinElmer) method after intraperitoneal D-Luciferin, Sodium Salt D (Yeasen, cat. 40902ES02) injection, and total flux was computed using the provided software (Living Image, PerkinElmer), which measures the brightness through typical circular regions of interest (ROIs). According to their initial tumor burden, mice were serpentine randomized to mock and treatment groups. T cells were infused intravenously via the tail vein in 100 μl volume. BsAb were injected intraperitoneally at 0.3 mg/kg in 200 μl volume per dose. Tumor progression was evaluated every few days, and the end points were determined by a tumor flux intensity above 5 × 10^8^. Mice were sacrificed with a CO_2_ chamber.

### PCR amplification and deep sequencing

Genomic DNA of sorted VHH STAR-JC5 cells was extracted using TIANamp Genomic DNA Kit (Tiangen, cat. DP304-02) and used as template in PCR amplification in a Bio-Red Thermal Cycler PCR System with the following program: initial denaturation step at 95 °C for 3 min followed by 25 cycles of 95 °C for 15 s, 59 °C for 15 s, and 70 °C for 30 s, then a final extension at 70 °C for 3 min, followed by a 4 °C hold. The primers used in PCR amplification were VHH-Forward (5’-GATGTGCAGCTGCAGGAGTC-3’) and VHH-Reverse (5’-TGAGGAGACGGTGACCTGGGT-3’), previously described, which were focused only on the VHH domain. The amplified PCR products were purified using the TIANgel Midi Purification Kit (Tiangen, cat. DP219-03) according to the manufacturer’s protocol. Sequencing was conducted by GENEWIZ using Illumina NovaSeq sequencing. Amino acid sequences of VHHs identified in this study were listed in Table EV5.

### Statistical analysis

Statistical analyses were performed using GraphPad Prism 9 software. Sample size was determined based on preliminary data or prior experience to ensure adequate statistical power. For in vitro experiments, each condition was tested in at least three independent replicates; for in vivo experiments, each group contained *n* ≥ 4 mice. No inclusion or exclusion criteria were applied. Blinding was not performed during the in vivo experiment because tumor sizes were visibly different between groups; however, all data quantification (e.g., luminescence intensity) was conducted using automated software (IVIS Living Image) with standardized regions of interest, thus avoiding operator-dependent bias. Randomization was performed using a serpentine (tumor burden-based) method to ensure balanced baseline tumor burden across groups. Statistical comparisons between two groups were determined by two-tailed Student’s *t*-tests for unpaired data. Statistical comparisons between multiple groups were determined by two-way analysis of variance (ANOVA). Data were reported as mean ± SEM. *P < 0.05; **P < 0.01; ***P < 0.001; ****P < 0.0001; ns not significant.

## Supplementary information

Table EV1

Table EV2

Table EV3

Table EV4

Table EV5

Peer Review File

Source data Fig. 1

Source data Fig. 2

Source data Fig. 3

Source data Fig. 4

Source data Fig. 5

Source data Fig. 6

Source data Fig. 7

Source data Fig. 8

Expanded View Figures

## Data Availability

The original NGS DNA-seq data have been deposited in the Sequence Read Archive (https://www.ncbi.nlm.nih.gov/sra) under accession numbers PRJNA1172294. The source data of this paper are collected in the following database record: biostudies:S-SCDT-10_1038-S44321-026-00455-z.
