# Supplementary material for: Functional screening of TCR-like antibodies using STAR-T cell library for cancer immunotherapy
Source: EMBO Mol Med. 2026 Jun 8;18(7):2748–76. doi: 10.1038/s44321-026-00455-z (PMC13365543; doi:10.1038/s44321-026-00455-z)
Supplement: Supplementary file 1 — Table EV1 [file 44321_2026_455_MOESM1_ESM.docx]

**Table EV1**

Diversity and sequencing metrics across selection rounds of phage display bio-panning

from P53 VHH screening NGS data

| Sample | row | S_obs | S_obs_ge2 | Chao1 | expH | invSimpson | Top100_cov% | Top100_reads | Total  Reads |
| --- | --- | --- | --- | --- | --- | --- | --- | --- | --- |
| P0 | 2.71E+08 | 2.71E+08 | 6.37E+07 | 9.65E+08 | 2.12E+07 | 1.09E+04 | 5.07 | 3.74E+07 | 7.39E+08 |
| P1 | 7.58E+07 | 7.58E+07 | 1.60E+07 | 4.05E+08 | 6.93E+06 | 1.26E+04 | 4.64 | 8.59E+06 | 1.85E+08 |
| P4 | 4.24E+07 | 4.24E+07 | 7.02E+06 | 2.41E+08 | 3.38E+06 | 1.07E+04 | 5.21 | 1.41E+07 | 2.70E+08 |
| P5 | 2.63E+07 | 2.63E+07 | 7.06E+06 | 7.70E+07 | 1.55E+04 | 7.05E+01 | 44.15 | 9.41E+07 | 2.13E+08 |
| P6 | 1.28E+07 | 1.28E+07 | 2.42E+06 | 6.38E+07 | 5.33E+02 | 1.82E+01 | 68.75 | 1.88E+08 | 2.73E+08 |

Row: Sample identifier representing the initial library and selection rounds. P0: Initial naïve alpaca VHHs-phage library. P1, P4, P5, P6: Phage pools after rounds 1, 4, 5, and 6 of bio-panning, respectively (rounds 2 and 3 were not sequenced).

S_obs: Number of observed unique amino acid sequences.

S_obs_ge2: Number of unique sequences observed at least twice.

Chao1: Chao1 estimator, predicting the lower bound of total sequence richness.

expH: Exponential of Shannon entropy, representing the effective number of sequences.

invSimpson: Inverse Simpson index, a measure of diversity that weights towards dominant sequences.

Top100_cov%: Cumulative relative abundance (%) of the top 100 most abundant unique sequences.

Top100_reads: Total number of sequencing reads mapping to the top 100 most abundant unique sequences.

TotalReads: Total number of high-quality sequencing reads after preprocessing for each sample.
