# Supplementary material for: Functional screening of TCR-like antibodies using STAR-T cell library for cancer immunotherapy
Source: EMBO Mol Med. 2026 Jun 8;18(7):2748–76. doi: 10.1038/s44321-026-00455-z (PMC13365543; doi:10.1038/s44321-026-00455-z)
Supplement: Supplementary file 2 — Table EV2 [file 44321_2026_455_MOESM2_ESM.docx]

**Table EV2**

Diversity and sequencing metrics across selection rounds of E-A functional screening

from P53 VHH screening NGS data

| Sample | row | S_obs | S_obs_ge2 | Chao1 | expH | invSimpson | Top100_cov% | Top100_reads | Total  Reads |
| --- | --- | --- | --- | --- | --- | --- | --- | --- | --- |
| EA0 | 3.14E+05 | 3.14E+05 | 9.40E+04 | 1.02E+06 | 1.02E+03 | 2.66E+01 | 59.37 | 3.43E+06 | 5.78E+06 |
| EA1 | 2.29E+05 | 2.29E+05 | 6.72E+04 | 7.59E+05 | 6.84E+02 | 2.13E+01 | 60.99 | 2.97E+06 | 4.86E+06 |
| EA2 | 1.78E+05 | 1.78E+05 | 5.80E+04 | 5.04E+05 | 1.88E+02 | 9.10E+00 | 72.56 | 3.43E+06 | 4.73E+06 |

Row: Sample identifier representing the initial library and selection rounds. EA0: Initial alpaca VHH lib-STAR-JC5 cell library. EA1 and EA2: Cell products after rounds 1 and 2 of E-A functional screening, respectively.

S_obs: Number of observed unique amino acid sequences.

S_obs_ge2: Number of unique sequences observed at least twice.

Chao1: Chao1 estimator, predicting the lower bound of total sequence richness.

expH: Exponential of Shannon entropy, representing the effective number of sequences.

invSimpson: Inverse Simpson index, a measure of diversity that weights towards dominant sequences.

Top100_cov%: Cumulative relative abundance (%) of the top 100 most abundant unique sequences.

Top100_reads: Total number of sequencing reads mapping to the top 100 most abundant unique sequences.

TotalReads: Total number of high-quality sequencing reads after preprocessing for each sample.
