# Supplementary material for: Functional screening of TCR-like antibodies using STAR-T cell library for cancer immunotherapy
Source: EMBO Mol Med. 2026 Jun 8;18(7):2748–76. doi: 10.1038/s44321-026-00455-z (PMC13365543; doi:10.1038/s44321-026-00455-z)
Supplement: Supplementary file 3 — Table EV3 [file 44321_2026_455_MOESM3_ESM.docx]

**Table EV3**

Abundance of amino acid sequences (%) from phage display-enriched clones

determined by P53 VHH screening NGS

| Target ID | P0 | P1 | P4 | P5 | P6 |
| --- | --- | --- | --- | --- | --- |
| P53-1 Concat. | 0 | 0.0037 | 0.20194 | 10.1598 | 18.9554 |
| P53-2 Concat. | 2.00E-06 | 3.00E-06 | 1.40E-05 | 0.017251 | 0.017381 |
| P53-3 Concat. | 3.00E-06 | 2.00E-06 | 0.003596 | 0.040563 | 0.010557 |

P0: Initial phage library. P1, P4, P5, P6: Phage pools after rounds 1, 4, 5, and 6 of bio-panning, respectively (rounds 2 and 3 were not sequenced). Concat.: The listed VHH sequences were derived from the concatenation of the 50 N-terminal and 50 C-terminal amino acids from NGS-PE150 reads, as the ~380 bp full-length VHHs were not fully covered by sequencing.
