# Supplementary material for: Functional screening of TCR-like antibodies using STAR-T cell library for cancer immunotherapy
Source: EMBO Mol Med. 2026 Jun 8;18(7):2748–76. doi: 10.1038/s44321-026-00455-z (PMC13365543; doi:10.1038/s44321-026-00455-z)
Supplement: Supplementary file 4 — Table EV4 [file 44321_2026_455_MOESM4_ESM.docx]

**Table EV4**

Abundance of amino acid sequences (%) from E-A functional screening-enriched clones

determined by P53 VHH screening NGS

| Target ID | E0 | E1 | E2 | E3 |
| --- | --- | --- | --- | --- |
| P53-1 | 17.905064 | 20.346 | 22.8498 | 32.3249 |
| P53-2 | 0.022713 | 0.049749 | 0.268138 | 0.190689 |
| P53-3 | 0.098401 | 0.10287 | 0.218112 | 0.228075 |

EA0: Initial cell library. EA1 and EA2: Cell products after rounds 1 and 2 of E-A functional screening, respectively. The listed VHH sequences are full-length, as determined by NGS-PE250 sequencing.
