# Supplementary material for: Functional screening of TCR-like antibodies using STAR-T cell library for cancer immunotherapy
Source: EMBO Mol Med. 2026 Jun 8;18(7):2748–76. doi: 10.1038/s44321-026-00455-z (PMC13365543; doi:10.1038/s44321-026-00455-z)
Supplement: Supplementary file 5 — Table EV5 [file 44321_2026_455_MOESM5_ESM.docx]

**Table EV5**

Sequence of VHHs identified from functional screening platform in this study.

| ID | VHH amino acid sequence (CDR3 is highlighted) |
| --- | --- |
| CD22-1 | DVQLQESGGGSVQAGGSLRLSCAASGATFSSLATAWFRQGPGKERVIVAALGWSGPTEYYSDSVRGRFTISRDIAKNTMYLQMNSLNSDDTAVYYCAA**DPSFDTPWYRYAY**WGQGTQVTVSS |
| CD22-2 | DVQLQESGGGLVQPGGSLRLSCAASGATFSSLATAWFRQGPGKERVIVAALGWSGPTEYYSDSVRGRFTISRDIAKNTMYLQMNSLNSDDTAVYYCAA**DPSFDTPWYRYAY**WGQGTQVTVSS |
| CD22-3 | DVQLQESGGGLVESGGSLRLSCAASGFTFSGYWMYWVRRAPGKGLEWVSAINTGGGSTYYADSVKDRFTITRNVAEATVYLQMNNLKPEDTAVYYCST**YFEGVQY**WGQGTQVTVSS |
| CD22-4 | DVQLQESGGGLVQPGGSLRLSCAASGINLDYYSVGWFRQVPGKEREGVACITRGSTSVRKKESVKDRFFVSRDDAKTTVYLEMDSLKPEDTAVYYCAA**GVLSVCELTDSYQY**WGQGTQVTVSS |
| CD22-5 | DVQLQESGGGLVQAGQSLTLSCVASGYTFNGTPMGWFRQAPGKEREFIAGTRRRGQRTYSTDYADSVKGRFFISRSDARKTIYLQMSSLKPEDTAVYSCAG**AVDAEVYNH**WGQGTQVTVSS |
| CD22-6 | DVQLQESGGGLVQTGGSLRLSCAASGATFSSLATAWFRQGPGKERVIVAALGWSGPTEYYSDSVRGRFTISRDIAKNTMYLQMNSLNSDDTAVYYCAA**DPSFDTPWYRYAY**WGQGTQVTVSS |
| CD22-7 | DVQLQESGGGLVQAGGSLRLSCAASGIILAANDMGWYRQAPGKERELVGRISGGGSTFYADAVKGRFTISRDNAKNTLYLQMNSLQPADTAVYFCHA**PALVVFGTGS**WGQGTQVTVSS |
| CD22-8 | DVQLQESGGGLVRPGGSLRLSCAASGINLDYYSVGWFRQVPGKEREGVACITRGSTSVRKKESVKDRFFVSRDDAKTTVYLEMDSLKPEDTAVYYCAA**GVLSVCELTDSYQY**WGQGTQVTVSS |
| CD22-9 | DVQLQESGGGLVQAGGSLRLSCAASGRPGDGTAMGWFRQAPGKEREFVAAISWSGTRKDYADSVKDRFAITTDNTKNTAYLQLNSLKPEDTAVYYCAA**KIATNPWAVFTTSYDYSD**WGQGTQVTVSS |
| CD22-10 | DVQLQESGGGLVQAGDSLRLSCAASGRTFSAYAMGWFRQAPGKEREFVAAISWSAGITYYADSVKGRFATSKDNAKNTVYLQMNSLKPEDTAVYYCAA**RLRLGILMVSSGYDY**WGQGTQVTVSS |
| P53-1 | DVQLQESGGGLVQAGDSLRLSCAASGRTFSSYTMGWFRRPPGKEREFVASVSGSGSSTYYADSVKGRFTISRDNVKNTVYLQMNSLKPEDTAVYYCNL**AGYPDDY**WGQGTQVTVSS |
| P53-2 | DVQLQESGGGLVQPGGSLRLSCAASGFTFSSYAMSWVRQAPGKGAEWVSTIYPYSSNTYYADSVKGRFTISRDNTKSAVYLQMVNLKPEDTAVYYCNA**GGNPWDENDY**WGQGTQVTVSS |
| P53-3 | DVQLQESGGGLVQTGGSLRLSCAASGLTVSNYDIGWFRQAPGKEREIVASIRWSGSTYYVDSVKGRFTISRDSAKNTVYLQMNSLKPEDTAVYFCNA**ELRAVTSTRERNFEL**WGQGTQVTVSS |
| P53-4 | DVQLQESGGGLVQPGGSLRLSCVASGFTFSDYYMSWVRQAPGKGLEWVSSVYGDGSTTYYADSVKGRFTISRDNAKNTVYLQMNSLKPEDTALYICTV**YVHDLHWGTPYDV**WGRGTQVTVSS |
| P53-5 | DVQLQESGGGLVRPGESLRLSCAASGRLFGITTMGWYRQAPGKRRELVAEVTSGGTKNYADSAKGRFTISREDSRKVFLQMDNLKPEDTAVYYCKA**DKASINWYEPPNHY**WGQGTQVTVSS |
