# Supplementary figures and images for: Functional screening of TCR-like antibodies using STAR-T cell library for cancer immunotherapy (part 1 of 2)
Source: EMBO Mol Med. 2026 Jun 8;18(7):2748–76. doi: 10.1038/s44321-026-00455-z (PMC13365543; doi:10.1038/s44321-026-00455-z)

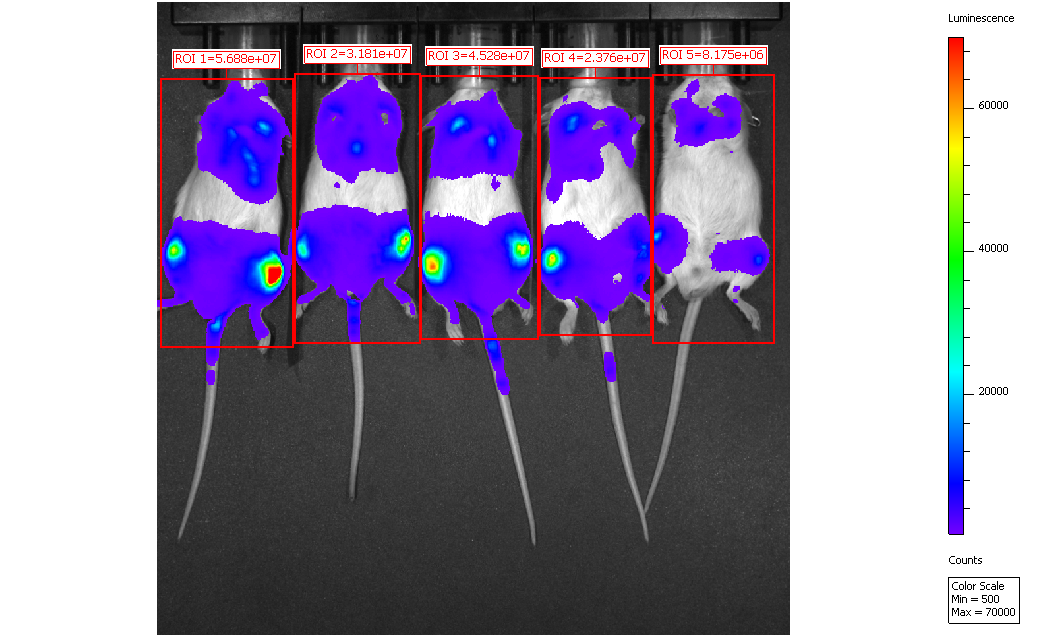

Supplement: Supplementary file 10 — Source data Fig. 4 [file 44321_2026_455_MOESM10_ESM.zip › Figure4/Panel B/DAY 11/1- 47 99 84 94 95 luc.tif]

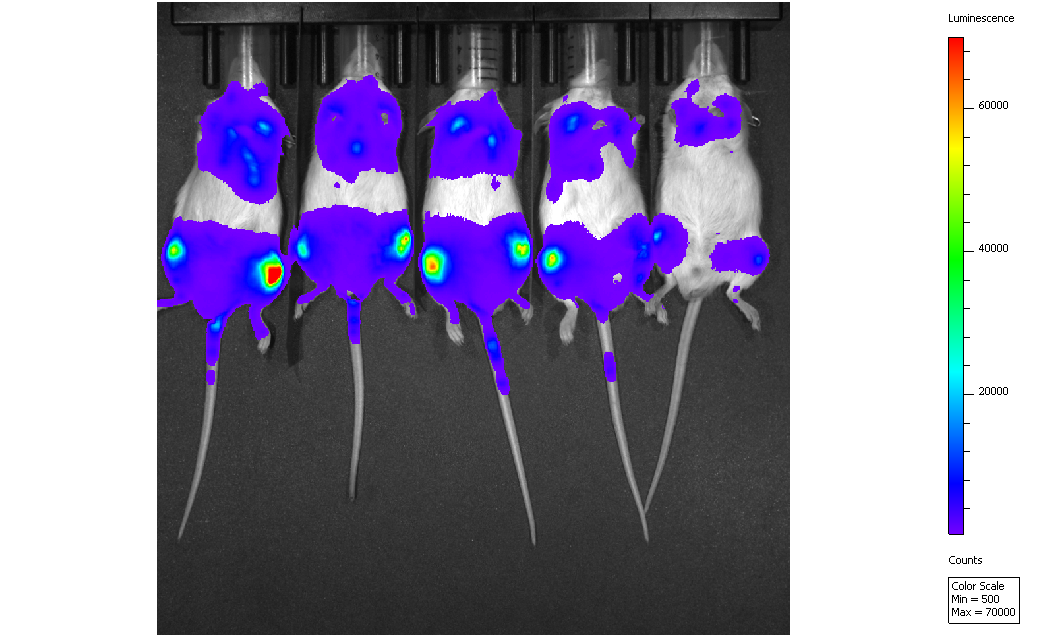

Supplement: Supplementary file 10 — Source data Fig. 4 [file 44321_2026_455_MOESM10_ESM.zip › Figure4/Panel B/DAY 11/1- 47 99 84 94 95 -2.tif]

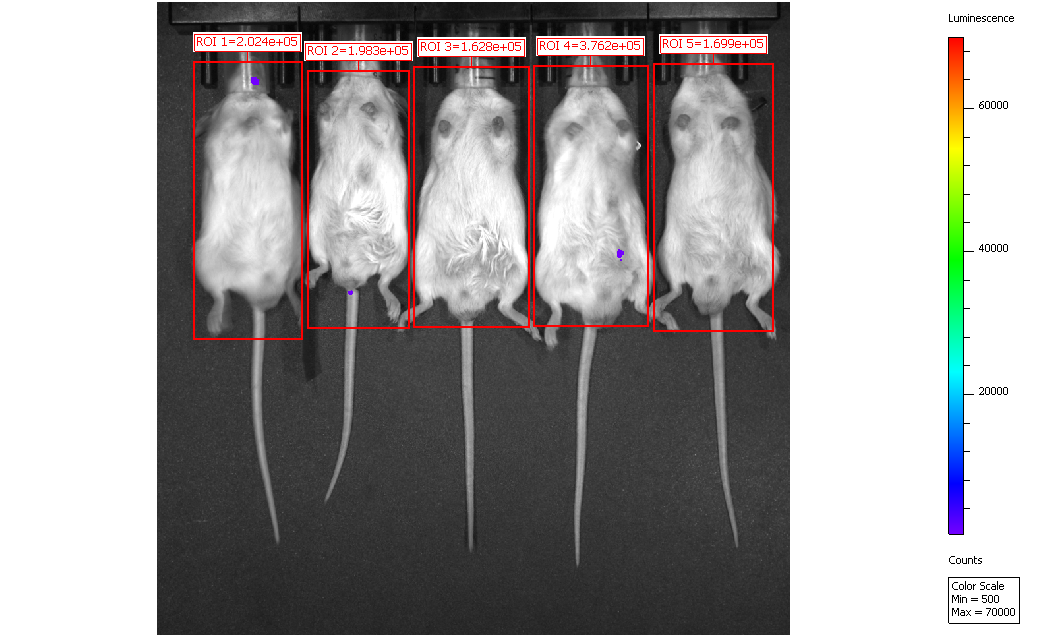

Supplement: Supplementary file 10 — Source data Fig. 4 [file 44321_2026_455_MOESM10_ESM.zip › Figure4/Panel B/DAY 11/2- 78 96 98 86 76 luc.tif]

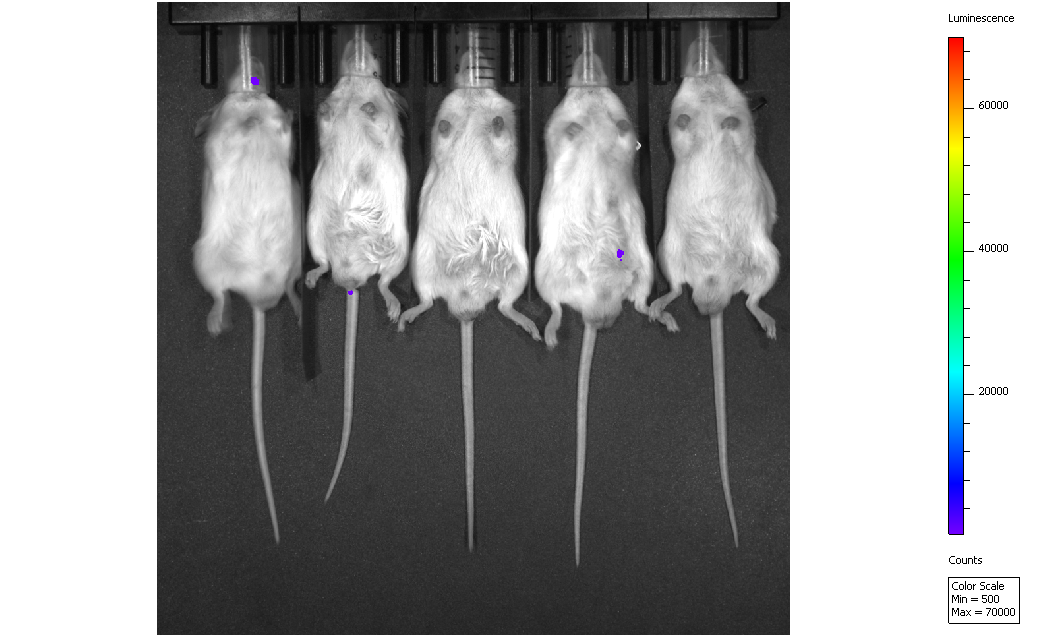

Supplement: Supplementary file 10 — Source data Fig. 4 [file 44321_2026_455_MOESM10_ESM.zip › Figure4/Panel B/DAY 11/2- 78 96 98 86 76.tif]

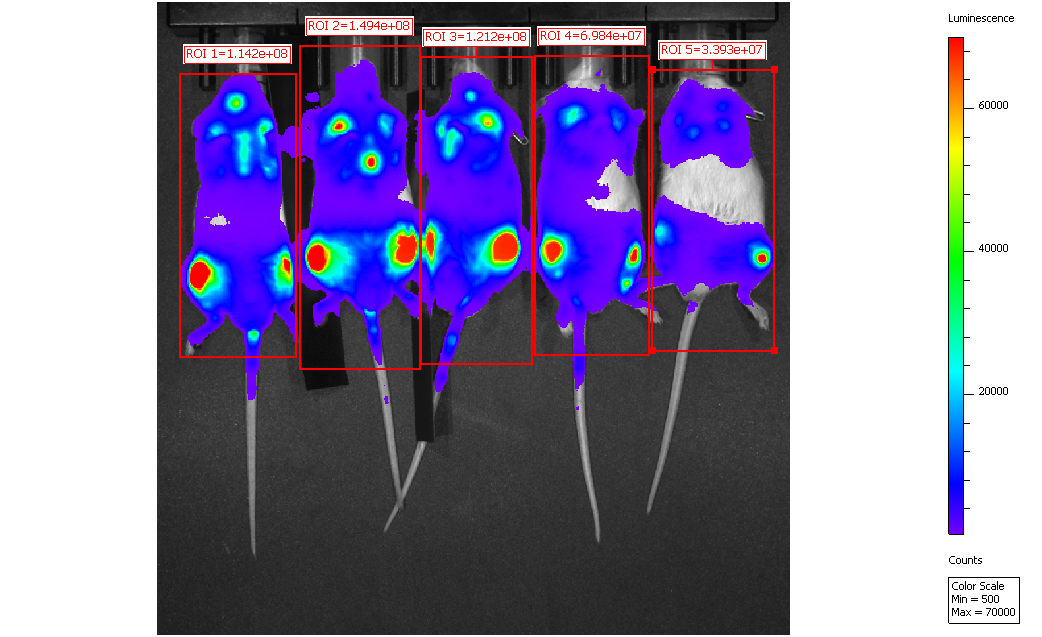

Supplement: Supplementary file 10 — Source data Fig. 4 [file 44321_2026_455_MOESM10_ESM.zip › Figure4/Panel B/DAY 14/1- 47 99 84 94 95 luc.tif]

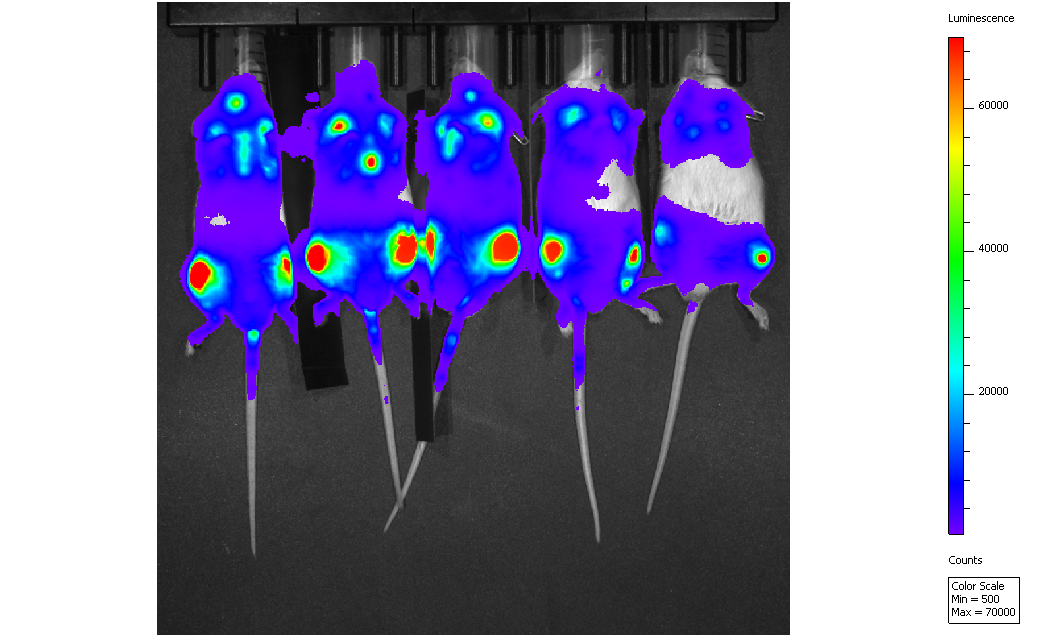

Supplement: Supplementary file 10 — Source data Fig. 4 [file 44321_2026_455_MOESM10_ESM.zip › Figure4/Panel B/DAY 14/1- 47 99 84 94 95.tif]

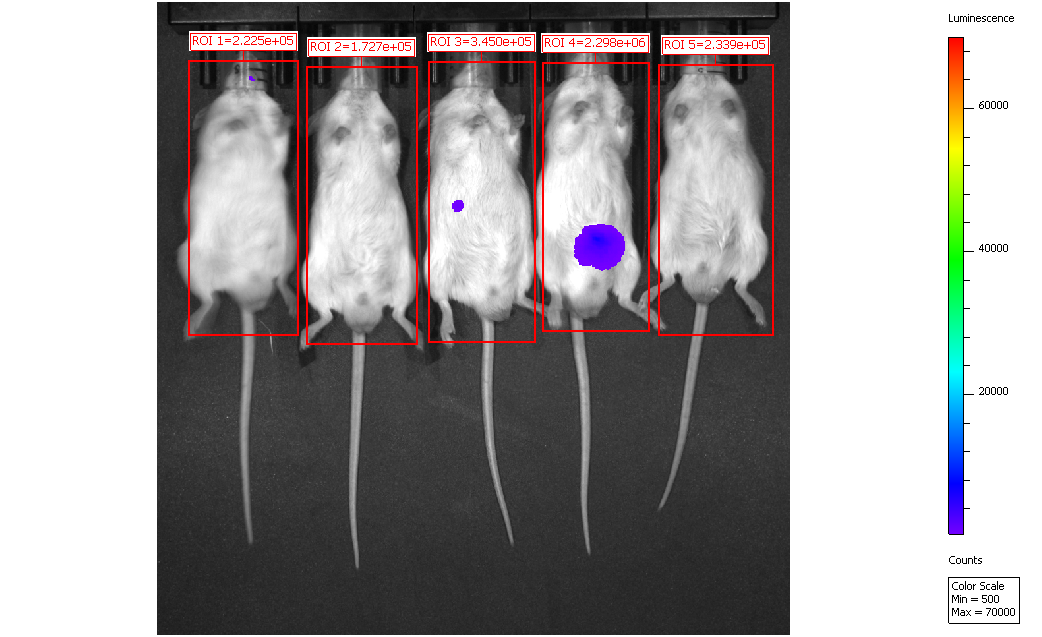

Supplement: Supplementary file 10 — Source data Fig. 4 [file 44321_2026_455_MOESM10_ESM.zip › Figure4/Panel B/DAY 14/2- 78 96 98 86 76 luc.tif]

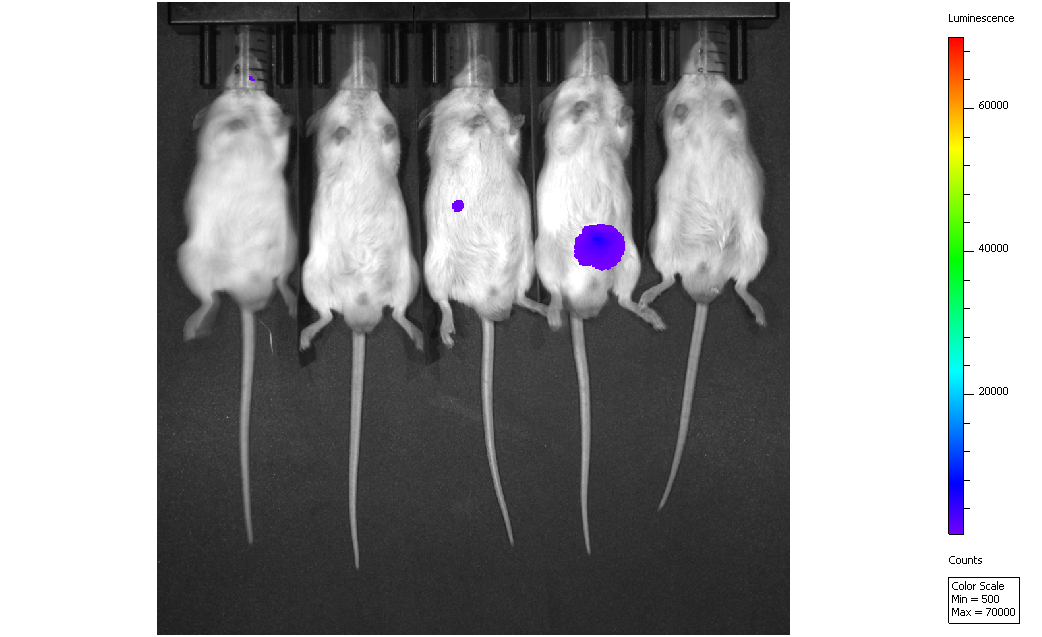

Supplement: Supplementary file 10 — Source data Fig. 4 [file 44321_2026_455_MOESM10_ESM.zip › Figure4/Panel B/DAY 14/2- 78 96 98 86 76.tif]

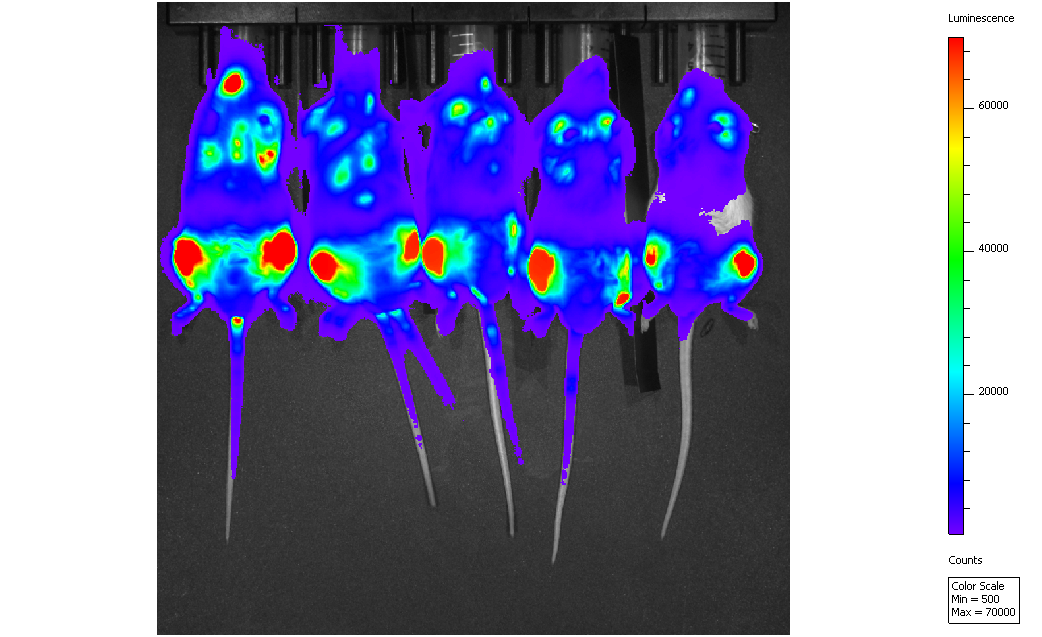

Supplement: Supplementary file 10 — Source data Fig. 4 [file 44321_2026_455_MOESM10_ESM.zip › Figure4/Panel B/DAY 17/1- 47 99 84 94 95.tif]

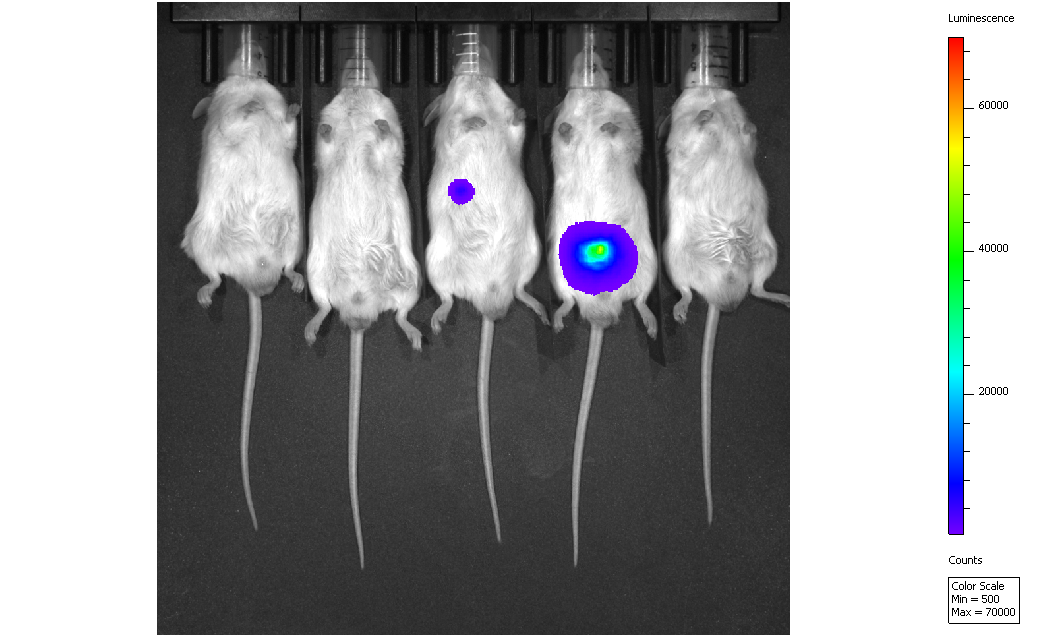

Supplement: Supplementary file 10 — Source data Fig. 4 [file 44321_2026_455_MOESM10_ESM.zip › Figure4/Panel B/DAY 17/2- 78 96 98 86 76.tif]

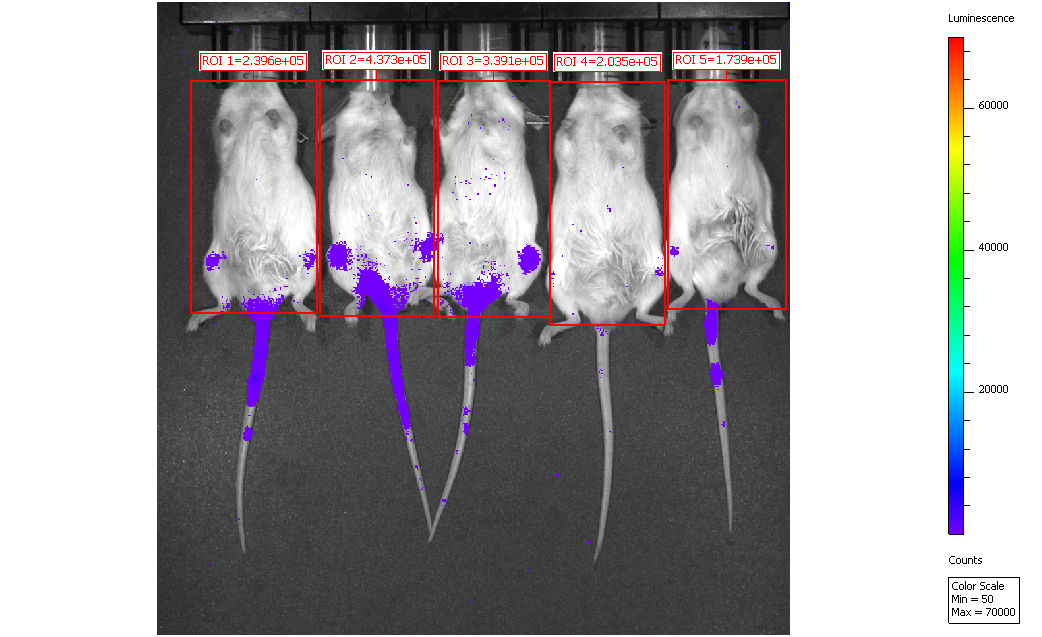

Supplement: Supplementary file 10 — Source data Fig. 4 [file 44321_2026_455_MOESM10_ESM.zip › Figure4/Panel B/DAY 4/1- 76-80 luc.tif]

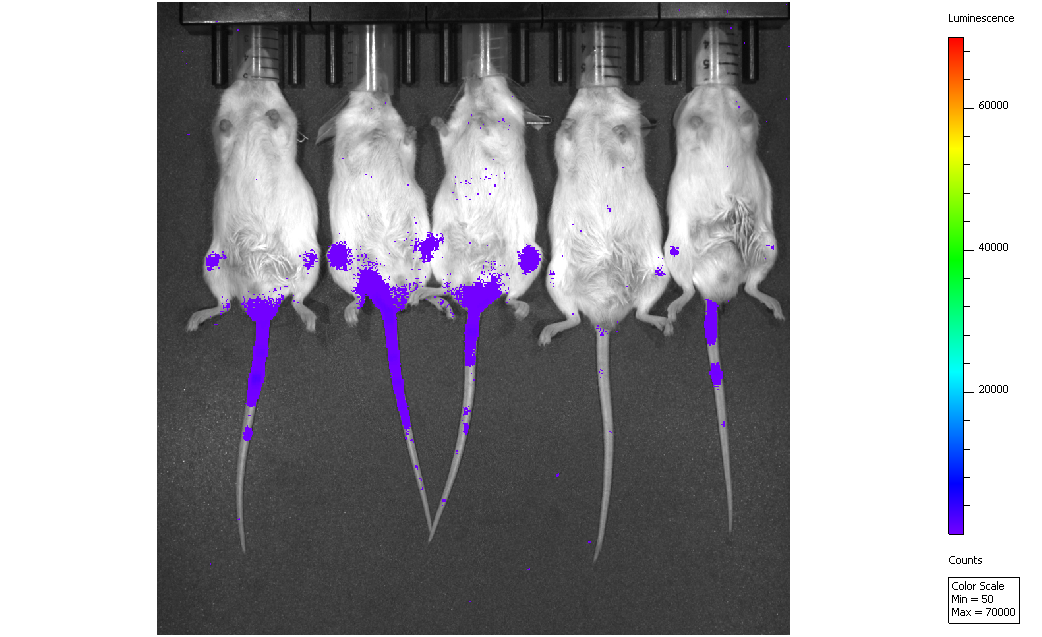

Supplement: Supplementary file 10 — Source data Fig. 4 [file 44321_2026_455_MOESM10_ESM.zip › Figure4/Panel B/DAY 4/1- 76-80.tif]

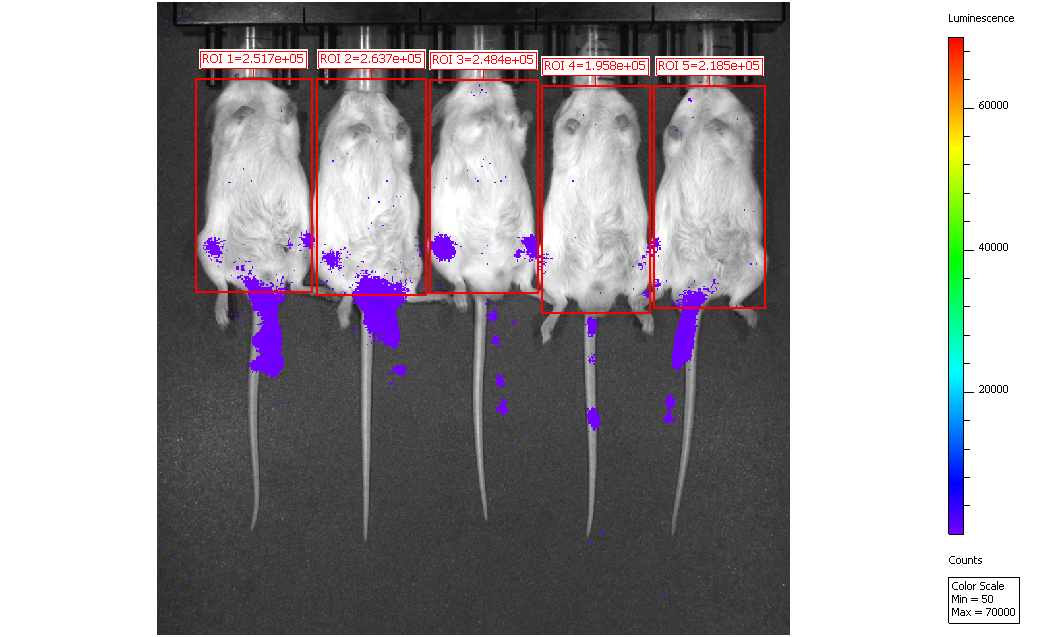

Supplement: Supplementary file 10 — Source data Fig. 4 [file 44321_2026_455_MOESM10_ESM.zip › Figure4/Panel B/DAY 4/10-66-70 luc.tif]

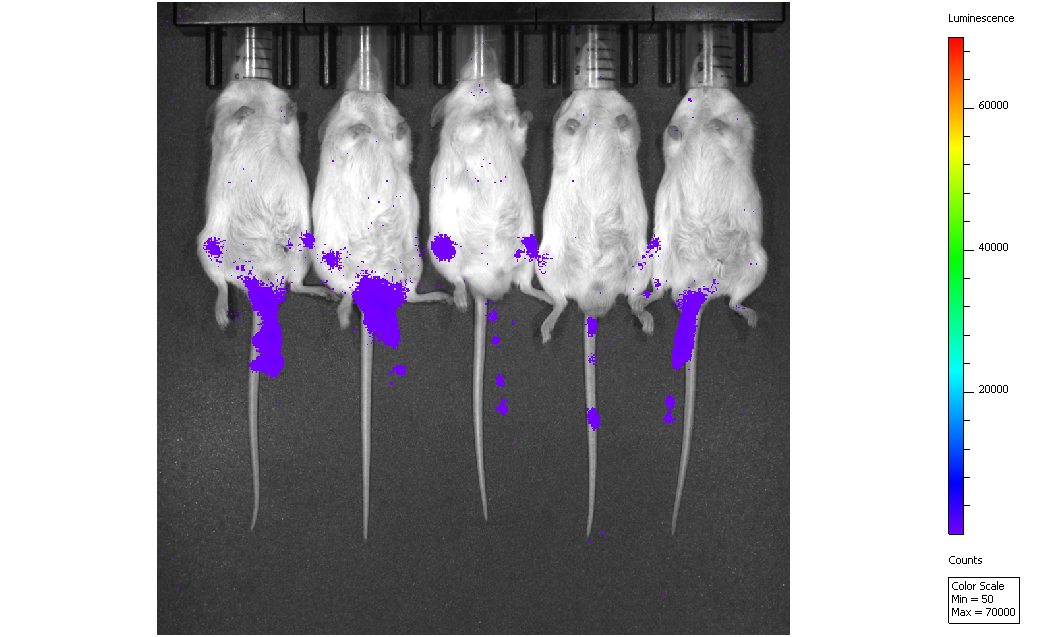

Supplement: Supplementary file 10 — Source data Fig. 4 [file 44321_2026_455_MOESM10_ESM.zip › Figure4/Panel B/DAY 4/10-66-70.tif]

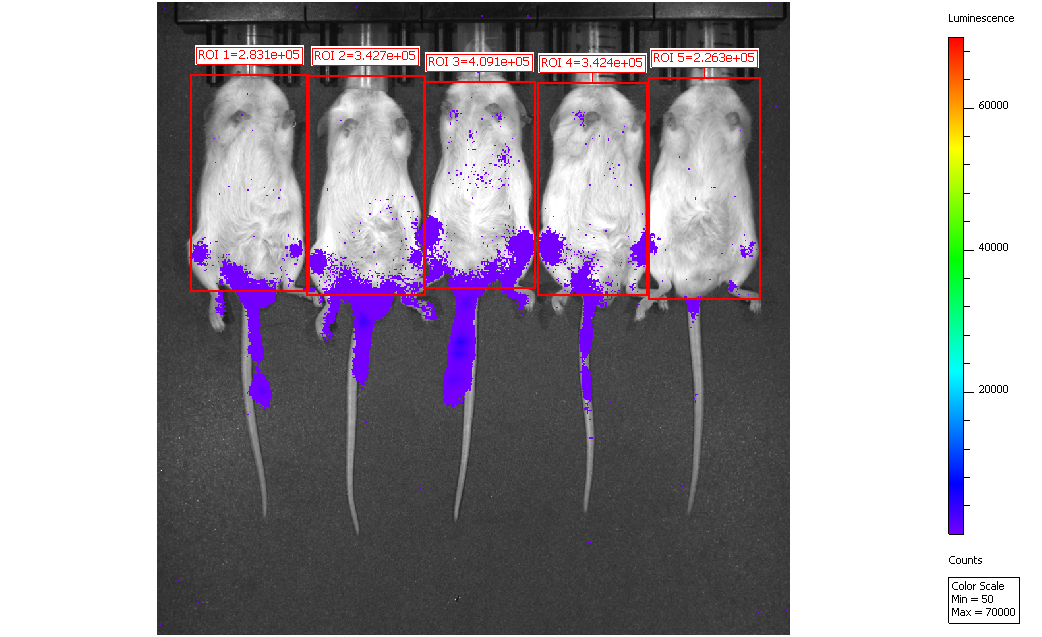

Supplement: Supplementary file 10 — Source data Fig. 4 [file 44321_2026_455_MOESM10_ESM.zip › Figure4/Panel B/DAY 4/11- 71-75 luc.tif]

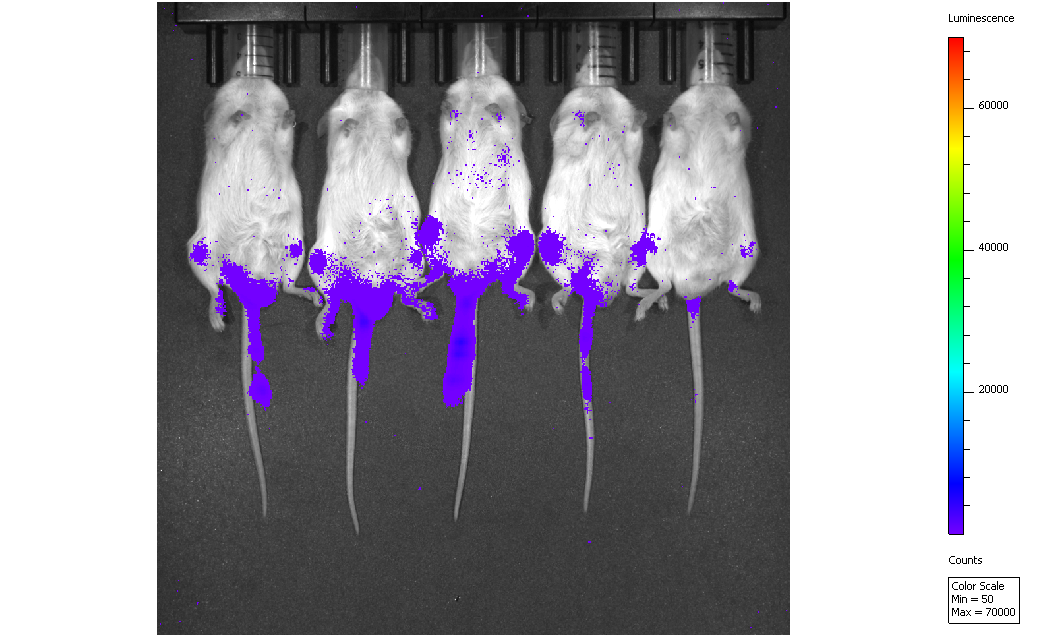

Supplement: Supplementary file 10 — Source data Fig. 4 [file 44321_2026_455_MOESM10_ESM.zip › Figure4/Panel B/DAY 4/11- 71-75.tif]

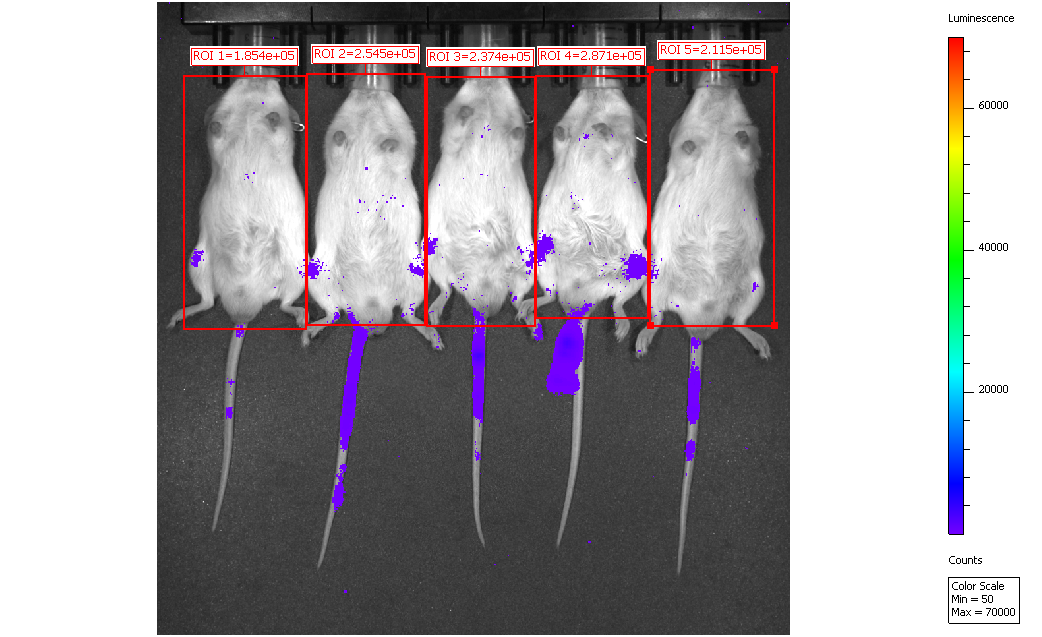

Supplement: Supplementary file 10 — Source data Fig. 4 [file 44321_2026_455_MOESM10_ESM.zip › Figure4/Panel B/DAY 4/2- 81 -85 luc.tif]

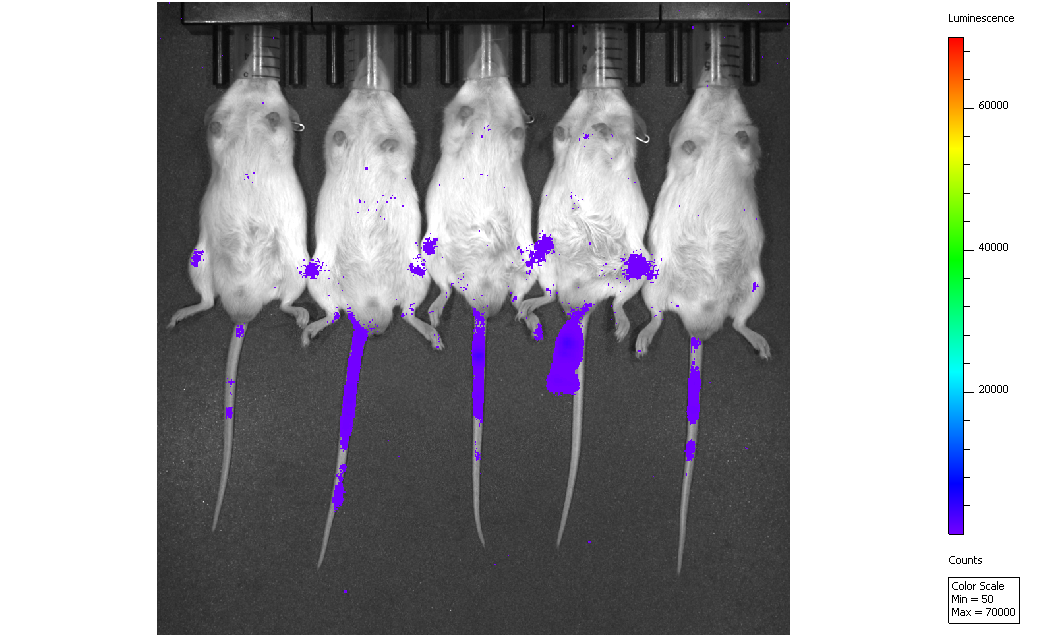

Supplement: Supplementary file 10 — Source data Fig. 4 [file 44321_2026_455_MOESM10_ESM.zip › Figure4/Panel B/DAY 4/2- 81 -85.tif]

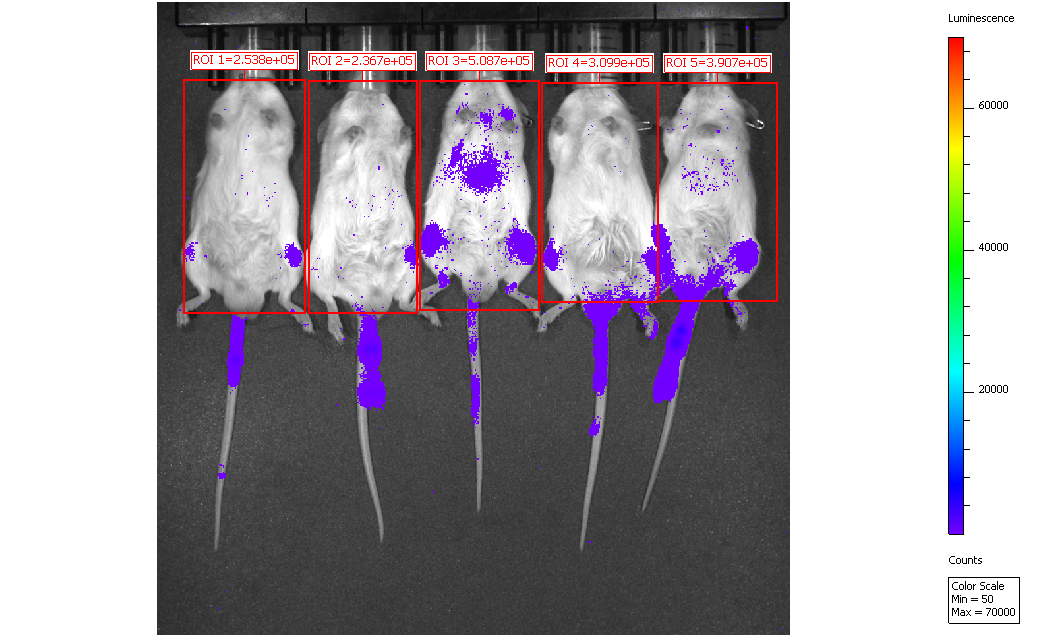

Supplement: Supplementary file 10 — Source data Fig. 4 [file 44321_2026_455_MOESM10_ESM.zip › Figure4/Panel B/DAY 4/3- 86-90 luc.tif]

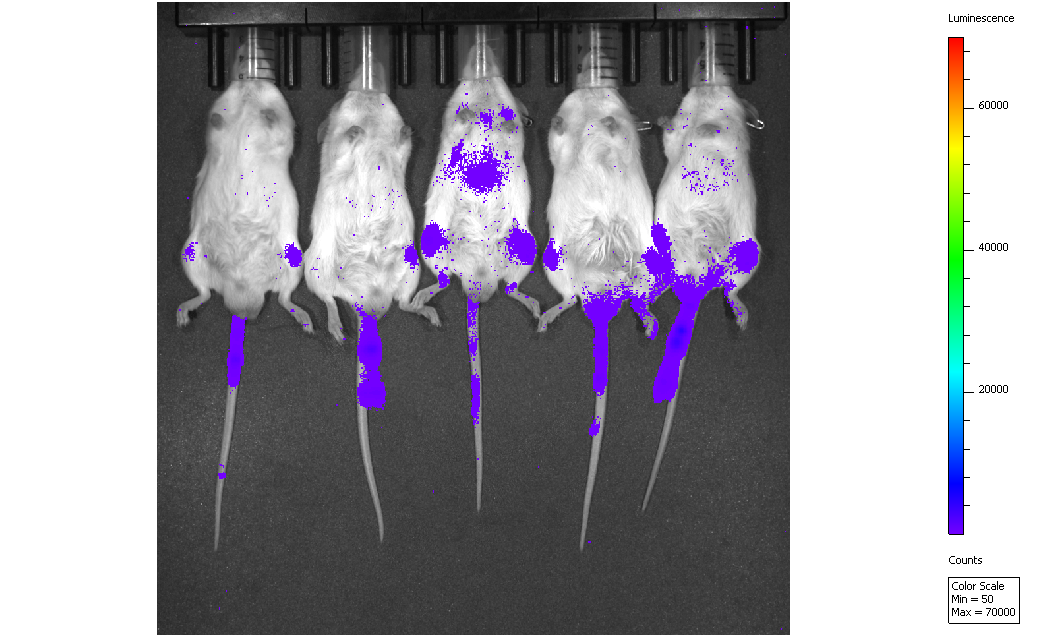

Supplement: Supplementary file 10 — Source data Fig. 4 [file 44321_2026_455_MOESM10_ESM.zip › Figure4/Panel B/DAY 4/3- 86-90.tif]

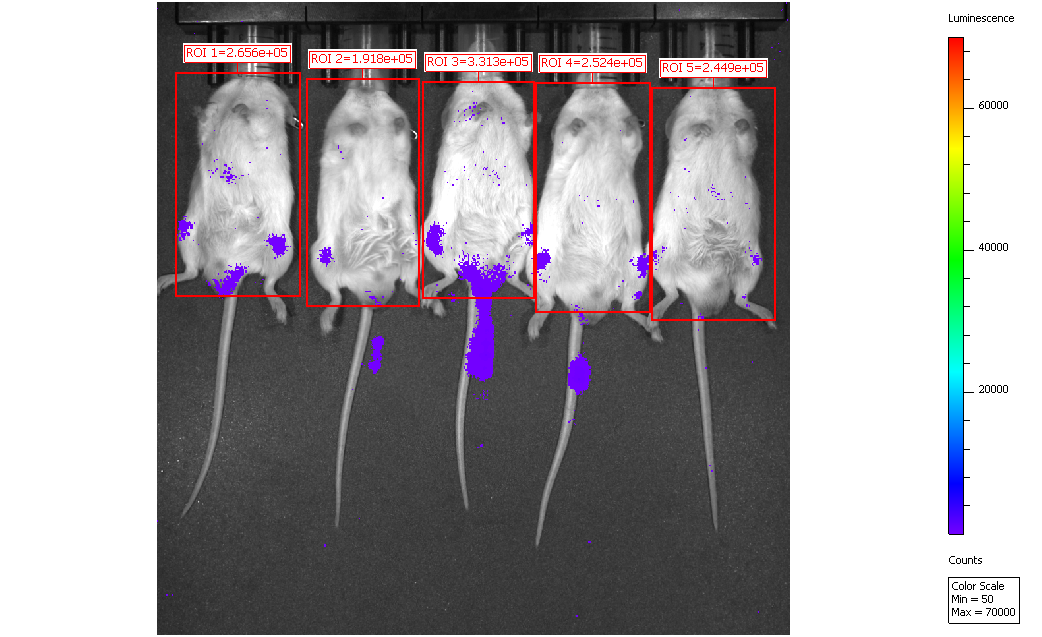

Supplement: Supplementary file 10 — Source data Fig. 4 [file 44321_2026_455_MOESM10_ESM.zip › Figure4/Panel B/DAY 4/4- 91-95 luc.tif]

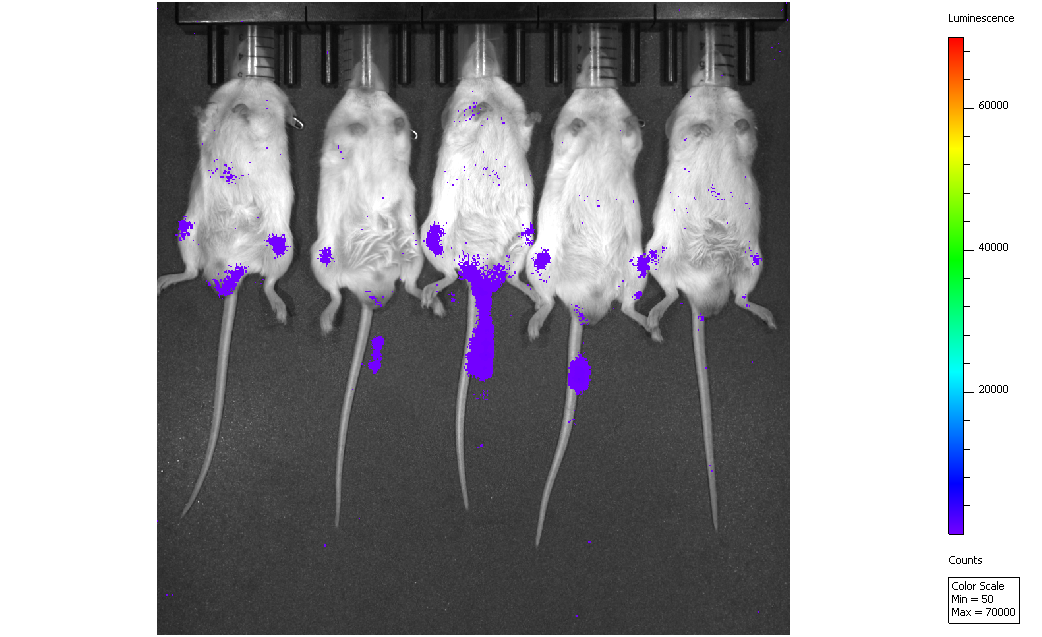

Supplement: Supplementary file 10 — Source data Fig. 4 [file 44321_2026_455_MOESM10_ESM.zip › Figure4/Panel B/DAY 4/4- 91-95.tif]

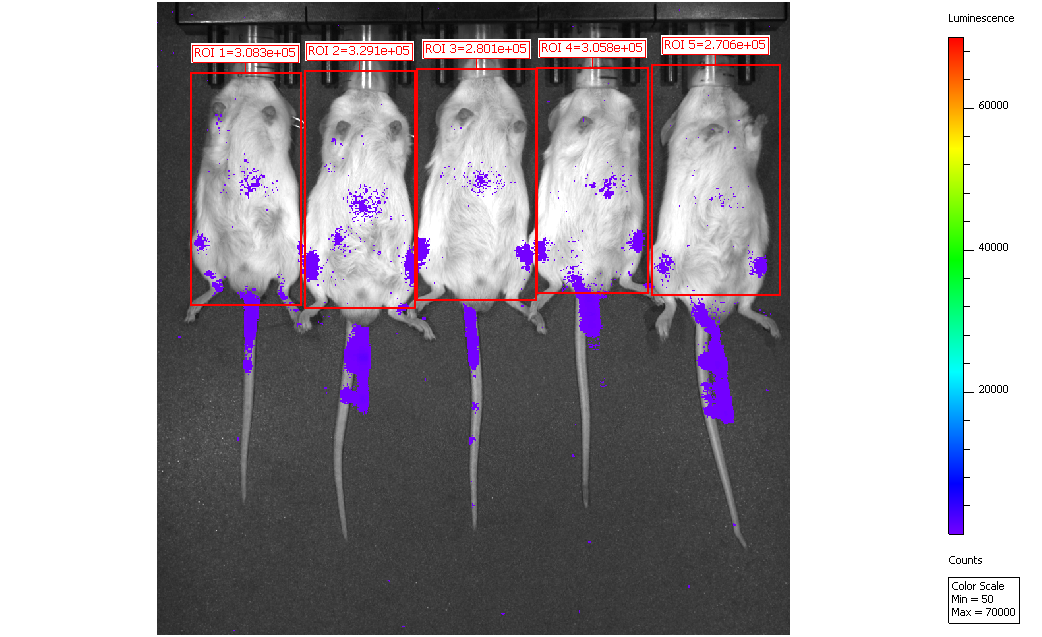

Supplement: Supplementary file 10 — Source data Fig. 4 [file 44321_2026_455_MOESM10_ESM.zip › Figure4/Panel B/DAY 4/5- 96-100 luc.tif]

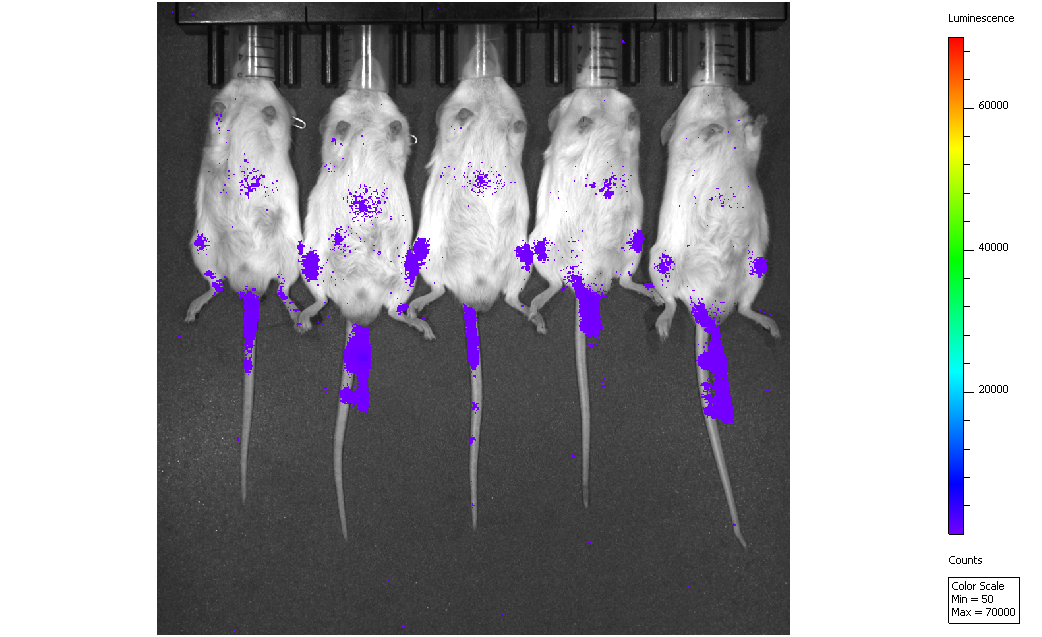

Supplement: Supplementary file 10 — Source data Fig. 4 [file 44321_2026_455_MOESM10_ESM.zip › Figure4/Panel B/DAY 4/5- 96-100.tif]

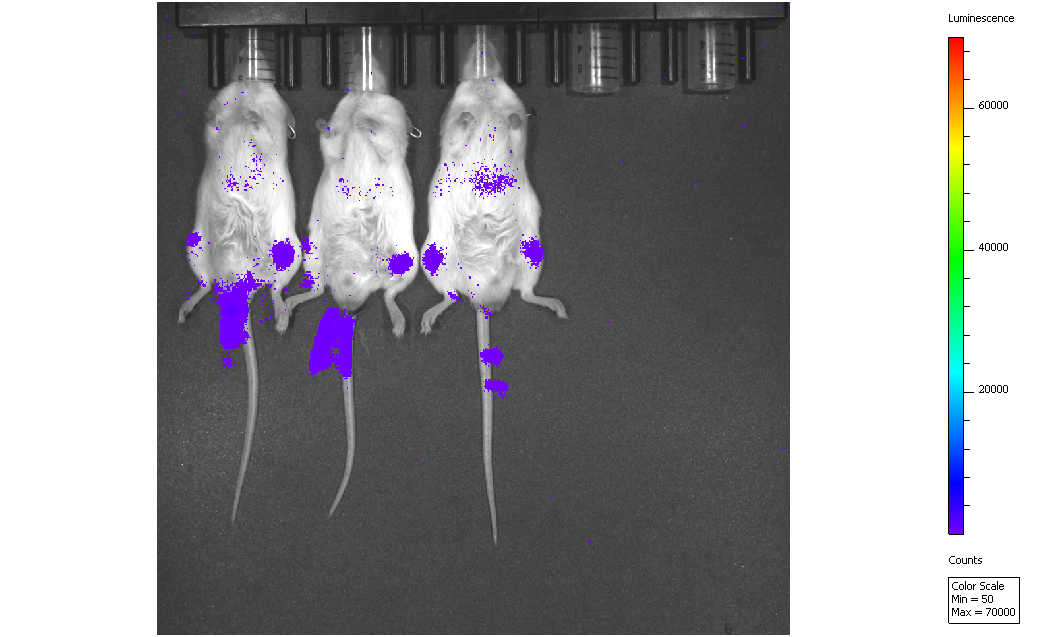

Supplement: Supplementary file 10 — Source data Fig. 4 [file 44321_2026_455_MOESM10_ESM.zip › Figure4/Panel B/DAY 4/6- 47 48 49 .tif]

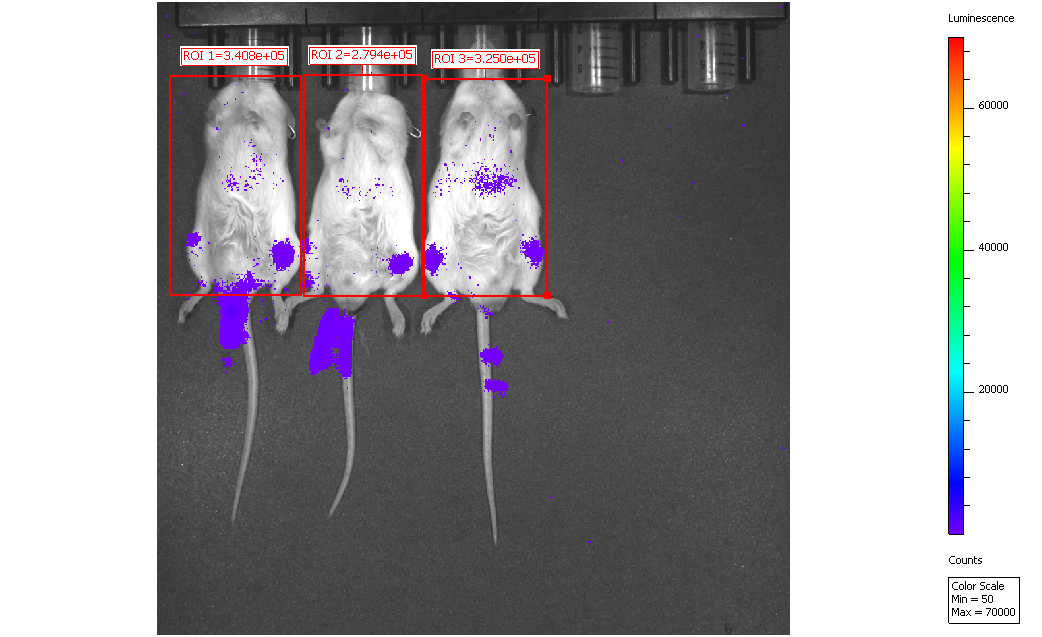

Supplement: Supplementary file 10 — Source data Fig. 4 [file 44321_2026_455_MOESM10_ESM.zip › Figure4/Panel B/DAY 4/6- 47 48 49 luc.tif]

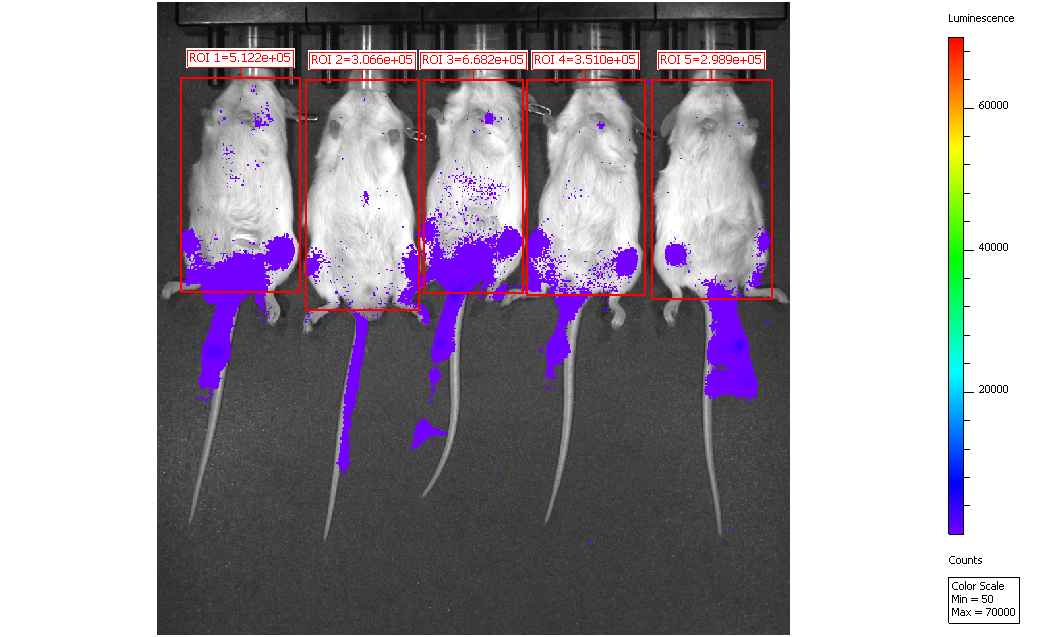

Supplement: Supplementary file 10 — Source data Fig. 4 [file 44321_2026_455_MOESM10_ESM.zip › Figure4/Panel B/DAY 4/7- 51-55 luc.tif]

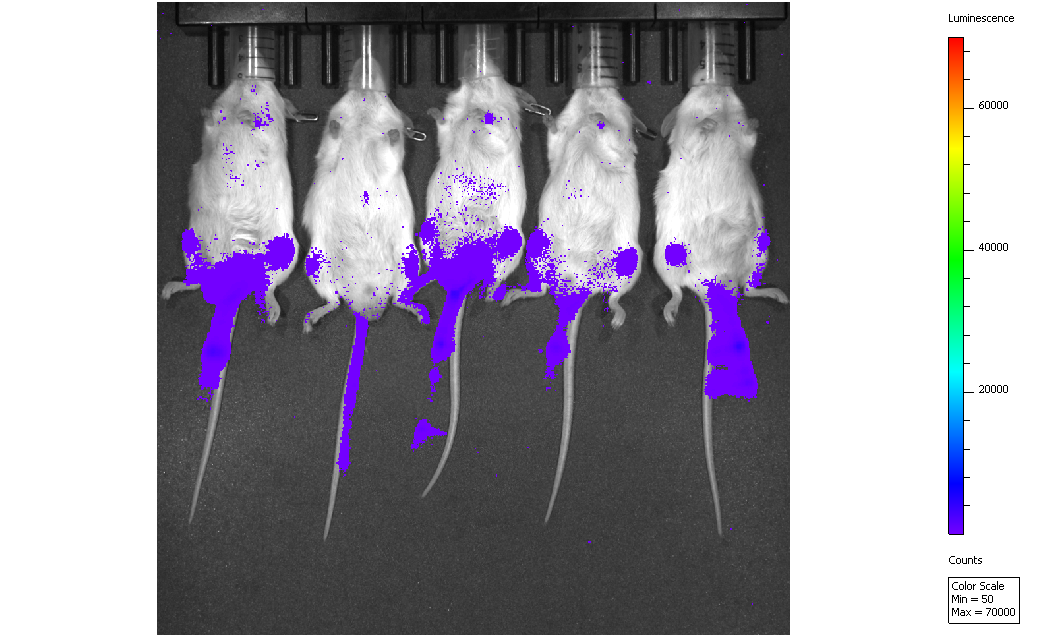

Supplement: Supplementary file 10 — Source data Fig. 4 [file 44321_2026_455_MOESM10_ESM.zip › Figure4/Panel B/DAY 4/7- 51-55.tif]

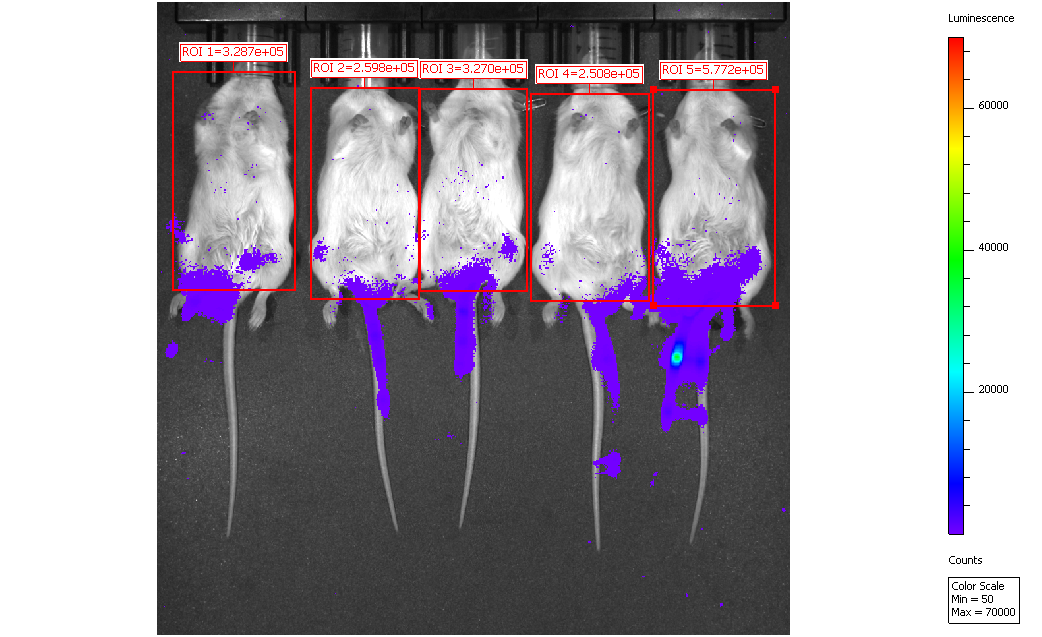

Supplement: Supplementary file 10 — Source data Fig. 4 [file 44321_2026_455_MOESM10_ESM.zip › Figure4/Panel B/DAY 4/8- 56-60 luc.tif]

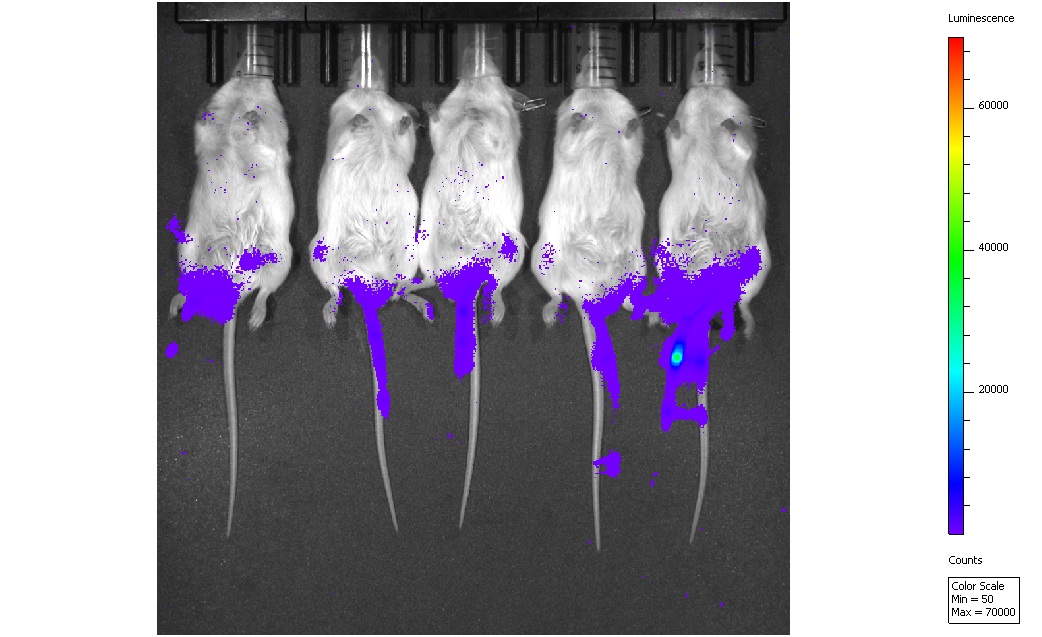

Supplement: Supplementary file 10 — Source data Fig. 4 [file 44321_2026_455_MOESM10_ESM.zip › Figure4/Panel B/DAY 4/8- 56-60.tif]

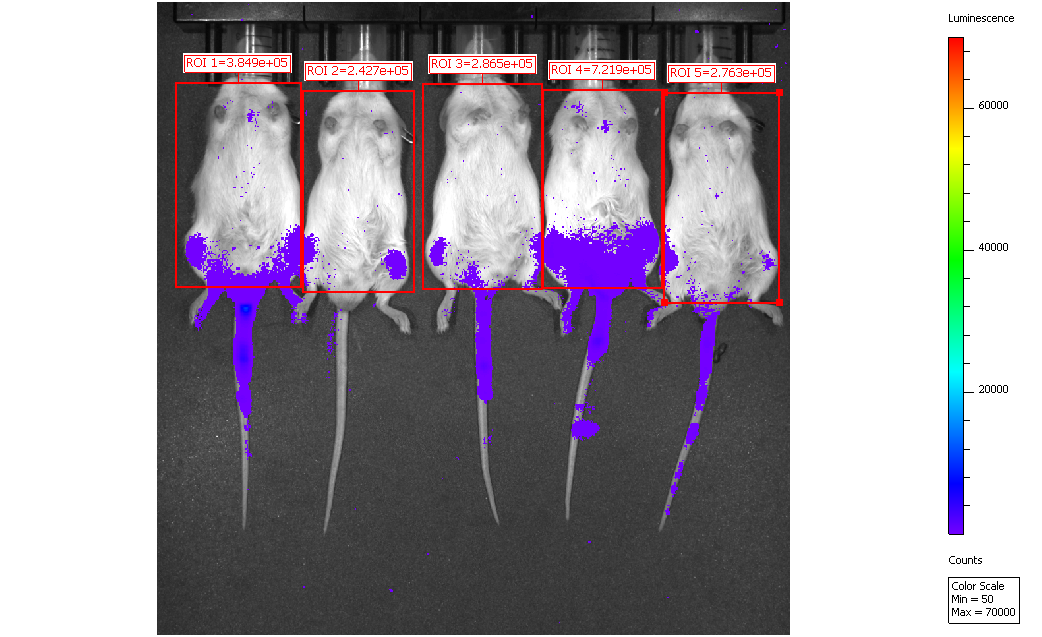

Supplement: Supplementary file 10 — Source data Fig. 4 [file 44321_2026_455_MOESM10_ESM.zip › Figure4/Panel B/DAY 4/9- 61-65 luc.tif]

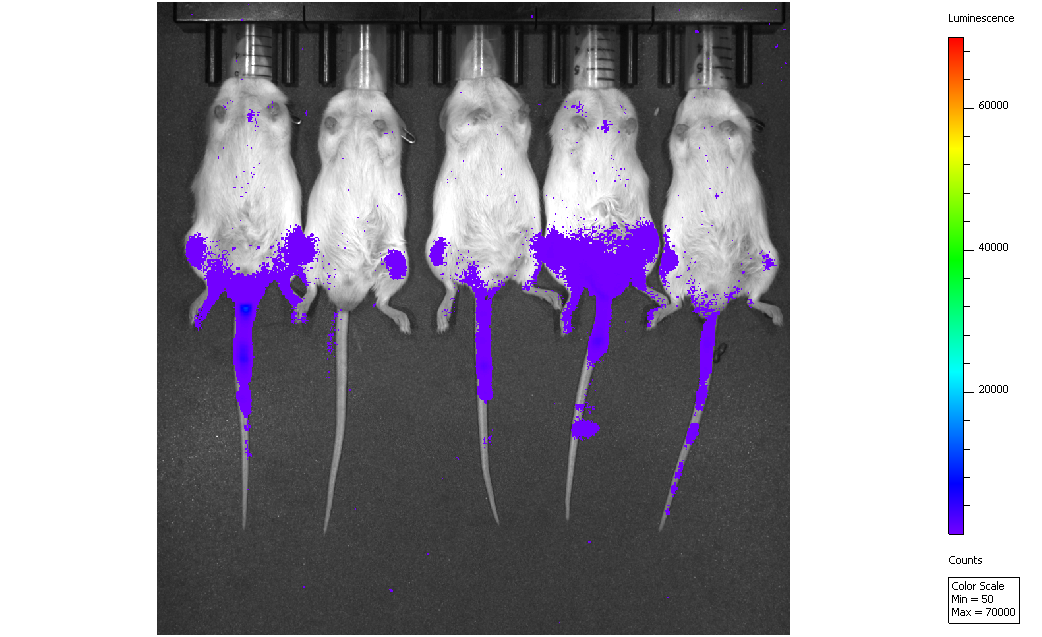

Supplement: Supplementary file 10 — Source data Fig. 4 [file 44321_2026_455_MOESM10_ESM.zip › Figure4/Panel B/DAY 4/9- 61-65.tif]

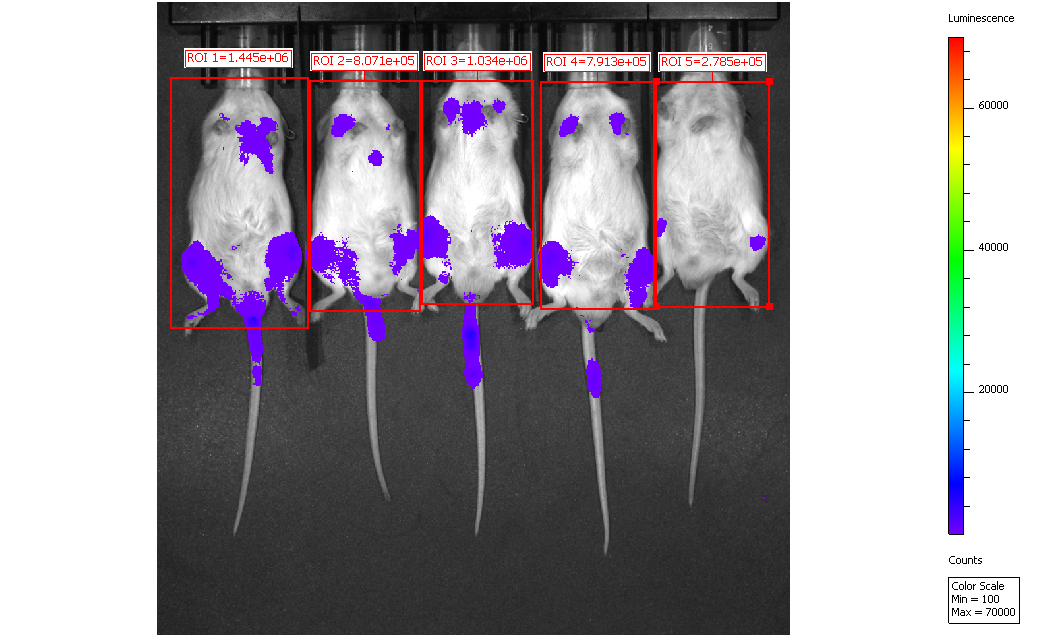

Supplement: Supplementary file 10 — Source data Fig. 4 [file 44321_2026_455_MOESM10_ESM.zip › Figure4/Panel B/DAY 7/1- 47 99 84 94 95 -1 luc.tif]

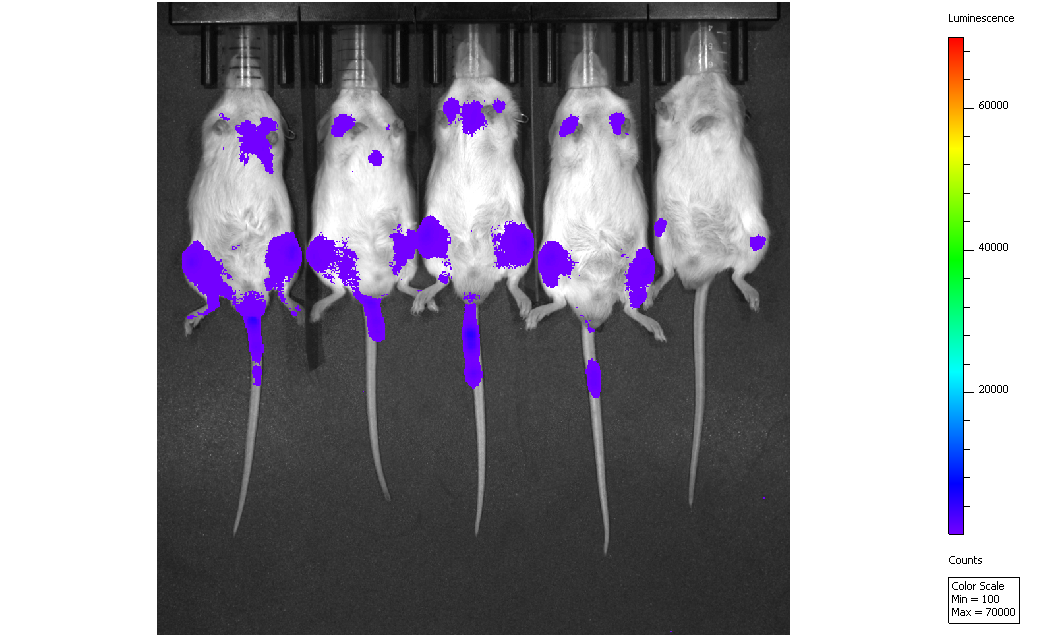

Supplement: Supplementary file 10 — Source data Fig. 4 [file 44321_2026_455_MOESM10_ESM.zip › Figure4/Panel B/DAY 7/1- 47 99 84 94 95 -1.tif]

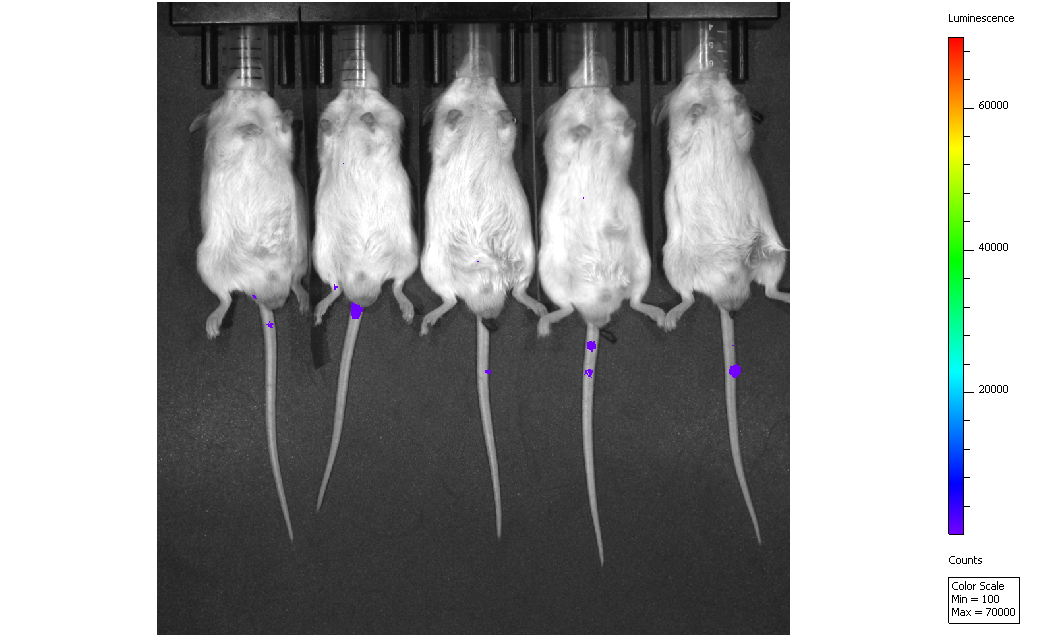

Supplement: Supplementary file 10 — Source data Fig. 4 [file 44321_2026_455_MOESM10_ESM.zip › Figure4/Panel B/DAY 7/2- 78 96 98 86 76 -1.tif]

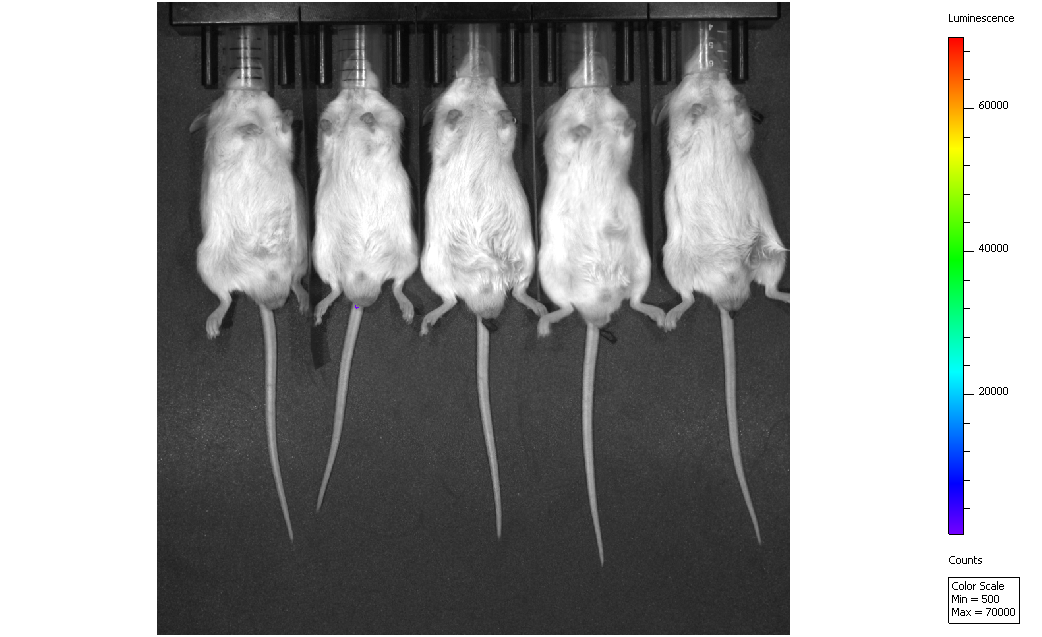

Supplement: Supplementary file 10 — Source data Fig. 4 [file 44321_2026_455_MOESM10_ESM.zip › Figure4/Panel B/DAY 7/2- 78 96 98 86 76 -2.tif]

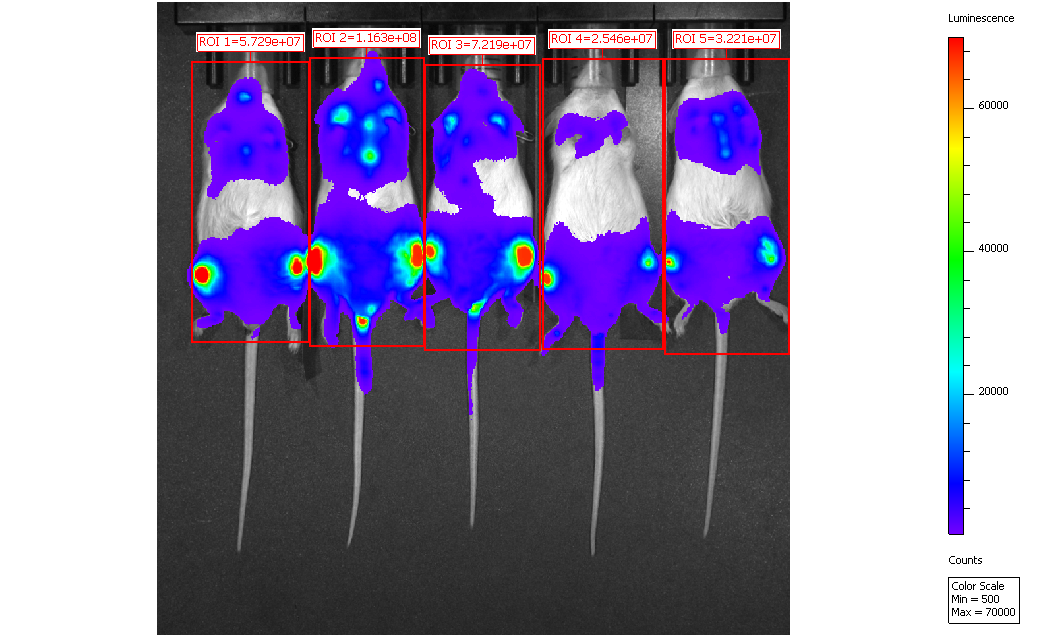

Supplement: Supplementary file 10 — Source data Fig. 4 [file 44321_2026_455_MOESM10_ESM.zip › Figure4/Panel F/DAY 11/6- mock2- 68 72 75 70 69 luc.tif]

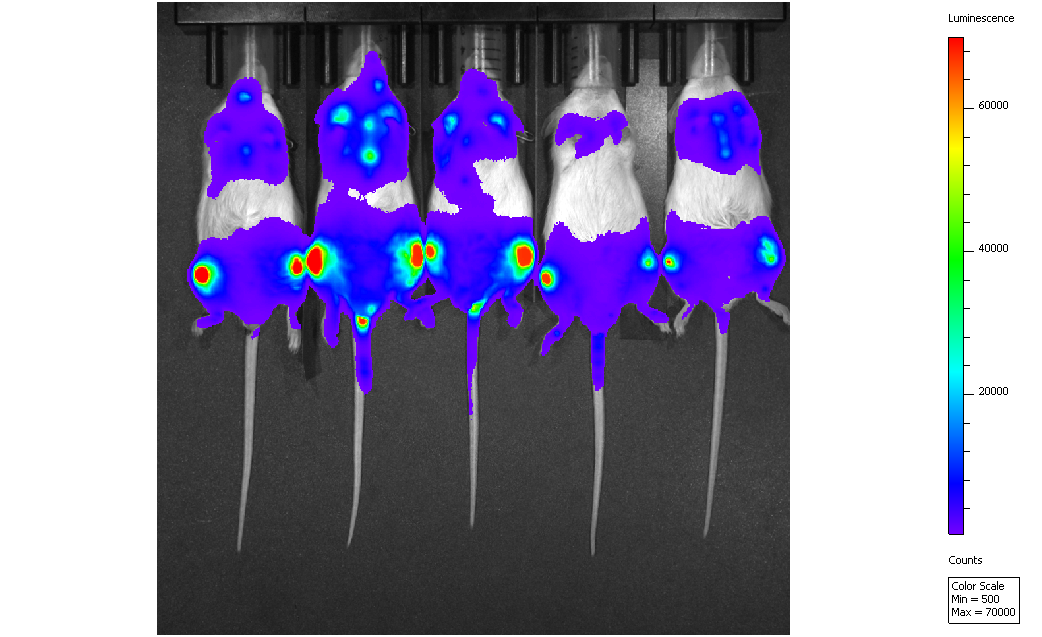

Supplement: Supplementary file 10 — Source data Fig. 4 [file 44321_2026_455_MOESM10_ESM.zip › Figure4/Panel F/DAY 11/6- mock2- 68 72 75 70 69.tif]

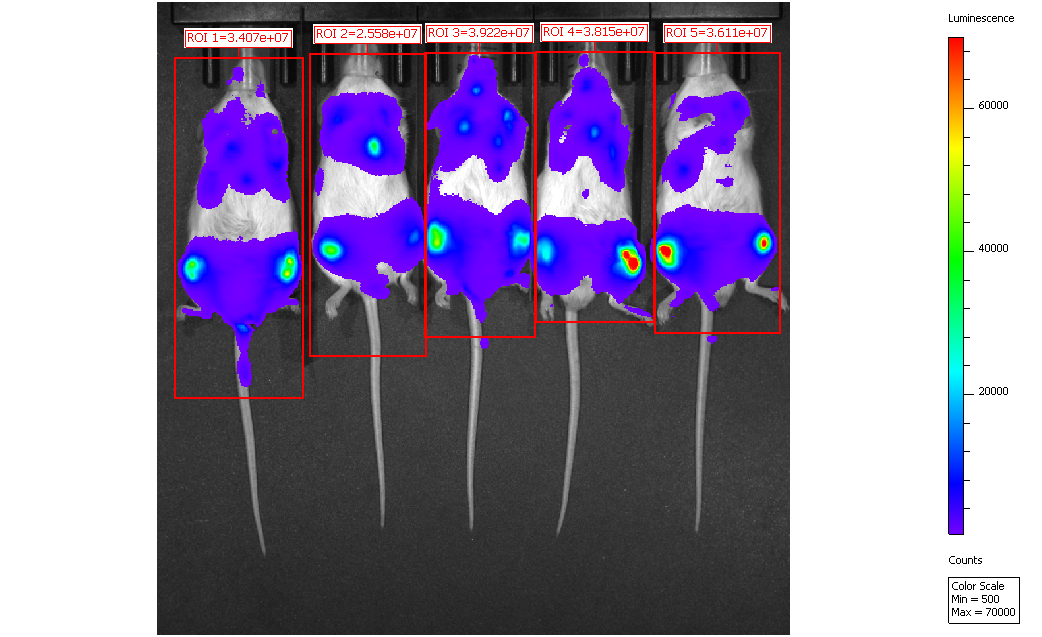

Supplement: Supplementary file 10 — Source data Fig. 4 [file 44321_2026_455_MOESM10_ESM.zip › Figure4/Panel F/DAY 11/7- 60 61 58 63 66 luc.tif]

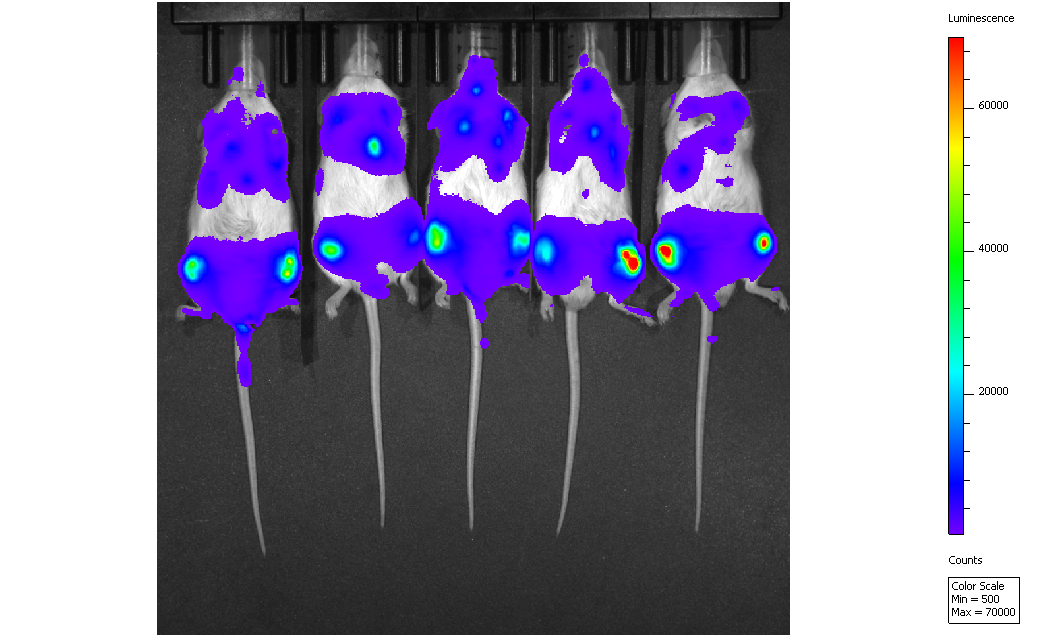

Supplement: Supplementary file 10 — Source data Fig. 4 [file 44321_2026_455_MOESM10_ESM.zip › Figure4/Panel F/DAY 11/7- 60 61 58 63 66.tif]

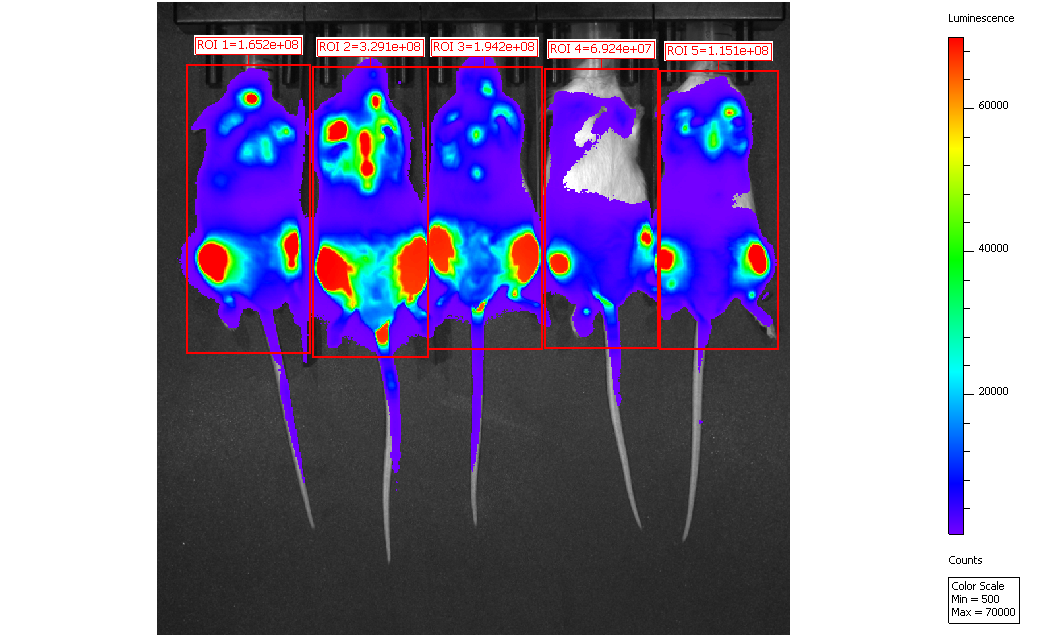

Supplement: Supplementary file 10 — Source data Fig. 4 [file 44321_2026_455_MOESM10_ESM.zip › Figure4/Panel F/DAY 14/5-mock-68 62 75 70 69 luc.tif]

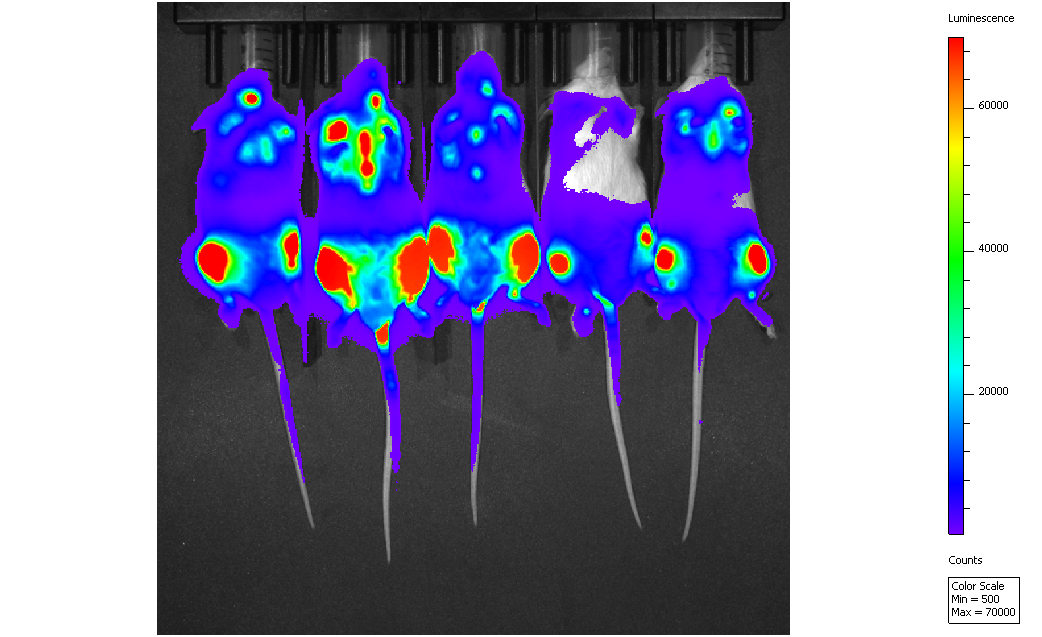

Supplement: Supplementary file 10 — Source data Fig. 4 [file 44321_2026_455_MOESM10_ESM.zip › Figure4/Panel F/DAY 14/5-mock-68 62 75 70 69.tif]

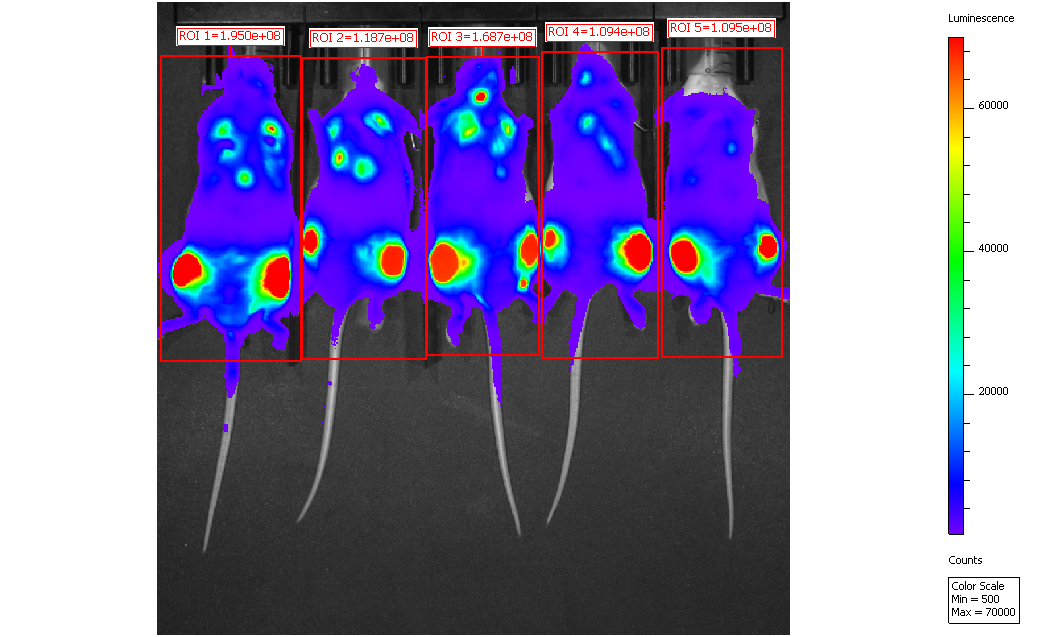

Supplement: Supplementary file 10 — Source data Fig. 4 [file 44321_2026_455_MOESM10_ESM.zip › Figure4/Panel F/DAY 14/7- 60 61 58 63 66 luc.tif]

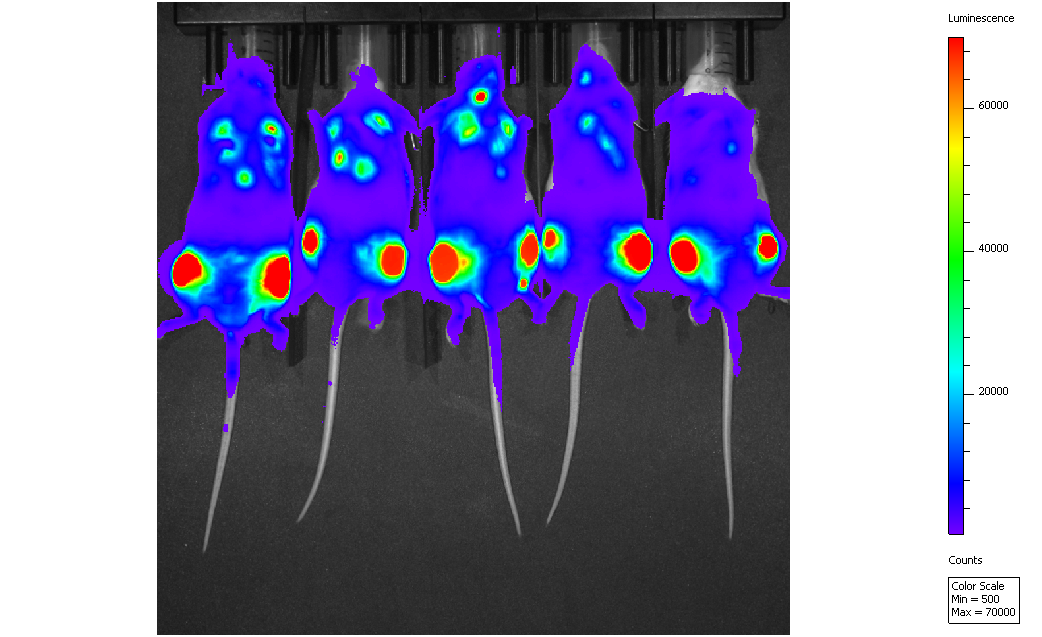

Supplement: Supplementary file 10 — Source data Fig. 4 [file 44321_2026_455_MOESM10_ESM.zip › Figure4/Panel F/DAY 14/7- 60 61 58 63 66.tif]

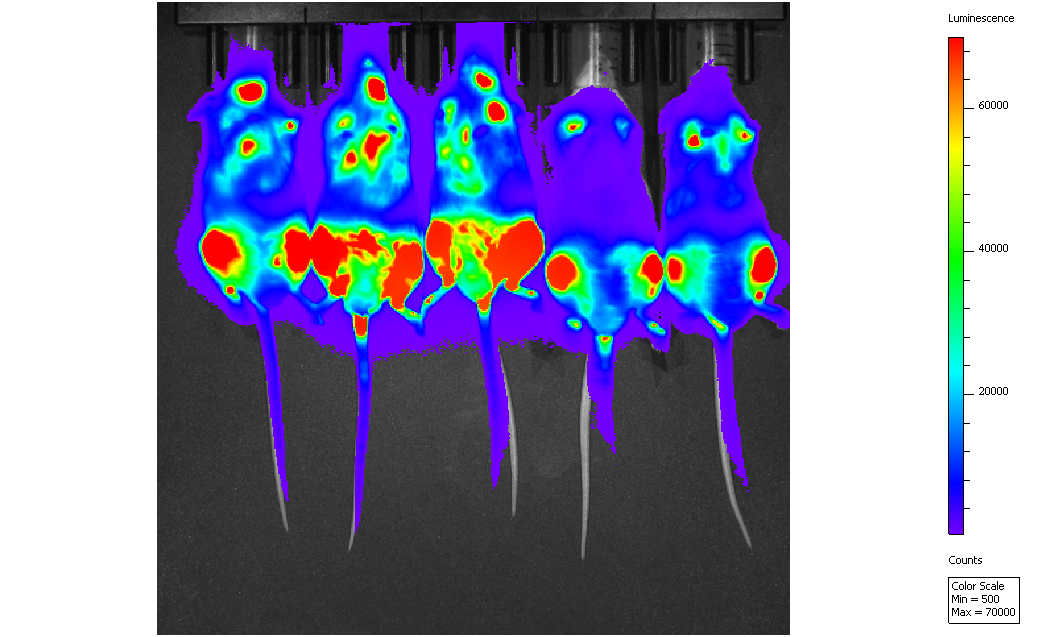

Supplement: Supplementary file 10 — Source data Fig. 4 [file 44321_2026_455_MOESM10_ESM.zip › Figure4/Panel F/DAY 17/5- 68 62 75 70 69.tif]

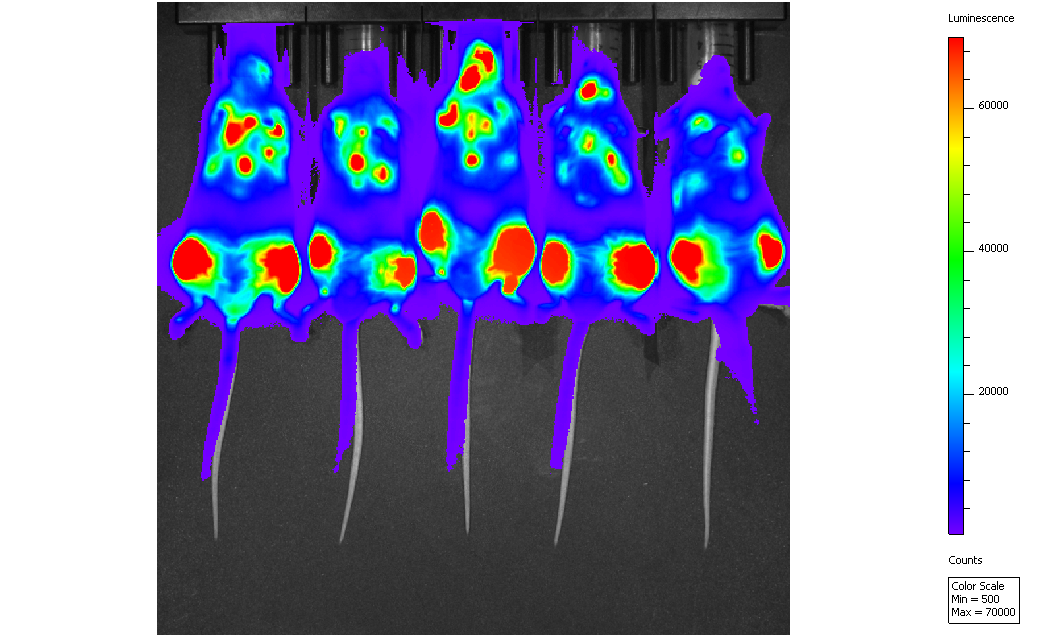

Supplement: Supplementary file 10 — Source data Fig. 4 [file 44321_2026_455_MOESM10_ESM.zip › Figure4/Panel F/DAY 17/7- 60 61 58 63 66.tif]

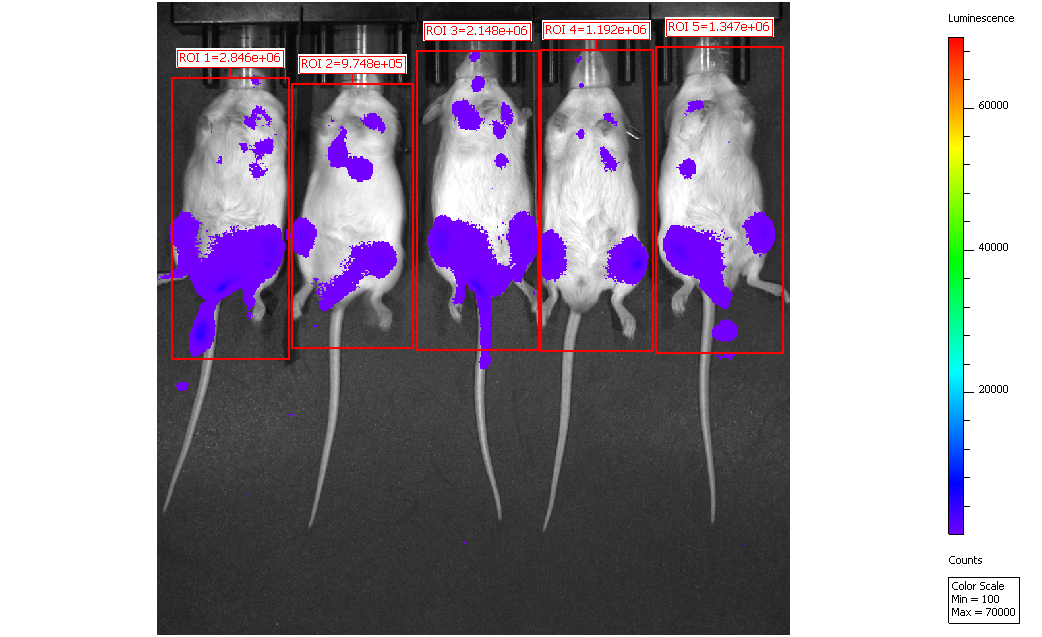

Supplement: Supplementary file 10 — Source data Fig. 4 [file 44321_2026_455_MOESM10_ESM.zip › Figure4/Panel F/DAY 7/7- 60 61 58 63 66 -1 luc.tif]

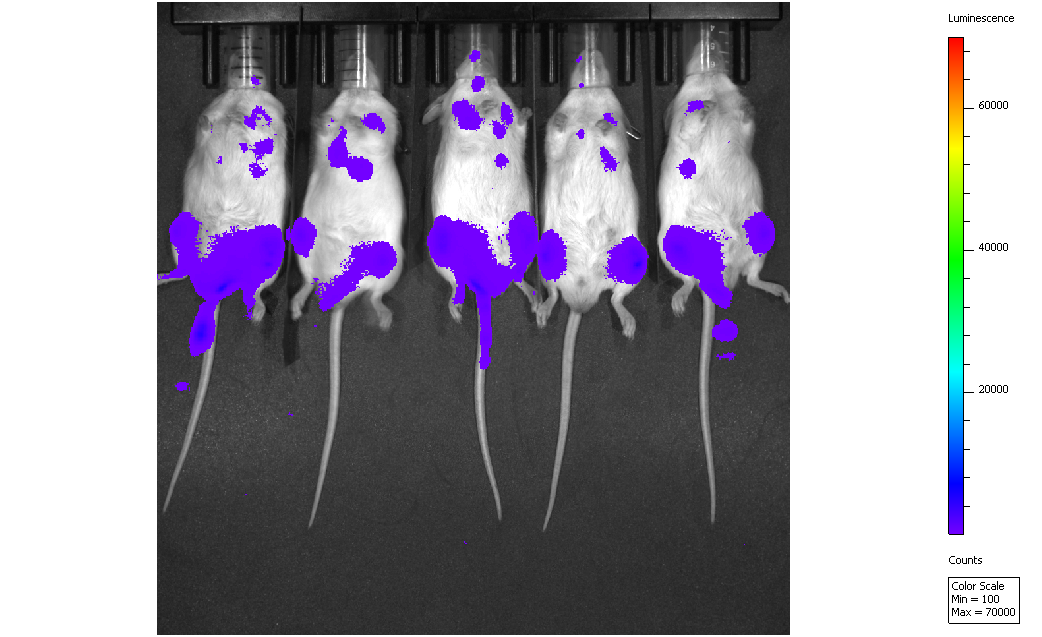

Supplement: Supplementary file 10 — Source data Fig. 4 [file 44321_2026_455_MOESM10_ESM.zip › Figure4/Panel F/DAY 7/7- 60 61 58 63 66 -1.tif]

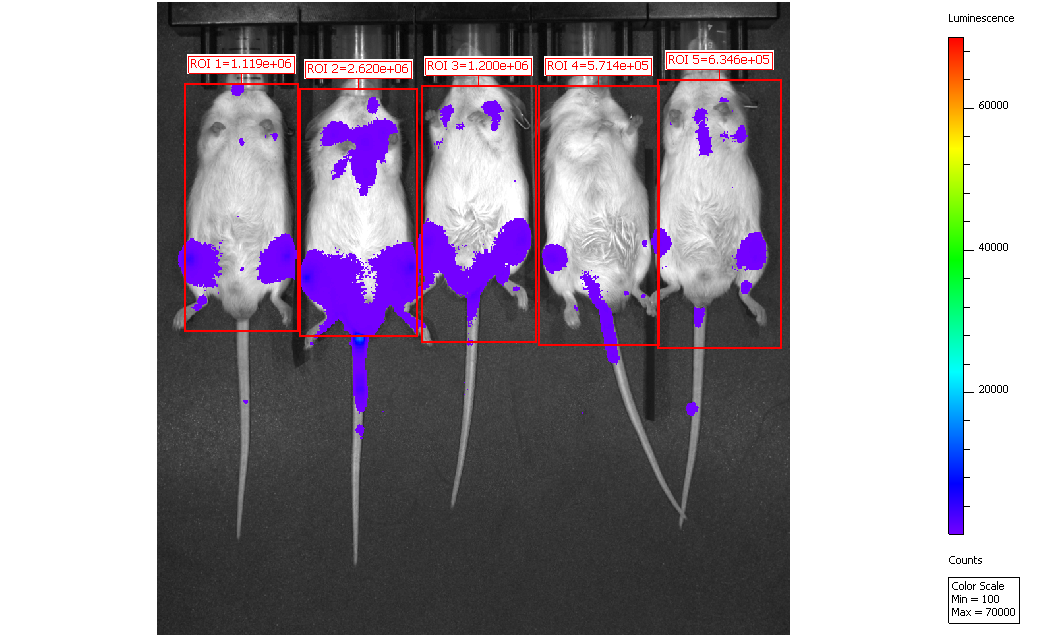

Supplement: Supplementary file 10 — Source data Fig. 4 [file 44321_2026_455_MOESM10_ESM.zip › Figure4/Panel F/DAY 7/MOCK-2- 68 62 75 70 69 luc.tif]

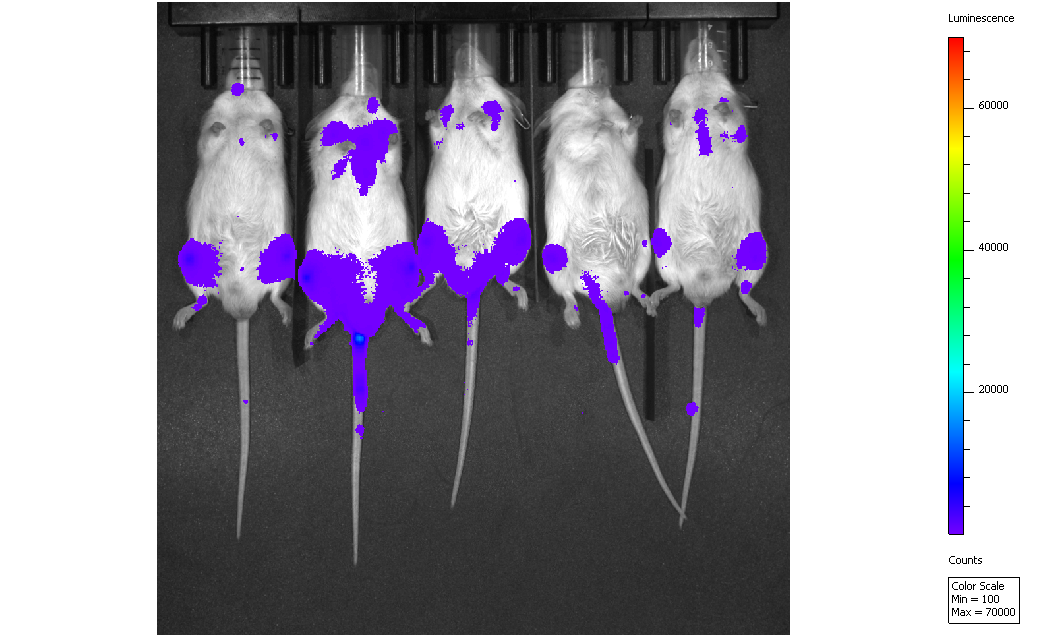

Supplement: Supplementary file 10 — Source data Fig. 4 [file 44321_2026_455_MOESM10_ESM.zip › Figure4/Panel F/DAY 7/MOCK-2- 68 62 75 70 69.tif]

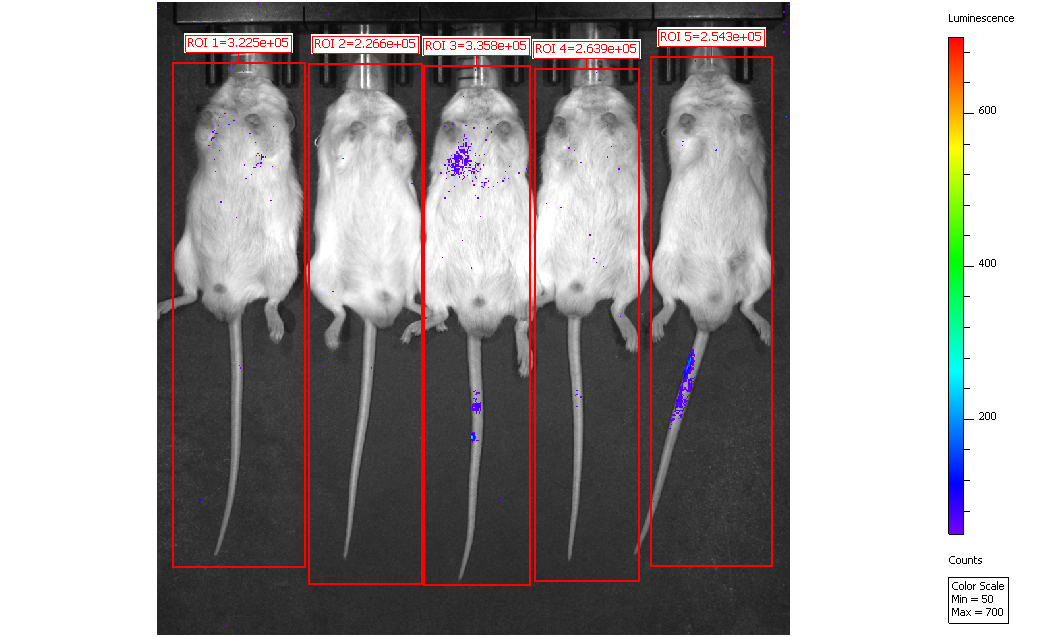

Supplement: Supplementary file 13 — Source data Fig. 7 [file 44321_2026_455_MOESM13_ESM.zip › Figure7/Panel B/DAY 0/1- 51 52 53 54 55 luc.tif]

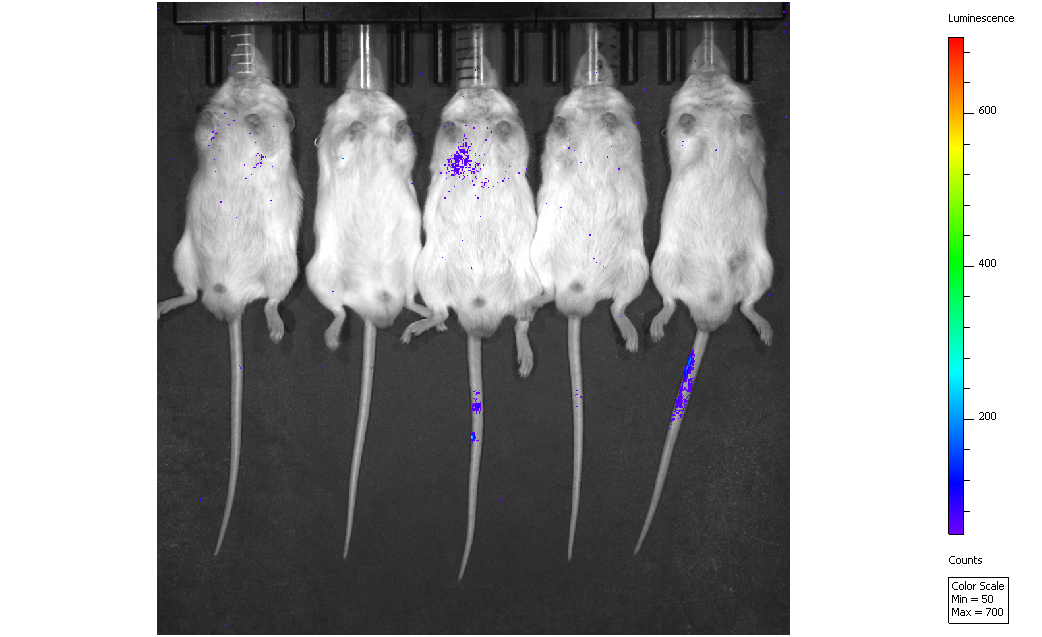

Supplement: Supplementary file 13 — Source data Fig. 7 [file 44321_2026_455_MOESM13_ESM.zip › Figure7/Panel B/DAY 0/1- 51 52 53 54 55.tif]

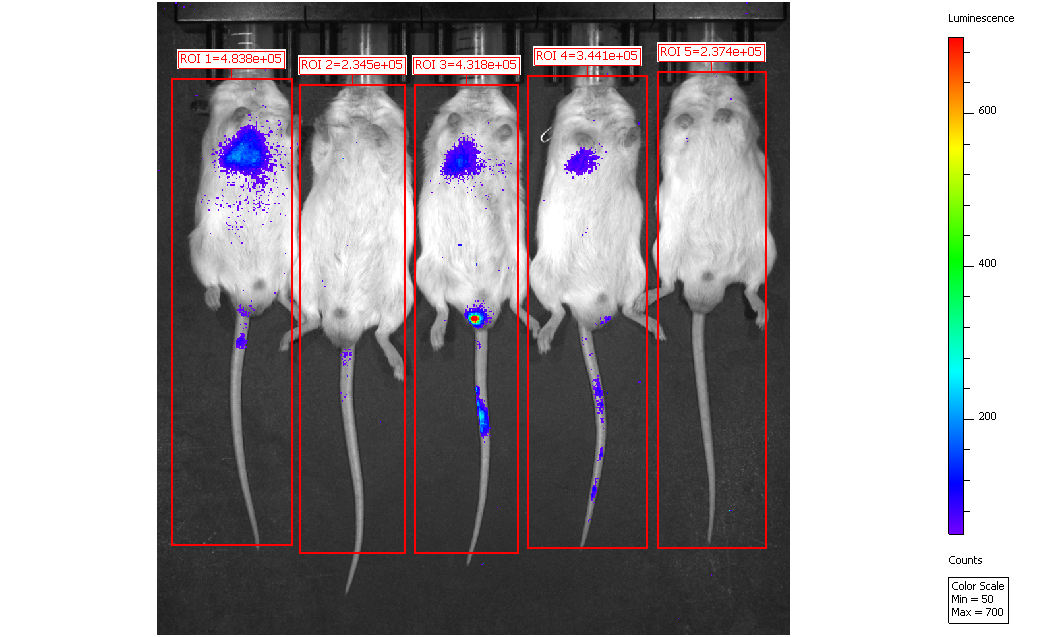

Supplement: Supplementary file 13 — Source data Fig. 7 [file 44321_2026_455_MOESM13_ESM.zip › Figure7/Panel B/DAY 0/2- 56 57 58 59 60 luc.tif]

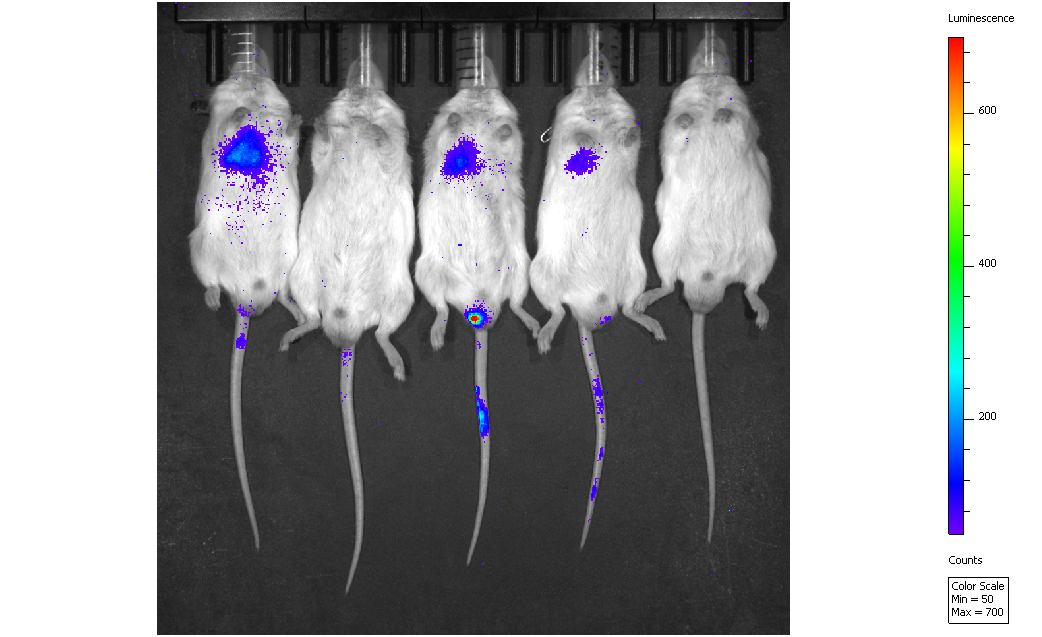

Supplement: Supplementary file 13 — Source data Fig. 7 [file 44321_2026_455_MOESM13_ESM.zip › Figure7/Panel B/DAY 0/2- 56 57 58 59 60.tif]

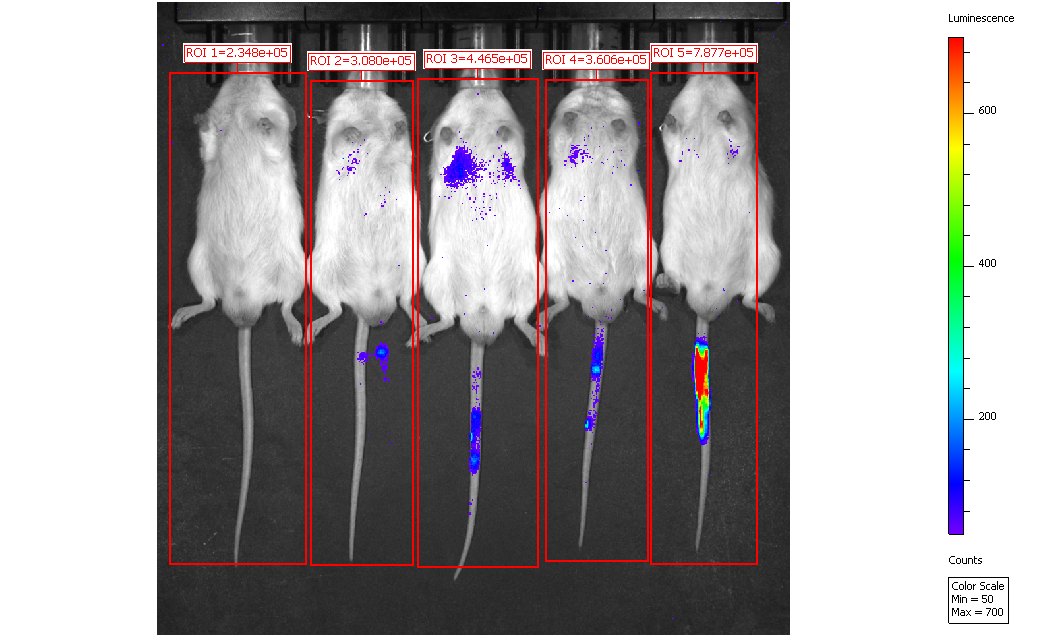

Supplement: Supplementary file 13 — Source data Fig. 7 [file 44321_2026_455_MOESM13_ESM.zip › Figure7/Panel B/DAY 0/3- 61 62 63 64 65 luc.tif]

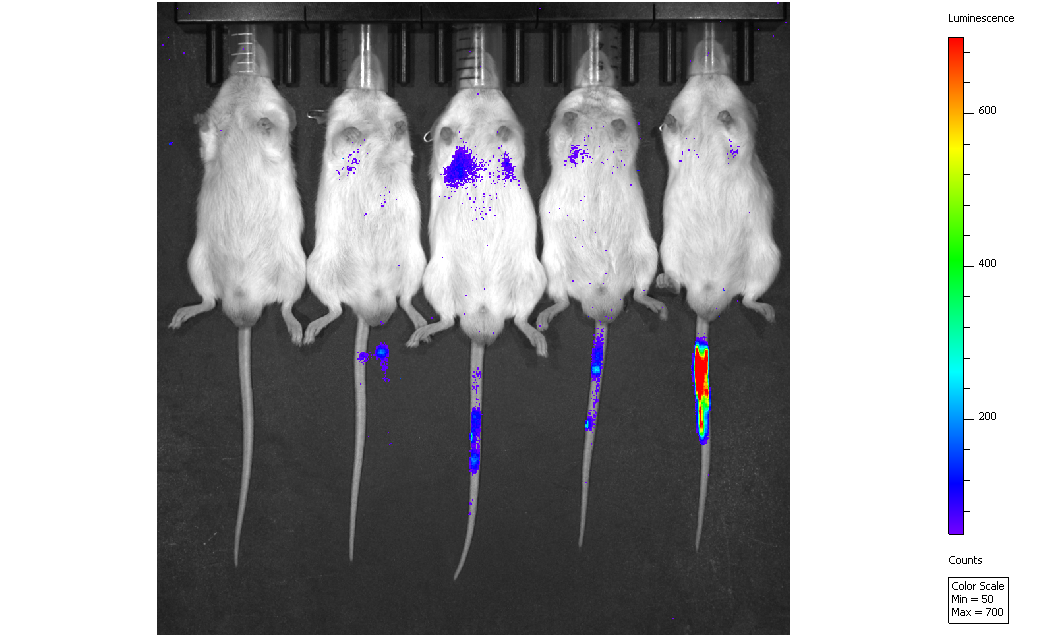

Supplement: Supplementary file 13 — Source data Fig. 7 [file 44321_2026_455_MOESM13_ESM.zip › Figure7/Panel B/DAY 0/3- 61 62 63 64 65.tif]

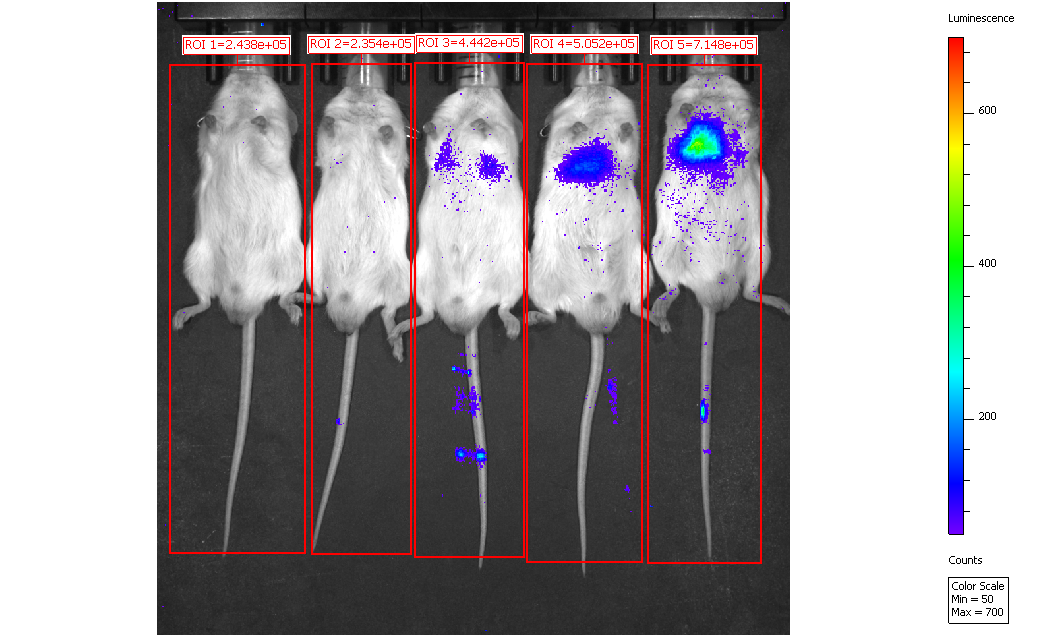

Supplement: Supplementary file 13 — Source data Fig. 7 [file 44321_2026_455_MOESM13_ESM.zip › Figure7/Panel B/DAY 0/4- 66 67 68 69 70 luc.tif]

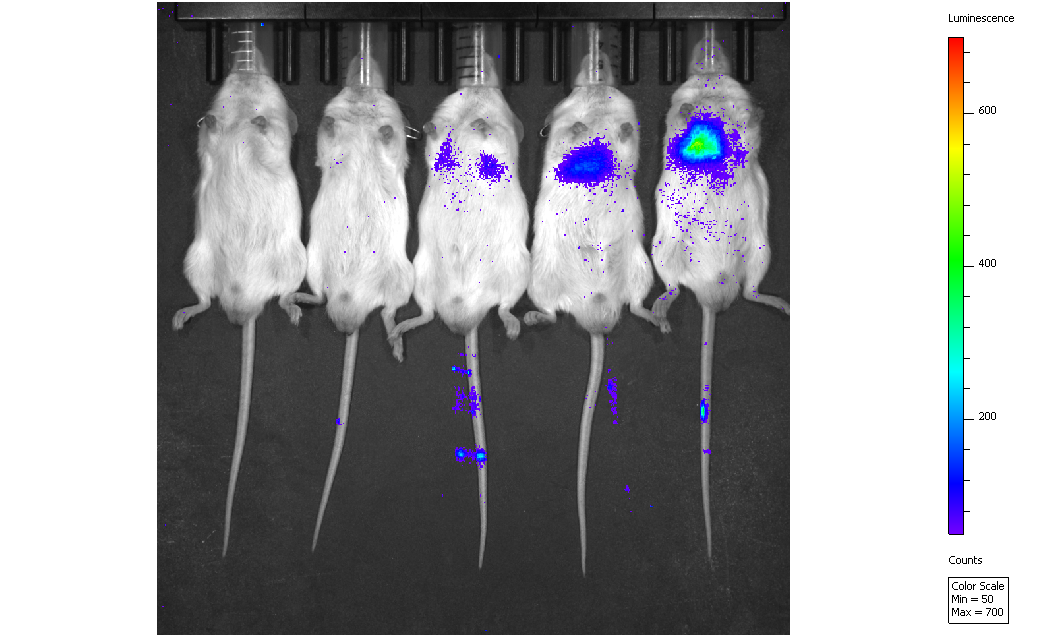

Supplement: Supplementary file 13 — Source data Fig. 7 [file 44321_2026_455_MOESM13_ESM.zip › Figure7/Panel B/DAY 0/4- 66 67 68 69 70.tif]

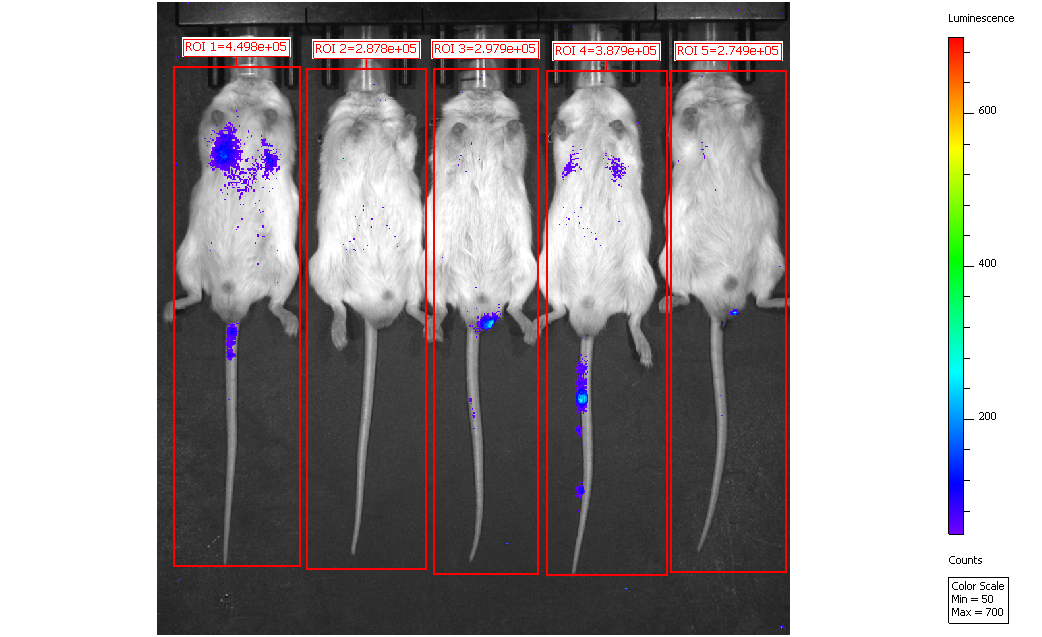

Supplement: Supplementary file 13 — Source data Fig. 7 [file 44321_2026_455_MOESM13_ESM.zip › Figure7/Panel B/DAY 0/5- 71 72 73 74 75 luc.tif]

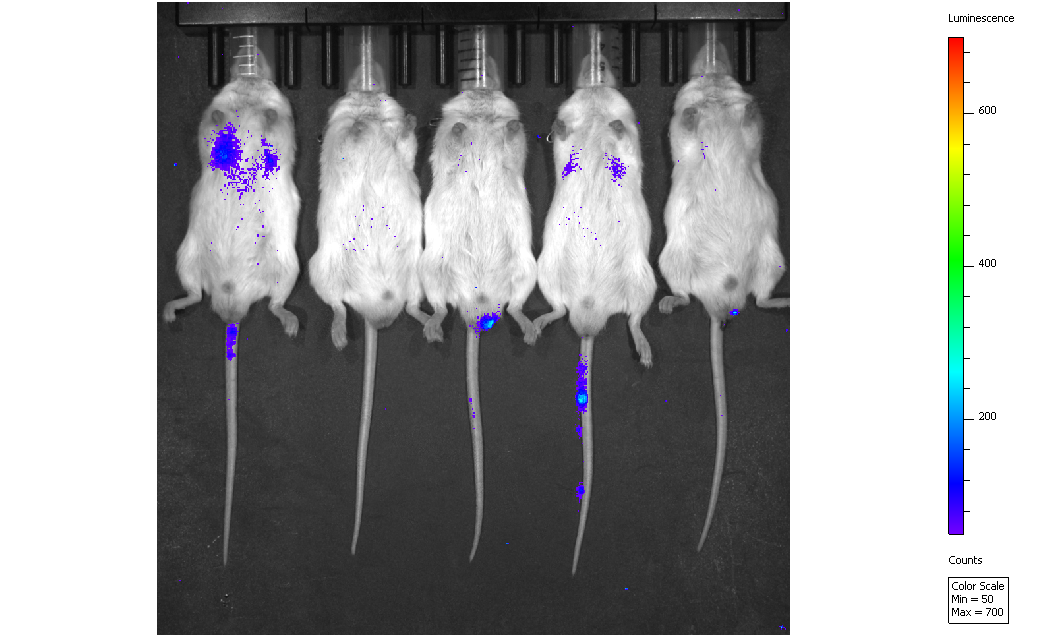

Supplement: Supplementary file 13 — Source data Fig. 7 [file 44321_2026_455_MOESM13_ESM.zip › Figure7/Panel B/DAY 0/5- 71 72 73 74 75.tif]

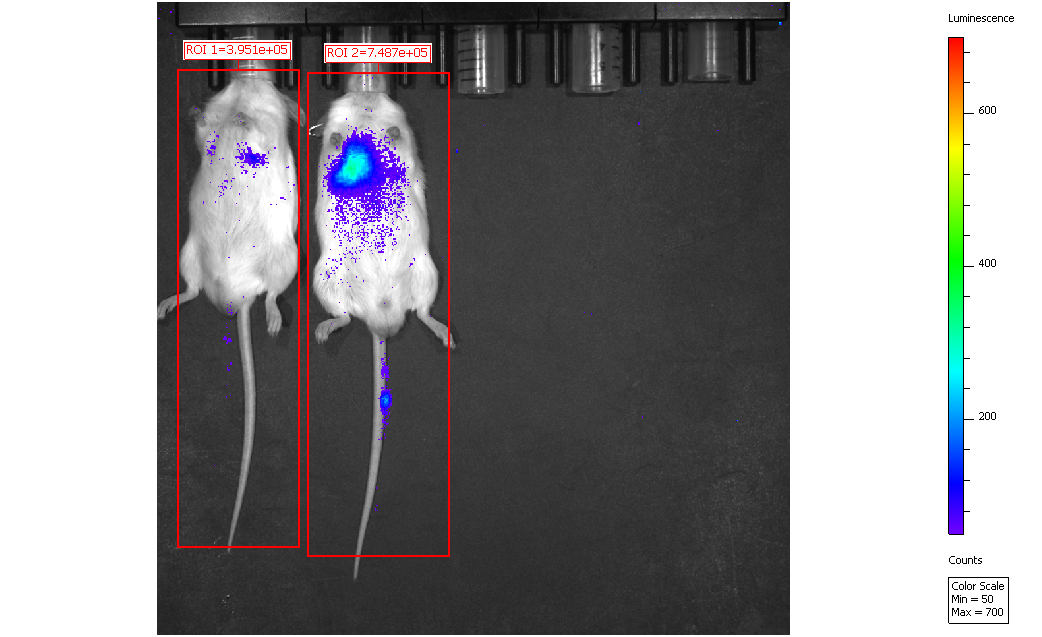

Supplement: Supplementary file 13 — Source data Fig. 7 [file 44321_2026_455_MOESM13_ESM.zip › Figure7/Panel B/DAY 0/6- 76 77 luc.tif]

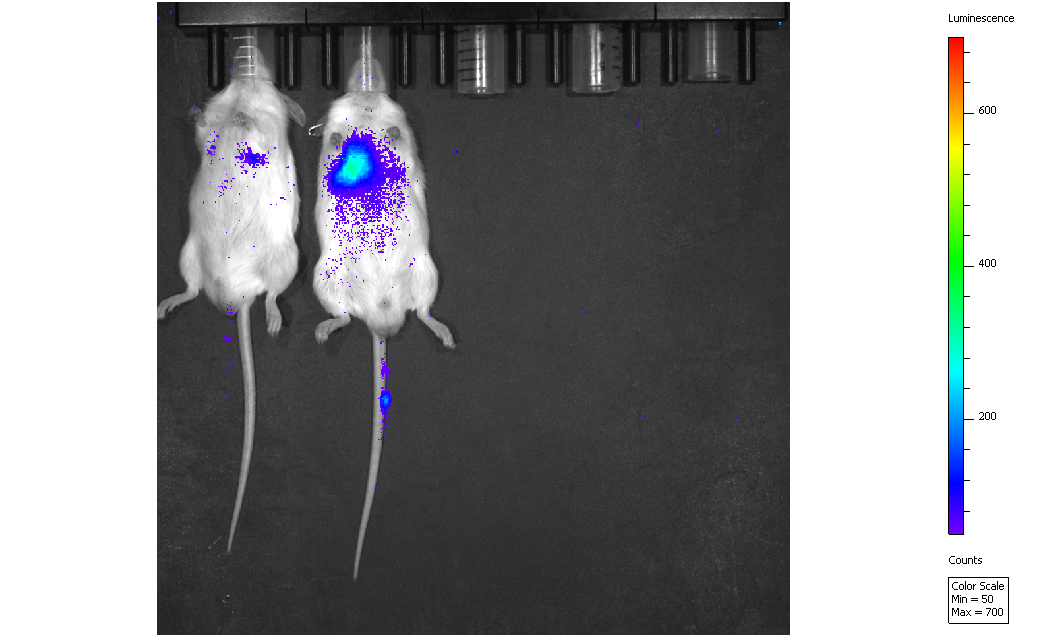

Supplement: Supplementary file 13 — Source data Fig. 7 [file 44321_2026_455_MOESM13_ESM.zip › Figure7/Panel B/DAY 0/6- 76 77.tif]

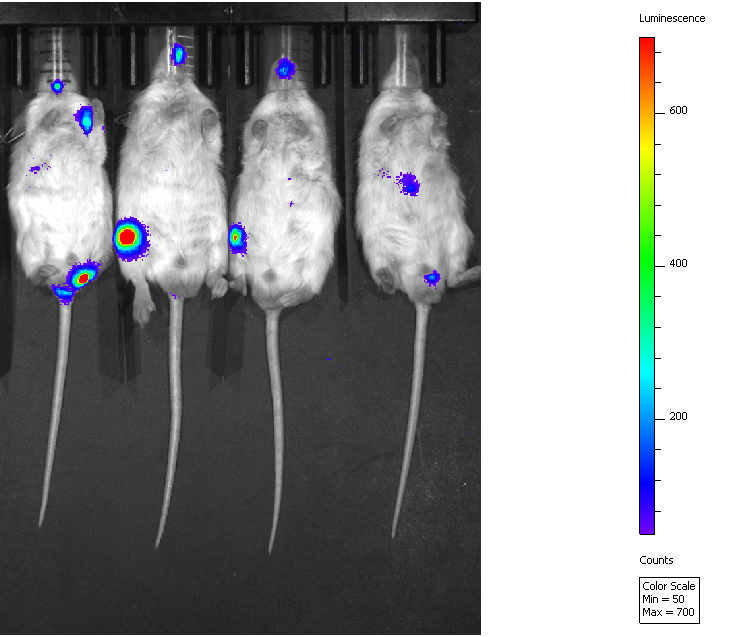

Supplement: Supplementary file 13 — Source data Fig. 7 [file 44321_2026_455_MOESM13_ESM.zip › Figure7/Panel B/DAY 22/1- 59 53 63 67.tif]

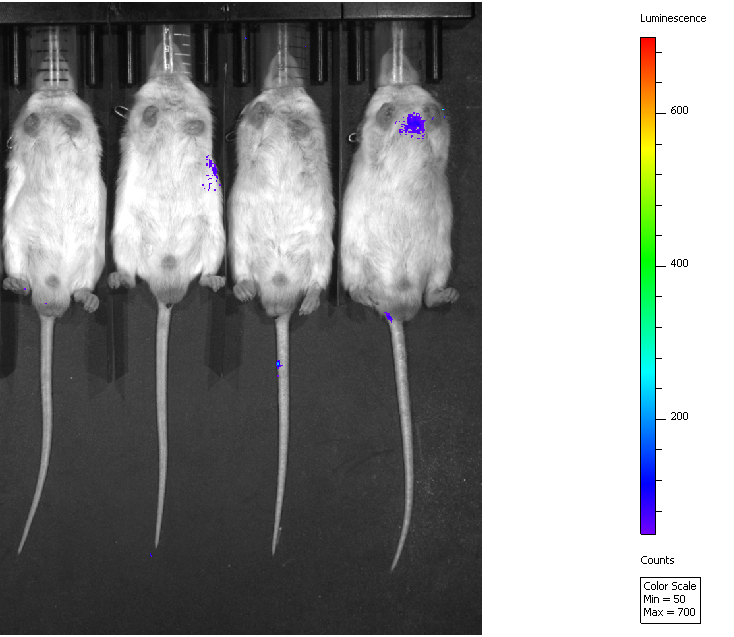

Supplement: Supplementary file 13 — Source data Fig. 7 [file 44321_2026_455_MOESM13_ESM.zip › Figure7/Panel B/DAY 22/3-74 62 55 57.tif]

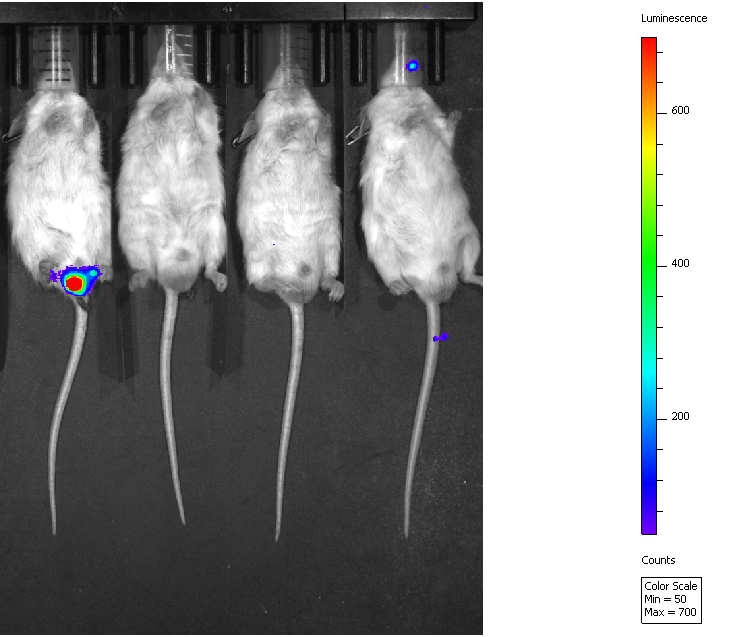

Supplement: Supplementary file 13 — Source data Fig. 7 [file 44321_2026_455_MOESM13_ESM.zip › Figure7/Panel B/DAY 22/5- 76 73 54 52.tif]

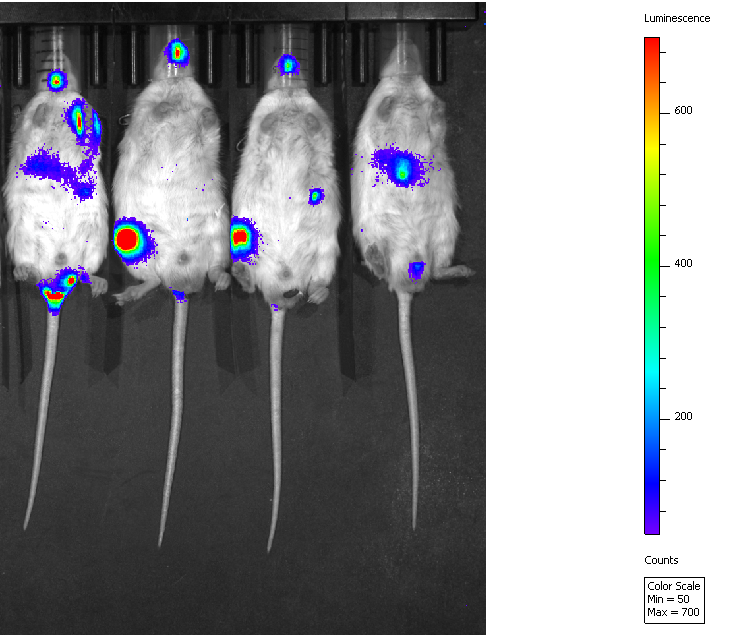

Supplement: Supplementary file 13 — Source data Fig. 7 [file 44321_2026_455_MOESM13_ESM.zip › Figure7/Panel B/DAY 25/1- 59 53 63 67.tif]

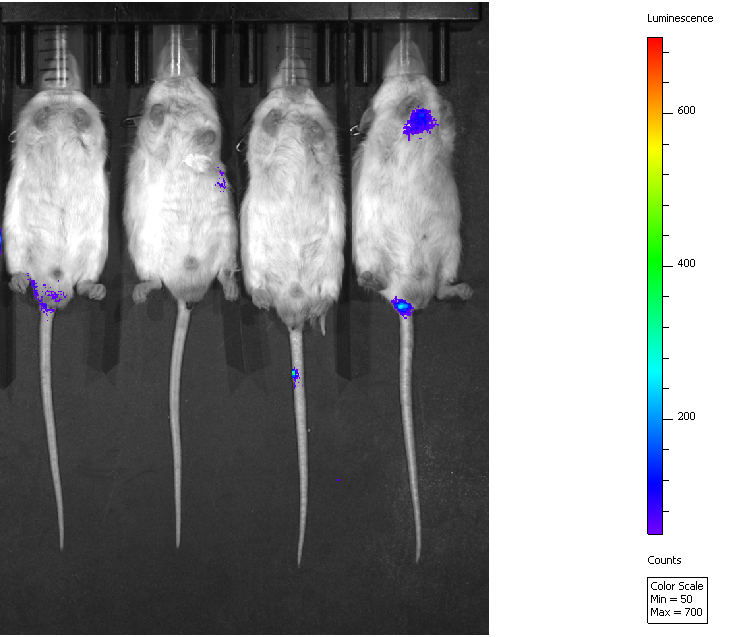

Supplement: Supplementary file 13 — Source data Fig. 7 [file 44321_2026_455_MOESM13_ESM.zip › Figure7/Panel B/DAY 25/3- 74 62 55 57.tif]

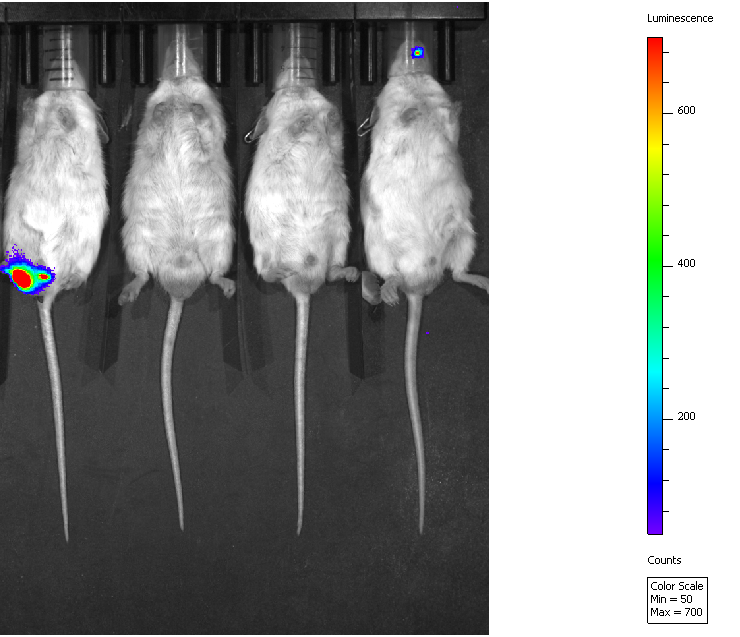

Supplement: Supplementary file 13 — Source data Fig. 7 [file 44321_2026_455_MOESM13_ESM.zip › Figure7/Panel B/DAY 25/5- 58 72 75 65.tif]

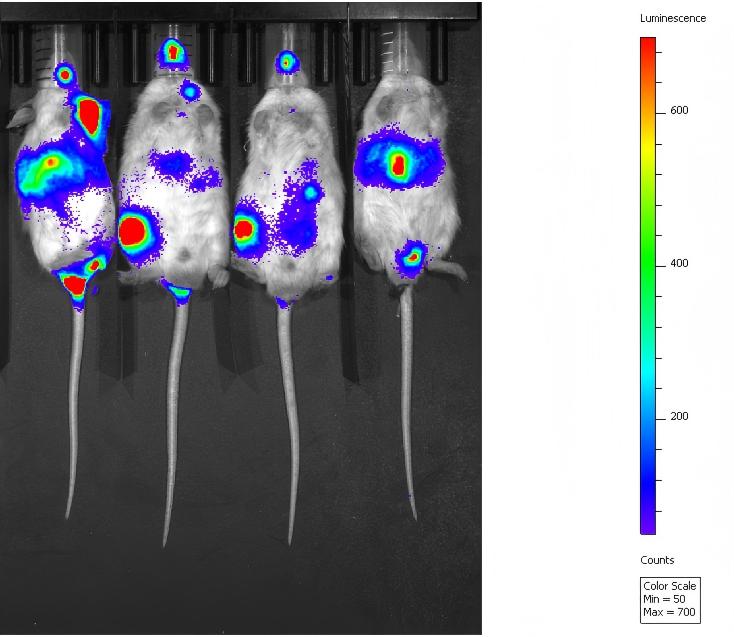

Supplement: Supplementary file 13 — Source data Fig. 7 [file 44321_2026_455_MOESM13_ESM.zip › Figure7/Panel B/DAY 29/1- 59 53 63 67.jpg]

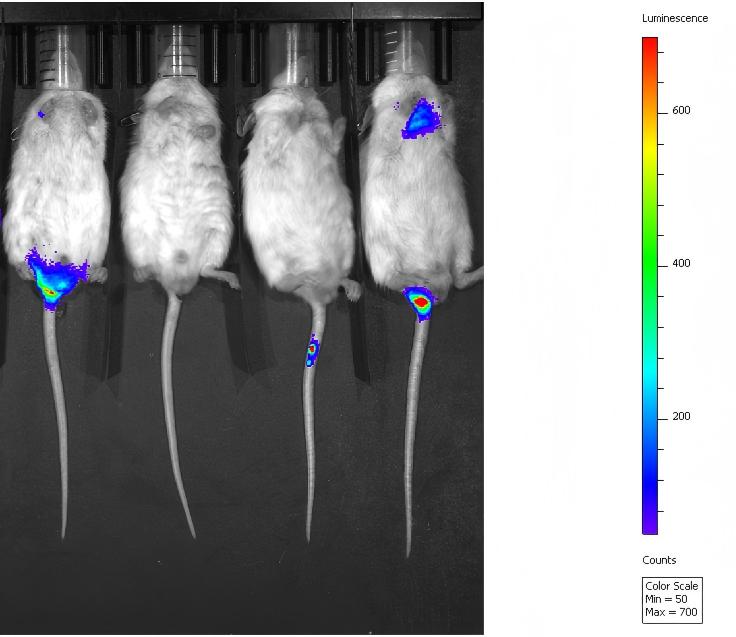

Supplement: Supplementary file 13 — Source data Fig. 7 [file 44321_2026_455_MOESM13_ESM.zip › Figure7/Panel B/DAY 29/3- 74 62 55 57.jpg]

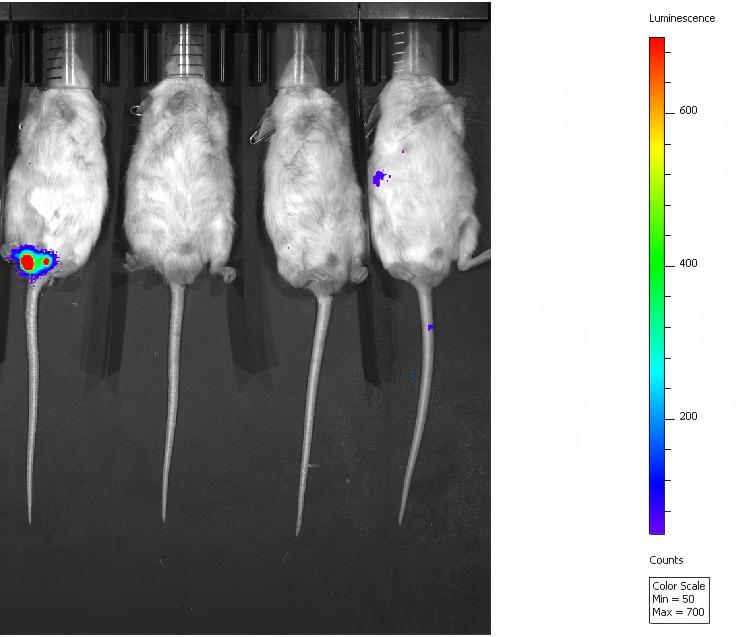

Supplement: Supplementary file 13 — Source data Fig. 7 [file 44321_2026_455_MOESM13_ESM.zip › Figure7/Panel B/DAY 29/5- 58 72 75 65.jpg]

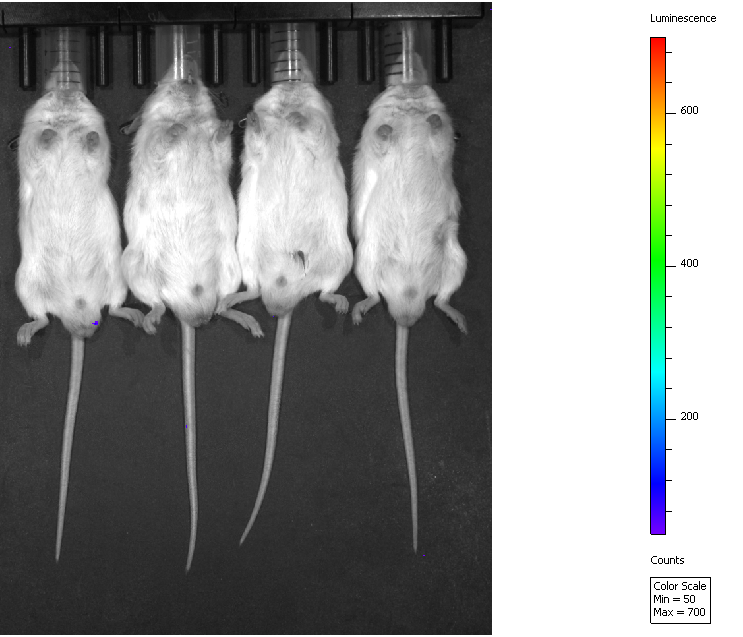

Supplement: Supplementary file 13 — Source data Fig. 7 [file 44321_2026_455_MOESM13_ESM.zip › Figure7/Panel B/DAY 3/1- 59 53 63 67.tif]

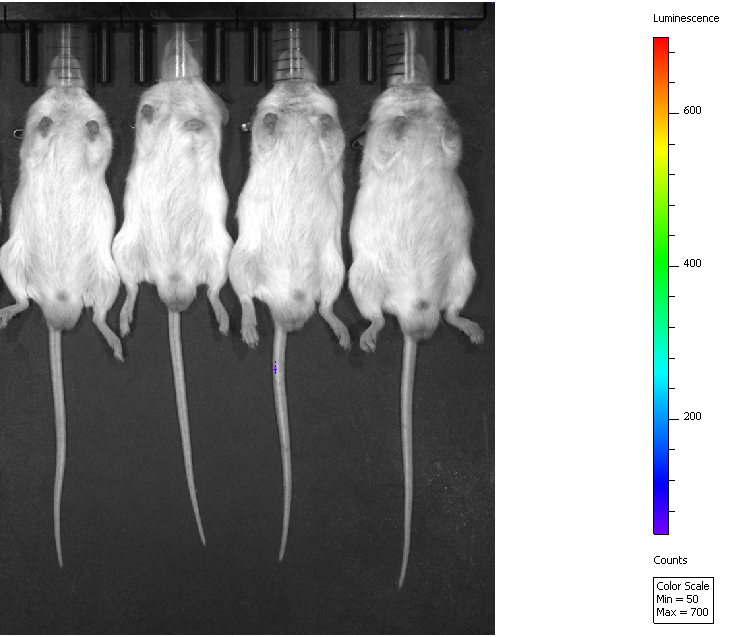

Supplement: Supplementary file 13 — Source data Fig. 7 [file 44321_2026_455_MOESM13_ESM.zip › Figure7/Panel B/DAY 3/3- 74 62 55 57.tif]

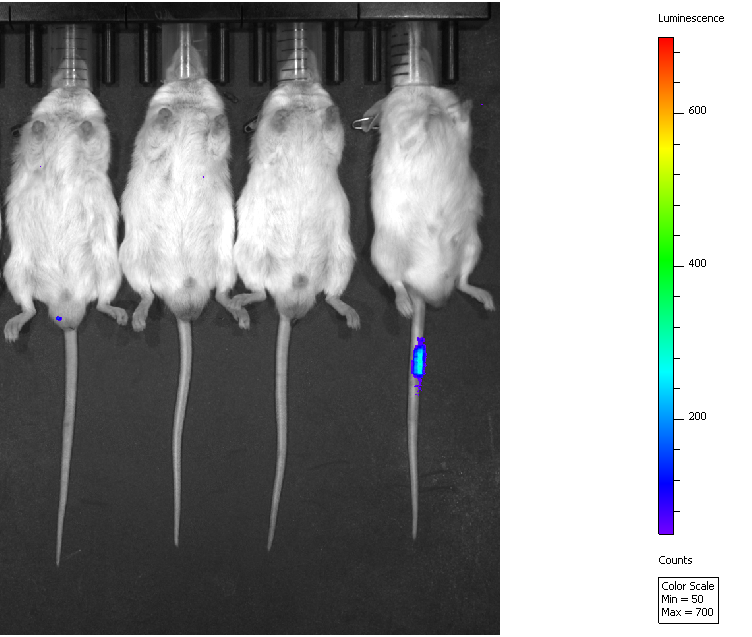

Supplement: Supplementary file 13 — Source data Fig. 7 [file 44321_2026_455_MOESM13_ESM.zip › Figure7/Panel B/DAY 3/5- 58 72 75 65.tif]

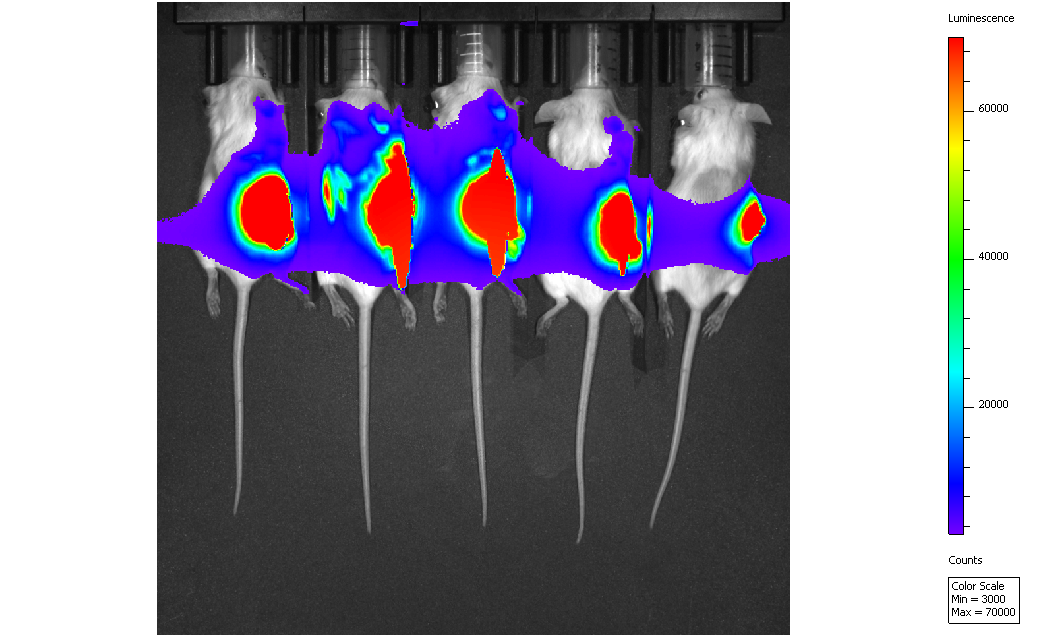

Supplement: Supplementary file 13 — Source data Fig. 7 [file 44321_2026_455_MOESM13_ESM.zip › Figure7/Panel E/DAY 17/1- 17 21 3 12 10-3000.tif]

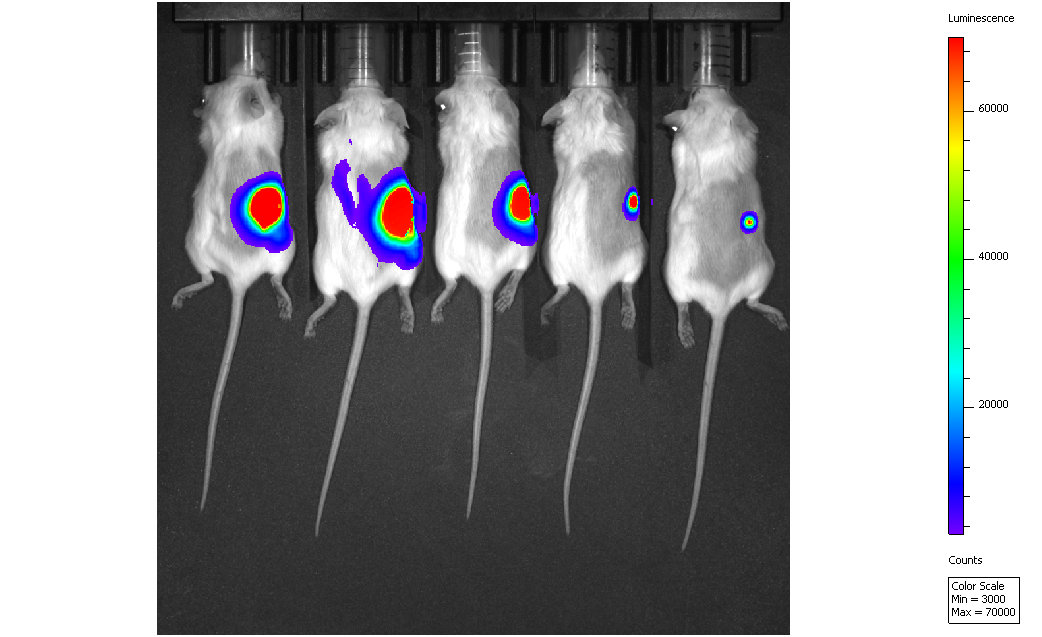

Supplement: Supplementary file 13 — Source data Fig. 7 [file 44321_2026_455_MOESM13_ESM.zip › Figure7/Panel E/DAY 17/2- 20 7 1 9 6 -3000.tif]

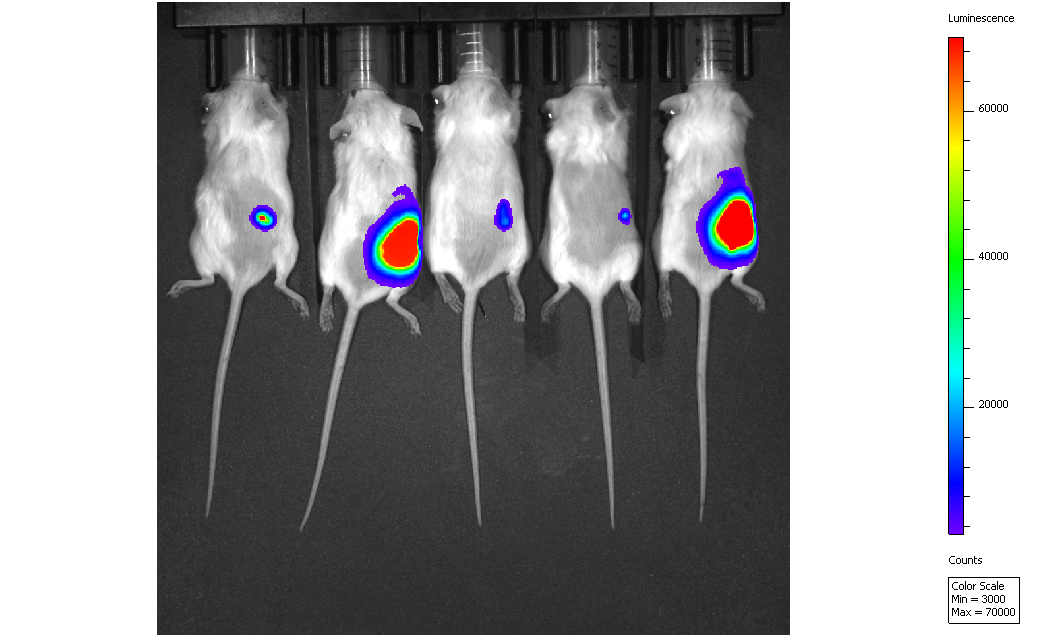

Supplement: Supplementary file 13 — Source data Fig. 7 [file 44321_2026_455_MOESM13_ESM.zip › Figure7/Panel E/DAY 17/5- 40 29 27 42 35.tif]

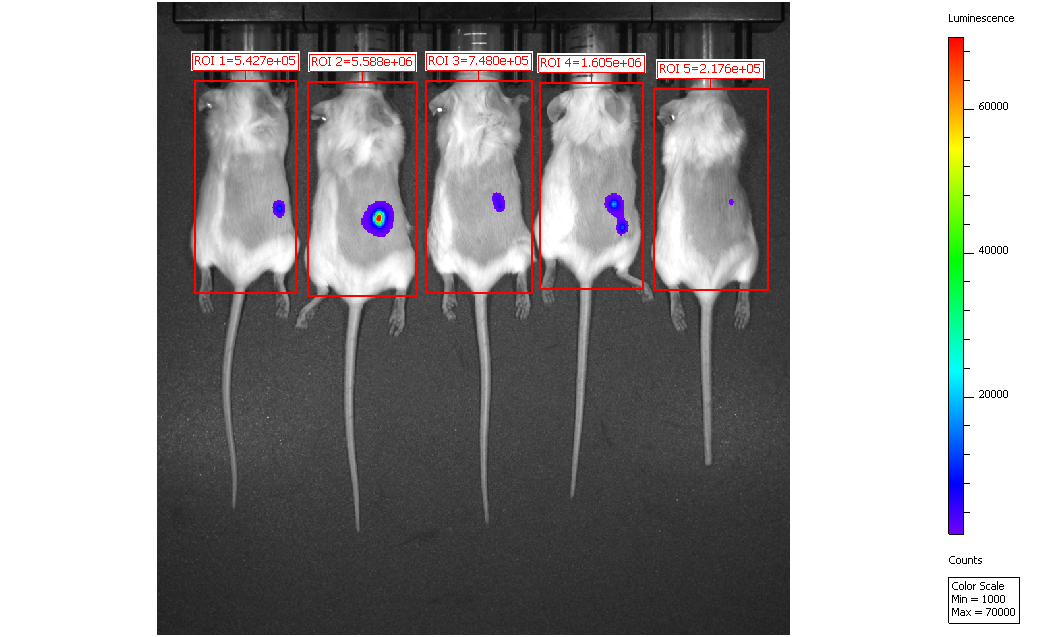

Supplement: Supplementary file 13 — Source data Fig. 7 [file 44321_2026_455_MOESM13_ESM.zip › Figure7/Panel E/DAY 3/1-5 luc.tif]

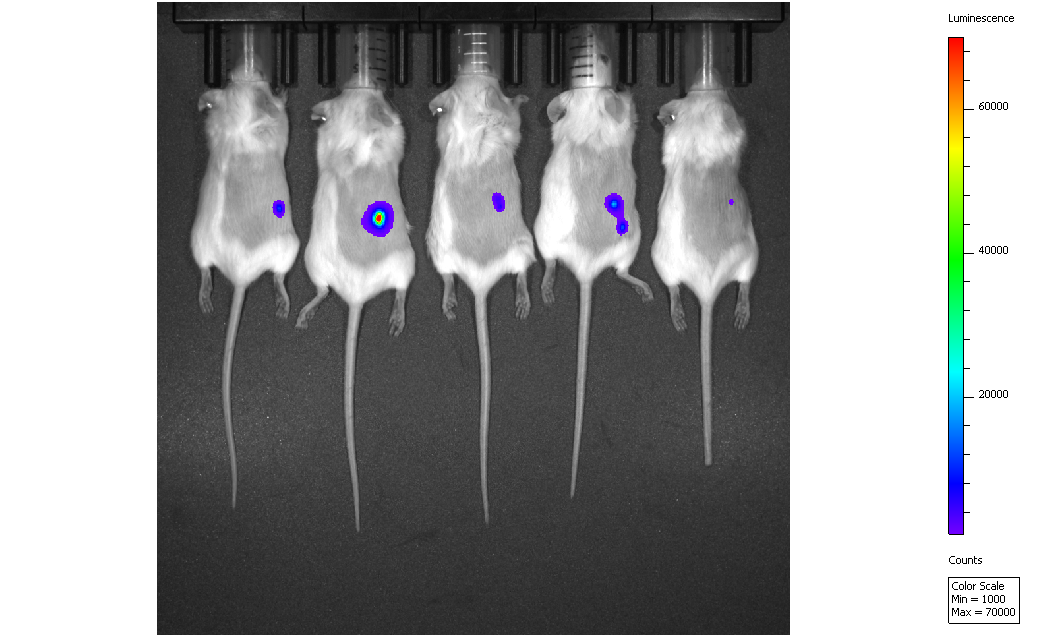

Supplement: Supplementary file 13 — Source data Fig. 7 [file 44321_2026_455_MOESM13_ESM.zip › Figure7/Panel E/DAY 3/1-5.tif]

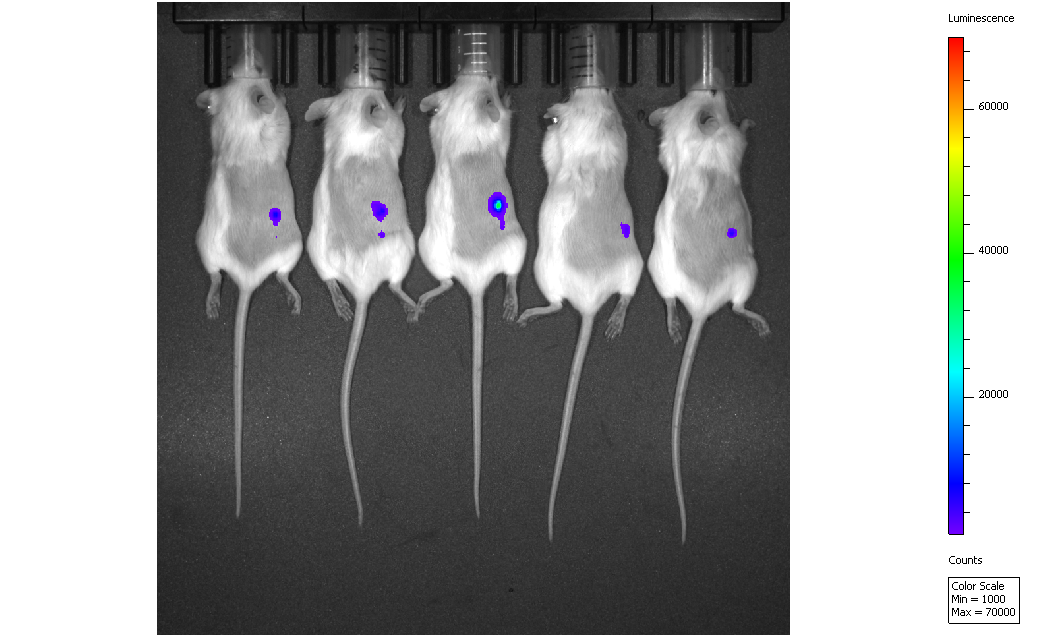

Supplement: Supplementary file 13 — Source data Fig. 7 [file 44321_2026_455_MOESM13_ESM.zip › Figure7/Panel E/DAY 3/11-15.tif]

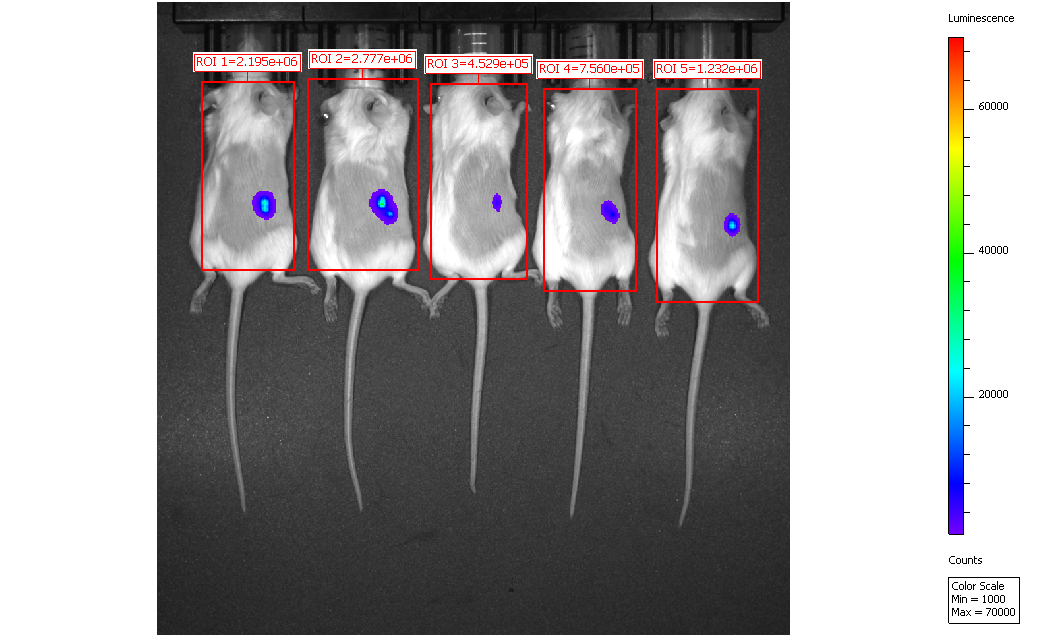

Supplement: Supplementary file 13 — Source data Fig. 7 [file 44321_2026_455_MOESM13_ESM.zip › Figure7/Panel E/DAY 3/16-20 luc.tif]

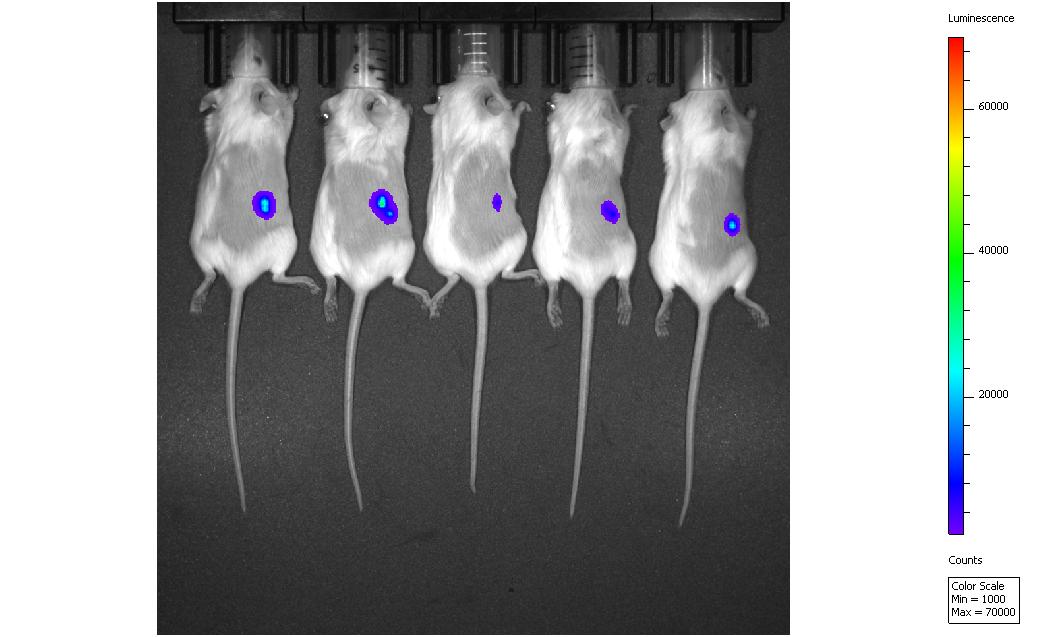

Supplement: Supplementary file 13 — Source data Fig. 7 [file 44321_2026_455_MOESM13_ESM.zip › Figure7/Panel E/DAY 3/16-20.tif]

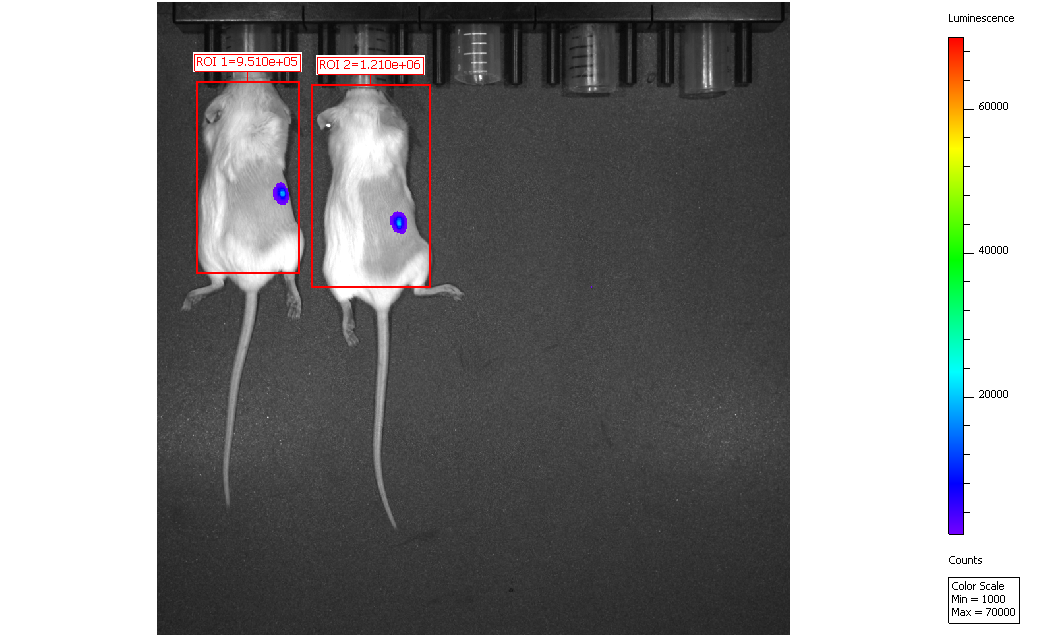

Supplement: Supplementary file 13 — Source data Fig. 7 [file 44321_2026_455_MOESM13_ESM.zip › Figure7/Panel E/DAY 3/21-22 luc.tif]

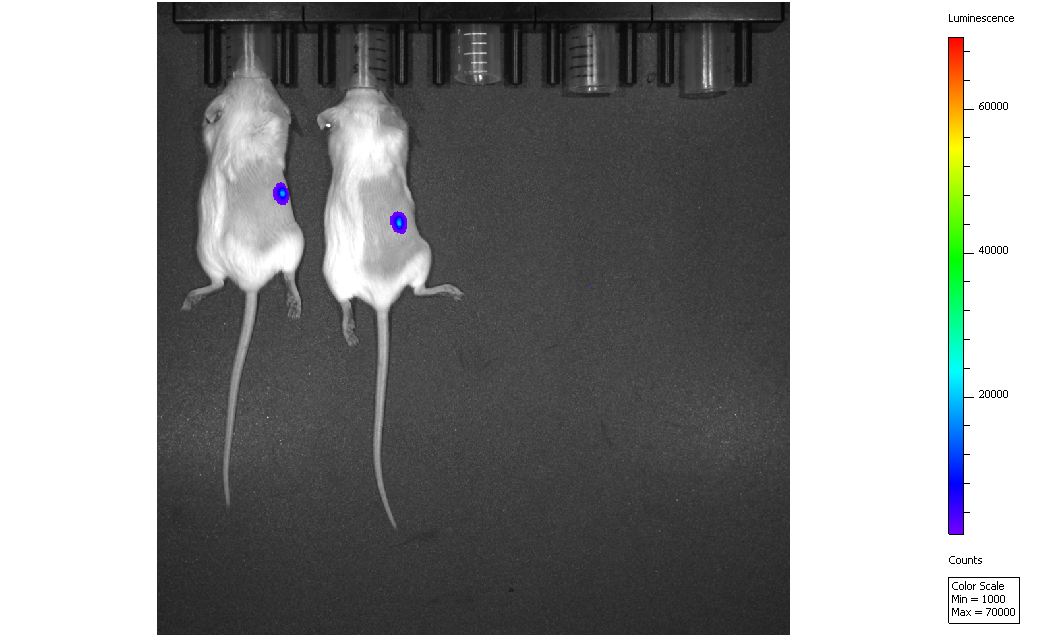

Supplement: Supplementary file 13 — Source data Fig. 7 [file 44321_2026_455_MOESM13_ESM.zip › Figure7/Panel E/DAY 3/21-22.tif]

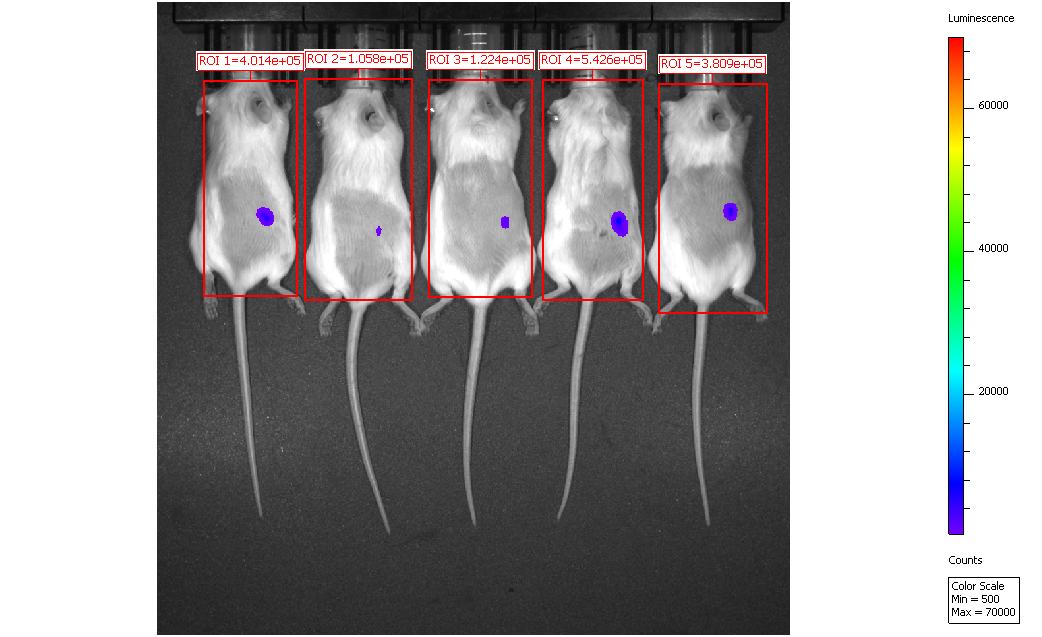

Supplement: Supplementary file 13 — Source data Fig. 7 [file 44321_2026_455_MOESM13_ESM.zip › Figure7/Panel E/DAY 3/26-30 luc.tif]

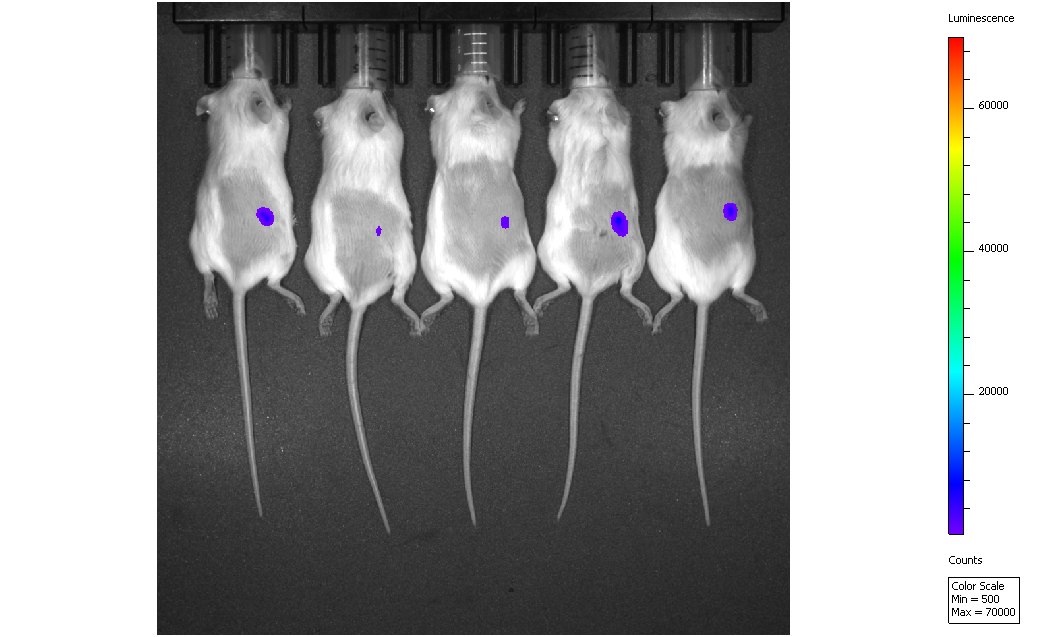

Supplement: Supplementary file 13 — Source data Fig. 7 [file 44321_2026_455_MOESM13_ESM.zip › Figure7/Panel E/DAY 3/26-30.tif]

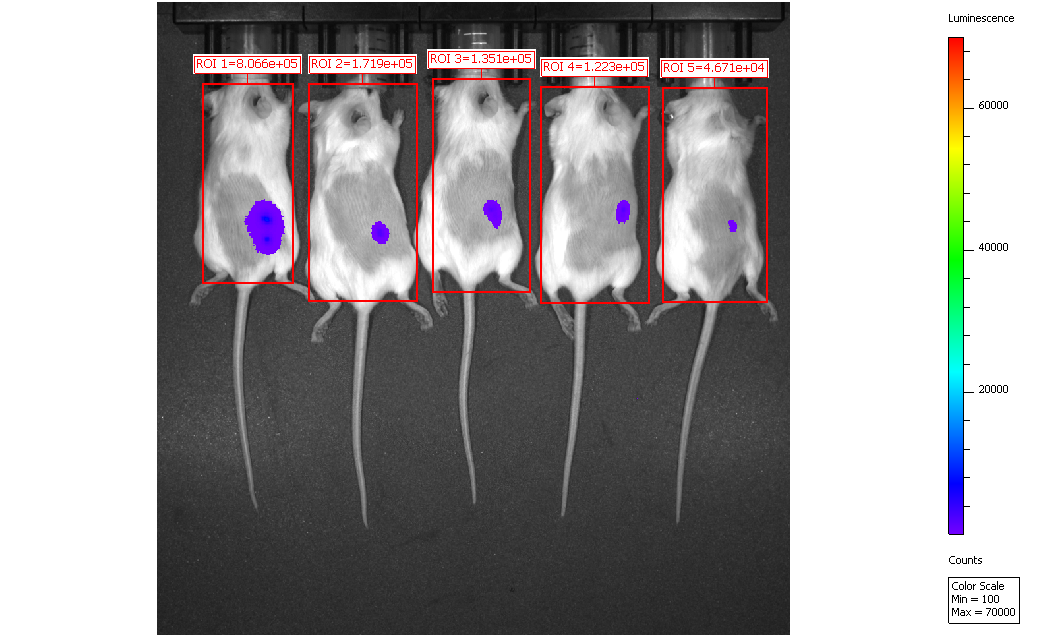

Supplement: Supplementary file 13 — Source data Fig. 7 [file 44321_2026_455_MOESM13_ESM.zip › Figure7/Panel E/DAY 3/31-35 luc 100--70000.tif]

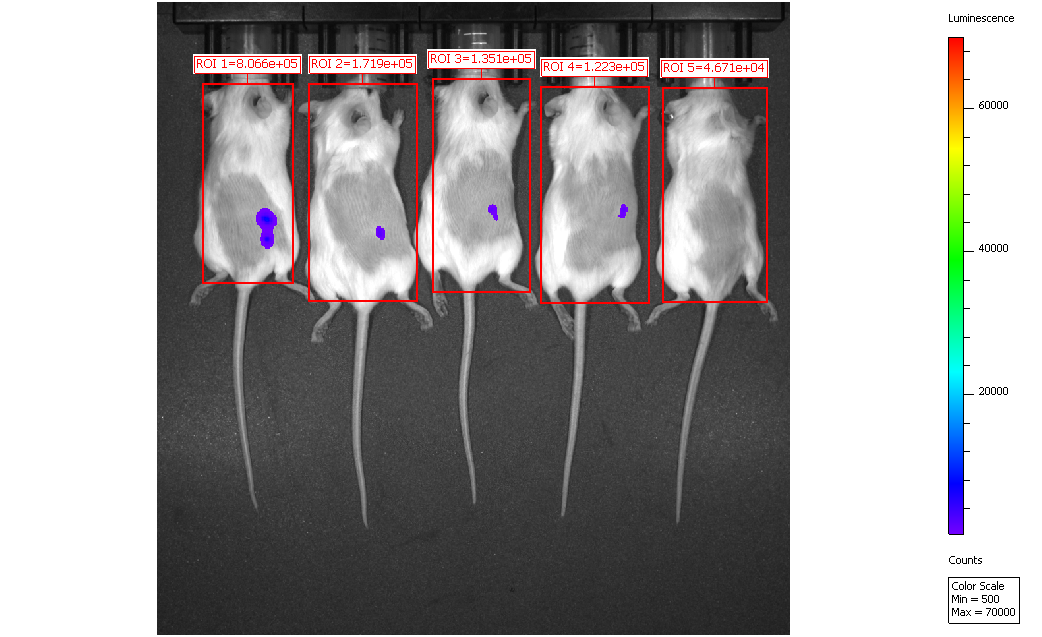

Supplement: Supplementary file 13 — Source data Fig. 7 [file 44321_2026_455_MOESM13_ESM.zip › Figure7/Panel E/DAY 3/31-35 luc.tif]

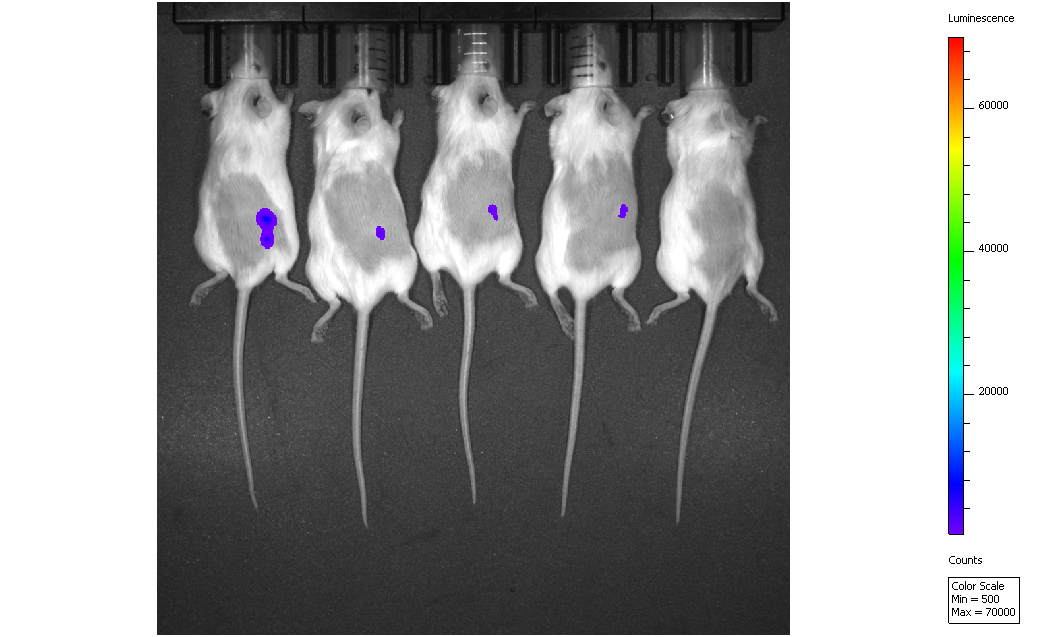

Supplement: Supplementary file 13 — Source data Fig. 7 [file 44321_2026_455_MOESM13_ESM.zip › Figure7/Panel E/DAY 3/31-35.tif]

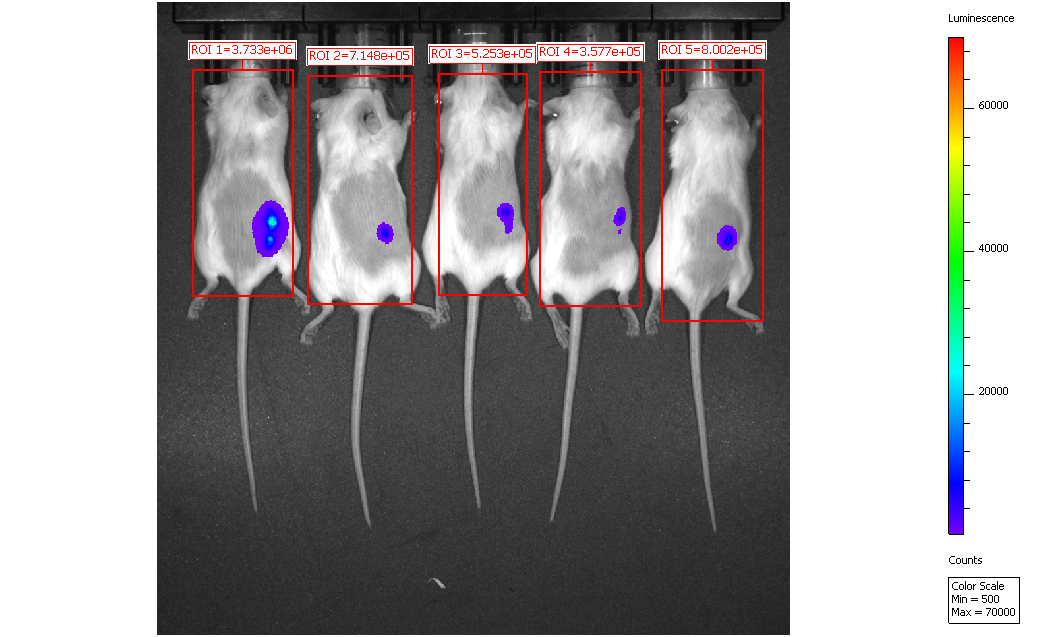

Supplement: Supplementary file 13 — Source data Fig. 7 [file 44321_2026_455_MOESM13_ESM.zip › Figure7/Panel E/DAY 3/36-40 luc.tif]

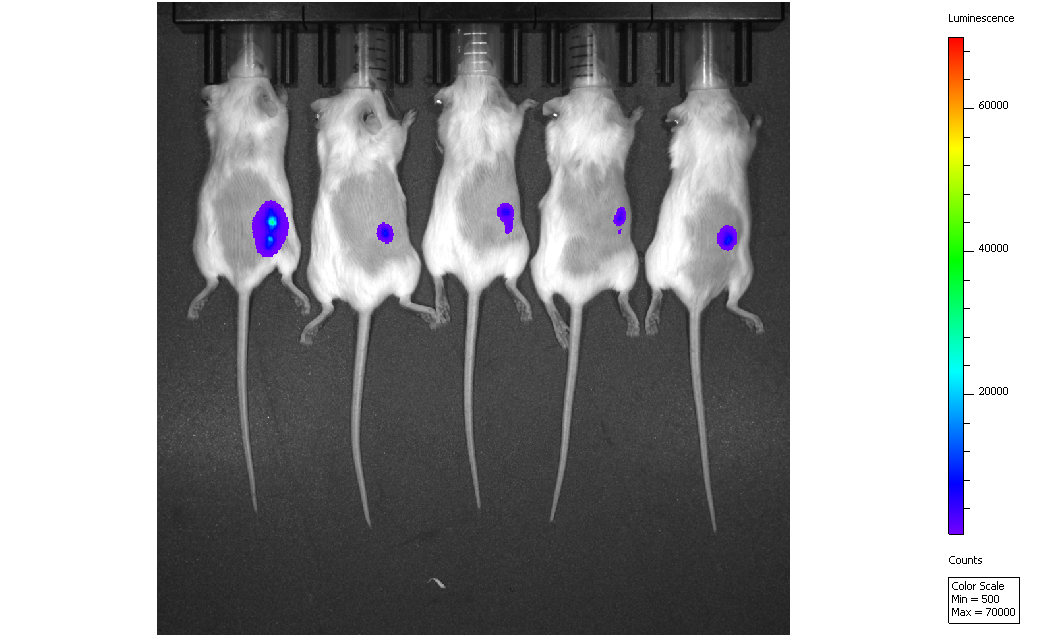

Supplement: Supplementary file 13 — Source data Fig. 7 [file 44321_2026_455_MOESM13_ESM.zip › Figure7/Panel E/DAY 3/36-40.tif]

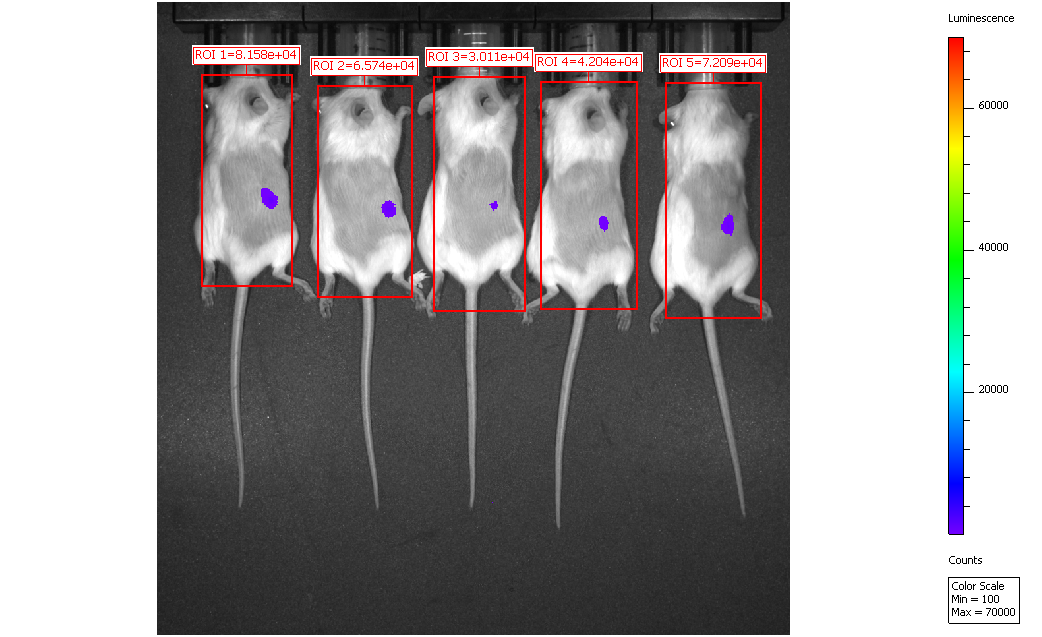

Supplement: Supplementary file 13 — Source data Fig. 7 [file 44321_2026_455_MOESM13_ESM.zip › Figure7/Panel E/DAY 3/41-45 luc.tif]

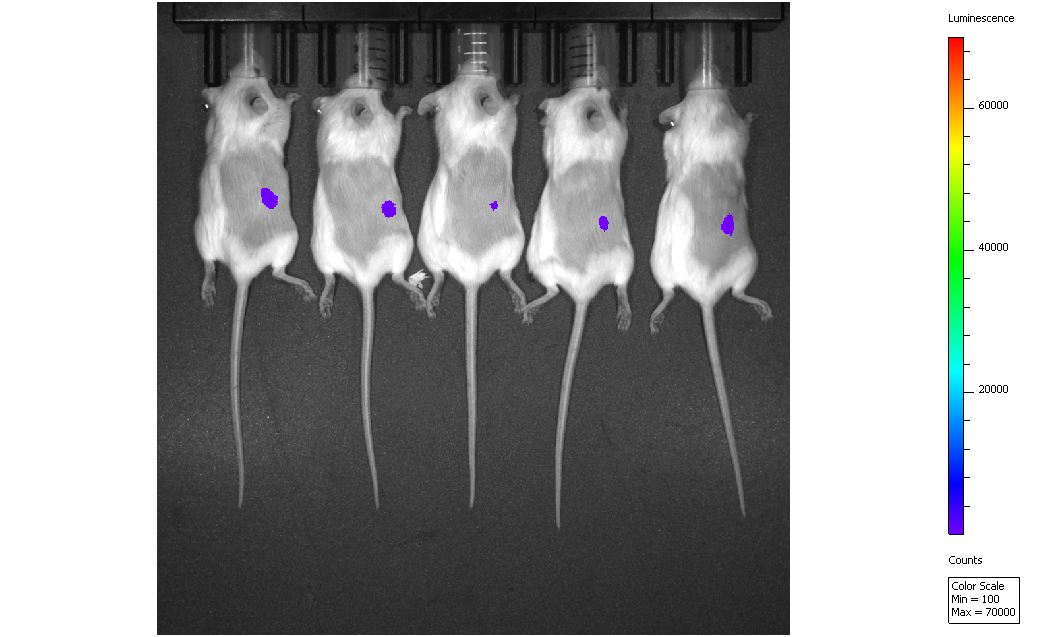

Supplement: Supplementary file 13 — Source data Fig. 7 [file 44321_2026_455_MOESM13_ESM.zip › Figure7/Panel E/DAY 3/41-45.tif]

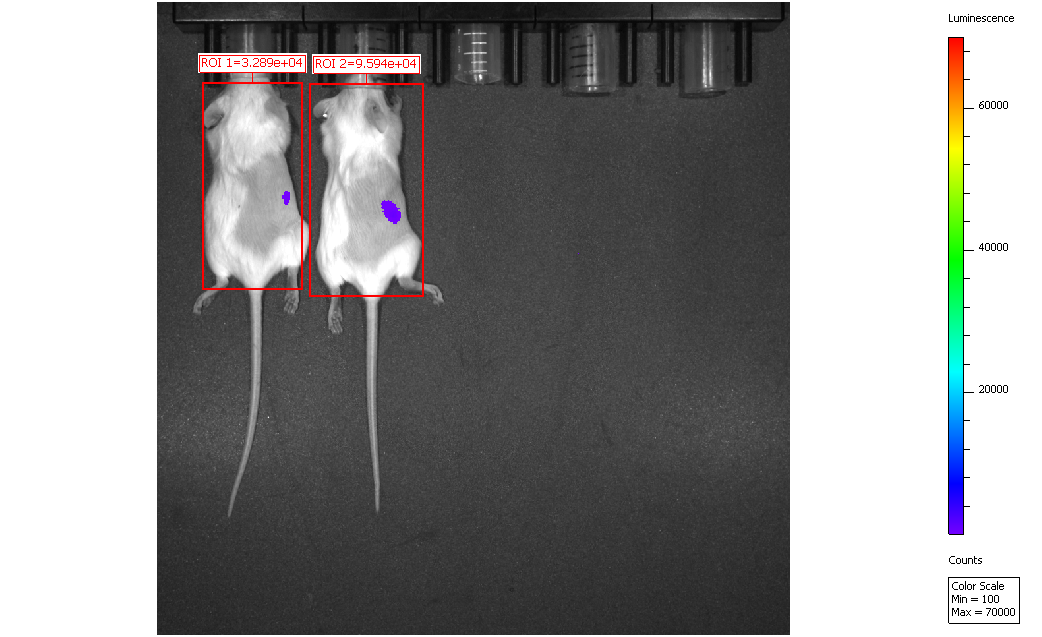

Supplement: Supplementary file 13 — Source data Fig. 7 [file 44321_2026_455_MOESM13_ESM.zip › Figure7/Panel E/DAY 3/46- 23 24 luc.tif]

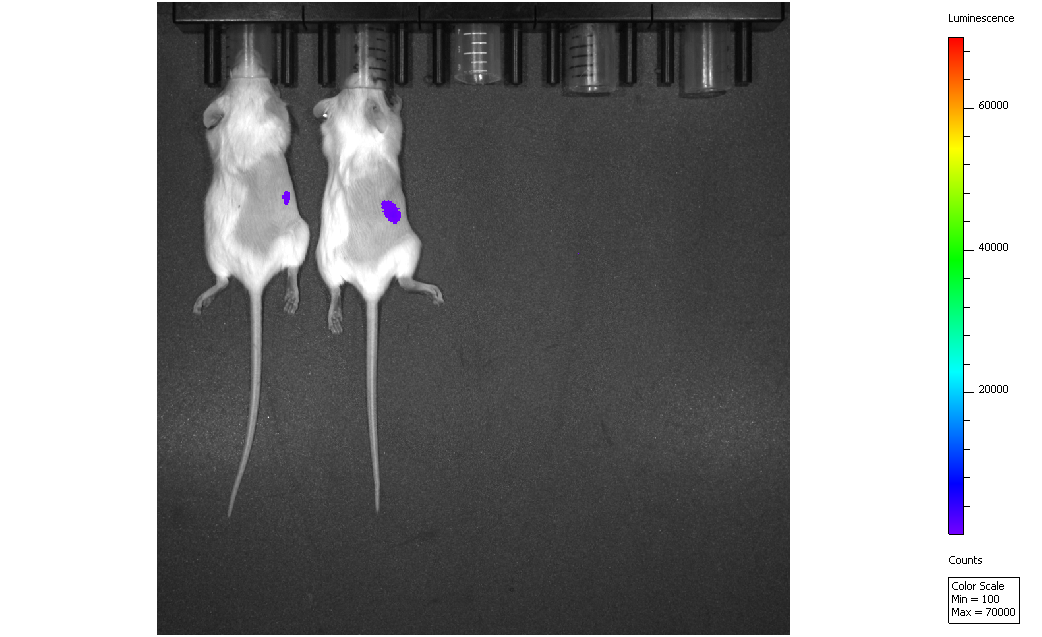

Supplement: Supplementary file 13 — Source data Fig. 7 [file 44321_2026_455_MOESM13_ESM.zip › Figure7/Panel E/DAY 3/46- 23 24.tif]

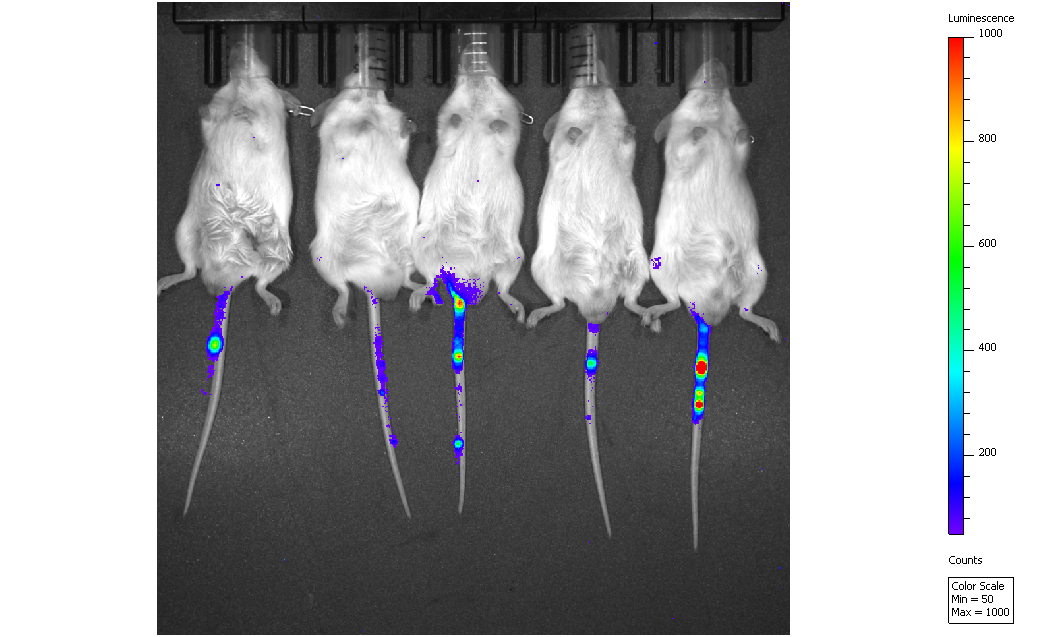

Supplement: Supplementary file 13 — Source data Fig. 7 [file 44321_2026_455_MOESM13_ESM.zip › Figure7/Panel E/DAY 3/51-55.tif]

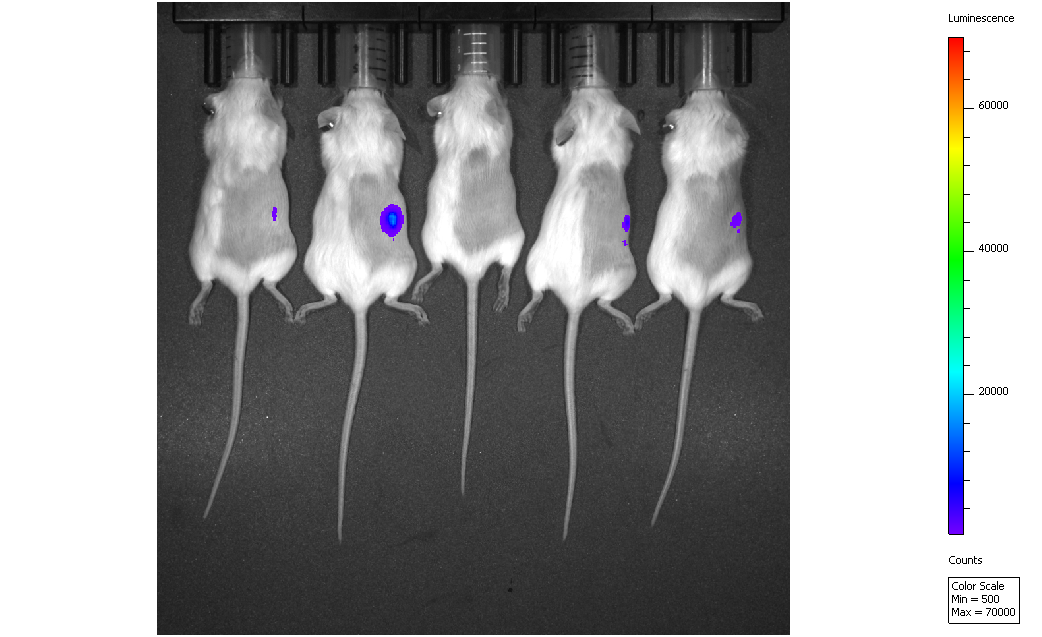

Supplement: Supplementary file 13 — Source data Fig. 7 [file 44321_2026_455_MOESM13_ESM.zip › Figure7/Panel E/DAY 3/6-10-.tif]

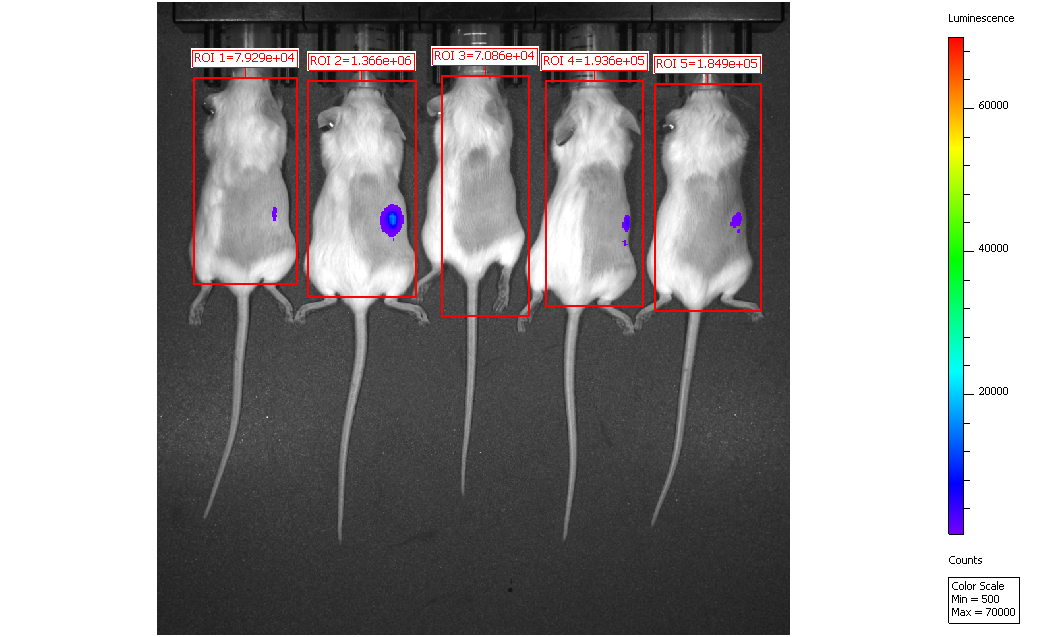

Supplement: Supplementary file 13 — Source data Fig. 7 [file 44321_2026_455_MOESM13_ESM.zip › Figure7/Panel E/DAY 3/6-10.tif]

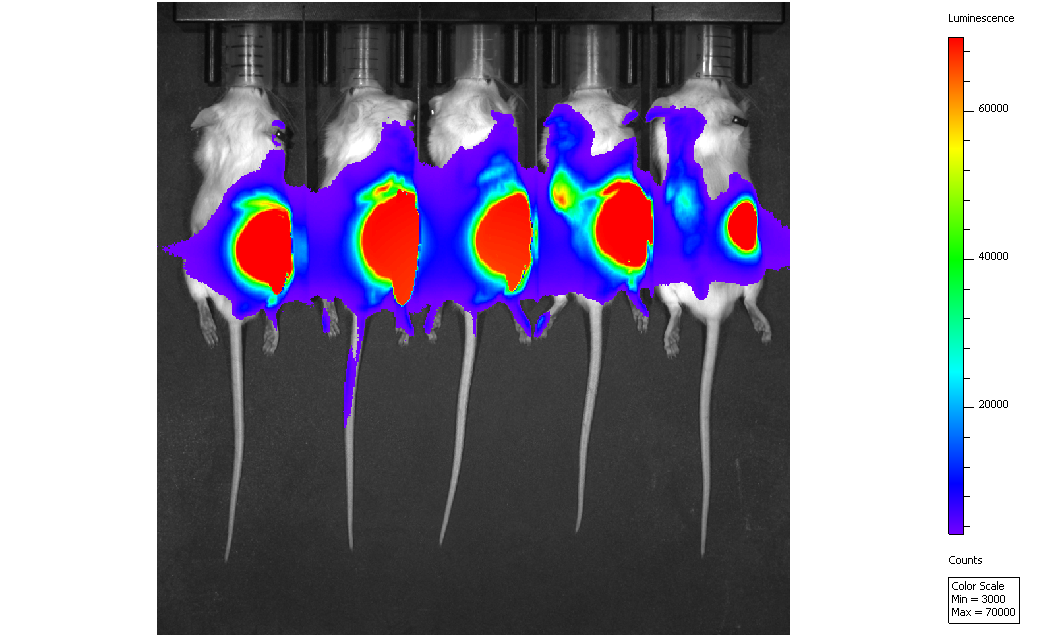

Supplement: Supplementary file 13 — Source data Fig. 7 [file 44321_2026_455_MOESM13_ESM.zip › Figure7/Panel H/DAY 17/1- 3 5 7 18 11.tif]

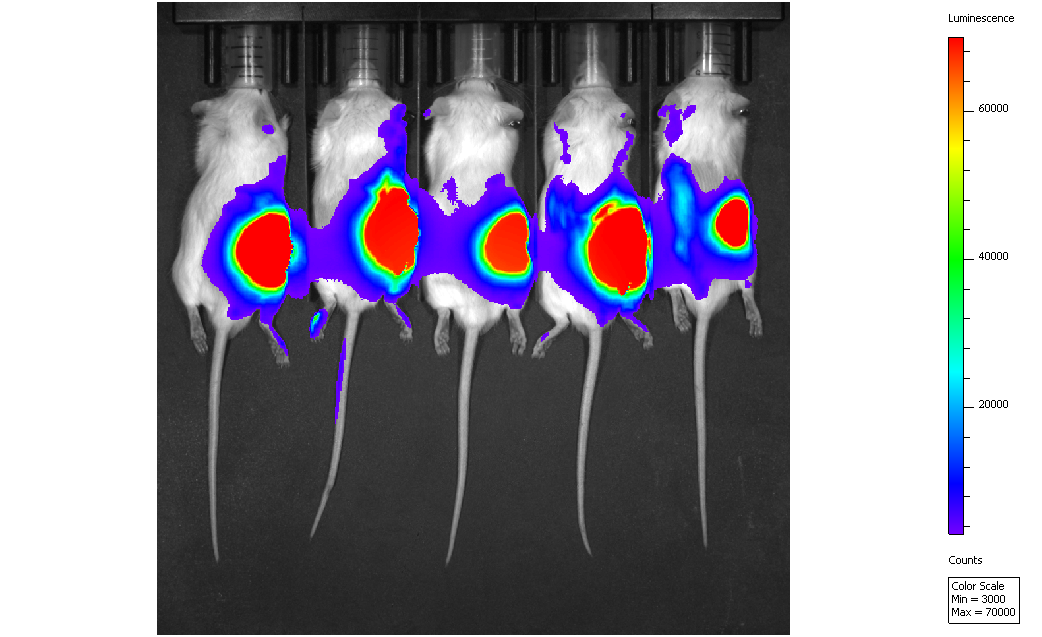

Supplement: Supplementary file 13 — Source data Fig. 7 [file 44321_2026_455_MOESM13_ESM.zip › Figure7/Panel H/DAY 17/2- 9 13 16 21 23.tif]
