# Supplementary figures and images for: Functional screening of TCR-like antibodies using STAR-T cell library for cancer immunotherapy (part 2 of 2)
Source: EMBO Mol Med. 2026 Jun 8;18(7):2748–76. doi: 10.1038/s44321-026-00455-z (PMC13365543; doi:10.1038/s44321-026-00455-z)

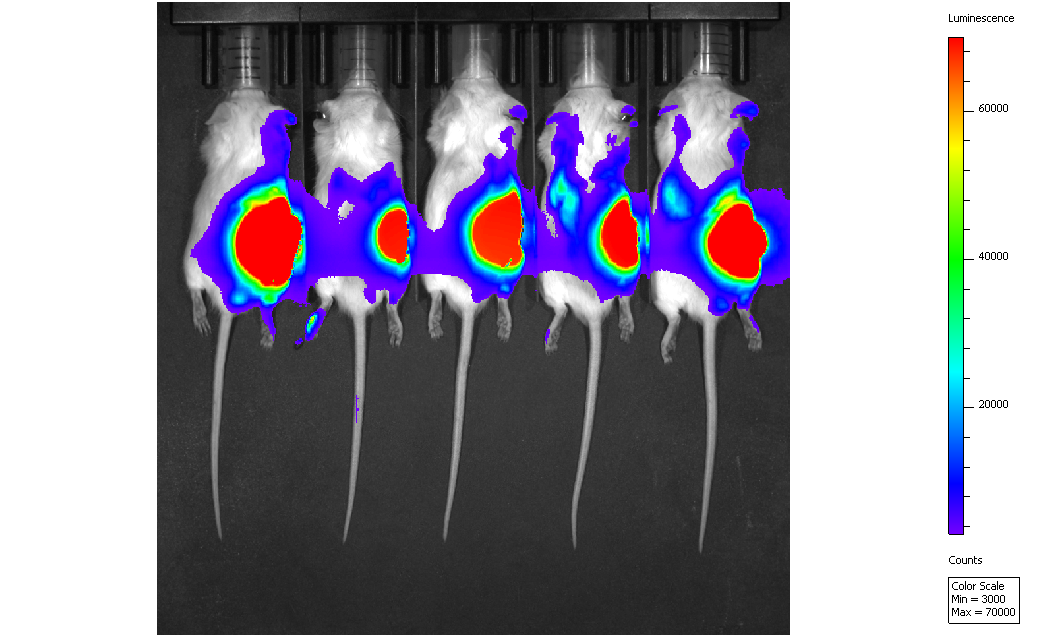

Supplement: Supplementary file 13 — Source data Fig. 7 [file 44321_2026_455_MOESM13_ESM.zip › Figure7/Panel H/DAY 17/3- 1 6 14 20 22.tif]

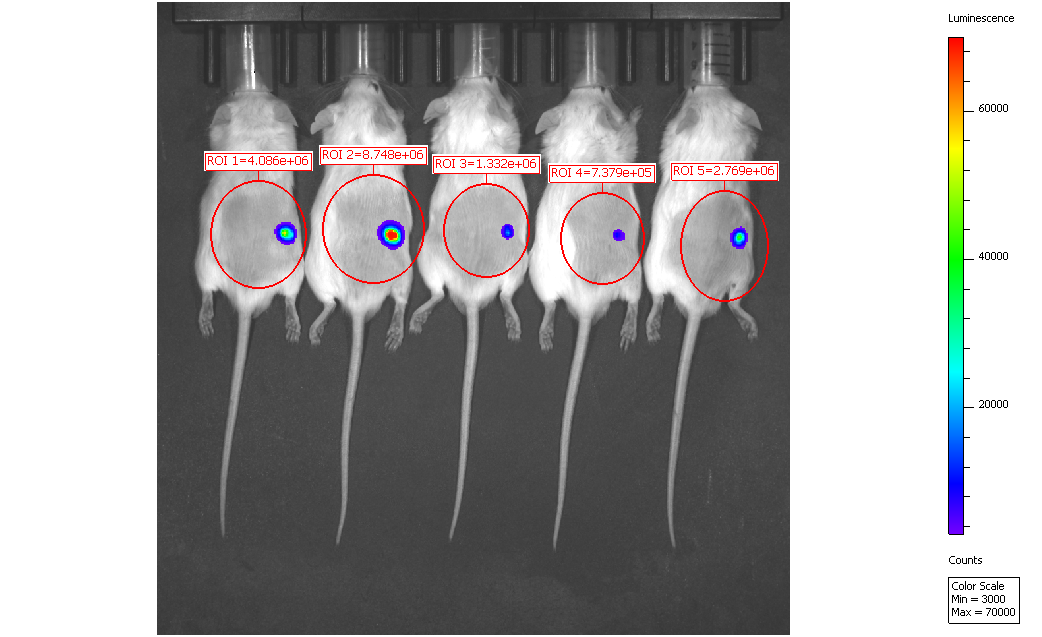

Supplement: Supplementary file 13 — Source data Fig. 7 [file 44321_2026_455_MOESM13_ESM.zip › Figure7/Panel H/DAY 3/1-5-luc.tif]

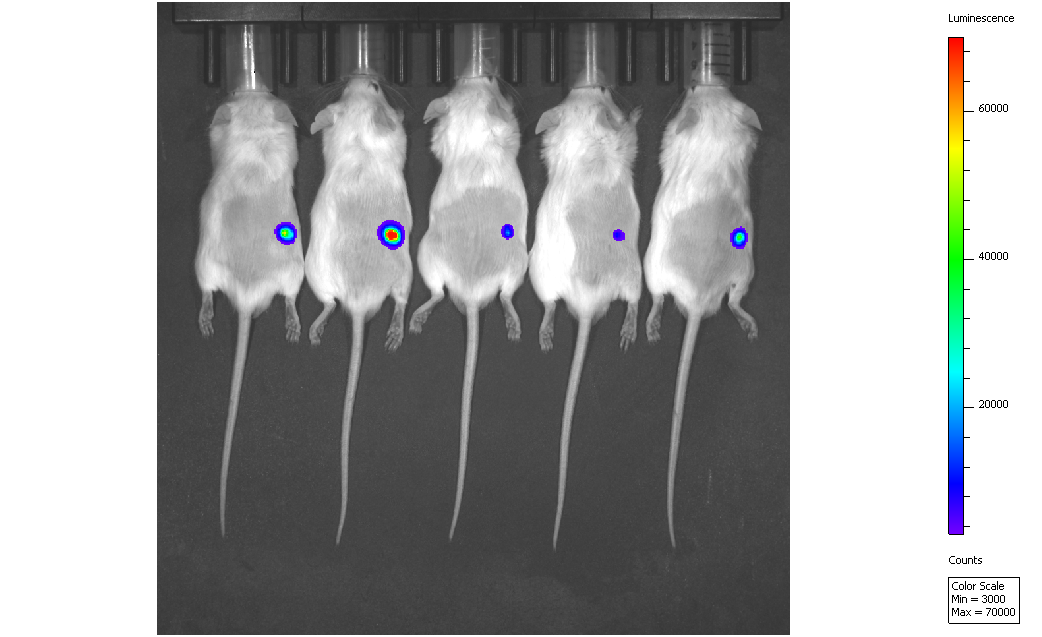

Supplement: Supplementary file 13 — Source data Fig. 7 [file 44321_2026_455_MOESM13_ESM.zip › Figure7/Panel H/DAY 3/1-5.tif]

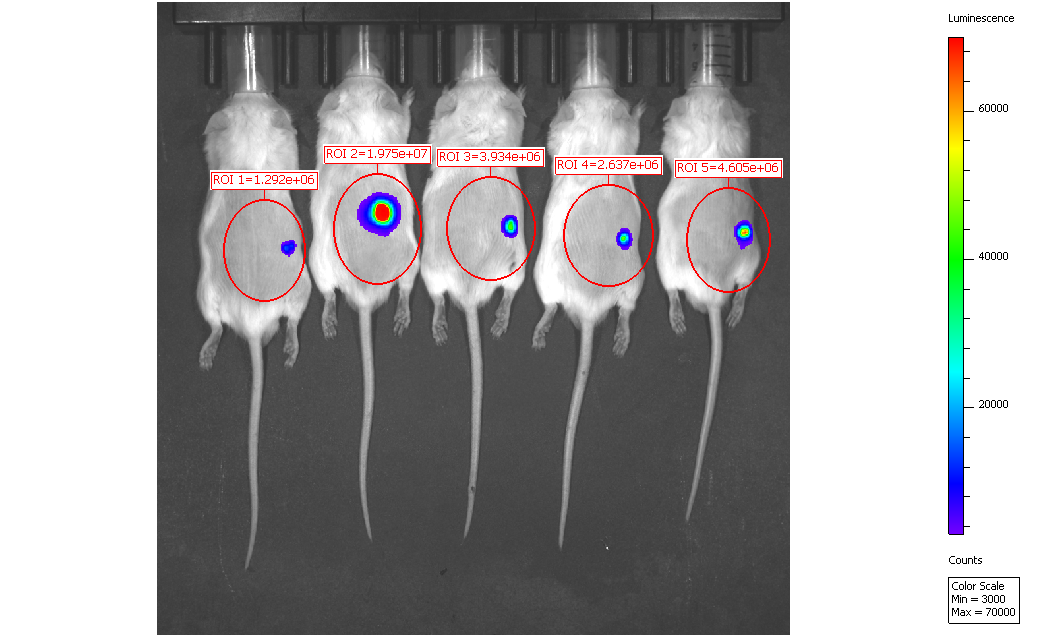

Supplement: Supplementary file 13 — Source data Fig. 7 [file 44321_2026_455_MOESM13_ESM.zip › Figure7/Panel H/DAY 3/11-15-luc.tif]

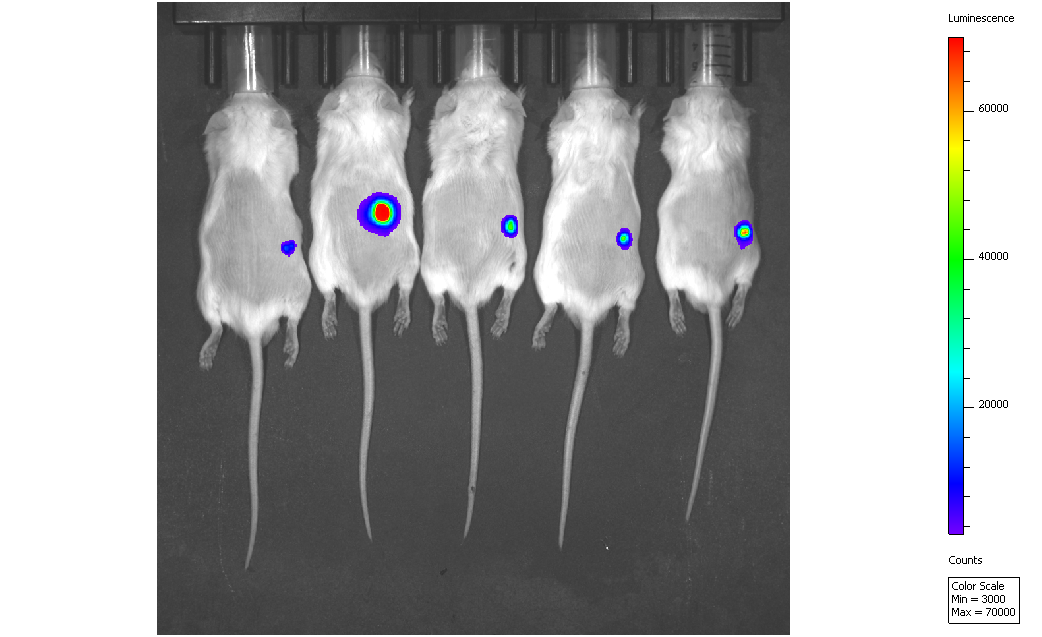

Supplement: Supplementary file 13 — Source data Fig. 7 [file 44321_2026_455_MOESM13_ESM.zip › Figure7/Panel H/DAY 3/11-15.tif]

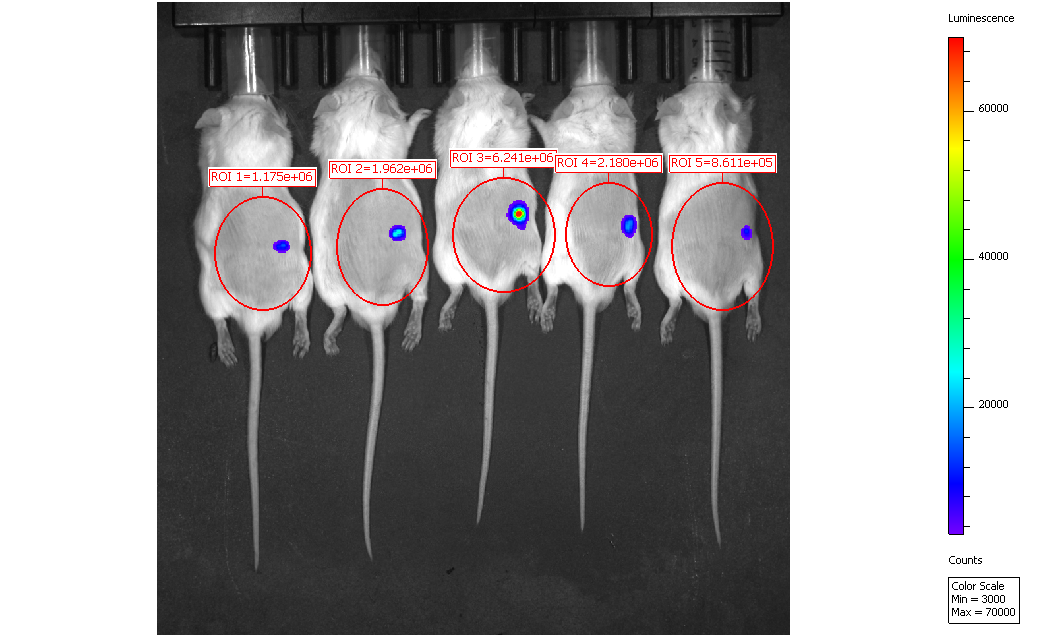

Supplement: Supplementary file 13 — Source data Fig. 7 [file 44321_2026_455_MOESM13_ESM.zip › Figure7/Panel H/DAY 3/16-20-luc.tif]

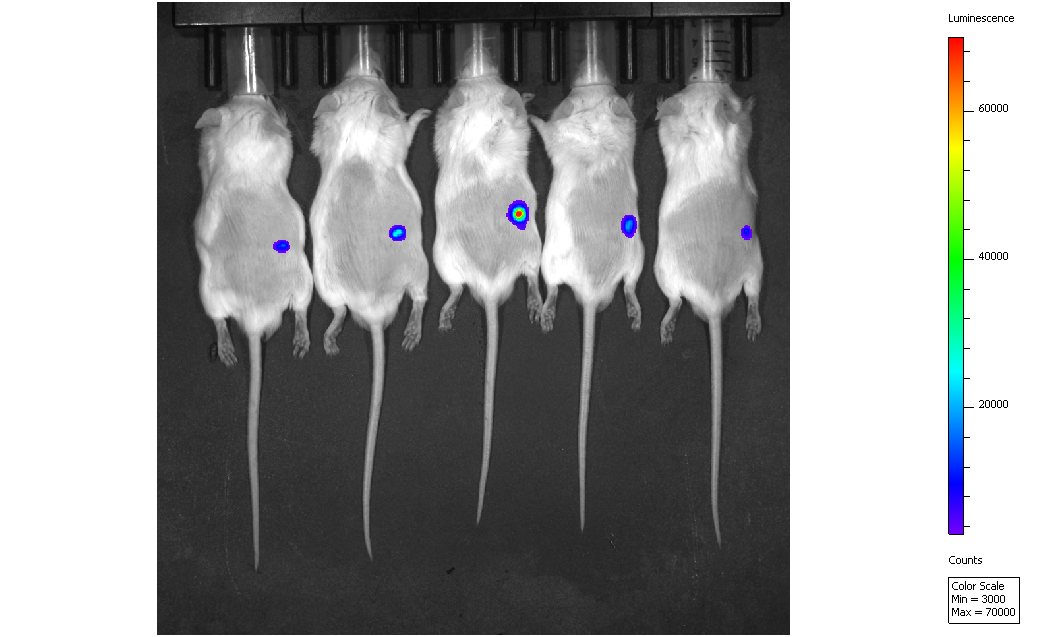

Supplement: Supplementary file 13 — Source data Fig. 7 [file 44321_2026_455_MOESM13_ESM.zip › Figure7/Panel H/DAY 3/16-20.tif]

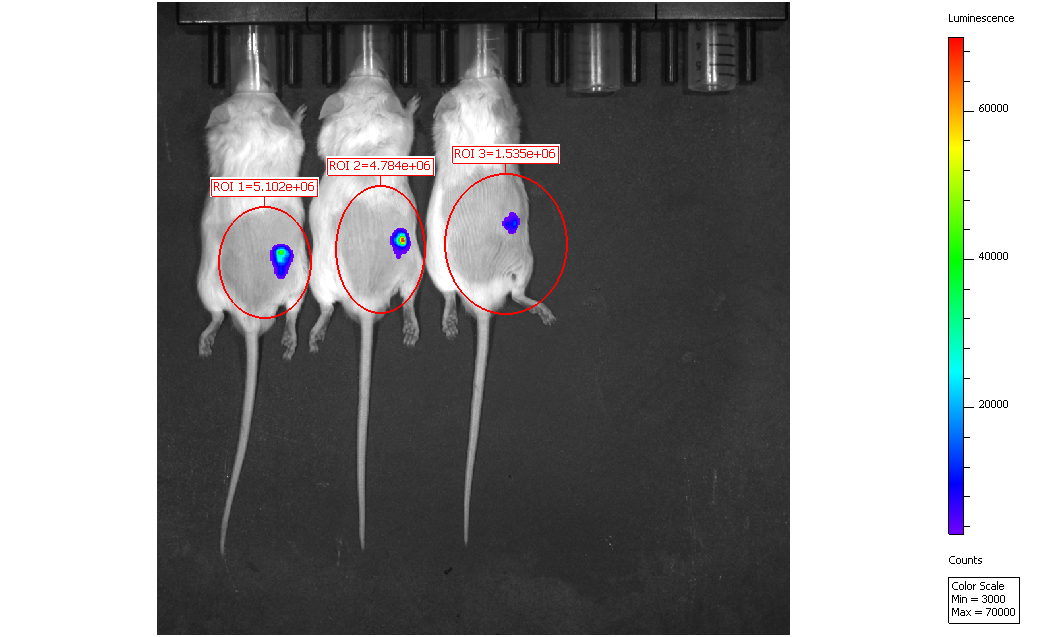

Supplement: Supplementary file 13 — Source data Fig. 7 [file 44321_2026_455_MOESM13_ESM.zip › Figure7/Panel H/DAY 3/21 22 23-luc.tif]

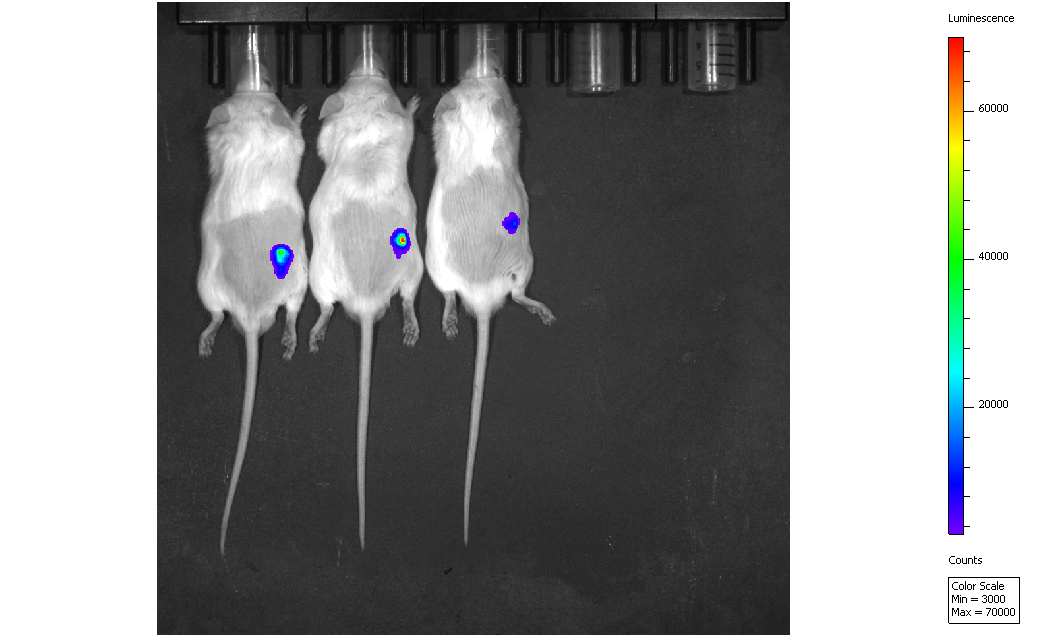

Supplement: Supplementary file 13 — Source data Fig. 7 [file 44321_2026_455_MOESM13_ESM.zip › Figure7/Panel H/DAY 3/21 22 23.tif]

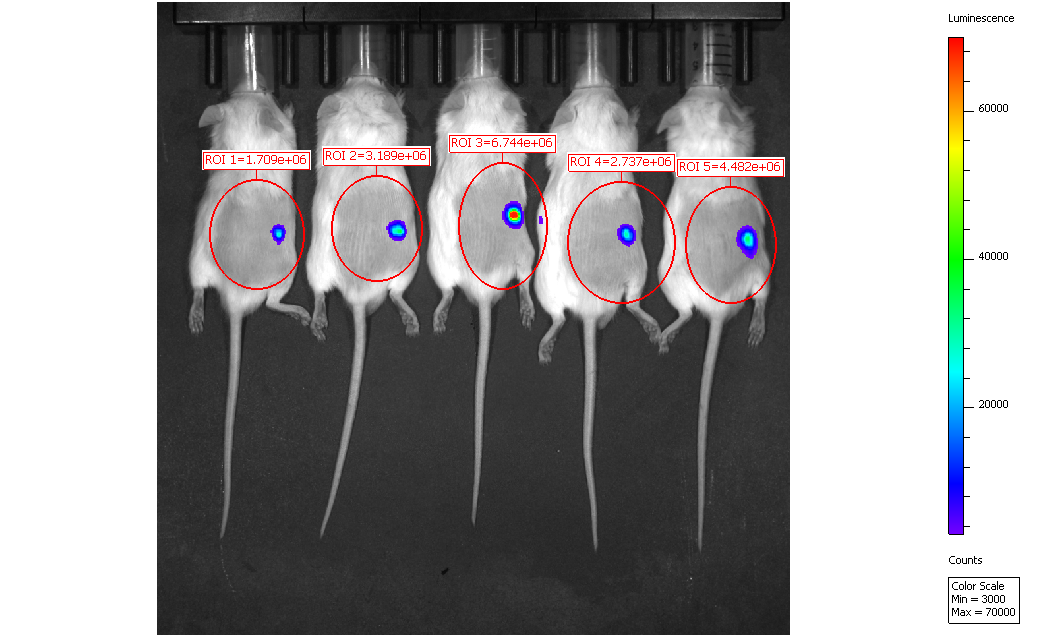

Supplement: Supplementary file 13 — Source data Fig. 7 [file 44321_2026_455_MOESM13_ESM.zip › Figure7/Panel H/DAY 3/6-10-luc.tif]

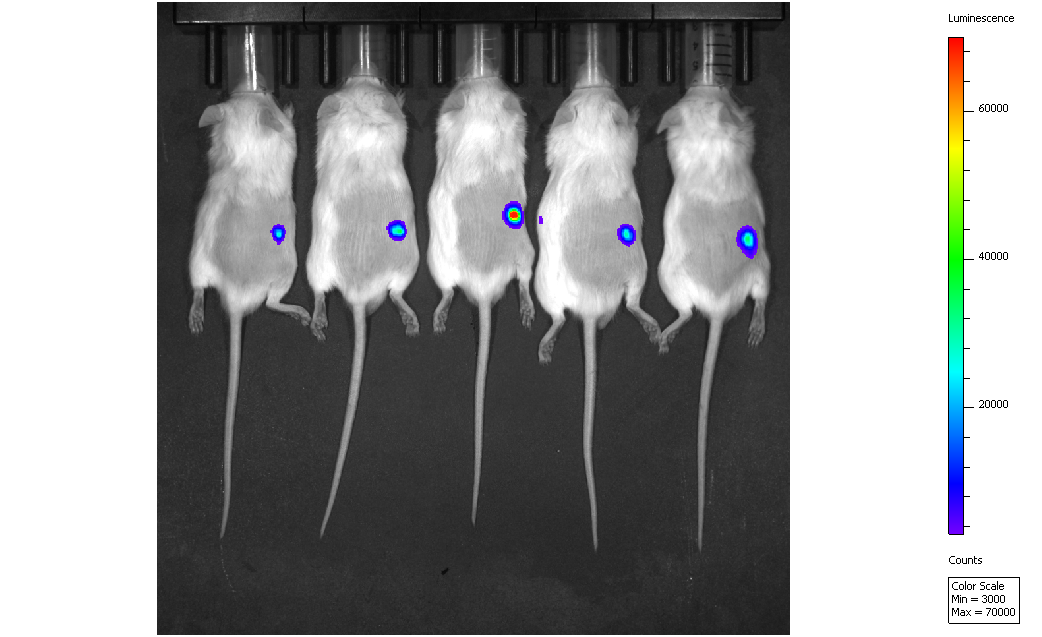

Supplement: Supplementary file 13 — Source data Fig. 7 [file 44321_2026_455_MOESM13_ESM.zip › Figure7/Panel H/DAY 3/6-10.tif]

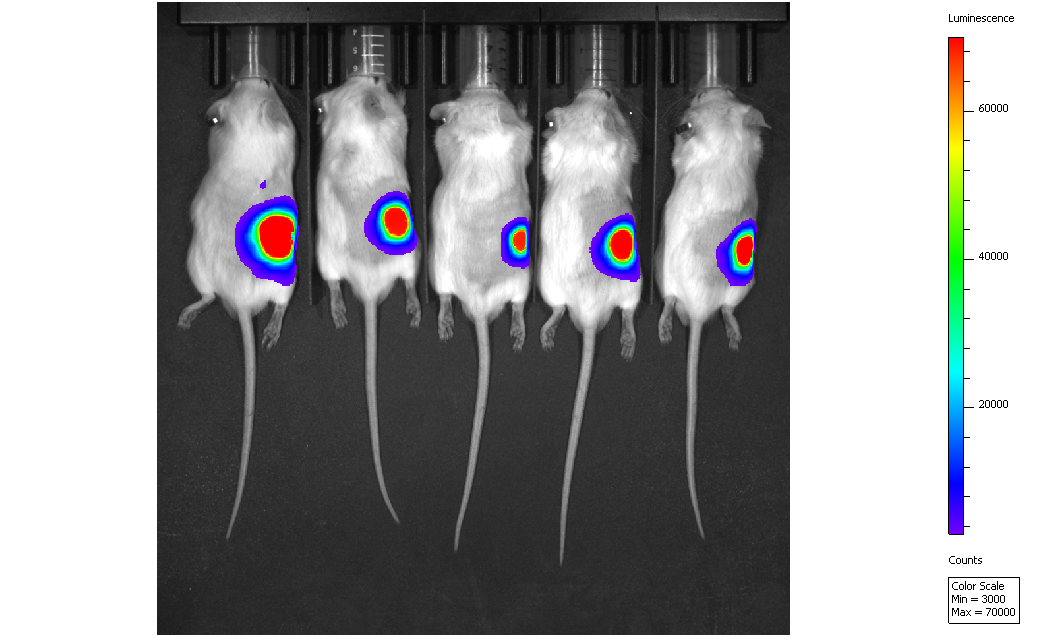

Supplement: Supplementary file 14 — Source data Fig. 8 [file 44321_2026_455_MOESM14_ESM.zip › Figure8/Panel E/DAY 11/1 7 30 38 39.tif]

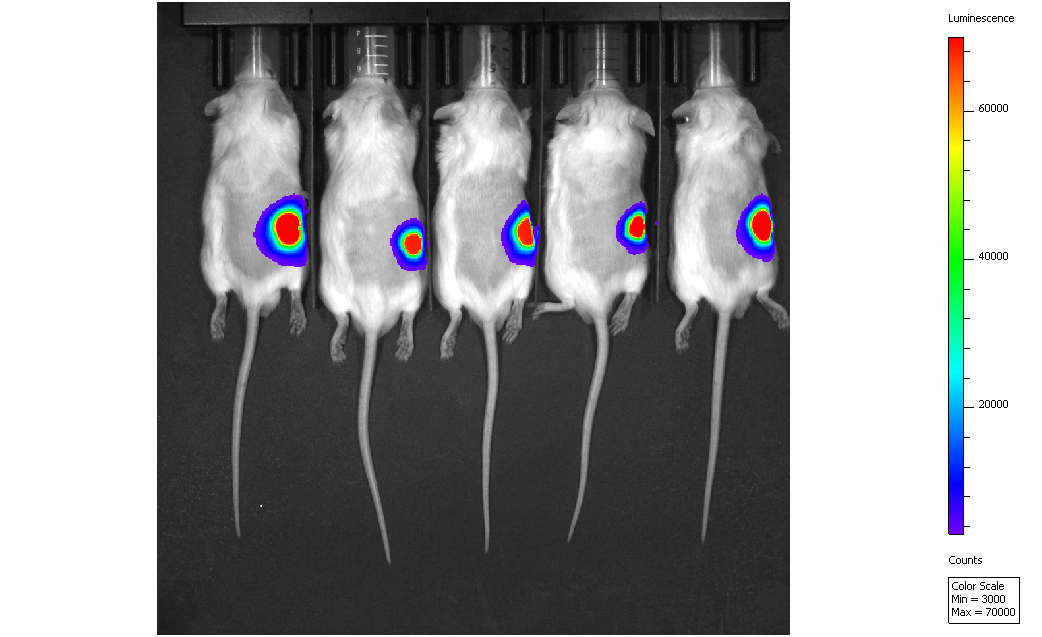

Supplement: Supplementary file 14 — Source data Fig. 8 [file 44321_2026_455_MOESM14_ESM.zip › Figure8/Panel E/DAY 11/12 13 22 31 32.tif]

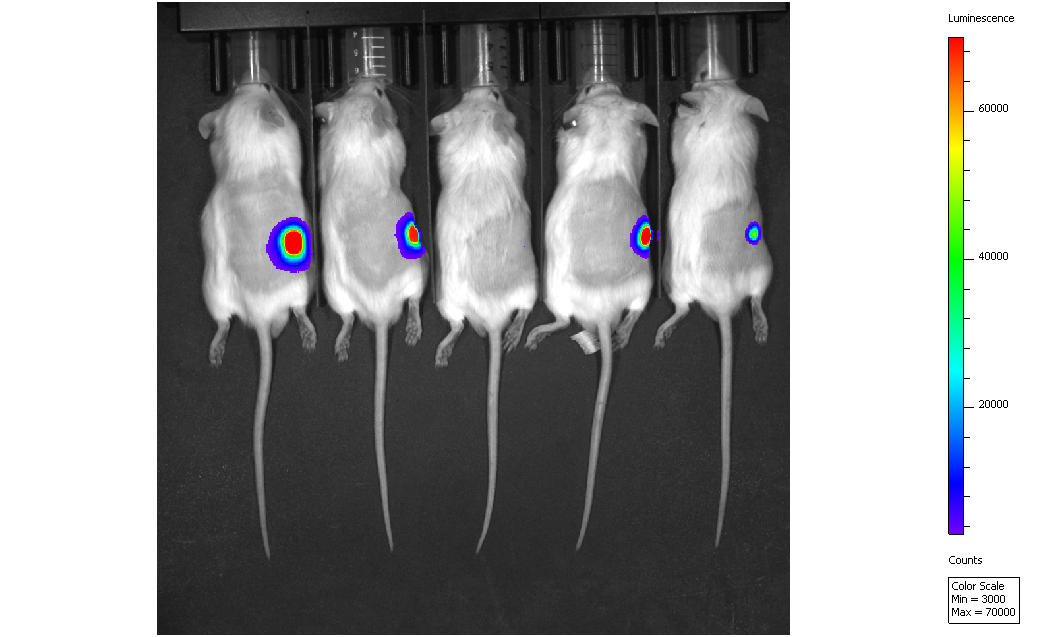

Supplement: Supplementary file 14 — Source data Fig. 8 [file 44321_2026_455_MOESM14_ESM.zip › Figure8/Panel E/DAY 11/2 14 18 26 35.tif]

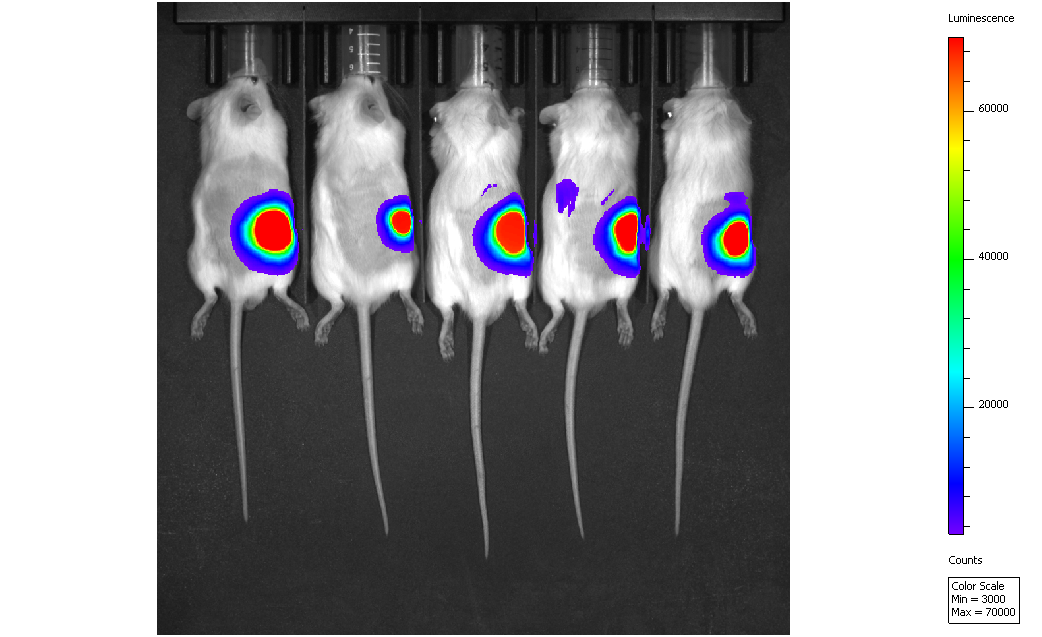

Supplement: Supplementary file 14 — Source data Fig. 8 [file 44321_2026_455_MOESM14_ESM.zip › Figure8/Panel E/DAY 11/6 8 15 36 37.tif]

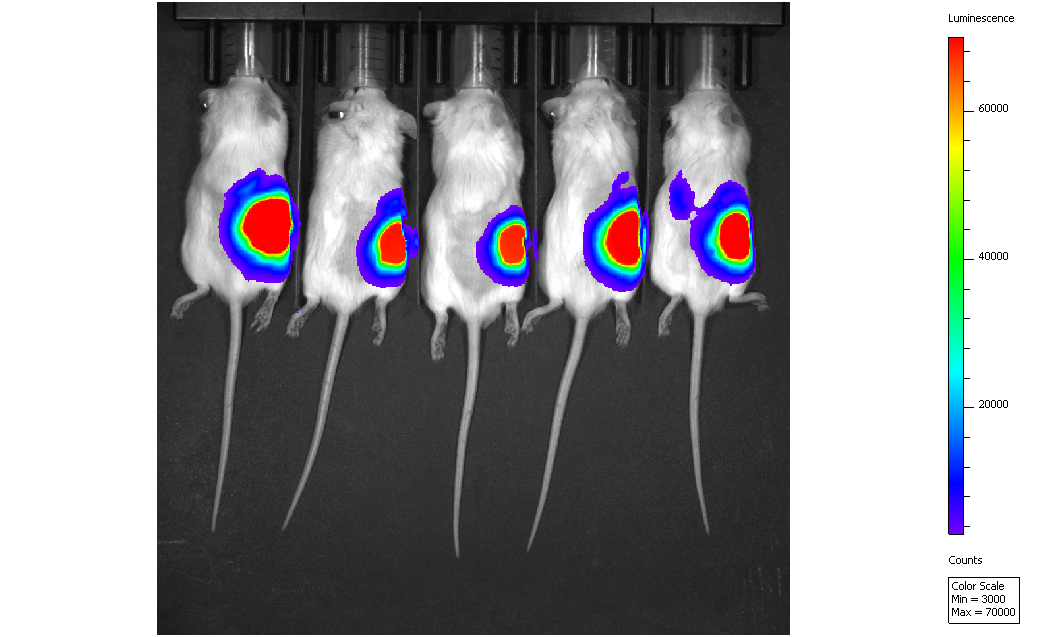

Supplement: Supplementary file 14 — Source data Fig. 8 [file 44321_2026_455_MOESM14_ESM.zip › Figure8/Panel E/DAY 14/1 7 30 38 39.tif]

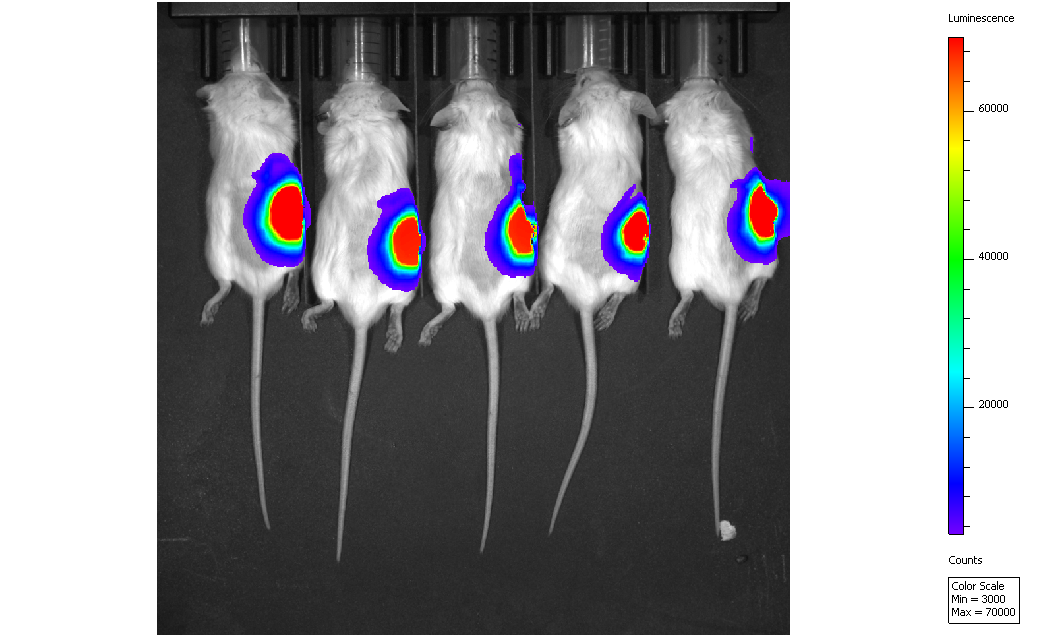

Supplement: Supplementary file 14 — Source data Fig. 8 [file 44321_2026_455_MOESM14_ESM.zip › Figure8/Panel E/DAY 14/12 13 22 31 32.tif]

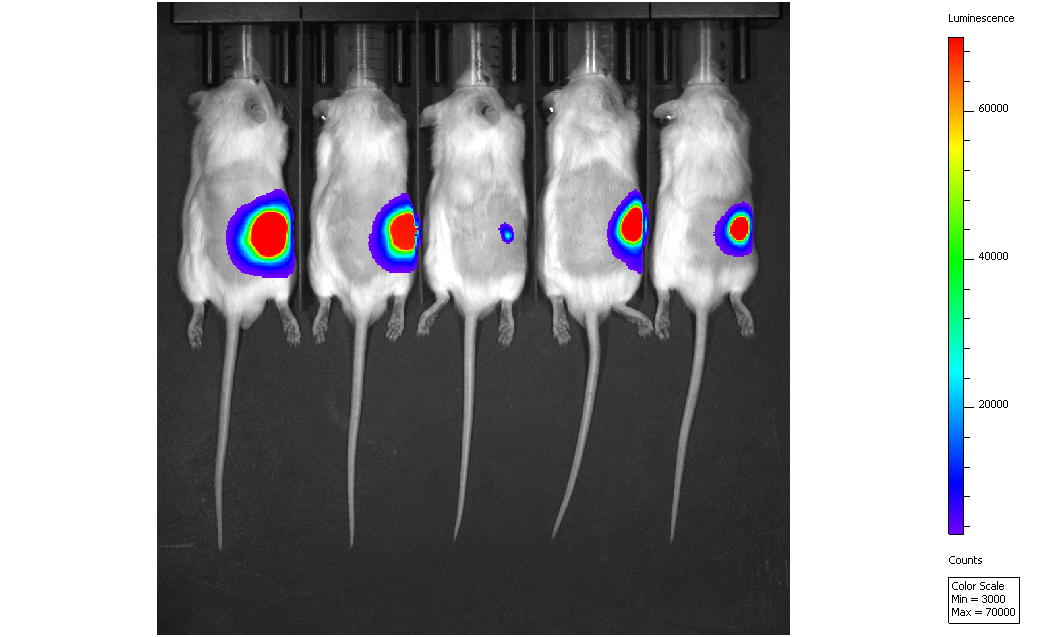

Supplement: Supplementary file 14 — Source data Fig. 8 [file 44321_2026_455_MOESM14_ESM.zip › Figure8/Panel E/DAY 14/2 14 18 26 35.tif]

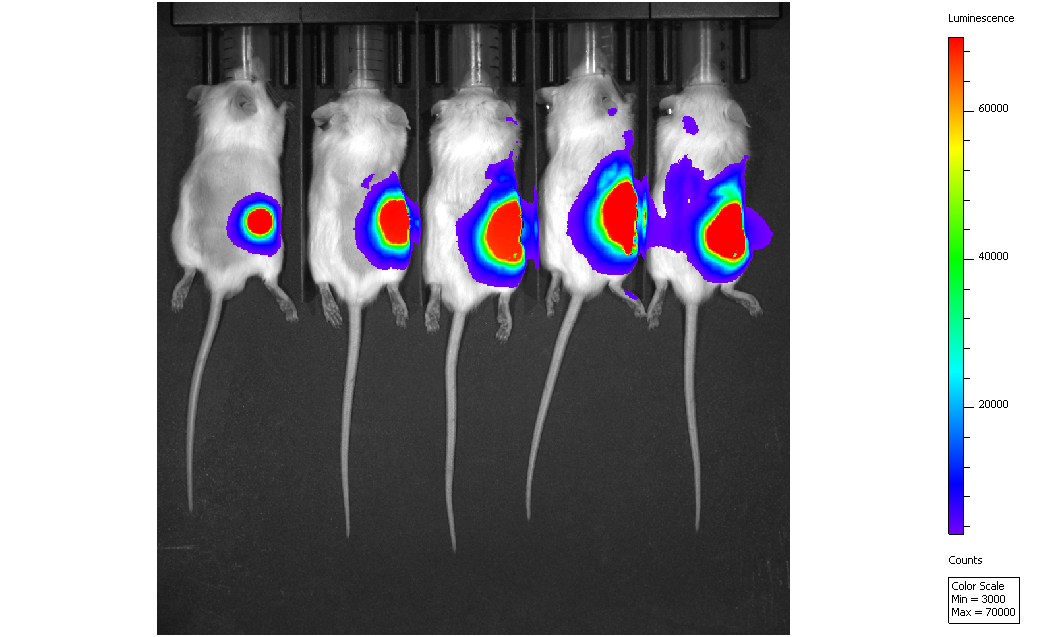

Supplement: Supplementary file 14 — Source data Fig. 8 [file 44321_2026_455_MOESM14_ESM.zip › Figure8/Panel E/DAY 14/6 8 15 36 37.tif]

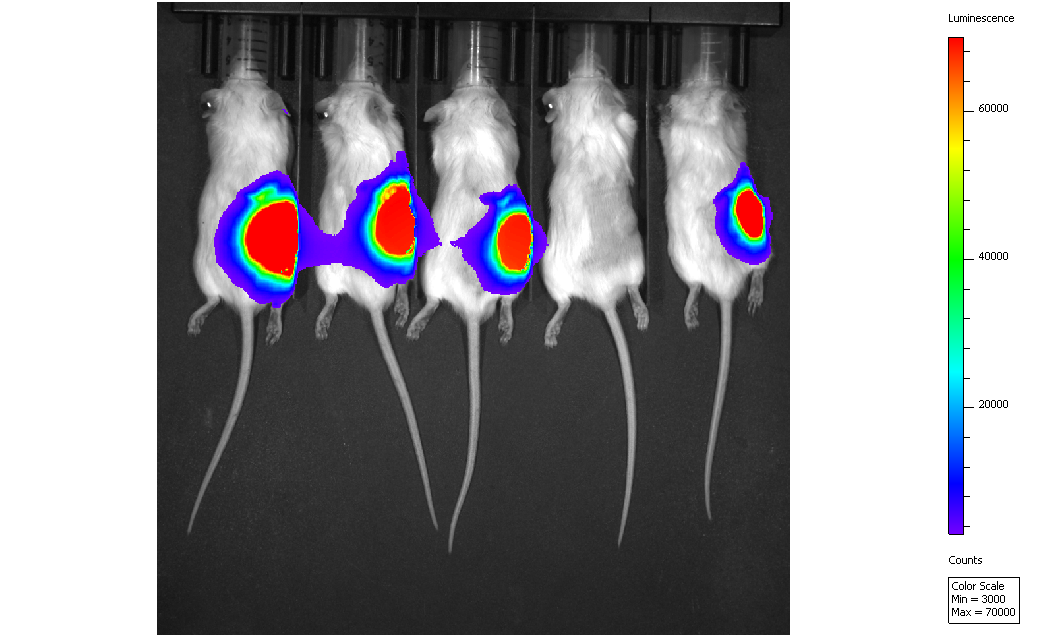

Supplement: Supplementary file 14 — Source data Fig. 8 [file 44321_2026_455_MOESM14_ESM.zip › Figure8/Panel E/DAY 17/1 7 30 38 39.tif]

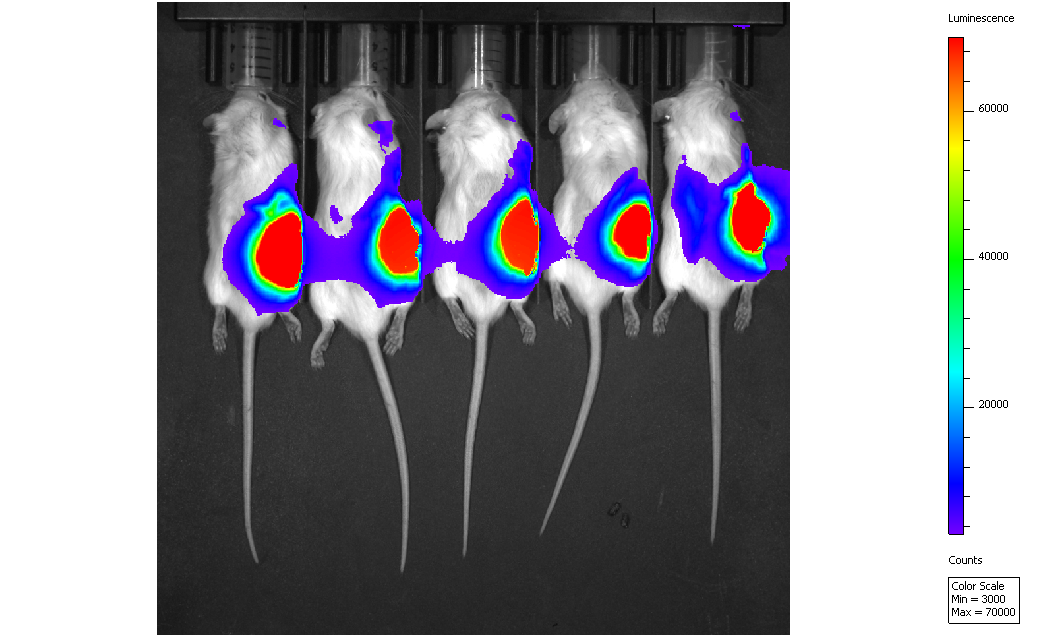

Supplement: Supplementary file 14 — Source data Fig. 8 [file 44321_2026_455_MOESM14_ESM.zip › Figure8/Panel E/DAY 17/12 13 22 31 32.tif]

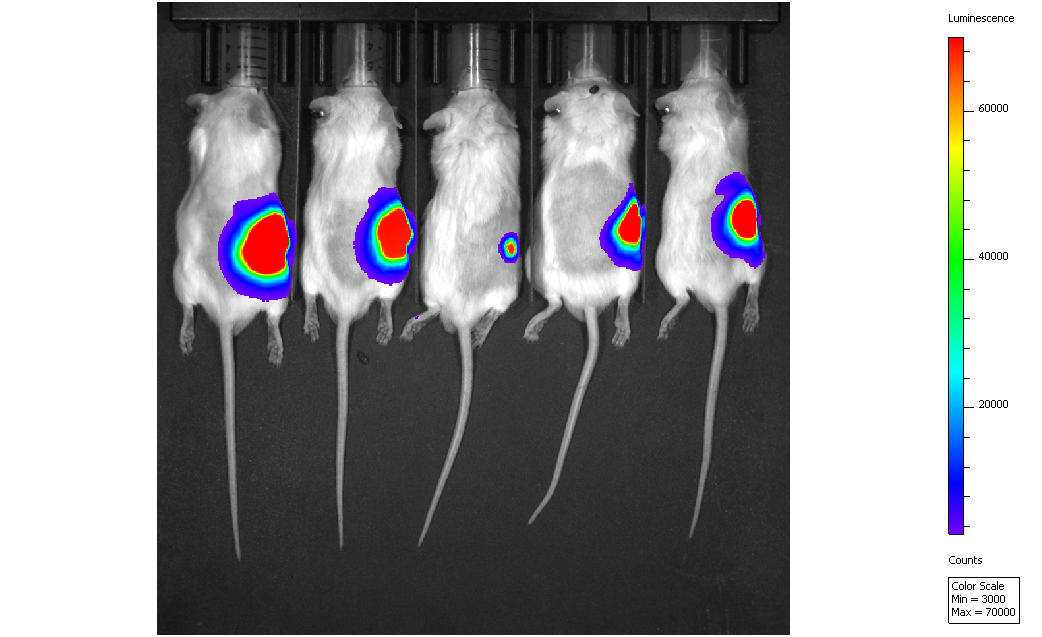

Supplement: Supplementary file 14 — Source data Fig. 8 [file 44321_2026_455_MOESM14_ESM.zip › Figure8/Panel E/DAY 17/2 14 18 26 35.tif]

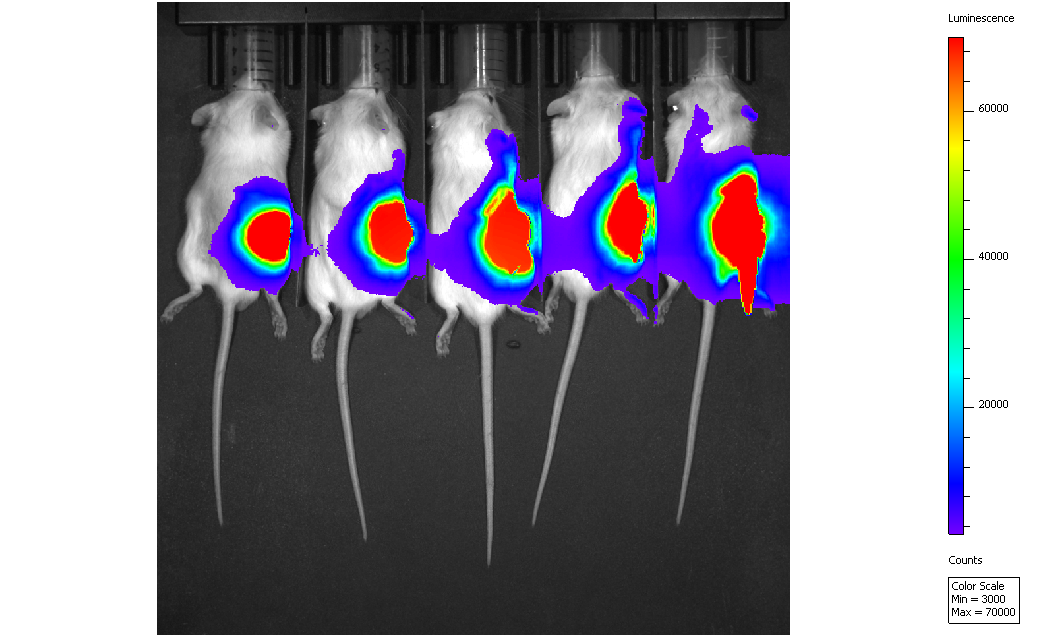

Supplement: Supplementary file 14 — Source data Fig. 8 [file 44321_2026_455_MOESM14_ESM.zip › Figure8/Panel E/DAY 17/6 8 15 37 38.tif]

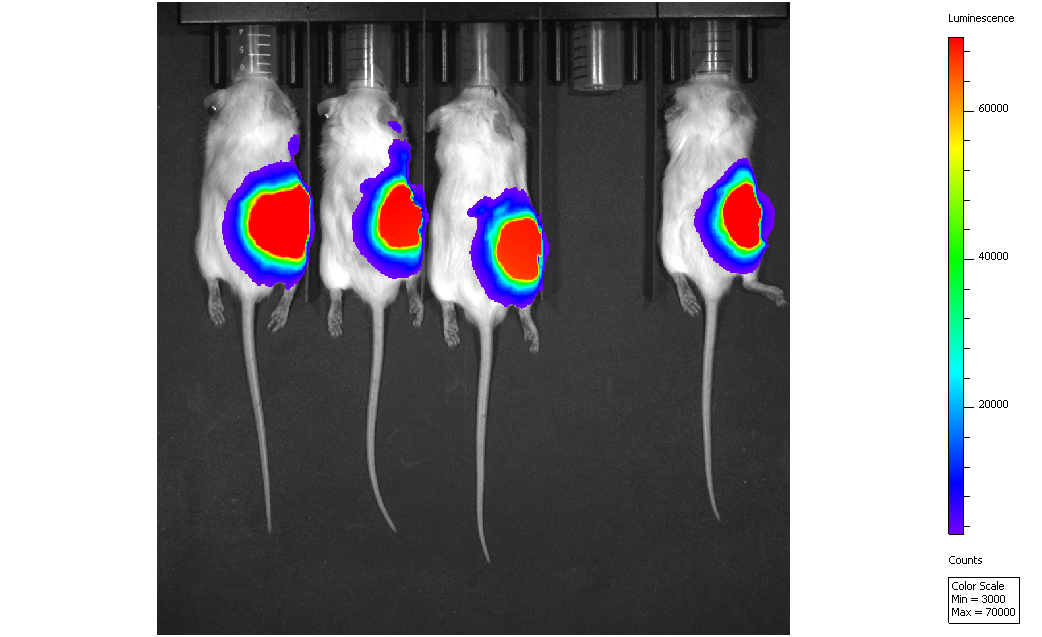

Supplement: Supplementary file 14 — Source data Fig. 8 [file 44321_2026_455_MOESM14_ESM.zip › Figure8/Panel E/DAY 21/1 7 30 39.tif]

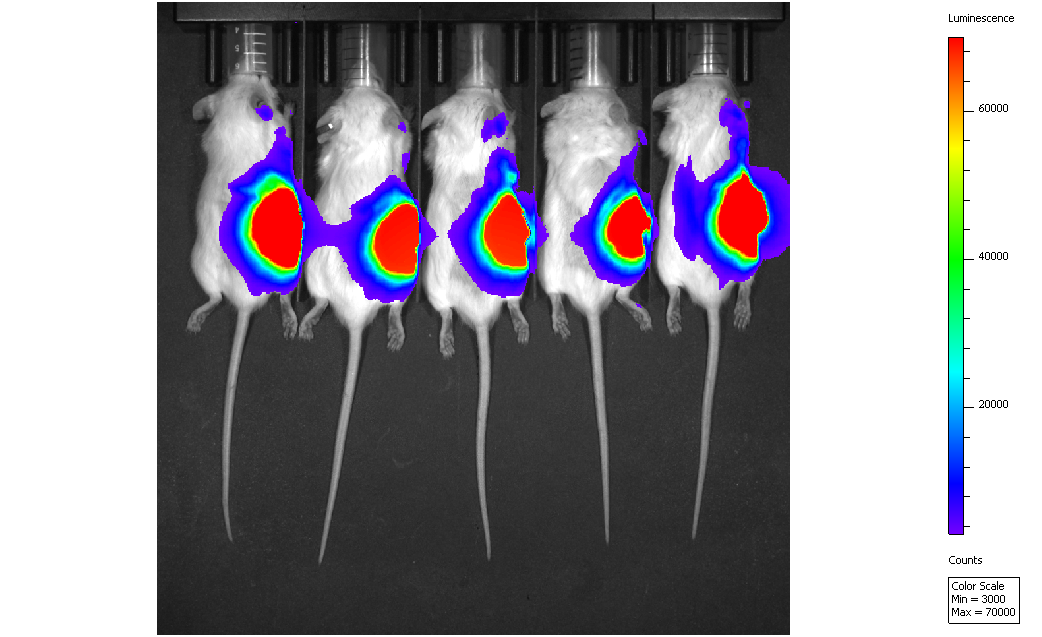

Supplement: Supplementary file 14 — Source data Fig. 8 [file 44321_2026_455_MOESM14_ESM.zip › Figure8/Panel E/DAY 21/12 13 22 31 32.tif]

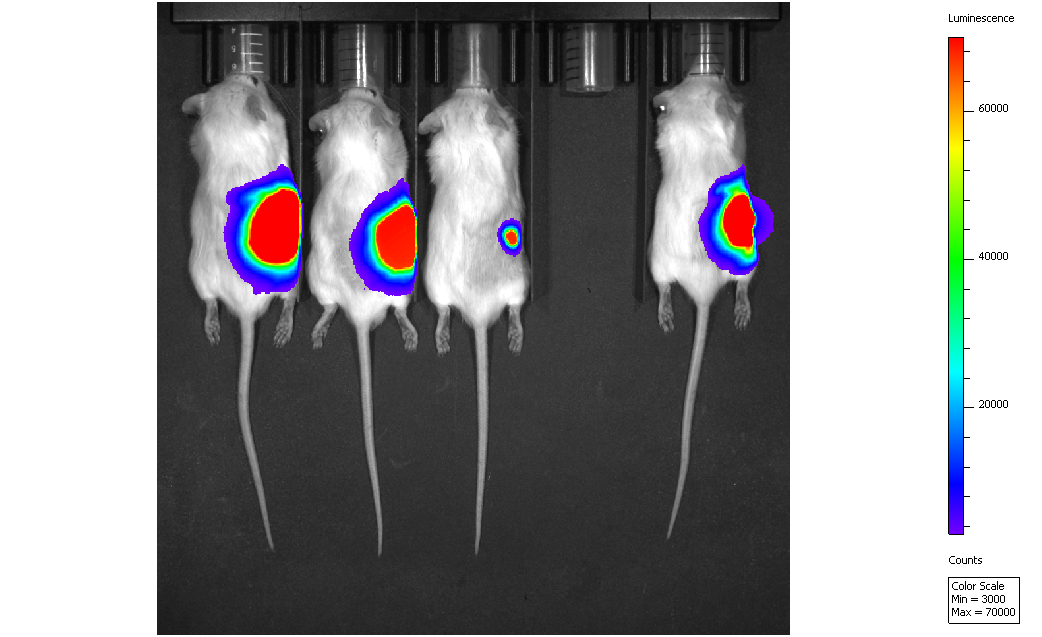

Supplement: Supplementary file 14 — Source data Fig. 8 [file 44321_2026_455_MOESM14_ESM.zip › Figure8/Panel E/DAY 21/2 14 18 35.tif]

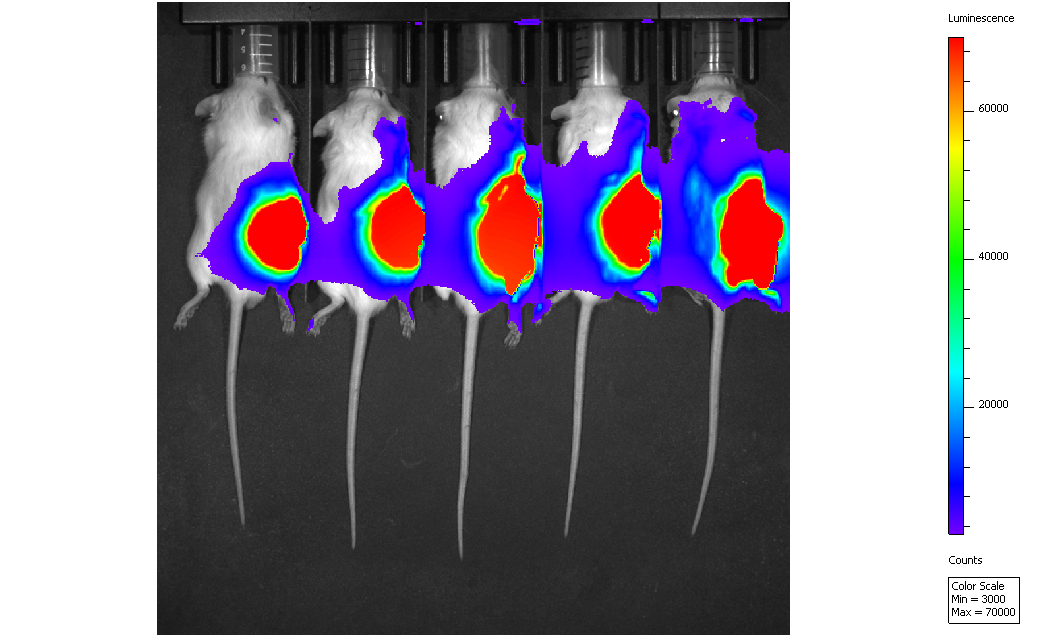

Supplement: Supplementary file 14 — Source data Fig. 8 [file 44321_2026_455_MOESM14_ESM.zip › Figure8/Panel E/DAY 21/6 8 15 36 37.tif]

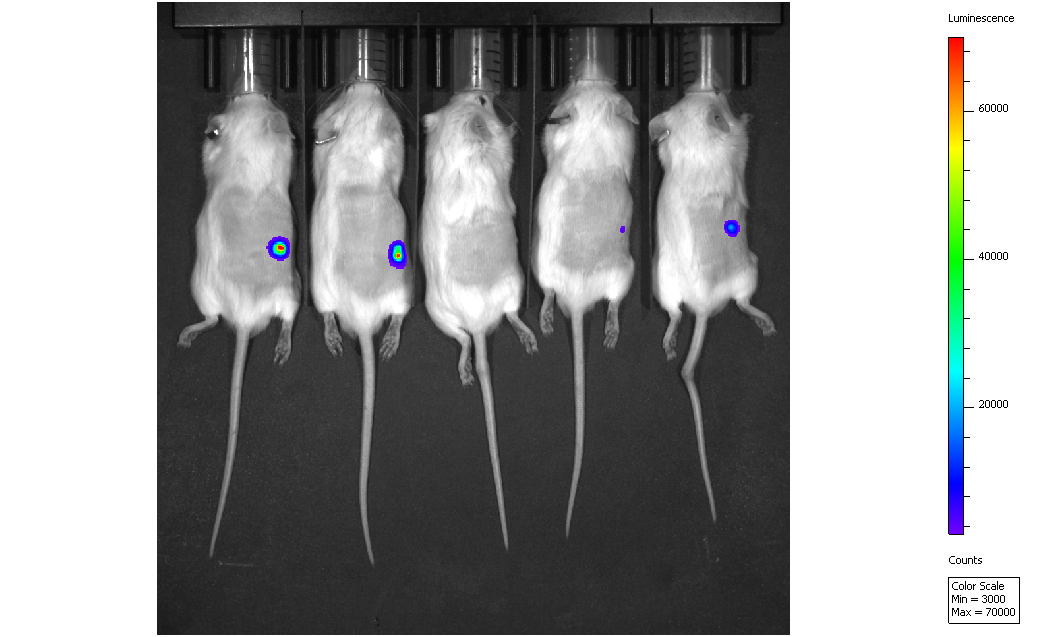

Supplement: Supplementary file 14 — Source data Fig. 8 [file 44321_2026_455_MOESM14_ESM.zip › Figure8/Panel E/DAY 3/1 2 3 4 5(1).tif]

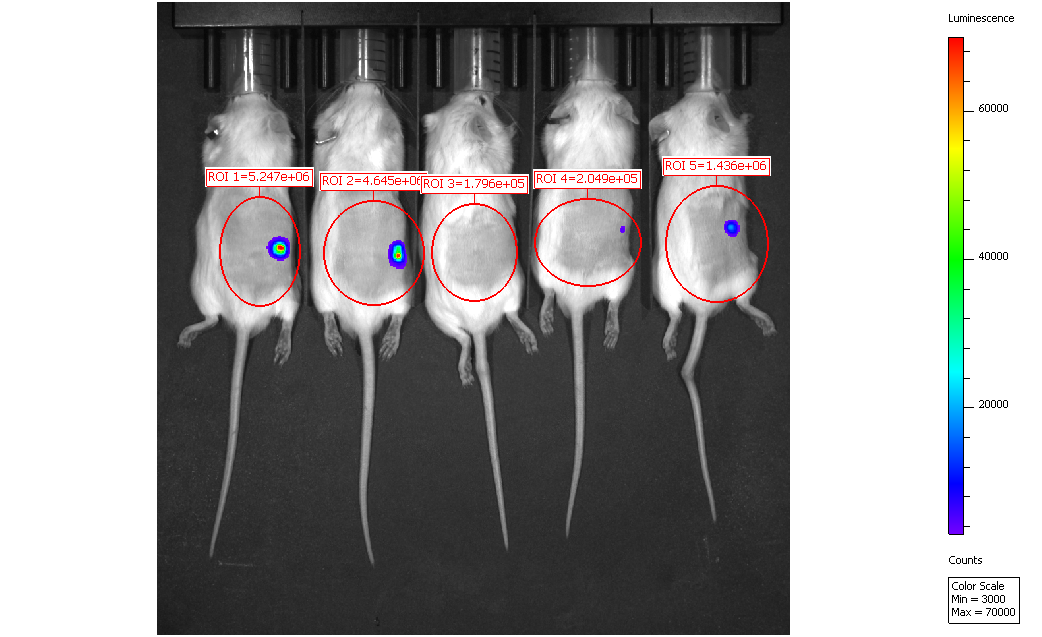

Supplement: Supplementary file 14 — Source data Fig. 8 [file 44321_2026_455_MOESM14_ESM.zip › Figure8/Panel E/DAY 3/1 2 3 4 5-LUC(1).tif]

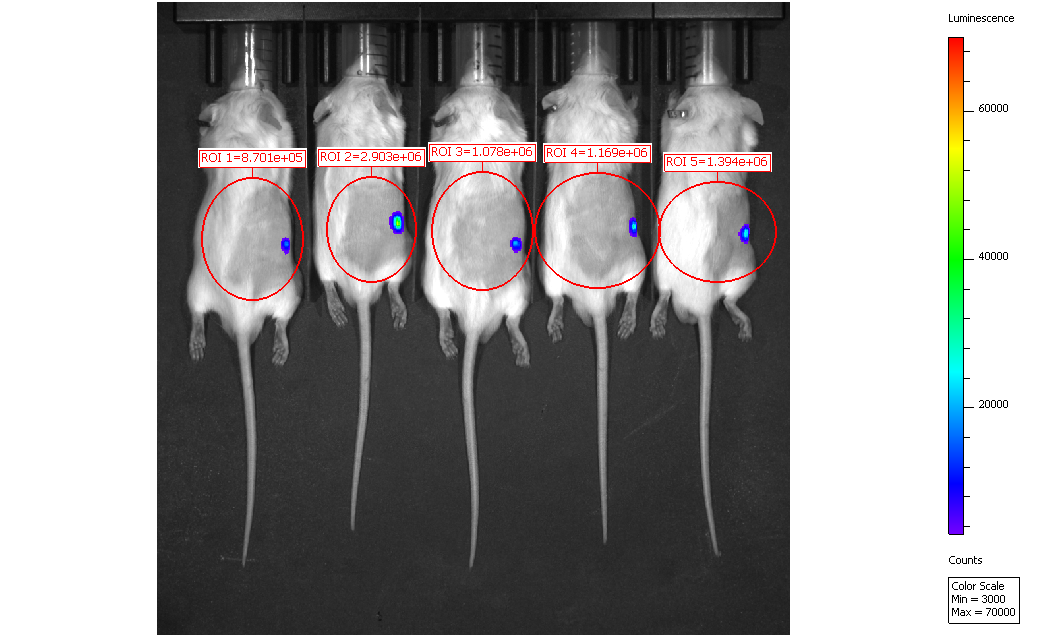

Supplement: Supplementary file 14 — Source data Fig. 8 [file 44321_2026_455_MOESM14_ESM.zip › Figure8/Panel E/DAY 3/11 12 13 14 15 LUC(1).tif]

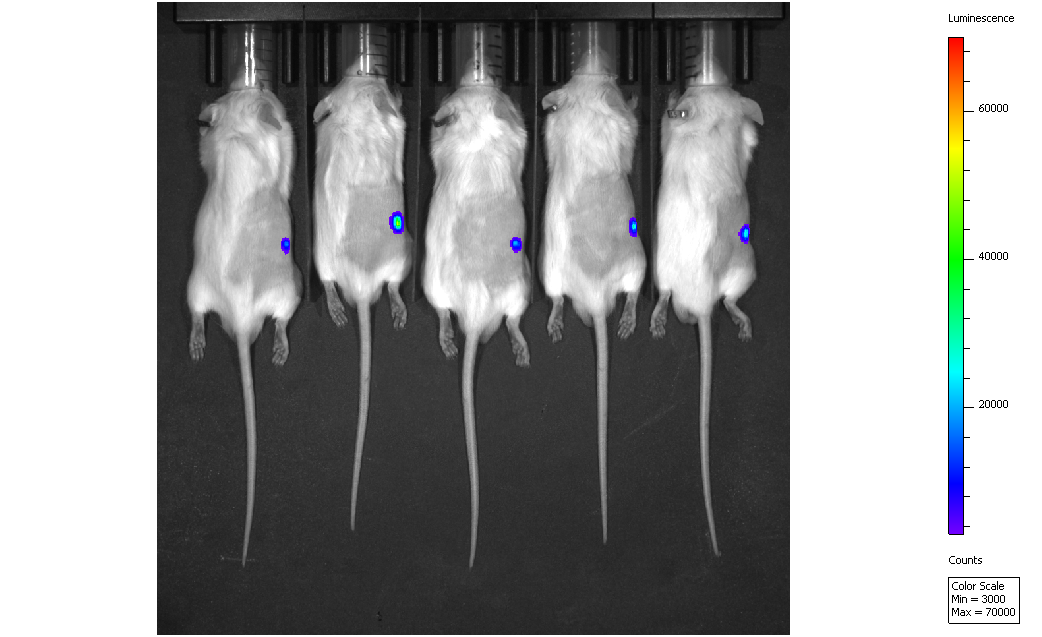

Supplement: Supplementary file 14 — Source data Fig. 8 [file 44321_2026_455_MOESM14_ESM.zip › Figure8/Panel E/DAY 3/11 12 13 14 15(1).tif]

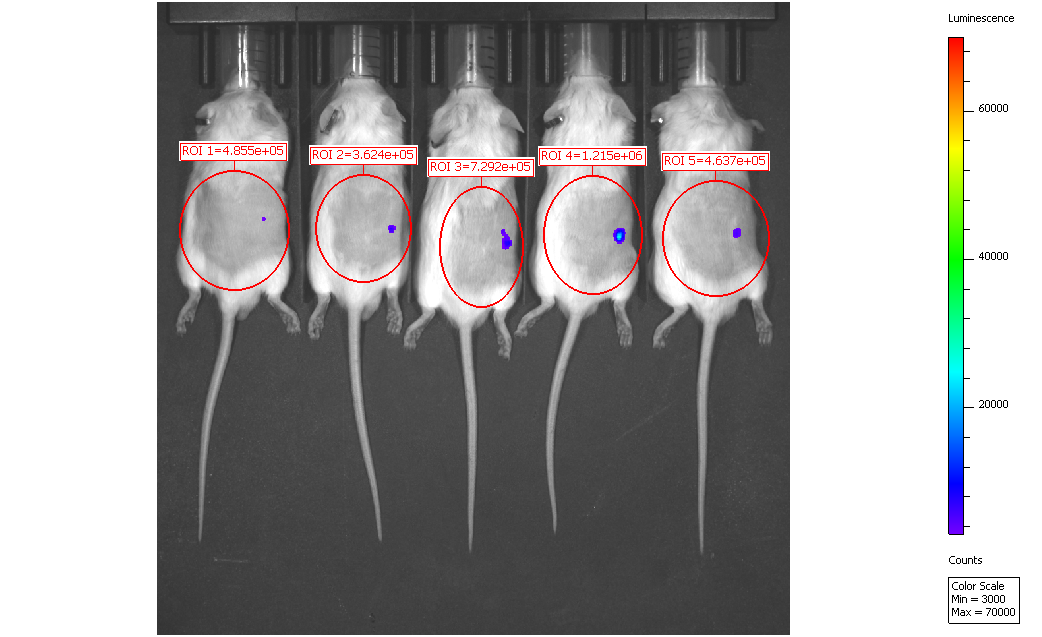

Supplement: Supplementary file 14 — Source data Fig. 8 [file 44321_2026_455_MOESM14_ESM.zip › Figure8/Panel E/DAY 3/16 17 18 19 20 luc(1).tif]

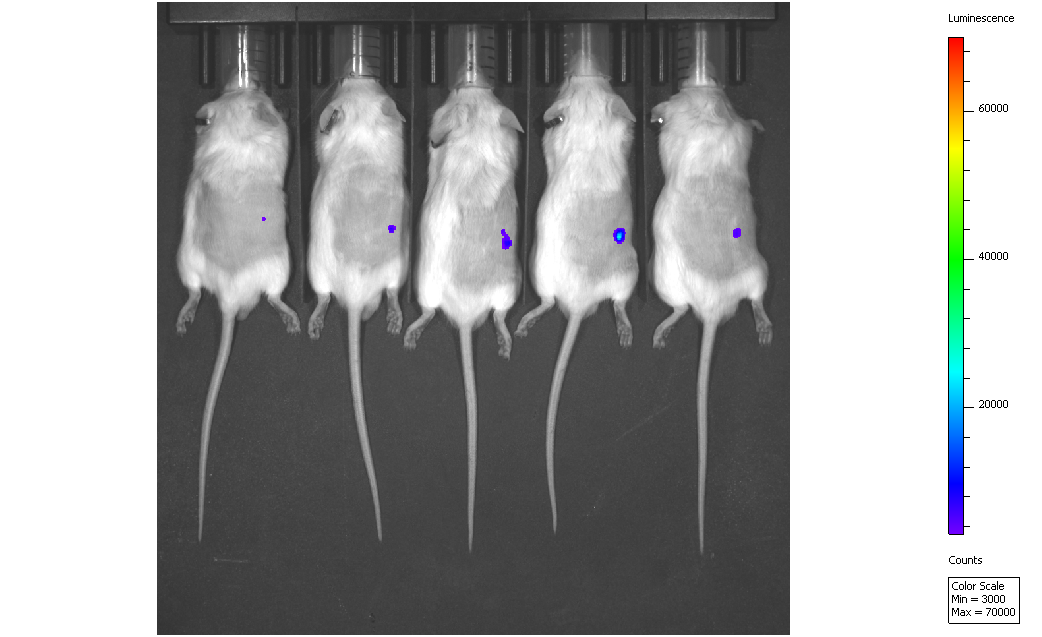

Supplement: Supplementary file 14 — Source data Fig. 8 [file 44321_2026_455_MOESM14_ESM.zip › Figure8/Panel E/DAY 3/16 17 18 19 20(1).tif]

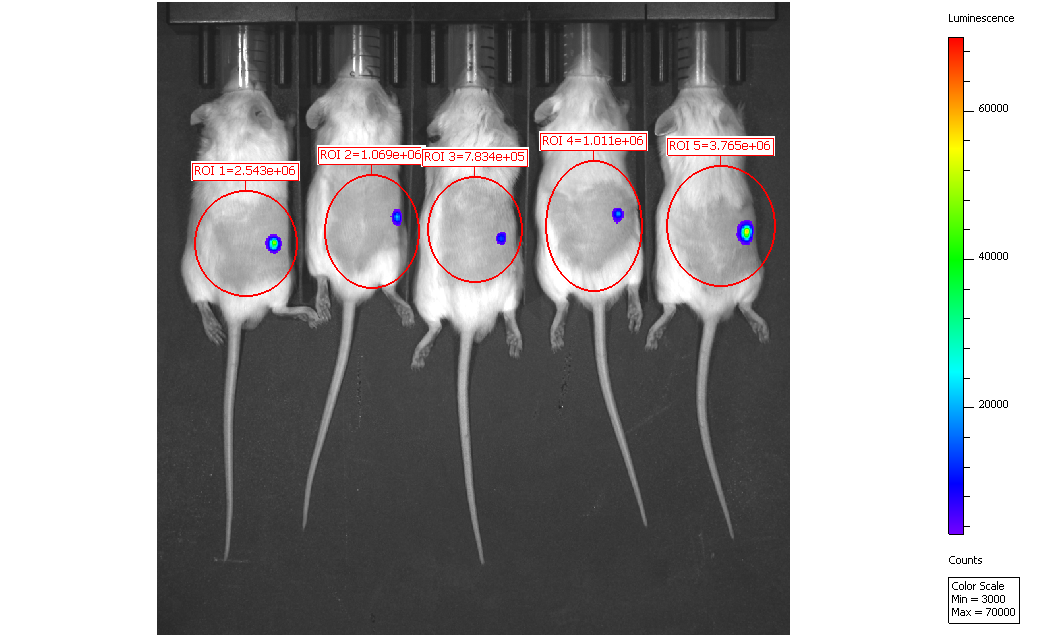

Supplement: Supplementary file 14 — Source data Fig. 8 [file 44321_2026_455_MOESM14_ESM.zip › Figure8/Panel E/DAY 3/21 22 23 24 25 LUC(1).tif]

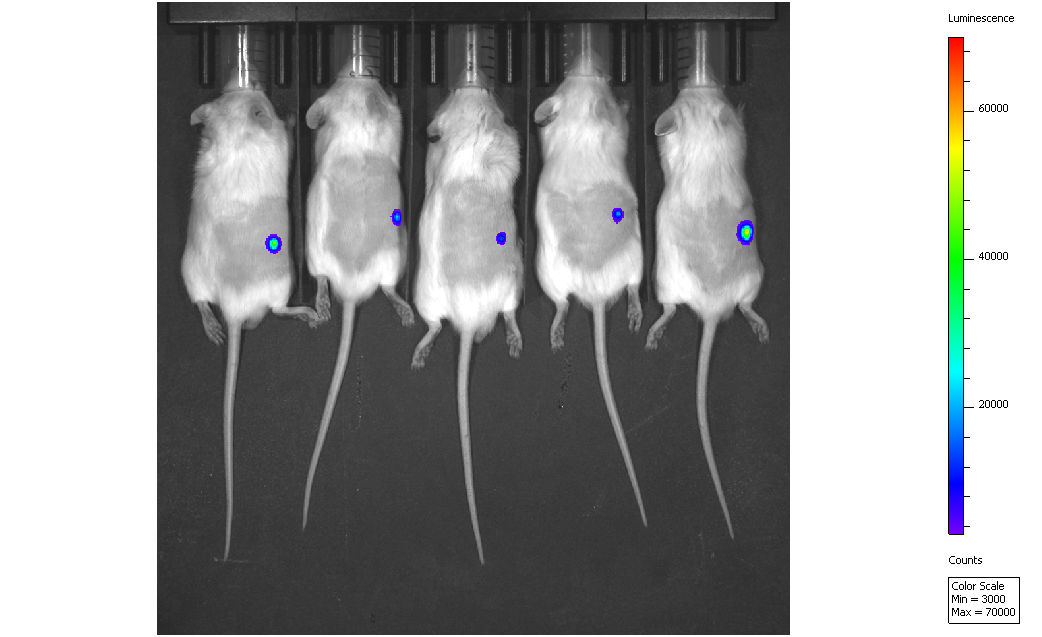

Supplement: Supplementary file 14 — Source data Fig. 8 [file 44321_2026_455_MOESM14_ESM.zip › Figure8/Panel E/DAY 3/21 22 23 24 25(1).tif]

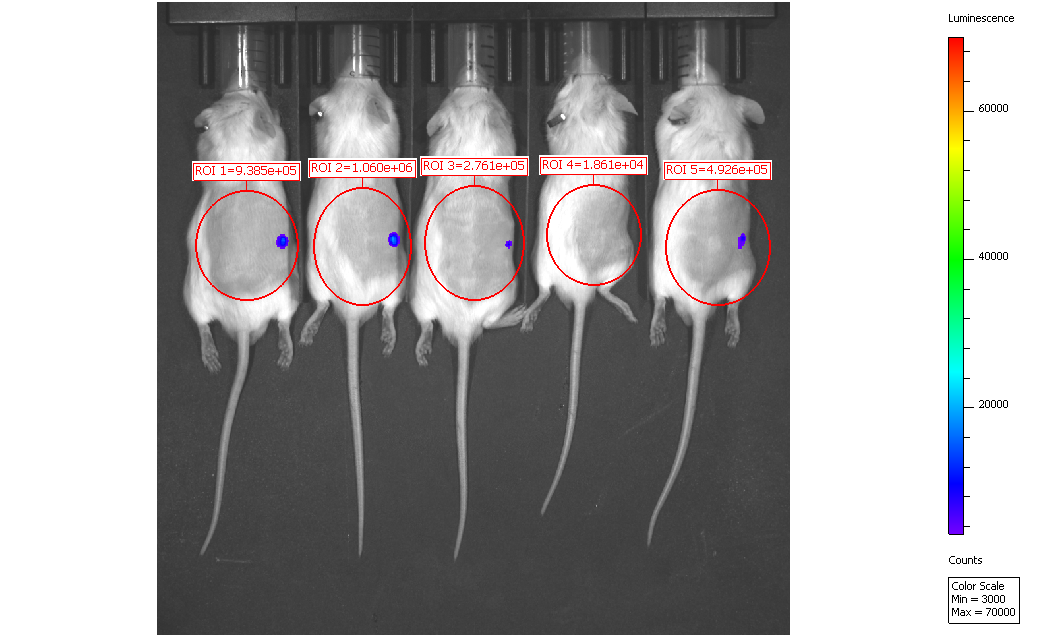

Supplement: Supplementary file 14 — Source data Fig. 8 [file 44321_2026_455_MOESM14_ESM.zip › Figure8/Panel E/DAY 3/26 27 28 29 30 LUC(1).tif]

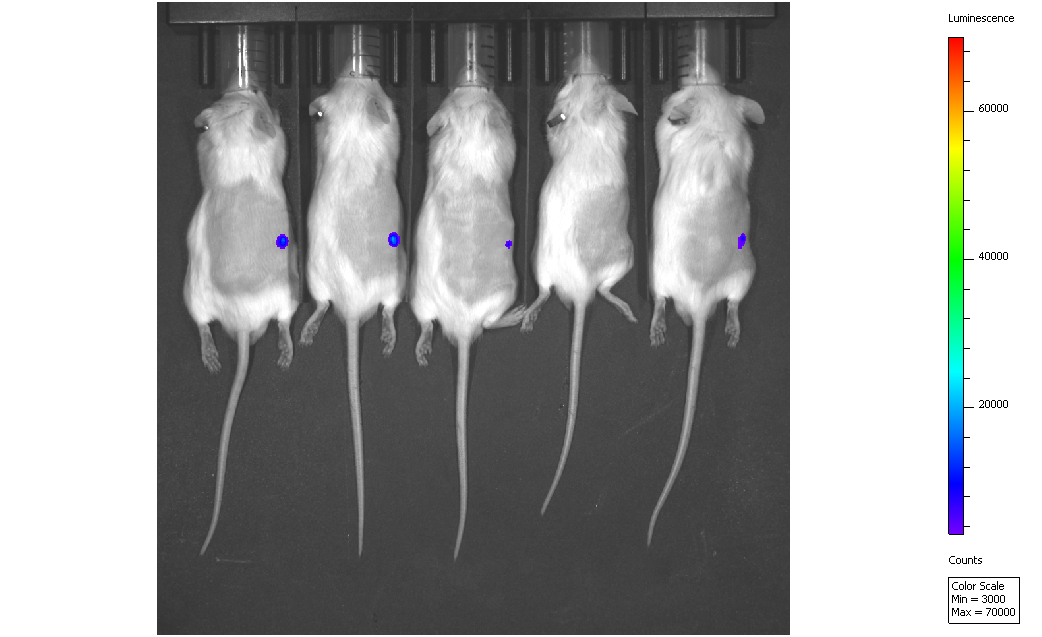

Supplement: Supplementary file 14 — Source data Fig. 8 [file 44321_2026_455_MOESM14_ESM.zip › Figure8/Panel E/DAY 3/26 27 28 29 30(1).tif]

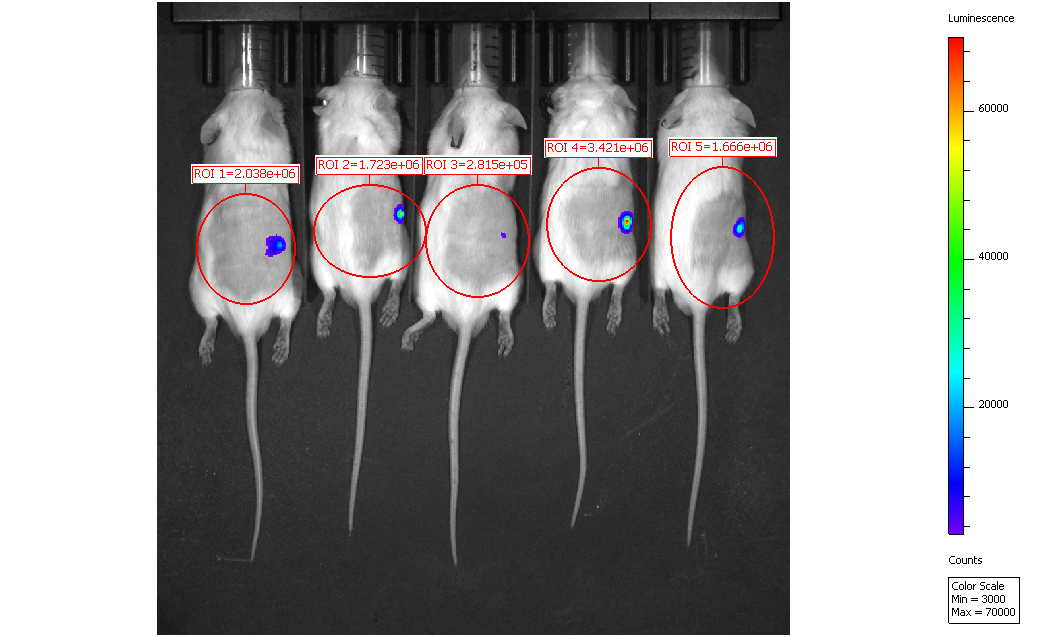

Supplement: Supplementary file 14 — Source data Fig. 8 [file 44321_2026_455_MOESM14_ESM.zip › Figure8/Panel E/DAY 3/31 32 33 34 35 LUC(1).tif]

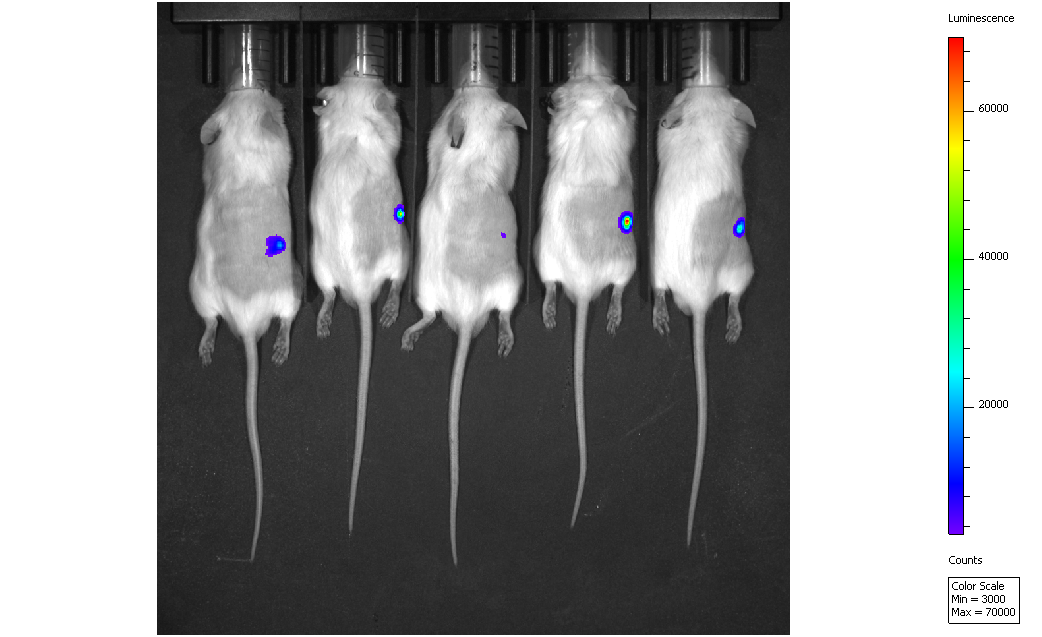

Supplement: Supplementary file 14 — Source data Fig. 8 [file 44321_2026_455_MOESM14_ESM.zip › Figure8/Panel E/DAY 3/31 32 33 34 35(1).tif]

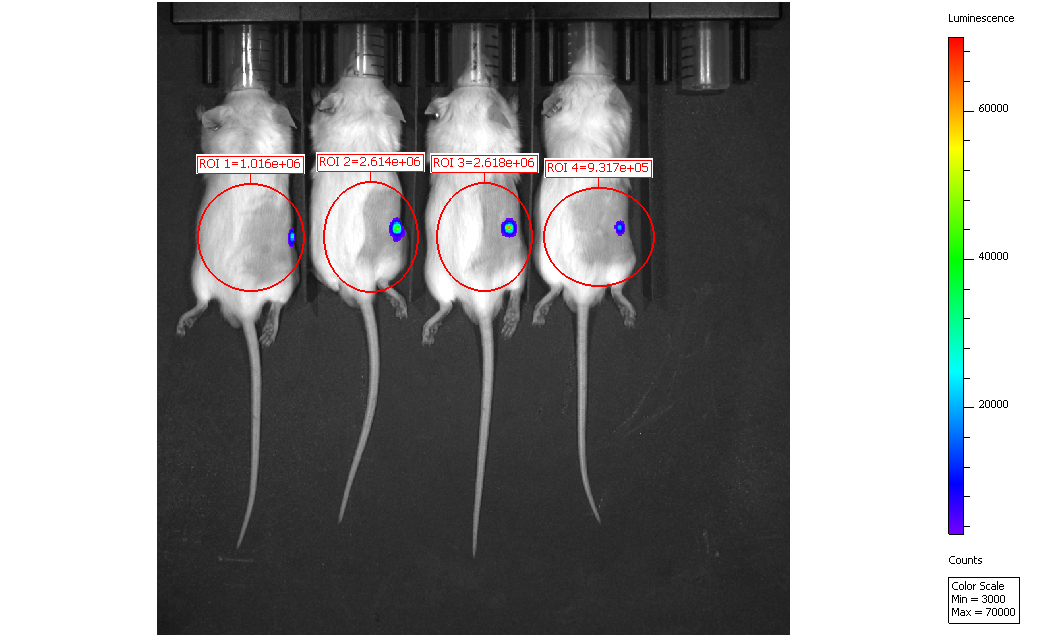

Supplement: Supplementary file 14 — Source data Fig. 8 [file 44321_2026_455_MOESM14_ESM.zip › Figure8/Panel E/DAY 3/36 37 38 39 Luc(1).tif]

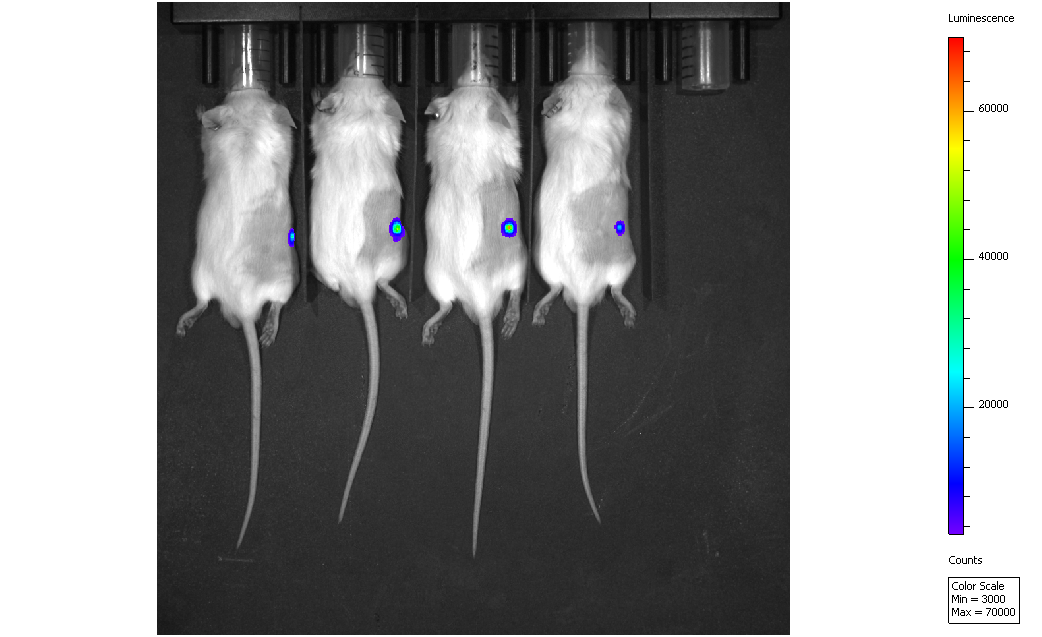

Supplement: Supplementary file 14 — Source data Fig. 8 [file 44321_2026_455_MOESM14_ESM.zip › Figure8/Panel E/DAY 3/36 37 38 39(1).tif]

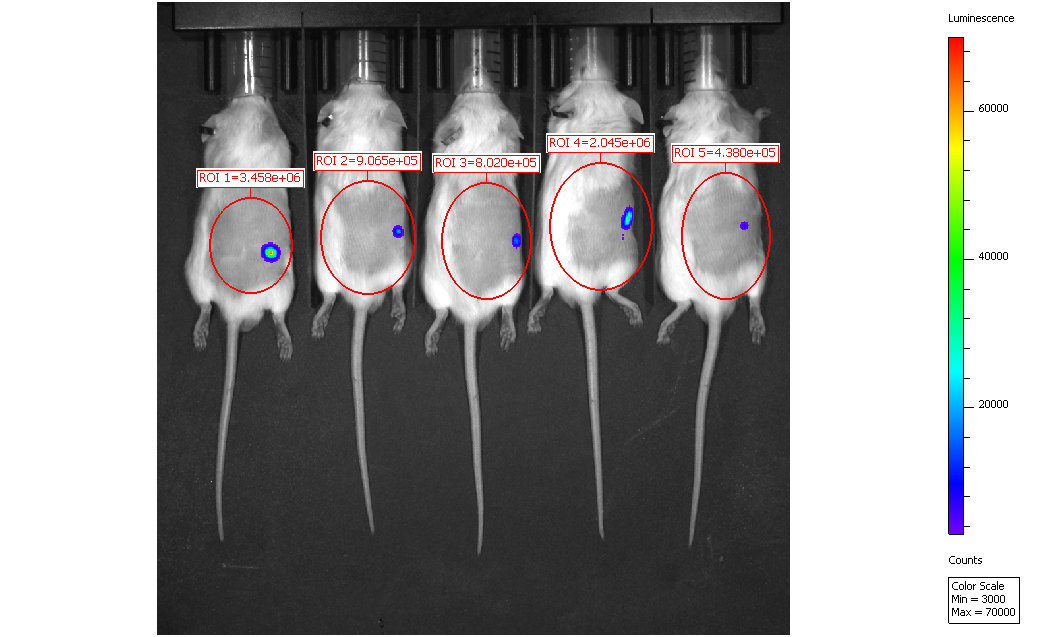

Supplement: Supplementary file 14 — Source data Fig. 8 [file 44321_2026_455_MOESM14_ESM.zip › Figure8/Panel E/DAY 3/6 7 8 9 10 LUC(1).tif]

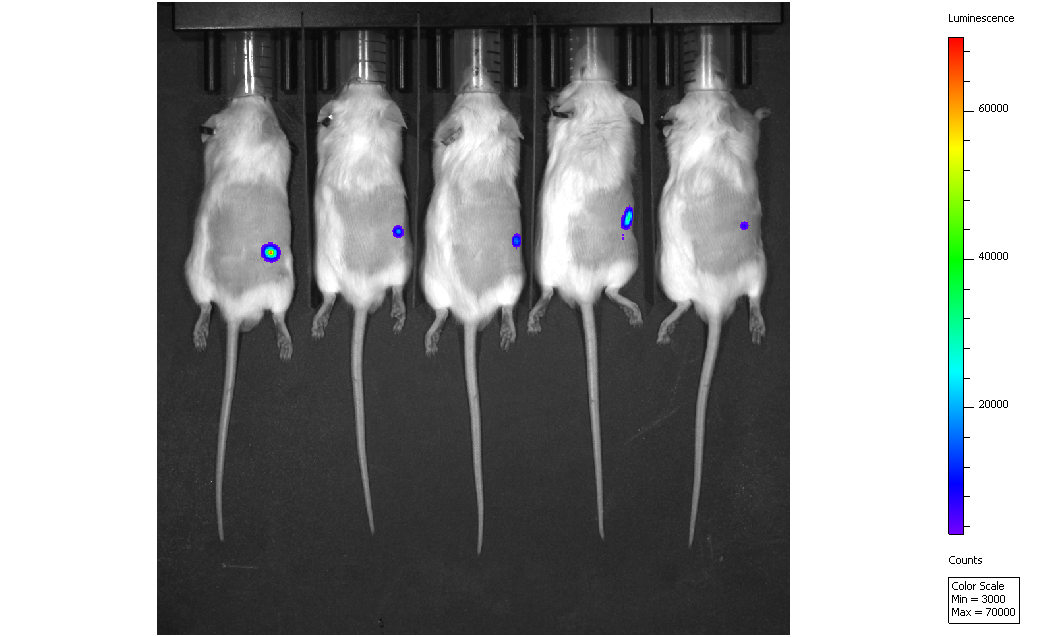

Supplement: Supplementary file 14 — Source data Fig. 8 [file 44321_2026_455_MOESM14_ESM.zip › Figure8/Panel E/DAY 3/6 7 8 9 10(1).tif]

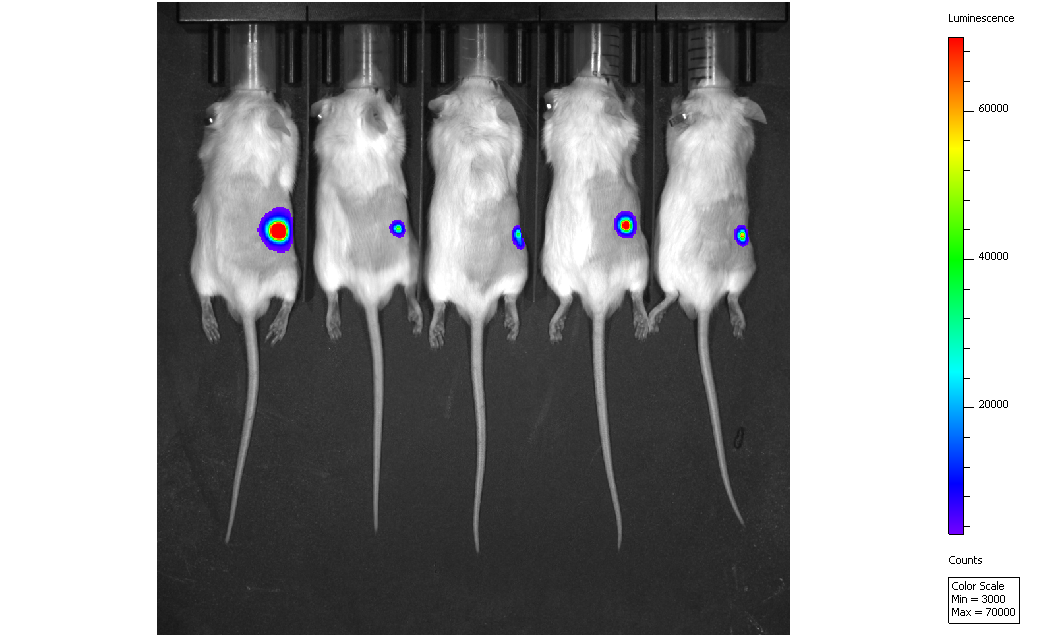

Supplement: Supplementary file 14 — Source data Fig. 8 [file 44321_2026_455_MOESM14_ESM.zip › Figure8/Panel E/DAY 5/1 7 30 38 39.tif]

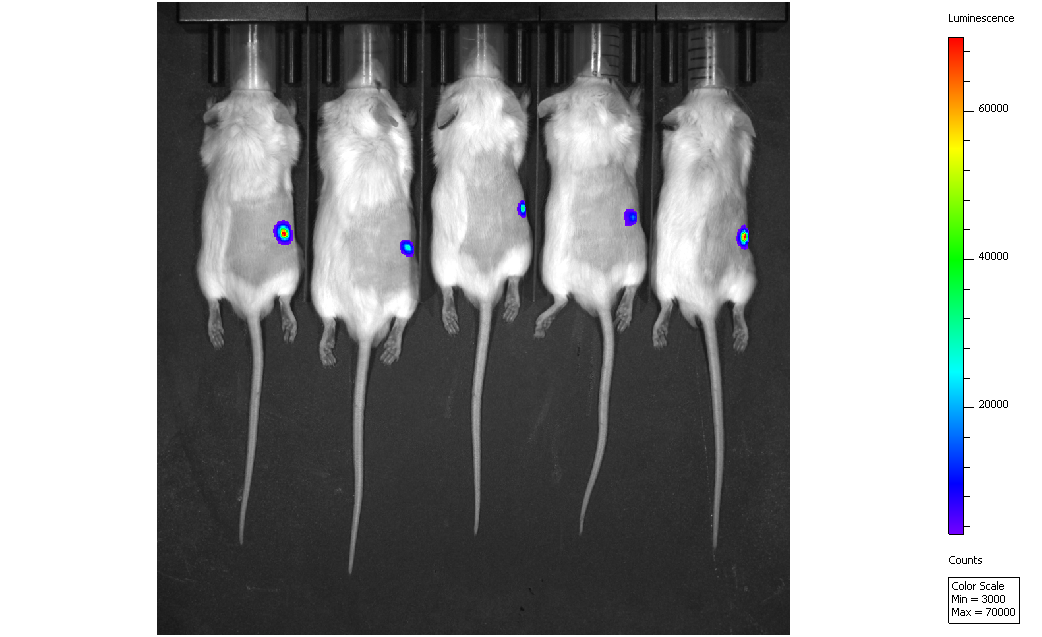

Supplement: Supplementary file 14 — Source data Fig. 8 [file 44321_2026_455_MOESM14_ESM.zip › Figure8/Panel E/DAY 5/12 13 22 31 32.tif]

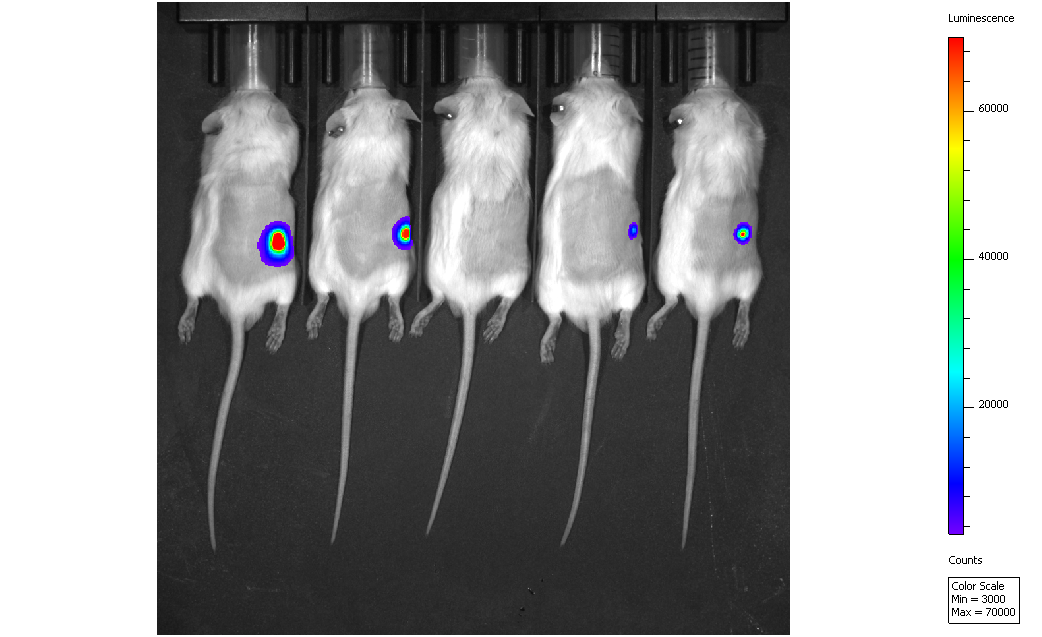

Supplement: Supplementary file 14 — Source data Fig. 8 [file 44321_2026_455_MOESM14_ESM.zip › Figure8/Panel E/DAY 5/2 14 18 26 35.tif]

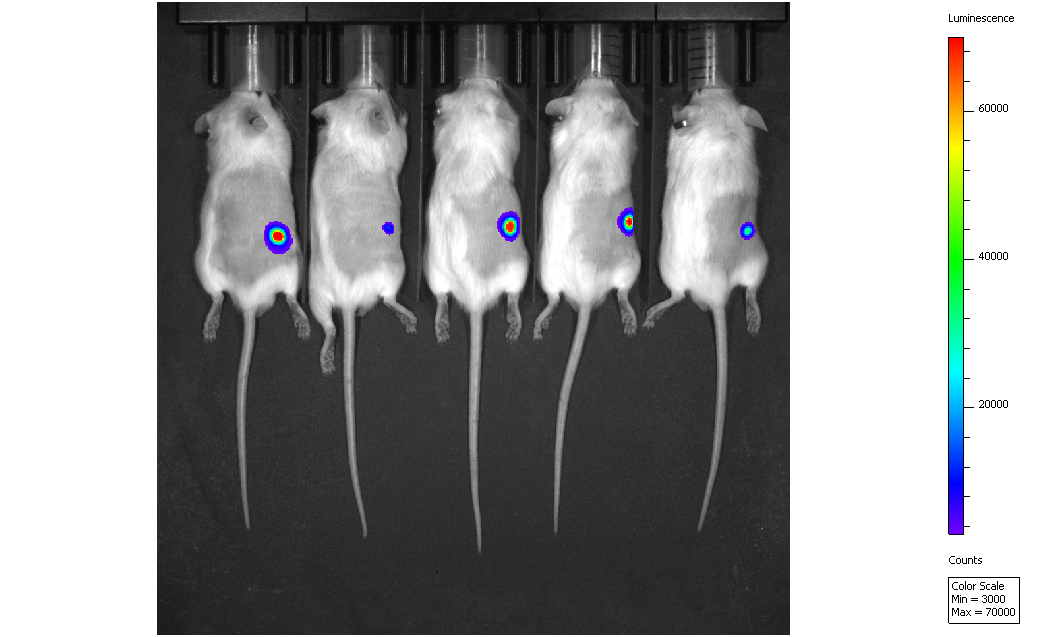

Supplement: Supplementary file 14 — Source data Fig. 8 [file 44321_2026_455_MOESM14_ESM.zip › Figure8/Panel E/DAY 5/6 8 15 36 37.tif]

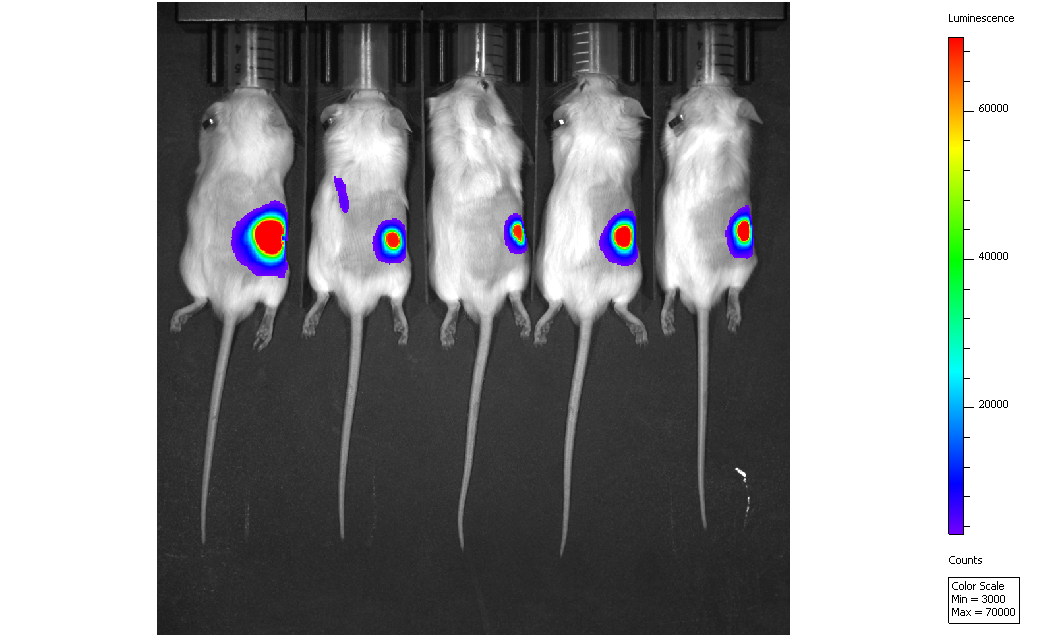

Supplement: Supplementary file 14 — Source data Fig. 8 [file 44321_2026_455_MOESM14_ESM.zip › Figure8/Panel E/DAY 8/1 7 30 38 39.tif]

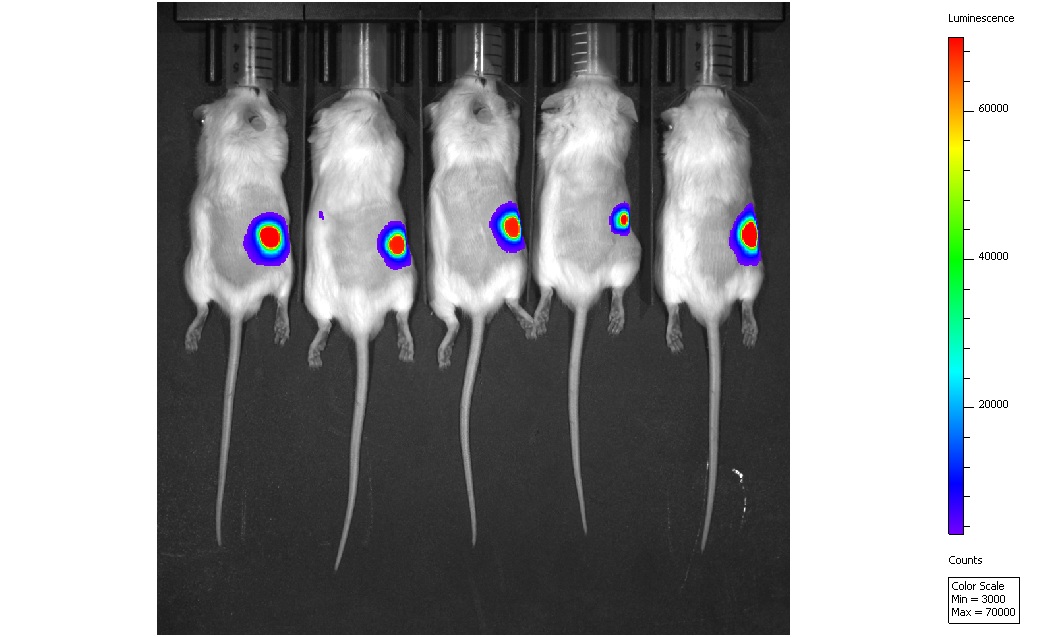

Supplement: Supplementary file 14 — Source data Fig. 8 [file 44321_2026_455_MOESM14_ESM.zip › Figure8/Panel E/DAY 8/12 13 22 31 32.tif]

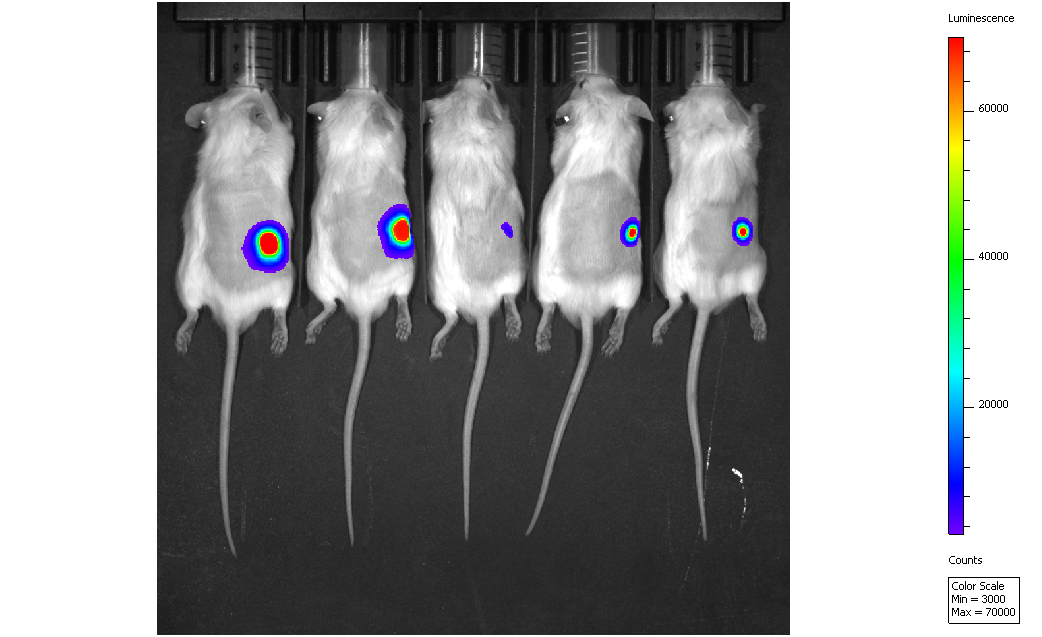

Supplement: Supplementary file 14 — Source data Fig. 8 [file 44321_2026_455_MOESM14_ESM.zip › Figure8/Panel E/DAY 8/2 14 18 26 35.tif]

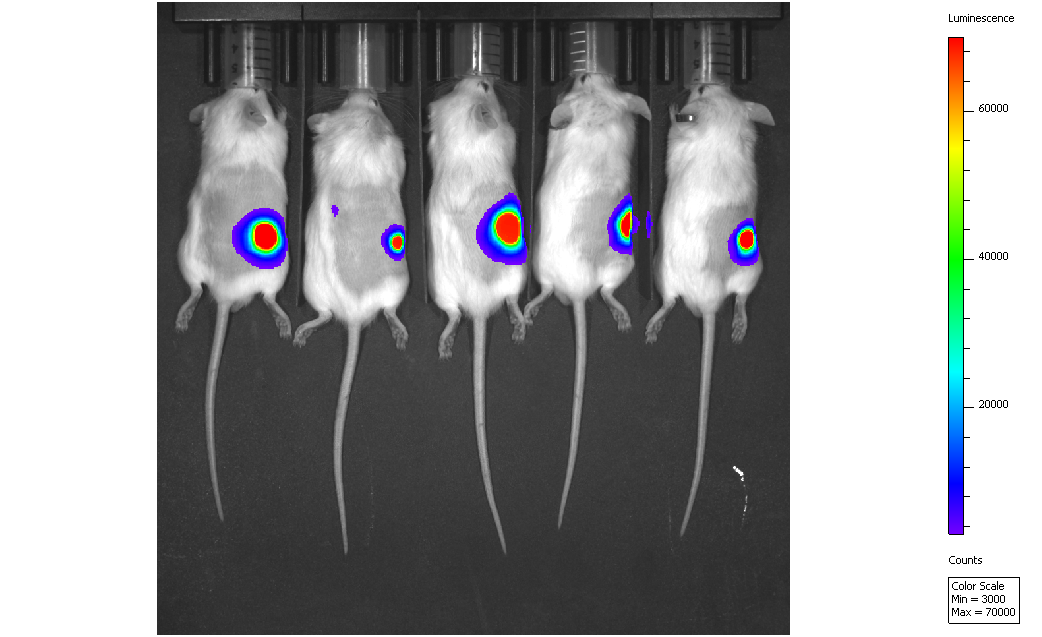

Supplement: Supplementary file 14 — Source data Fig. 8 [file 44321_2026_455_MOESM14_ESM.zip › Figure8/Panel E/DAY 8/6 8 15 36 37.tif]
