# Supplementary material for: Functional screening of TCR-like antibodies using STAR-T cell library for cancer immunotherapy
Source: EMBO Mol Med. 2026 Jun 8;18(7):2748–76. doi: 10.1038/s44321-026-00455-z (PMC13365543; doi:10.1038/s44321-026-00455-z)
Supplement: Supplementary file 15 — Expanded View Figures [file 44321_2026_455_MOESM15_ESM.pdf]

Expanded View Figures

Figure EV1. Supporting characterization of the E-A functional index in STAR-T cells using VHH and scFv formats.

(**A**) Schematic structures of VHH STAR and scFv STAR constructs, each followed by an internal ribosome entry site (IRES) and a red fluorescent protein (RFP). (**B**) Flow cytometry gating for JC5 and K562 cell identification. JC5 cells were identified by RFP expression; K562 cells were identified by GFP expression. (**C**, **E**) Schematics of the co-culture assays of the VHH STAR-JC5 cells and scFv STAR-JC5 cells with swapped target antigens. For the VHH STAR group (**C**), K562 target cells were transduced to express tumor membrane antigen CD123 and co-cultured with JC5 cells transduced with cognate CD123 VHH STAR or non-cognate MSLN VHH STAR control. For scFv STAR group (**E**), K562 target cells were transduced to express tumor membrane antigen GPC3 and co-cultured with JC5 cells transduced with cognate GPC3 scFv STAR or non-cognate CD19 scFv STAR control. Same coloring indicates cognate antigen-antibody pairs. Red arrows represent the endocytosis process. Purple homodimers represent the surface molecule CD69. (**D**, **F**) Flow cytometry analysis of surface STAR (TCRα/β) and CD69 expression on RFP^+ JC5 cells in (**C**, **E**) after 24 h co-culture. (**G**) Kinetics of STAR endocytosis ($TCR\alpha/\beta^-$), CD69 activation ($CD69^+$), and the dual-parameter E-A functional index ($TCR\alpha/\beta^- CD69^+$) in RFP^+ JC5 cells co-cultured with cognate or non-cognate target cells. (**H**) Reversibility of the E-A functional index. STAR endocytosis and CD69 activation were analyzed in RFP^+ JC5 cells at the indicated time points before and after removal of antigen stimulation (via FACS sorting at 48 h). The E:T ratio used in all co-culture experiments is 1:1. Data in (**D**) and (**F**–**H**) are representative of three independent experiments. Data in (**G**) are presented as mean ± SEM.

© The Author(s)

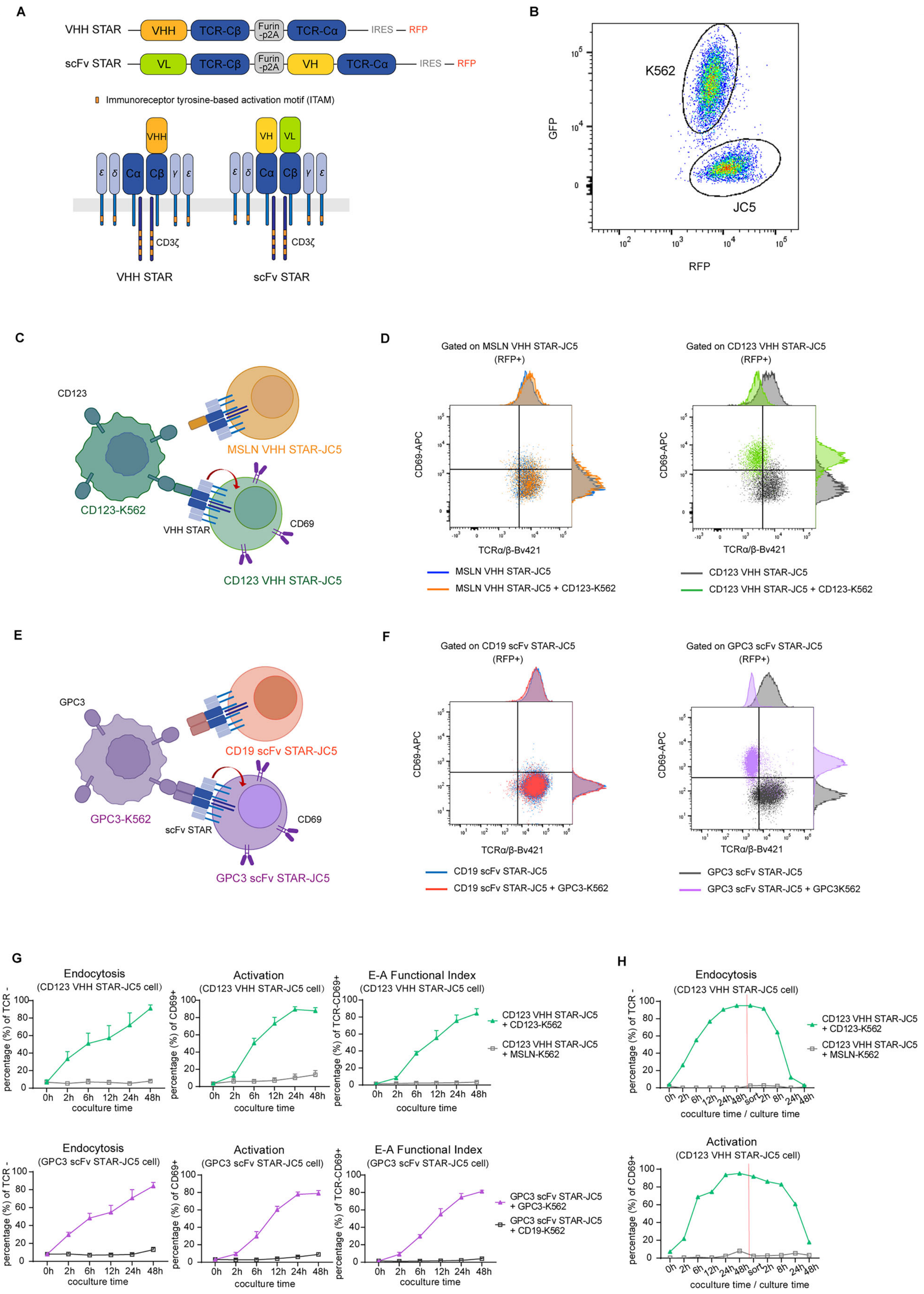

 © The Author(s)

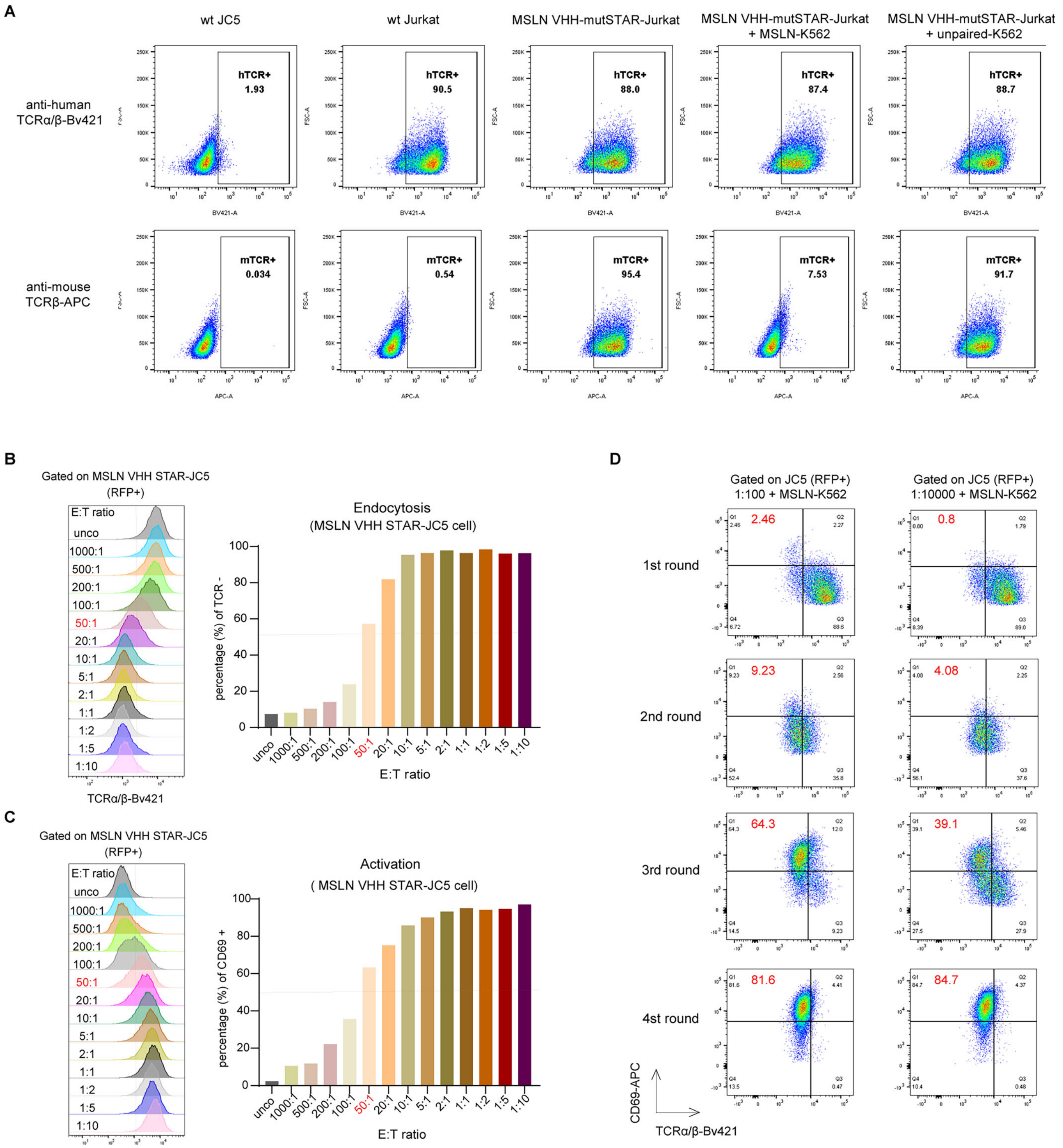

Figure EV2. Sensitivity and specificity of the E-A functional index in STAR-T cells.

(**A**) Flow cytometry analysis of TCR endocytosis in different T cell populations before and after co-culture with target cells. Cells were gated first by FSC for single cells, followed by staining with anti-human TCRα/β-BV421 (endogenous TCR) and anti-mouse TCRβ-APC (mutSTAR). Data are representative of three independent experiments. (**B**, **C**) Flow cytometry analysis of STAR endocytosis and CD69 activation in MSLN VHH STAR-JC5 cells after 24 h co-culture with target MSLN-K562 cells at different E:T ratios. "Unco" indicates JC5 cells cultured without target cells. (**D**) FACS plots of RFP^+ JC5 cells from 1:100 or 1:10,000 model libraries after each round of functional screening against MSLN-K562 cells. Panel D is also shown in Fig. 2B. Data in (**B**, **C**) are representative of three independent experiments.

© The Author(s)

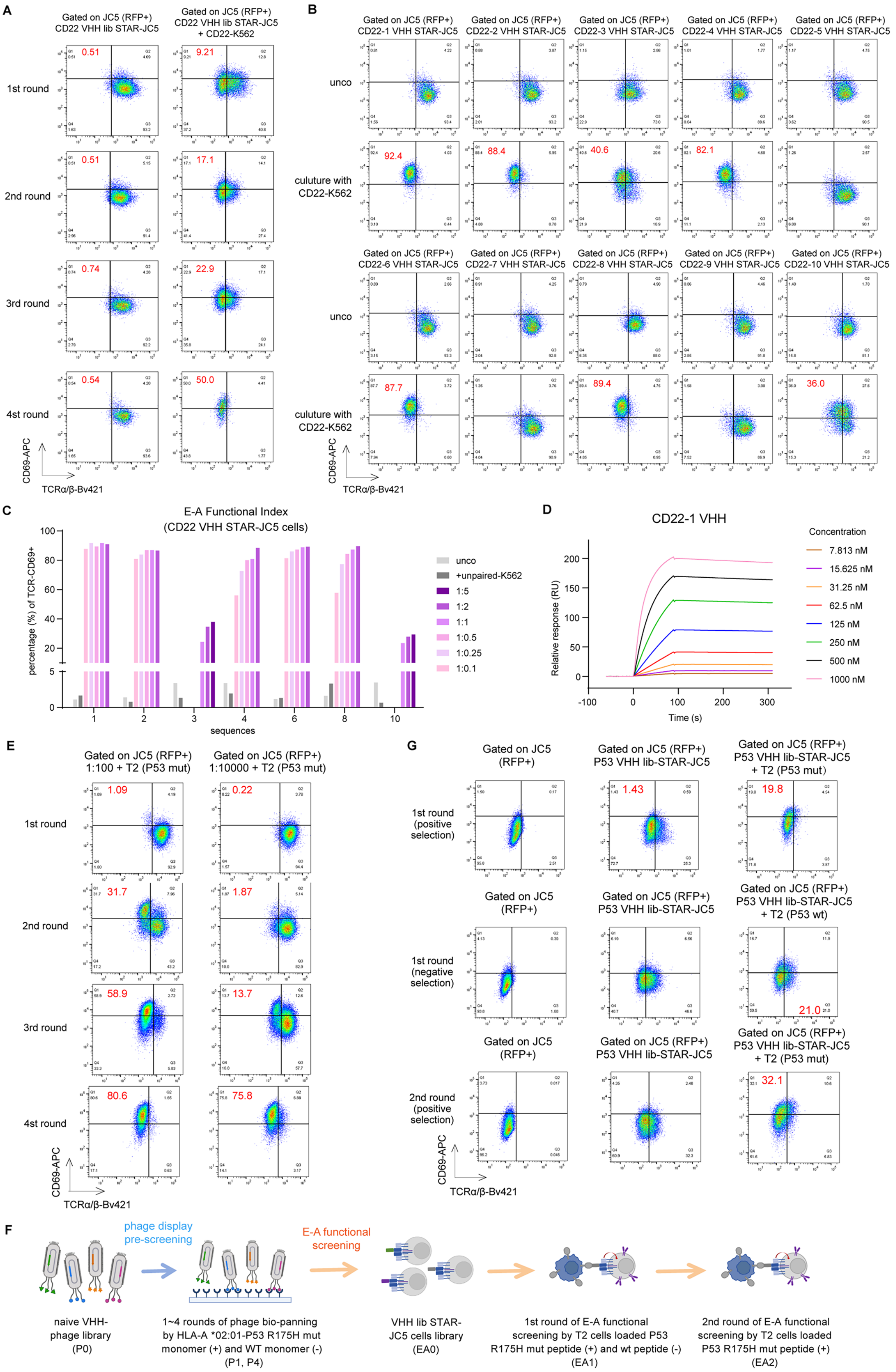
A
Gated on JC5 (RFP+) CD22 VHH lib STAR-JC5
Gated on JC5 (RFP+) CD22 VHH lib STAR-JC5 + CD22-K562
1st round
2nd round
3rd round
4st round
CD69-APC
TCRα/β-Bv421
B
Gated on JC5 (RFP+) CD22-1 VHH STAR-JC5
Gated on JC5 (RFP+) CD22-2 VHH STAR-JC5
Gated on JC5 (RFP+) CD22-3 VHH STAR-JC5
Gated on JC5 (RFP+) CD22-4 VHH STAR-JC5
Gated on JC5 (RFP+) CD22-5 VHH STAR-JC5
Gated on JC5 (RFP+) CD22-6 VHH STAR-JC5
Gated on JC5 (RFP+) CD22-7 VHH STAR-JC5
Gated on JC5 (RFP+) CD22-8 VHH STAR-JC5
Gated on JC5 (RFP+) CD22-9 VHH STAR-JC5
Gated on JC5 (RFP+) CD22-10 VHH STAR-JC5
unco
culuture with CD22-K562
C
E-A Functional Index (CD22 VHH STAR-JC5 cells)
percentage (%) of TCR-CD69+
sequences
unco
+unpaired-K562
1:5
1:2
1:1
1:0.5
1:0.25
1:0.1
D
CD22-1 VHH
Relative response (RU)
Time (s)
Concentration
7.813 nM
15.625 nM
31.25 nM
62.5 nM
125 nM
250 nM
500 nM
1000 nM
E
Gated on JC5 (RFP+) 1:100 + T2 (P53 mut)
Gated on JC5 (RFP+) 1:10000 + T2 (P53 mut)
1st round
2nd round
3rd round
4st round
CD69-APC
TCRα/β-Bv421
G
Gated on JC5 (RFP+)
Gated on JC5 (RFP+) P53 VHH lib-STAR-JC5
Gated on JC5 (RFP+) P53 VHH lib-STAR-JC5 + T2 (P53 mut)
Gated on JC5 (RFP+) P53 VHH lib-STAR-JC5 + T2 (P53 wt)
1st round (positive selection)
1st round (negative selection)
2nd round (positive selection)
CD69-APC
TCRα/β-Bv421
F
phage display pre-screening
E-A functional screening
naive VHH-phage library (P0)
1~4 rounds of phage bio-panning by HLA-A *02:01-P53 R175H mut monomer (+) and WT monomer (-) (P1, P4)
VHH lib STAR-JC5 cells library (EA0)
1st round of E-A functional screening by T2 cells loaded P53 R175H mut peptide (+) and wt peptide (-) (EA1)
2nd round of E-A functional screening by T2 cells loaded P53 R175H mut peptide (+) (EA2)

 © The Author(s)

Figure EV3. Supporting characterization of the E-A functional screening process for CD22 and P53^{R175H} VHHs.

(**A**) FACS analysis of RFP^{+} JC5 cells from the CD22 VHH lib STAR-JC5 cell library after each round of functional screening before and after co-culture with target CD22-K562 cells (E:T = 1:1). (**B**) FACS analysis of the top ten enriched CD22 VHHs expressed in STAR-JC5 cells before and after co-culture with target CD22-K562 cells (E:T = 1:1). (**C**) Flow cytometry analysis of seven E-A functional index-positive CD22 VHHs-based STAR-JC5 cells before and after co-culture with target CD22-K562 cells at different E:T ratios or with unpaired K562 cells as a negative control (The results of CD22-3 and CD22-10 groups at E:T ratios of 1:0.5, 1:0.25, and 1:0.1 are not involved in this experiment). (**D**) Surface plasmon resonance (SPR) binding analysis of CD22-1 VHH (human IgG1-Fc dimer) binding to human CD22. Sensorgrams show responses from multi-cycle kinetics at increasing analyte concentrations. All co-culture experiments were performed for 24 h. Data are representative of three independent experiments. (**E**) FACS analysis of RFP^{+} JC5 cells from 1:100 or 1:10,000 model libraries after each round of functional screening against T2 cells loaded with 10 µM P53 R175H mutant (mut) peptide. (**F**) Screening scheme for the P53 VHH lib STAR-JC5 library. "+" indicates positive selection using P53 R175H mut peptide; "–" indicates negative selection using P53 wild-type (wt) peptide. Labels in parentheses (P0, P1, P4, EA0, EA1, and EA2) denote samples subjected to NGS analysis, where "P" indicates phage library samples and "EA" indicates E-A functional screening cell library samples, with numbers indicating the initial library (0) or screening round. (**G**) FACS analysis of gated P53 VHH lib STAR-JC5 cells after each round of functional screening. Round 1 used T2 cells loaded with 1 µM P53 R175H peptide for positive selection and 1 µM P53 wt peptide for negative selection; round 2 used T2 cells loaded with 1 µM P53 R175H peptide for positive selection.

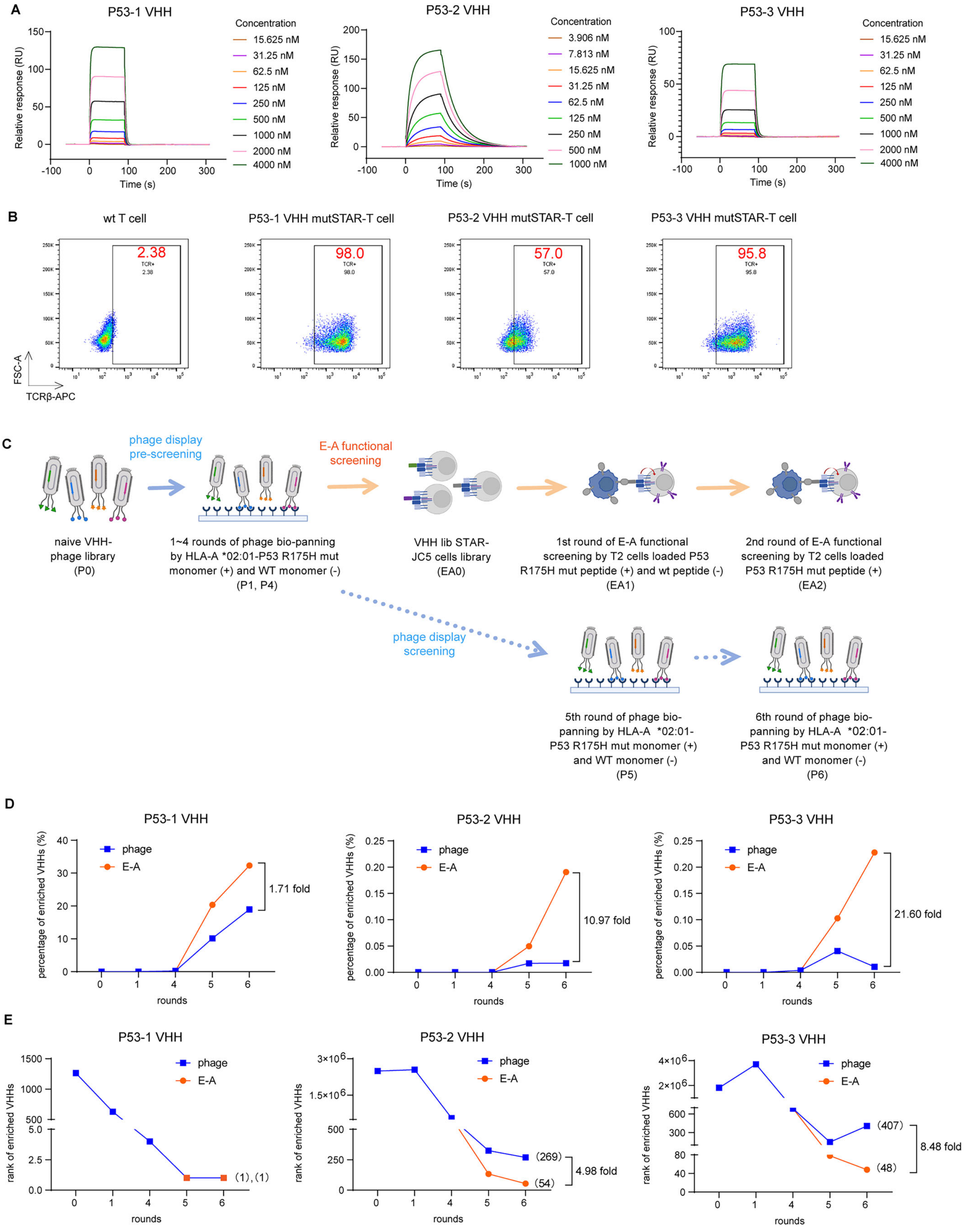
A
P53-1 VHH
Relative response (RU)
Time (s)
Concentration
15.625 nM
31.25 nM
62.5 nM
125 nM
250 nM
500 nM
1000 nM
2000 nM
4000 nM
P53-2 VHH
Concentration
3.906 nM
7.813 nM
15.625 nM
31.25 nM
62.5 nM
125 nM
250 nM
500 nM
1000 nM
P53-3 VHH
Concentration
15.625 nM
31.25 nM
62.5 nM
125 nM
250 nM
500 nM
1000 nM
2000 nM
4000 nM
B
wt T cell
P53-1 VHH mutSTAR-T cell
P53-2 VHH mutSTAR-T cell
P53-3 VHH mutSTAR-T cell
2.38
98.0
57.0
95.8
FSC-A
TCRβ-APC
C
phage display pre-screening
E-A functional screening
naive VHH-phage library (P0)
1~4 rounds of phage bio-panning by HLA-A *02:01-P53 R175H mut monomer (+) and WT monomer (-) (P1, P4)
VHH lib STAR-JC5 cells library (EA0)
1st round of E-A functional screening by T2 cells loaded P53 R175H mut peptide (+) and wt peptide (-) (EA1)
2nd round of E-A functional screening by T2 cells loaded P53 R175H mut peptide (+) (EA2)
phage display screening
5th round of phage bio-panning by HLA-A *02:01-P53 R175H mut monomer (+) and WT monomer (-) (P5)
6th round of phage bio-panning by HLA-A *02:01-P53 R175H mut monomer (+) and WT monomer (-) (P6)
D
P53-1 VHH
P53-2 VHH
P53-3 VHH
percentage of enriched VHHs (%)
phage
E-A
rounds
1.71 fold
10.97 fold
21.60 fold
E
P53-1 VHH
P53-2 VHH
P53-3 VHH
rank of enriched VHHs
phage
E-A
rounds
(1), (1)
(269)
(54)
4.98 fold
(407)
(48)
8.48 fold

 © The Author(s)

Figure EV4. Characterization of P53^{R175H}-specific VHHs and comparison of screening strategies (E-A index versus phage display).

(**A**) SPR binding analysis of three P53 VHHs (human IgG1-Fc dimers) to immobilized P53 R175H-HLA-A*02:01 monomer. Sensorgrams show binding responses from multi-cycle kinetics at increasing analyte concentrations. Data are representative of three independent experiments. (**B**) Flow cytometry analysis of surface expression of different P53 VHH mutSTAR constructs on primary human T cells. Cells were gated by FSC for single cells, and mutSTAR expression was detected using anti-mouse TCRβ-APC staining. (**C**) Schematic of the parallel screening strategy. Following four pre-screening rounds of phage display, two additional rounds of phage screening (P5, P6) were performed concurrently with two rounds of E-A functional screening of the converted cell library (EA1 and EA2). "+" indicates positive selection using P53 R175H mut peptide; "–" indicates negative selection using P53 wt peptide. Labels in parentheses (P0, P1, P4, P5, P6, EA0, EA1, and EA2) denote samples subjected to NGS analysis, where "P" indicates phage library samples and "EA" indicates E-A functional screening cell library samples, with numbers indicating the initial library (0) or screening round. (**D**) The relative amino acid sequence abundance (%) of functional antibodies P53-1, -2, and -3 at key stages of the screening process. (**E**) The rank positions of functional antibodies P53-1, -2, and -3 within the library across selection rounds. X-axis labels in (b, c): "0" indicates the initial naïve VHH library; "1" and "4" indicate phage pre-screening rounds 1 and 4; "5" indicates E-A cell library screening round 1 (EA1) and phage screening round 5 (P5); "6" indicates E-A cell library screening round 2 (EA2) and phage screening round 6 (P6). Fold-enrichment values compare the final output ratios (EA2 vs P6) between the two methods.

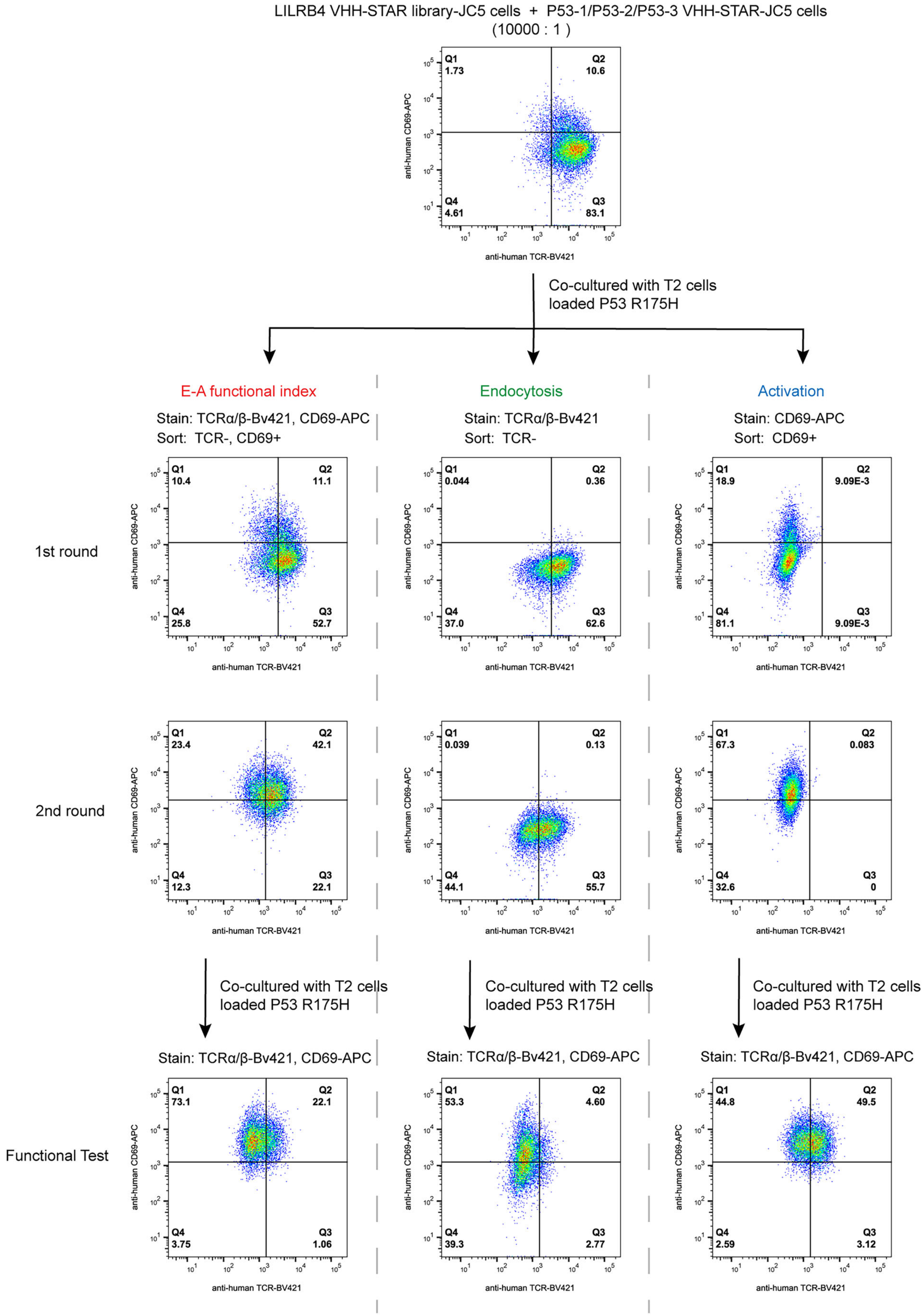
LILRB4 VHH-STAR library-JC5 cells + P53-1/P53-2/P53-3 VHH-STAR-JC5 cells
(10000 : 1)
Q1 1.73
Q2 10.6
Q4 4.61
Q3 83.1
anti-human CD69-APC
anti-human TCR-BV421
Co-cultured with T2 cells loaded P53 R175H
E-A functional index
Stain: TCRα/β-Bv421, CD69-APC
Sort: TCR-, CD69+
Endocytosis
Stain: TCRα/β-Bv421
Sort: TCR-
Activation
Stain: CD69-APC
Sort: CD69+
1st round
Q1 10.4
Q2 11.1
Q4 25.8
Q3 52.7
Q1 0.044
Q2 0.36
Q4 37.0
Q3 62.6
Q1 18.9
Q2 9.09E-3
Q4 81.1
Q3 9.09E-3
2nd round
Q1 23.4
Q2 42.1
Q4 12.3
Q3 22.1
Q1 0.039
Q2 0.13
Q4 44.1
Q3 55.7
Q1 67.3
Q2 0.083
Q4 32.6
Q3 0
Co-cultured with T2 cells loaded P53 R175H
Stain: TCRα/β-Bv421, CD69-APC
Functional Test
Q1 73.1
Q2 22.1
Q4 3.75
Q3 1.06
Q1 53.3
Q2 4.60
Q4 39.3
Q3 2.77
Q1 44.8
Q2 49.5
Q4 2.59
Q3 3.12

 © The Author(s)

Figure EV5. Screening workflow for evaluating different selection strategies.

A LILRB4-specific VHH STAR-JC5 cell library, generated from immunized alpaca and spiked with known functional clones (P53-1, -2, -3) at a 1:10,000 dilution, was co-cultured with P53 R175H mut peptide-loaded T2 cells and subjected to two rounds of selection using either the dual-parameter E-A functional index ($TCR^{-}CD69^{+}$), TCR^{-} only, or $CD69^{+}$ only strategies. Enriched populations were analyzed by flow cytometry for TCR endocytosis and CD69 expression after stimulation.

© The Author(s)
