# Supplementary material for: Functional screening of TCR-like antibodies using STAR-T cell library for cancer immunotherapy
Source: EMBO Mol Med. 2026 Jun 8;18(7):2748–76. doi: 10.1038/s44321-026-00455-z (PMC13365543; doi:10.1038/s44321-026-00455-z)
Supplement: Supplementary file 6 — Peer Review File [file 44321_2026_455_MOESM6_ESM.pdf]

EMBO Molecular Medicine

Functional screening of TCR-like antibodies using STAR-T cell library for cancer immunotherapy

Xin Lin, Yi Li, Daosheng Huang, Chang Liu, Zhixiao Zhou, Yilu Song, Dongmei Yang, Wei Rui, Zaopeng Yang, Li Yu, Chenliang Wang, Zheyu Zheng, Jiasheng Wang, and Yang-Xin Fu

Corresponding author(s): Xin Lin (linxin307@tsinghua.edu.cn)

Review Timeline:

Submission Date:	10th Dec 25
Editorial Decision:	28th Jan 26
Revision Received:	9th Apr 26
Editorial Decision:	22nd Apr 26
Revision Received:	7th May 26
Accepted:	13th May 26

Editor: Lise Roth

Transaction Report: This manuscript was submitted to EMBO Molecular Medicine after peer review at another journal.

(Note: With the exception of the correction of typographical or spelling errors that could be a source of ambiguity, letters and reports are not edited. Depending on transfer agreements, referee reports obtained elsewhere may or may not be included in this compilation. Referee reports are anonymous unless the Referee chooses to sign their reports.)

Reviewer #1

Comments on the Revised Manuscript:

The manuscript has improved substantially. The new data would address my concerns satisfactorily if the authors can explain the following discrepancy:

In Revised Suppl. Table 2, the frequencies of the P53 clones at EA0 in Table 2.2 should be similar to those at P4 in Table 2.1 as the EA0 cell library was derived directly from the P4 phage. However, the frequencies of the clones at EA0 are at least 27 fold (P53-3) higher than those at P4.

An additional observation is that phage-based panning often fails to further enrich binders (e.g. P53-2 VHH and P53-3 VHH in Suppl. Fig. 8) after around 5 for unknown reasons (perhaps due to outgrowth of non-specific phage clones with growth advantage). The E-A functional screening seems to help overcome this limitation and thus could be particularly useful for identifying rare binders that require more rounds of enrichment.

Response:

We sincerely appreciate your supportive feedback and the insightful comments on our revised manuscript.

Firstly, we appreciate you raising the point regarding the data inconsistency "the frequencies of the P53 clones at EA0 shown in Revised Suppl. Table 2.2 are significantly higher than those at P4 in Table 2.1", as it draws attention to a significant discrepancy that requires explanation.

As noted in our manuscript (lines 247–252:"Strikingly, the conversion of the phage library into the STAR-JC5 format dramatically reshaped the library landscape (Supplementary Table 1.2). This process alone substantially reduced the library complexity, with the Chao1 index plummeting by ~99.6% to 1.02 × 10^6 and S obs number falling by ~99.3% to 3.14 × 10^5. Most importantly, it led to a remarkable enrichment, as evidenced by the Top100 sequence coverage soaring to 59.37%—representing an increase of over 10-fold compared to the pre-conversion library."), this discrepancy stems primarily from the substantial library reshaping that occurs during the conversion from phage to cell-based display, which we further explained in lines 252–256:"We attribute this effective library enrichment primarily to the stringent selection imposed by eukaryotic expression, which filters out VHH clones incapable of proper folding, post-translational modification, or membrane localization in mammalian cells, thereby efficiently enriching for therapeutically compatible candidates."

Thus, we conclude that the conversion from phage to eukaryotic cell-display library selectively enriches dominant clones which are competent in mammalian cell folding, post-translational modifications, and membrane localization, thereby substantially elevating their frequency in the final library. If you consider it necessary, we can add a dedicated discussion on this point in the main text or discussion section, noting that

although absolute frequency comparisons between phage and cell-display systems should be interpreted with care, the relative enrichment profiles provide meaningful biological insights.

Secondly, your observation regarding the inherent limitations of phage-based panning is particularly astute and directly corresponds with our experimental findings. Indeed, we found that the E-A screening platform offers a crucial advantage in later enrichment rounds, as it circumvents the issue of non-specific clone overgrowth that often hampers phage systems. We will cite your perspective in the discussion section to specifically acknowledge this point, highlighting the E-A method as a valuable complementary strategy for isolating high-affinity or rare binders.

Once again, thank you for your highly constructive comments, which have enhanced the rigor and value of our manuscript.

Reviewer #2

Comments on the Revised Manuscript:

The authors are again thanked for their revisions of the manuscript to address criticisms raised in the first and second round of review. While the experimental additions to the manuscript seem to be helpful, particularly the NGS data that directly assesses the polyclonality of their STAR library, the writing (and in particular the new edits) are not very good and would need to be revised extensively to make the paper more readable. The NGS observations are especially poorly reported, both methodologically and in reporting of results. The data needs to be more simply analyzed and presented.

Response:

We sincerely thank you for acknowledging our revisions made in response to previous reviews. We truly value your constructive and detailed feedback, which has been key to improve the quality of our work.

We are particularly grateful for your positive note on the experimental additions, especially the NGS data, which we agree that provide valuable insights into the polyclonality of the STAR library.

To address these points, we will undertake a comprehensive revision of the manuscript with a focus on:

- Improving the overall writing clarity and logical flow, especially in the newly added and revised sections.
- Restructuring and simplifying the presentation of the NGS methodology and results, ensuring the data analysis is communicated in an accessible manner.
- Enhancing the visual presentation of the NGS data, where appropriate, to support better interpretation and readability.

We are committed to revising the manuscript extensively in line with your suggestions and will ensure the final version meets the expected standards of clarity and scientific presentation.

Thank you once again for your time, expertise, and thoughtful criticism, all of which have contributed significantly to strengthening our paper.

Point-by-Point Responses:

Comment #1:

The methods section for the NGS work is lacking in important details. What primers were used to amplify the library? Were these focused only on the VHH domain? These primers should be provided and much more detail on how the NGS was performed needs to be provided.

Response:

Thank you for raising these points regarding the NGS methods section. We agree that the details provided were insufficient and appreciate your specific guidance on what needs to be clarified.

In response to your comment, we will revise the Methods section to include:

- The specific primer sequences used for library amplification.
- Confirmation that they specifically target the VHH domain.
- A more detailed NGS protocol.

These additions will be integrated into the revised manuscript to ensure full transparency and reproducibility of the NGS experiments.

Comment #2:

The bioinformatic analysis of NGS data needs to be improved. While the raw diversity of the library provides some insight, the nature of antibodies requires more specific tools. Authors should focus on a phylogenetic analysis of the CDR3 domains to determine the number of unique antibody families present in the data.

Response:

Thank you for this constructive suggestion. We agree that a phylogenetic analysis focused on CDR3 domains would provide deeper insight into the antibody families present in our library.

In the revised manuscript, we will:

- Perform and include a phylogenetic analysis of CDR3 regions from the NGS

data.

- Report the number of unique antibody families identified.

We appreciate your guidance in strengthening the bioinformatic rigor of this study.

Comment #3:

The explanation of the NGS data in the text and the supplemental is too technical. The language should be more simplified, focusing on number of unique CDR3 families.

Response:

Thank you for your valuable feedback. We agree that the presentation of the NGS data can be made more accessible.

In response, we will:

- Simplify the language in both the main text and supplementary materials.
- Refocus the narrative to highlight the number of unique CDR3 families identified.
- Ensure technical details are presented clearly but concisely.

We appreciate your guidance in improving the clarity of our manuscript.

Comment #4:

In the introduction, some discussion of previous CAR library work should be included, such as the work by Alonso-Camino (https://pmc.ncbi.nlm.nih.gov/articles/PMC4817937/) and more recent work. The current introduction gives a false idea that similar CAR-cell library selection is not demonstrated, which is certainly not the case.

Response:

Thank you for this important suggestion. We agree that the introduction should properly acknowledge prior work on CAR library screening.

In the revised manuscript, we will:

- Add discussion of previous studies, including the work by Alonso-Camino et al..
- Incorporate relevant recent advances in CAR library methodologies.
- Clarify how our approach builds upon and differs from existing strategies.

We appreciate your guidance in ensuring a more accurate and comprehensive literature context.

Comment #5:

Following on the above comment, comparison between STAR E-A screening and what has been reported by others is not very well discussed. A quick search yielded more than 6 papers that have used cell library selection of CARs in the past 5 years.

Response:

Thank you for raising this important point. We agree that a more thorough comparison of STAR E-A screening with existing CAR cell library methods is needed.

In response, we will:

- Conduct a comprehensive and systematic review of the existing literature on CAR cell libraries.
- Add a dedicated discussion section to provide a detailed comparative analysis between the E-A cellular screening approach and current technologies. This will allow us to clearly articulate the positioning of our STAR E-A platform within the existing methodological landscape.

We appreciate your guidance in strengthening the contextual framing of our work.

Comment #6:

The most impactful element of the paper is the discovery of P53-mut targeting STARs, but this is only briefly discussed. What will you do with these? What would be needed for next steps of development.

Response:

Thank you for highlighting this critical point. We agree that the discussion on the P53-mutant-targeting STARs requires expansion. In the revised manuscript, we will enhance the discussion section to include:

- Further elaboration on the functional significance and therapeutic potential of the identified P53-mutant-specific STARs.
- Discussion of potential research plans for further development and application of these findings.

We appreciate your guidance in strengthening the scientific impact and translational relevance of this work.

Comment #7:

Line 243 - How were the pre-screened VHH library converted into STAR-JC5 cells?

What number of cells was used at what MOI? This information would be helpful to understand why the cell conversion was such a bottleneck for library construction.

Response:

Thank you for raising this important technical question. We recognize that the details on the cell conversion process were insufficiently described.

In the revised Methods section, we will provide the following information:

- A step-by-step description of the VHH-to-STAR-JC5 conversion protocol.
- The exact cell numbers used at each stage.
- The MOI values applied during viral transduction.

These details will help clarify why the cell conversion step represented a key bottleneck in library construction.

We appreciate your guidance in improving the technical clarity of our manuscript.

Comment #8:

Line 248 to 256 - Why is the remarkable enrichment not simply a technical artifact relating to the process of cellular transduction of the STAR library? This is very poorly explained.

Response:

Thank you for this critical observation. We agree that the explanation regarding the potential influence of technical artifacts in the enrichment process needs substantial clarification and more careful framing.

In the revised manuscript, we will strengthen this section by providing a clearer and more rigorous discussion that directly addresses the distinction between true biological enrichment and potential technical effects arising from the cellular transduction process.

We appreciate your guidance in ensuring the robustness and clarity of our technical interpretations.

Reviewer #3

Comments on the Revised Manuscript:

The authors have made substantial additional changes to the paper to address some remaining shortcomings and to provide more detail and clarity.

Our Response:

Thank you for your continued feedback and for acknowledging our revisions. We are pleased to note that the additional changes have successfully addressed the remaining concerns and improved the paper's detail and clarity.

Once again, we sincerely appreciate the time and constructive input you have dedicated to this work.

28th Jan 2026

Dear Prof. Lin,

Thank you for the submission of your manuscript to EMBO Molecular Medicine, and please accept my apologies for the delay in getting back to you during this busy time of the year.

You submitted your manuscript to EMM following post-review rejection at another journal. While we recognized that the initial referees had remaining minor concerns on data and text presentation, we found your revised manuscript interesting and within the scope of EMBO Molecular Medicine, and sent it to a single advisor for evaluation, together with your point-by-point rebuttal letter to the referees' concerns.
This advisor has now gotten back to us, and I am glad to say that they support publication of your work:

"I fully endorse its publication in its present form. In short, the authors went to great lengths to address the concerns raised by the reviewers. Furthermore, I consider the content highly valuable for the community. The science is in my view sound."

We would therefore welcome the submission of your manuscript revised as indicated in your rebuttal.

When submitting your revised manuscript, please carefully review the instructions that follow below. We perform an initial quality control of all revised manuscripts before re-review; failure to include requested items will delay the evaluation of your revision.

1) A .docx formatted version of the manuscript text (including legends for main figures, EV figures and tables). Please make sure that the changes are highlighted to be clearly visible.

2) Individual production quality figure files as .eps, .tif, .jpg (one file per figure). For guidance, download the 'Figure Guide PDF': https://media.springernature.com/original/springer-cms/rest/v1/content/27825798/data/v1.

3) A .docx formatted letter INCLUDING the reviewers' reports and your detailed point-by-point responses to their comments. As part of the EMBO Press transparent editorial process, the point-by-point response is part of the Review Process File (RPF), which will be published alongside your paper.

4) A complete author checklist, which you can download from our author guidelines. Please insert information in the checklist that is also reflected in the manuscript. The completed author checklist will also be part of the RPF.

5) Please note that all corresponding authors are required to supply an ORCID ID for their name upon submission of a revised manuscript.

6) It is mandatory to include a 'Data Availability' section after the Materials and Methods. Before submitting your revision, primary datasets produced in this study need to be deposited in an appropriate public database, and the accession numbers and database listed under 'Data Availability'. Please remember to provide a reviewer password if the datasets are not yet public.

In case you have no data that requires deposition in a public database, please state so in this section. Note that the Data Availability Section is restricted to new primary data that are part of this study.

7) For data quantification: please specify the name of the statistical test used to generate error bars and P values, the number (n) of independent experiments (specify technical or biological replicates) underlying each data point and the test used to calculate p-values in each figure legend. The figure legends should contain a basic description of n, P and the test applied. Graphs must include a description of the bars and the error bars (s.d., s.e.m.).

8) At EMBO Press we ask authors to provide source data for the main manuscript figures. You will receive a separate email with instructions for providing source data with your revised manuscript, including how to upload and organize the files.

9) Our journal encourages inclusion of *data citations in the reference list* to directly cite datasets that were re-used and obtained from public databases. Data citations in the article text are distinct from normal bibliographical citations and should directly link to the database records from which the data can be accessed. In the main text, data citations are formatted as follows: "Data ref: Smith et al, 2001" or "Data ref: NCBI Sequence Read Archive PRJNA342805, 2017". In the Reference list, data citations must be labeled with "[DATASET]". A data reference must provide the database name, accession number/identifiers and a resolvable link to the landing page from which the data can be accessed at the end of the reference.

10) We replaced Supplementary Information with Expanded View (EV) Figures and Tables that are collapsible/expandable online. A maximum of 5 EV Figures can be typeset. EV Figures should be cited as 'Figure EV1, Figure EV2" etc... in the text and

their respective legends should be included in the main text after the legends of regular figures.

- For the figures that you do NOT wish to display as Expanded View figures, they should be bundled together with their legends in a single PDF file called *Appendix*, which should start with a short Table of Content. Appendix figures should be referred to in the main text as: "Appendix Figure S1, Appendix Figure S2" etc.

- Additional Tables/Datasets should be labeled and referred to as Table EV1, Dataset EV1, etc. Legends have to be provided in a separate tab in case of .xls files. Alternatively, the legend can be supplied as a separate text file (README) and zipped together with the Table/Dataset file.

11) The paper explained: EMBO Molecular Medicine articles are accompanied by a summary of the articles to emphasize the major findings in the paper and their medical implications for the non-specialist reader. Please provide a draft summary of your article highlighting
- the medical issue you are addressing,
- the results obtained and
- their clinical impact.

This may be edited to ensure that readers understand the significance and context of the research. Please refer to any of our published articles for an example.

12) Author contributions: You will be asked to provide CRediT (Contributor Role Taxonomy) terms in the submission system. These replace a narrative author contribution section in the manuscript.

13) A "Disclosure and Competing Interests Statement" should be provided in the main text.

14) Every published paper includes a 'Synopsis' to further enhance discoverability. Synopses are displayed on the journal webpage and are freely accessible to all readers. They include a short stand first (maximum of 300 characters, including space) as well as 2-5 one-sentences bullet points that summarizes the paper. Please write the bullet points to summarize the key NEW findings. They should be designed to be complementary to the abstract - i.e. not repeat the same text. We encourage inclusion of key acronyms and quantitative information (maximum of 30 words / bullet point). Please use the passive voice. Please attach these in a separate file or send them by email, we will incorporate them accordingly.

Please also suggest a striking image or visual abstract to illustrate your article as a PNG file 550 px wide x 300-600 px high.

15) Include a Reagents and Tools Table as part of the Methods section, which can be downloaded from our author guidelines.

16) As part of the EMBO Publications transparent editorial process initiative (see our Editorial at http://embomolmed.embopress.org/content/2/9/329), EMBO Molecular Medicine will publish online a Review Process File (RPF) to accompany accepted manuscripts.
In the event of acceptance, this file will be published in conjunction with your paper and will include the anonymous referee reports, your point-by-point response and all pertinent correspondence relating to the manuscript. Let us know whether you agree with the publication of the RPF and as here, if you want to remove or not any figures from it prior to publication.
Please note that the Authors checklist will be published at the end of the RPF.

EMBO Molecular Medicine has a "scooping protection" policy, whereby similar findings that are published by others during review or revision are not a criterion for rejection. Should you decide to submit a revised version, I do ask that you get in touch after three months if you have not completed it, to update us on the status.

I look forward to receiving your revised manuscript.

Yours sincerely,

Lise Roth

Lise Roth, PhD
Senior Editor
EMBO Molecular Medicine

The authors addressed the editorial issues.

22nd Apr 2026

Dear Prof. Lin,

Thank you for submitting your revised study to EMBO Molecular Medicine. A few editorial matters remain to be addressed before we can accept your manuscript:

1/ Manuscript text:
- Please remove the highlighted text and only keep in track changes mode any new modification.
- Please correct the order and headings of the sections in the manuscript text to: Abstract / The Paper Explained / Introduction / Results / Discussion / Methods / Data Availability / Acknowledgements / Disclosure and Competing Interests Statement / References / Figure Legends / Expanded View Figure Legends.
- Please remove results/data not shown. The journal does not permit citation of "Data not shown". All data referred to in the paper should be displayed in the main or Expanded View figures.
- Methods:
o Cells: please indicate whether the cells were authenticated and tested for mycoplasma contamination.
o Animal experiments: please provide the origin of the mice, housing and husbandry conditions. Provide reference number for approval from authority grantic ethics approval.
o Please clarify whether the library generation was outsourced, or provide additional information on animals.
o Statistics: please provide a statement on sample size, inclusion/exclusion criteria, blinding and randomization.
- Data Availability: Please remove "The data that support the findings of this study are available from the corresponding author upon request." Please make sure the deposited datasets are accessible to the public.
- Acknowledgements: Funding listed in the acknowledgements and in the submission system should match, please check whether annual fund of Tsinghua-Peking Center for Life Sciences and Changping Laboratory should be added to the list of funders in our system.
- Author contributions: Please remove from the manuscript text and provide CRediT (Contributor Role Taxonomy) terms in the submission system.
- Disclosure and competing interests statement: please indicate that author Xin Lin is employed by BriSTAR Immunotech.
- References: please correct to alphabetical order and 10 author names listed before et al.

2/ Figures:
- Please provide individual data points where possible.
- Please upload the EV figures and EV tables as separate files, one per figure/table.
- Figure EV5: panel A can be removed, as there is only one panel.
- Please note that figure reuse may be allowed, but should be mentioned in the figure legend (Fig. 2B and Fig. EV2D).
- Please address the query from our data editors in the figure legends:
Please note that the exact p values are not provided in the legends of figures 4C, G; 7C, F; 8F

3/ Checklist:
- Newly created materials: please note that it is understood that by publishing a paper in an EMBO Press journal, the authors agree to make available to colleagues in academic research all new reagents, including organisms (or means to produce them), viruses, cells, nucleic acids and antibodies, that were used in the research reported and that are not available from public repositories or commercial suppliers. Materials must be made available at a reasonable cost that reflects production and distribution (https://link.springer.com/partners/embo-press/editorial-policies#Materials%20and%20data%20sharing)
- Please check that the subsection "microbes" applies to your study.

4/ Source Data:
- Please check the source data provided for Figure 3F, as two columns appear almost identical (please see document attached).
- Please note that the files for Figure 4D/4H are empty.

5/ Thank you for providing The paper explained. Please include it in the manuscript text, and change the paragraph headings to Problem/Results/Impact.

6/ I introduced minor edits in your synopsis to match our format, please let me know if you agree or amend as you see fit:

STAR-T cells exhibit antigen-dependent receptor endocytosis coupled with activation, enabling a functional screening platform that directly links antibody discovery to T-cell activity for rapid isolation of therapeutic TCR-like antibodies against both surface antigens and HLA-presented neoantigens.

- Antigen engagement in STAR-T cells triggers synchronous receptor endocytosis and CD69 upregulation, which together form a dual-parameter Endocytosis-Activation (E-A) functional readout.

- E-A functional screening identified CD22-specific nanobodies from a >10^6-diversity T-cell library, with lead candidate CD22-1 VHH exhibiting nanomolar affinity and potent in vivo anti-tumor efficacy in xenograft models.
- The platform identified multiple P53R175H/HLA-A*02:01-specific VHHs, with P53-1 VHH STAR-T cells demonstrating efficient tumor suppression in both KMS26 and K562 tumor models.
- E-A screening outperformed conventional phage display, achieving >10-fold enrichment of functional clones while eliminating clones defective for mammalian expression.
- Identified nanobodies were directly reformatted into STARs, CARs (BBz/28z), and bispecific antibodies, all retaining potent antigen-specific cytotoxicity in vitro and in vivo.

Thank you for providing a nice visual abstract. Please correct the dimensions to 550 px wide x 300 px high, and make sure that the text remains legible. You may have to simplify the overall schematic.

7/ As part of the EMBO Publications transparent editorial process initiative (see our Editorial at http://embomolmed.embopress.org/content/2/9/329), EMBO Molecular Medicine will publish online a Review Process File (RPF) to accompany accepted manuscripts.
This file will be published in conjunction with your paper and will include the anonymous referee reports, your point-by-point response and all pertinent correspondence relating to the manuscript. Let us know whether you agree with the publication of the RPF and as here, if you want to remove or not any figures from it prior to publication.
Please note that the Authors checklist will be published at the end of the RPF.

I look forward to receiving your revised manuscript.

Yours sincerely,

Lise Roth

Lise Roth, PhD
Senior Editor
EMBO Molecular Medicine

The authors addressed the remaining editorial issues.

13th May 2026

Dear Prof. Lin,

We are pleased to inform you that your manuscript is accepted for publication and will be sent to our publisher to be included in the next available issue of EMBO Molecular Medicine once we receive the last figure files.

Your manuscript will be processed for publication by EMBO Press. It will be copy edited and you will receive page proofs prior to publication. Please note that you will be contacted by Springer Nature Author Services to complete licensing and payment information.

You may qualify for financial assistance for your publication charges - either via a Springer Nature fully open access agreement or an EMBO initiative. Check your eligibility: https://link.springer.com/journal/44321/how-to-publish-with-us

Should you be planning a Press Release on your article, please get in contact with embo_production@springernature.com as early as possible in order to coordinate publication and release dates.

If you have any questions, please do not hesitate to contact the Editorial Office. Thank you for your contribution to EMBO Molecular Medicine.

Yours sincerely,

Lise Roth

Lise Roth, Ph.D
Senior Editor
EMBO Molecular Medicine

>>> Please note that it is EMBO Molecular Medicine policy for the transcript of the editorial process (containing referee reports and your response letter) to be published as an online supplement to each paper. If you do NOT want this, you will need to inform the Editorial Office via email immediately. More information is available here: https://link.springer.com/partners/embo-press/editorial-policies#Peer%20review

Study protocol	Information included in the manuscript?	In which section is the information available? (Reagents and Tools Table, Materials and Methods, Figures, Data Availability Section)
If study protocol has been **pre-registered, provide DOI in the manuscript**. For clinical trials, provide the trial registration number **OR** cite DOI.	Not Applicable	
Report the **clinical trial registration number** (at ClinicalTrials.gov or equivalent), where applicable.	Not Applicable	

Laboratory protocol	Information included in the manuscript?	In which section is the information available? (Reagents and Tools Table, Materials and Methods, Figures, Data Availability Section)
Provide DOI OR other citation details if **external detailed step-by-step protocols** are available.	Not Applicable	

Experimental study design and statistics	Information included in the manuscript?	In which section is the information available? (Reagents and Tools Table, Materials and Methods, Figures, Data Availability Section)
Include a statement about **sample size** estimate even if no statistical methods were used.	Yes	Methods
Were any steps taken to minimize the effects of subjective bias when allocating animals/samples to treatment (e.g. **randomization procedure**)? If yes, have they been described?	Yes	Methods
Include a statement about **blinding** even if no blinding was done.	Yes	Methods
Describe **inclusion/exclusion criteria** if samples or animals were excluded from the analysis. Were the criteria pre-established?<br><br>If sample or data points were omitted from analysis, report if this was due to attrition or intentional exclusion and provide justification.	Yes	Methods
For every figure, are **statistical tests** justified as appropriate? Do the data meet the assumptions of the tests (e.g., normal distribution)? Describe any methods used to assess it. Is there an estimate of variation within each group of data? Is the variance similar between the groups that are being statistically compared?	Yes	Methods

Sample definition and in-laboratory replication	Information included in the manuscript?	In which section is the information available? (Reagents and Tools Table, Materials and Methods, Figures, Data Availability Section)
In the figure legends: state number of times the experiment was **replicated** in laboratory.	Yes	Figures, Methods
In the figure legends: define whether data describe **technical or biological replicates**.	Yes	Figures, Methods

Ethics

Ethics	Information included in the manuscript?	In which section is the information available? (Reagents and Tools Table, Materials and Methods, Figures, Data Availability Section)
Studies involving **human participants**: State details of **authority granting ethics approval** (IRB or equivalent committee(s), provide reference number for approval.	Not Applicable	
Studies involving **human participants**: Include a statement confirming that **informed consent** was obtained from all subjects and that the experiments conformed to the principles set out in the WMA Declaration of Helsinki and the Department of Health and Human Services Belmont Report.	Not Applicable	
Studies involving **human participants:** For publication of **patient photos**, include a statement confirming that consent to publish was obtained.	Not Applicable	
Studies involving experimental **animals**: State details of **authority granting ethics approval** (IRB or equivalent committee(s), provide reference number for approval. Include a statement of compliance with ethical regulations.	Yes	Methods
Studies involving **specimen and field samples:** State if relevant **permits** obtained, provide details of authority approving study; if none were required, explain why.	Not Applicable	

Dual Use Research of Concern (DURC)	Information included in the manuscript?	In which section is the information available? (Reagents and Tools Table, Materials and Methods, Figures, Data Availability Section)
Could your study fall under dual use research restrictions? Please check biosecurity documents and list of **select agents and toxins** (CDC): https://www.selectagents.gov/sat/list.htm	Not Applicable	
If you used a select agent, is the security level of the lab appropriate and reported in the manuscript?	Not Applicable	
If a study is subject to dual use research of concern regulations, is the name of the **authority granting approval and reference number** for the regulatory approval provided in the manuscript?	Not Applicable	

Reporting

The MDAR framework recommends adoption of discipline-specific guidelines, established and endorsed through community initiatives. Journals have their own policy about requiring specific guidelines and recommendations to complement MDAR.

Adherence to community standards	Information included in the manuscript?	In which section is the information available? (Reagents and Tools Table, Materials and Methods, Figures, Data Availability Section)
State if relevant guidelines or checklists (e.g., **ICMJE, MIBBI, ARRIVE, PRISMA**) have been followed or provided.	Not Applicable	
For **tumor marker prognostic studies,** we recommend that you follow the **REMARK** reporting guidelines (see link list at top right). See author guidelines, under 'Reporting Guidelines'. Please confirm you have followed these guidelines.	Not Applicable	
For **phase II and III randomized controlled trials**, please refer to the **CONSORT** flow diagram (see link list at top right) and submit the CONSORT checklist (see link list at top right) with your submission. See author guidelines, under 'Reporting Guidelines'. Please confirm you have submitted this list.	Not Applicable	

Data Availability

Data availability	Information included in the manuscript?	In which section is the information available? (Reagents and Tools Table, Materials and Methods, Figures, Data Availability Section)
Have **primary datasets** been deposited according to the journal's guidelines (see 'Data Deposition' section) and the respective accession numbers provided in the Data Availability Section?	Yes	Data availability
Were **human clinical and genomic datasets** deposited in a public access-controlled repository in accordance to ethical obligations to the patients and to the applicable consent agreement?	Not Applicable	
Are **computational models** that are central and integral to a study available without restrictions in a machine-readable form? Were the relevant accession numbers or links provided?	Not Applicable	
If publicly available data were reused, provide the respective **data citations in the reference list**.	Not Applicable	
